# Ensemble clustering: A practical tutorial

**DOI:** 10.3758/s13428-026-03158-y

**Published:** 2026-09-03

**Authors:** Caroline X. Gao, Shengqi Wang, Ye Zhu, Myriam Ziou, Shu Mei Teo, Catherine L. Smith, Derek Chiu, Aline Talhouk, Mengmeng Wang, Wenhua Yu, Sue M. Cotton, Dominic Dwyer

**Affiliations:** 1 Centre for Youth Mental Health, The University of Melbourne, Parkville, VIC Australia; 2 Orygen, Parkville, VIC Australia; 3 Department of Epidemiology and Preventative Medicine, School of Public Health and Preventive Medicine, Monash University, Melbourne, VIC Australia; 4 School of Energy & Environment, Southeast University, Nanjing, 210096 China; 5 Centre for Cyber Resilience and Trust, Deakin University, Victoria, 3125 Australia; 6 Menzies Institute for Medical Research, University of Tasmania, Hobart, TAS Australia; 7 Department of Breast and Molecular Oncology, BC Cancer Agency, Vancouver, BC Canada; 8 Department of Obstetrics and Gynecology, University of British Columbia, Vancouver, BC Canada; 9 School of Psychological Sciences, Monash University, Clayton, VIC Australia

**Keywords:** Ensemble clustering, Ensemble learning, Clustering, Cluster analysis, Machine learning, Unsupervised learning

## Abstract

Cluster analysis is an explorative analytical method, serving as a critical tool in psychology, psychiatry, and related fields to map heterogeneous data into meaningful subgroups. Despite their extensive historical use, traditional clustering techniques suffer from a lack of stability, robustness, and generalisability. These issues stem from the inherent difficulties of the clustering optimisation problem as well as the stochastic nature of algorithm optimisers. To address these challenges, we demonstrate the use of methods utilising ensemble learning techniques to combine clustering results from different algorithms, model specifications, and/or sampled sub-datasets to form a single, more reliable consensus of clustering solutions. We detail ensemble clustering principles and variations in base clustering generation models and ensemble methods. More importantly, detailed introductions in existing R libraries and practical examples using R code are provided to guide users in both implementing and optimising ensemble clustering models. As a practical tutorial, we then include simulation studies of real-world data to demonstrate the substantial benefit of ensemble clustering compared with single-run clustering models. The resources presented here will enable researchers to apply advanced clustering techniques to decompose heterogeneous and complex psychological data into stable subgroups.

**Supplementary Information:**

The online version contains supplementary material available at https://doi.org/10.3758/s13428-026-03158-y.

## Introduction

Categorising groups of similar objects, elements, or individuals is a cornerstone of scientific advancement, but the process can be influenced by human bias (Yoon, 2009). Statistical clustering arising in the mid-twentieth century promised to reduce this bias by automatically grouping data points based on their similarity (see terminology definition in Table S1).

The term “cluster analysis” was first introduced in psychology, dating back to 1939, when R.C. Tryon (1939) introduced it to identify “clusters” of variables. This approach remains a foundational method for exploring symptom clusters and contributing to disease nosology (Forbes et al., 2021, 2024). Over time, the application of clustering models in health shifted towards exploring the heterogeneity of individuals rather than just variables, enabling researchers to identify distinct subgroups of people based on shared behavioural, biological, or clinical characteristics (Cattell, 1943).

To date, clustering models are widely used in psychology, psychiatry, and other related fields for a range of purposes, such as understanding the heterogeneity of disorders using data from clinical profiles, genetic risks, brain pathophysiology, and blood markers (Beijers et al., 2019; Dwyer et al., 2022; Marquand et al., 2016); evaluating treatment response heterogeneity (Bondar et al., 2020; Case et al., 2011); identifying clinical complexity profiles for service optimisation (Cotton et al., 2022; Gao et al., 2023a, 2023b); exploring symptom profiles (Bondar et al., 2020; Oladottir et al., 2022); extracting biological/pathological markers (Franklyn et al., 2022; Sæther et al., 2023); and mapping longitudinal patterns (Hall et al., 2019; Pierce et al., 2021). Over 60,000 articles have been published to date and more than 5,000 in 2024 alone.^1^

Despite this remarkable volume of research, their impact on research, practice, and policy remains severely limited (Feczko & Fair, 2020; Milligan & Cooper, 1987; Thorndike, 1953). Several core issues hinder the greater impact of clustering studies, such as a lack of clustering stability (the ability of results to remain consistent under repeated runs), robustness (resilience to noise in the data), and generalisability (applicability to different datasets and conditions); see detailed discussion elsewhere (Gao et al., 2023a, 2023b). Commonly used methods in psychology and psychiatry to address these issues have been ineffective (e.g., multiple start-points for *k*-means), evident from their statistical sensitivity to distributions of data points, minor data perturbations, and choice of clustering algorithms (Kleinberg, 2002; Milligan, 1980; Shireman et al., 2016; Steinley, 2003; Wolpert & Macready, 1997). Such failure is theorised to be related to an impossibility of simultaneously optimising across multiple criteria (e.g., the “***impossibility theorem***”, no clustering method can satisfy all desirable properties at once) (Kleinberg, 2002) in the context of a problem that does not have an exact solution (Steinley, 2003). Most algorithms rely on assumptions and trade-offs between different desirable properties.

One major downstream implication is the replication crisis. Identified subtypes often vary substantially across studies; and when broadly similar patterns emerge, they frequently reflect arbitrary cut-offs along one severity dimension rather than providing meaningful insight into true heterogeneity (Brucar et al., 2023; Yu et al., 2026). For example, despite decades of research, current evidence does not support reproducible, biologically valid, and clinically meaningful neuroimaging subtypes for mental illnesses that are broadly agreed upon (Beijers et al., 2019; Brucar et al., 2023; Yu et al., 2026).

These issues that continue to hamper research in psychology and psychiatry have been more effectively addressed in other medical fields (e.g., oncology and tumour subtyping), resulting in expanded impact and real-world translation (Fred & Jain, 2002, 2005; Monti et al., 2003; Polikar, 2006; Strehl & Ghosh, 2002; Topchy et al., 2004). Ensemble clustering methods^2^ are the primary driver, which combine clustering solutions from different data distributions, clustering algorithms, and optimisations before reaching a consensus on the final solution (Fred & Jain, 2002, 2005; Monti et al., 2003; Polikar, 2006; Strehl & Ghosh, 2002; Topchy et al., 2004). As a meta-method, ensemble clustering models not only offer more stable and robust solutions but also help in determining the optimal number of clusters from a stability perspective (Monti et al., 2003; Șenbabaoğlu et al., 2014). Another advantage of ensemble clustering is that it can facilitate the detection of complex decision boundaries that may not have been present in the underlying single-run clustering solutions (Brière et al., 2021; Fu et al., 2020; Jain, 2010; Rappoport & Shamir, 2018; Xu et al., 2013; Yang & Wang, 2018).

Despite the availability of generalist methodological reviews (Boongoen & Iam-On, 2018; Golalipour et al., 2021; Nie et al., 2023; Vega-Pons & Ruiz-Shulcloper, 2011) and tutorials designed for machine learners and statisticians (Abbasi et al., 2019; Chiu & Talhouk, 2018), there is a critical lack of conceptual and practical guides to ensemble clustering specifically designed for an audience who are not familiar with advanced machine learning and statistical models, particularly in psychology and psychiatry, where clustering methods are frequently applied. This tutorial aims to fill this gap by providing a review of theoretical principles as well as practical implementations in ensemble clustering methods. Focusing on the R programming language, which is commonly used in psychology and psychiatry research, this practical tutorial presents (1) a brief overview of relevant clustering methods, (2) an introduction to ensemble clustering principles with a summary of R packages, (3) examples of applications with detailed steps and code in the R language, and (4) a simulation study comparing ensemble clustering with other models to emphasise the urgent need for the use of these methods in psychology and psychiatry. This tutorial also requires familiarity with basic clustering methods; therefore, we recommend readers consult our earlier methodological overview (Gao et al., 2023a, 2023b), which provides a detailed description of different clustering models as well as applications in mental health research.

## Types of clustering methods

**Fig. 1:**
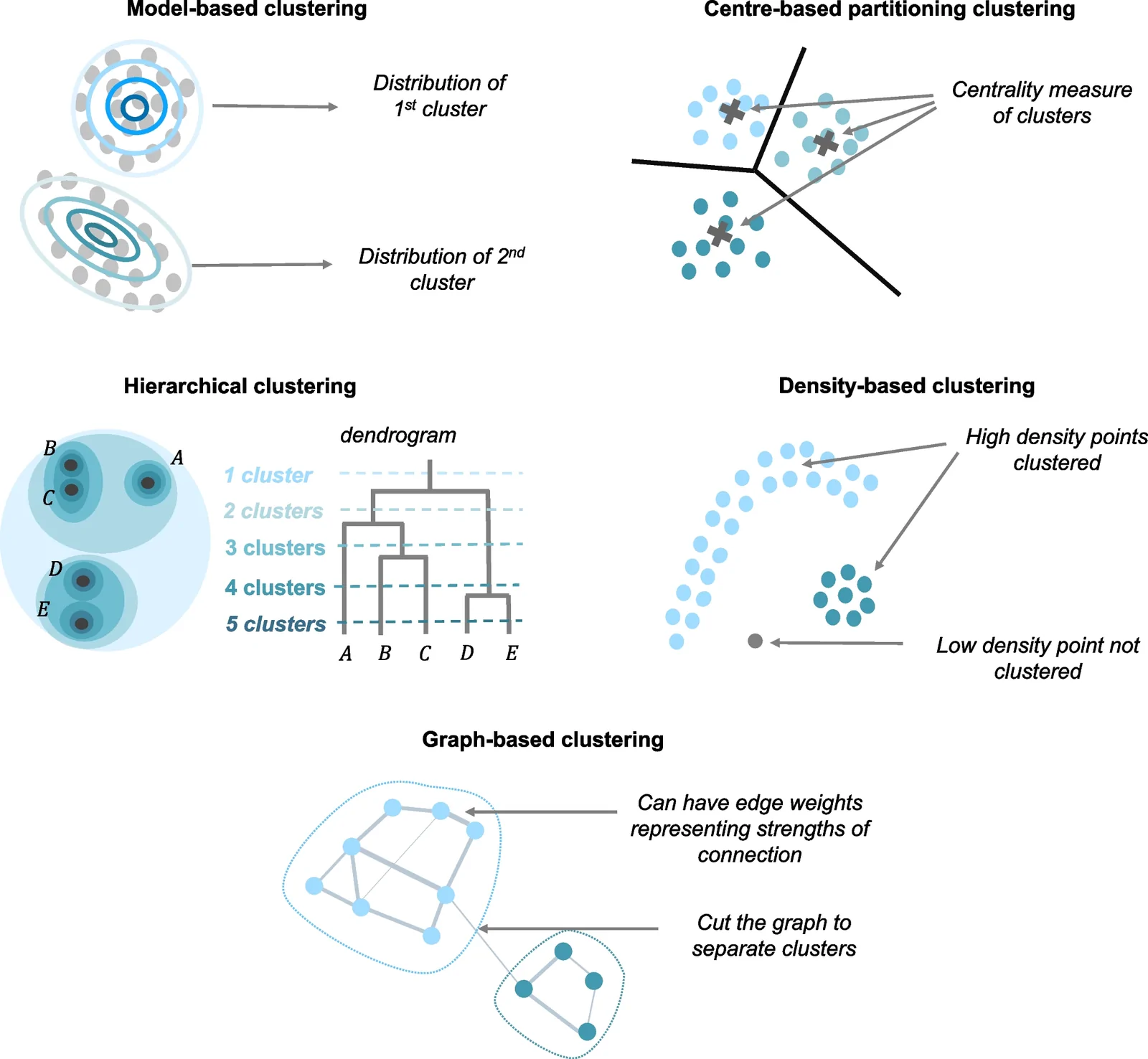
Illustration of main types of clustering algorithms

Since the 1960 s, numerous clustering algorithms have been developed. Here we provide a brief description of a few main models (see Fig. 1) to aid in understanding their use in ensemble clustering. The most common clustering method used in psychology and psychiatry is ***model-based clustering***, which assumes that the data originate from latent subpopulations, each having different distributions (e.g., finite mixture models such as ***latent class analysis [LCA]*** and ***latent profile analysis [LPA]***) (Clatworthy et al., 2005). Another popular family of methods is ***centre-based partitioning clustering*** (e.g., *k*-means), which iterates partitions subgroups based on distances between data points and cluster centres. A deterministic approach is ***hierarchical clustering,*** which constructs a tree of clusters (as a dendrogram) by either iteratively merging similar data points or dividing dissimilar subclusters.

Several more modern clustering methods have also been widely used. For example, ***density-based clustering*** is designed to address problems associated with outliers by forming clusters in high-density regions and excluding outliers in low-density regions. ***Graph-based clustering*** refers to a range of diverse methods designed to segment networks of interconnected elements (e.g., social networks) and includes the detection of groups of similar points as modules or spectra (e.g., ***spectral clustering***), or by considering pathways between network elements.

## Principles and processes of ensemble clustering

Ensemble clustering methods refer to ***meta-learning methods*** for pooling, aggregating, and integrating results from different clustering algorithms or different runs of the same algorithm (with different data, parameter settings, or initialisations) to form a consensus (i.e., a stable grouping pattern that recurs across clustering solutions, rather than complete agreement between them). Conceptually, they are analogous to ensemble methods in supervised machine learning, in which predictions from multiple models (or repeated runs of the same model) are combined to reduce sensitivity to the limitations of any single model, improve robustness and avoid overfitting (Polikar, 2006). In ensemble clustering, however, the goal is to improve the stability and reproducibility of subgroup identification rather than prediction accuracy.

**Fig. 2:**
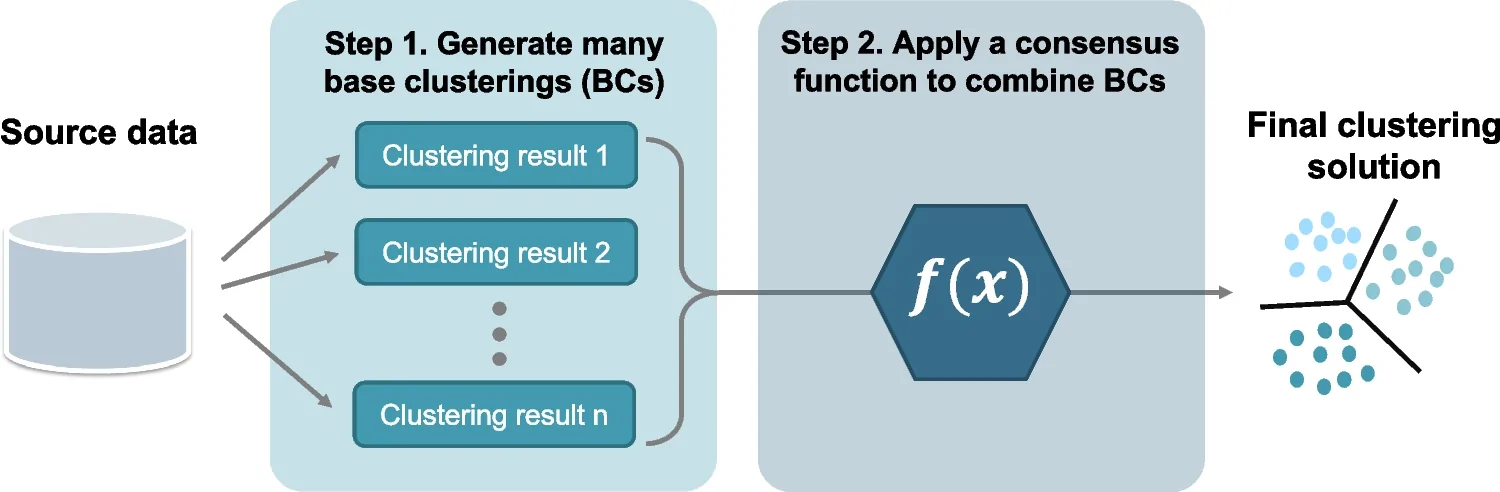
Common framework of ensemble clustering

Although many clustering ensemble methods have been proposed, they all follow a similar framework (see Fig. 2). Ensemble clustering generally involves two stages: generating base clustering (BCs, sometimes also referred to as base clusters) and obtaining consensus. The ensemble processes are commonly designed to achieve two goals: to obtain robust clustering solutions or to combine clustering results from different sources. The latter is known as multi-view ensemble clustering,^3^ where for example separate clustering solutions can be obtained from brain imaging, qualitative interviews, video or audio recordings, genetic data, and blood biomarkers, which can be integrated into a single solution (Gao et al., 2023a, 2023b). As the processes of multi-view ensemble clustering are slightly different, they are not the focus of this tutorial. Interested readers can find further information on multi-view clustering elsewhere (Chao et al., 2021; Fu et al., 2020; Yang & Wang, 2018; Zhang, 2022).

### Generating BCs

**Fig. 3:**
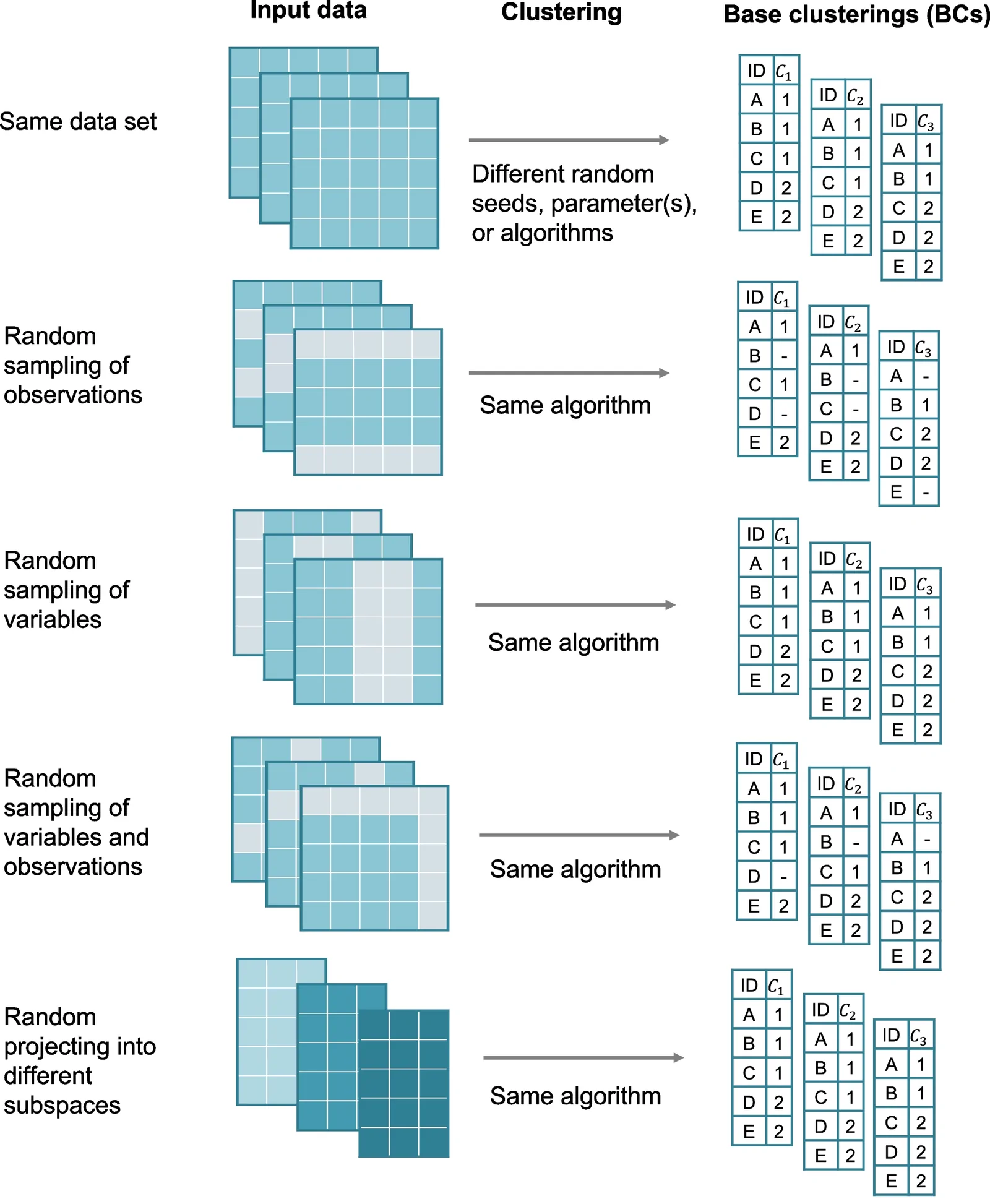
Simple illustration of main methods for generating diverse base clusters. *Note:* Blue colour indicates selected observations or variables; ${C}_{1}, {C}_{2}, \mathrm{a}\mathrm{n}\mathrm{d}\, {C}_{3}$ represent three base clusterings (BCs), each with two partitions labelled as 1 and 2

The relatively simple first step of ensemble clustering involves generating many different clustering solutions, known as BCs. BCs can be obtained by running different models (e.g., different random seeds, parameters, or algorithms) on the same data or the same model on different data (e.g., random samples of observations and/or variables, variables randomly projected to different subspaces) or combinations of different approaches (see Fig. 3). In practical tasks, random sampling of observations and/or variables is often used due to the ease of operationalisation and interpretation. Dimensionality reduction is often required prior to generating BCs, particularly with large numbers of variables or strong intercorrelations to improve the performance of BCs (Gao et al., 2023a, 2023b).

In general, BCs are recommended to be both diverse (presented with differences in clustering solutions) and of high quality (optimal at separating observations that can be measured using different criteria) (Fern & Lin, 2008; Kuncheva & Hadjitodorov, 2004; Li et al., 2019). Achieving these two objectives simultaneously is challenging because the models that increase diversity (e.g., aggressive sub‑sampling, randomising algorithmic) can also degrade the quality of individual partitions (e.g., some clustering models do not fit data well), and conversely, optimisation of quality often drives all BCs towards a similar solution, reducing diversity. However, their relative importance in enhancing ensemble clustering effectiveness is not universally agreed upon, making it a key domain of ongoing research in the field (Golalipour et al., 2021; Li et al., 2019).

### Achieving consensus

The second stage, obtaining consensus, however, is more complex than generating BCs, and various methods have been proposed. The simplest approach is to use a majority voting method among the partitions in BCs (Zhou & Tang, 2006). For instance, if BCs categorise observations into three clusters (e.g., low, medium, and high), the consensus classification for a specific observation would be the category to which the majority of BCs assign that observation. Clustering models normally return random cluster labels (e.g., the low, medium, and high groups in one model are labelled as first, second, and third, which can change to third, second, and first in another model). Therefore, BCs will need to be aligned or relabelled (similar clusters given the same label) before they can be combined (Zhou & Tang, 2006). However, when the BCs are very diverse or generated from a method such as ***random-k***, majority voting cannot be conducted.

**Fig. 4:**
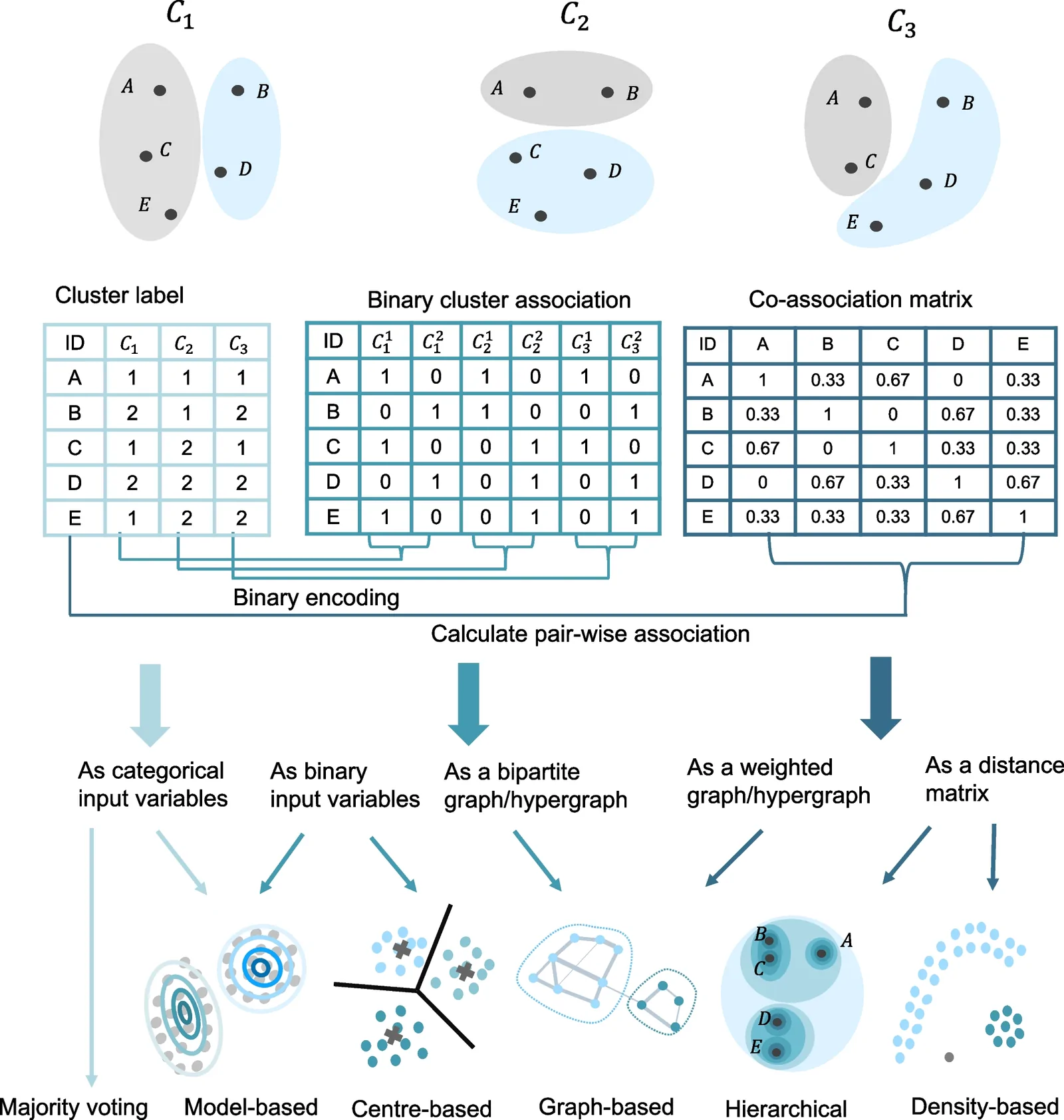
Simple illustration of several ensemble clustering methods. *Note:* Top panels illustrate three base clustering (BC) solutions derived from different algorithms or modelling conditions (${C}_{1}, {C}_{2}, \mathrm{a}\mathrm{n}\mathrm{d}\, {C}_{3}$) with two partitions (labelled as 1 and 2) for five data points. Middle panels represent possible cluster summarising matrices that can be used to integrate BC solutions as well as their associations (e.g., how binary cluster association can be derived from cluster labels). Bottom panels demonstrate possible modelling approaches to summarise cluster results and obtain the final ensemble clustering solution

To avoid the relabelling step, address the practical challenges of majority voting, and improve consensus quality, more complex methods are needed. A commonly applied technique is to first summarise BCs and then carry out a second-stage clustering model (see Fig. 4).

The cluster labels from BCs can be treated as categorical variables. Therefore, consensus can be obtained using a finite mixture model (Topchy et al., 2004; Yu et al., 2014) or other methods of clustering categorical variables such as ***k-mode*** (Chiu & Talhouk, 2018; Huang, 1997). The cluster labels can also be converted into a ***binary cluster association matrix*** using binary encoding (each cluster label is converted to multiple binary variables representing individual partitions), which can be used as input data for methods such as *k*-means, e.g., ***k-means-based consensus clustering (KCC)*** (Lin et al., 2023; Wu et al., 2015). The binary cluster association matrix can be transformed into a ***bipartite graph*** (a graph connecting clusters with observations; see Fig. S1 in Supplementary Material I) and consensus can be achieved with graph-based models (Yu et al., 2007). A ***hypergraph-partitioning algorithm (HGPA)***, for example, proposed by Strehl and Ghosh (2002) utilised this approach.

The cluster labels can also be summarised by ***pairwise associations*** between observations (e.g., a ***co-association matrix***, which measures how frequently pairs of data points are grouped together across BCs). The co-association matrix can be used directly as distance measures for a second-stage clustering model such as hierarchical clustering, which was one of the earliest and most widely employed methods, also known as ***evidence accumulation clustering (EAC)*** (Fred & Jain, 2002, 2005) or ***consensus clustering*** (Monti et al., 2003). The co-association matrix can also be converted to ***weighted graphs*** (nodes are the observations and the weights between two nodes are the corresponding values in the co-association matrix) or ***hypergraphs*** (observations represented by nodes connected by ***hyperedges*** representing the membership of the same cluster; see Fig. S1 in Supplementary Material I). Then the final consensus can be treated as a graph partitioning problem (Liu et al., 2015); for example, the ***cluster-based similarity partitioning algorithm (CSPA)*** applies a graph-based clustering model, METIS, on the co-association matrix (Strehl & Ghosh, 2002). As the co-association matrix only evaluates the similarity between pairs of observations but not between BCs, other similarity matrices have been proposed, e.g., ***connected-triple-based similarity (CTS***) proposed in the ***link-based clustering ensemble (LCE)*** (Iam-On & Garrett, 2010; Iam-On et al., 2011).

Several alternative consensus methods have been formulated. For instance, the ***meta-clustering algorithm (MCLA)*** involves consideration of each BC as a hypergraph. Its consensus function merges similar hyperedges (Strehl & Ghosh, 2002). Another approach for obtaining consensus from BCs is to estimate a mean (median) partition that minimises the disagreement of all BCs using methods such as aggregating partitions or refining cluster centroids (Bradley & Fayyad, 1998; Gionis et al., 2007; Jain, 2018). ***Adaptive clustering ensemble (ACE)*** is another method based on the binary clustering association matrix, which iteratively merges binary representations based on a similarity measure between clusters (Alqurashi & Wang, 2019). Recent developments also include ensemble clustering using deep learning (Cai et al., 2021; Ma et al., 2016). Detailed reviews and summaries of diverse ensemble clustering models are available in the machine-learning literature (Boongoen & Iam-On, 2018; Ghaemi et al., 2009; Golalipour et al., 2021; Vega-Pons & Ruiz-Shulcloper, 2011; Xanthopoulos, 2014).

### Choosing BC algorithm(s) and ensemble methods

BCs can be generated through a variety of sampling and modelling techniques, tailored to the specific characteristics and dimensions of the dataset (e.g., types and distributions of variables, number of observations) under consideration. Resampling of observations is commonly applied in larger samples, and variable or feature resampling strategies are particularly useful in high-dimensional settings. When choosing algorithms for BCs, it is important to consider the dataset’s specific characteristics, including data size, variable types, and the anticipated configurations of clusters. In practice, *k*-means is the most commonly used clustering algorithm due to its low computational demands; however, it is not without its limitations (e.g., assumption of spherical clusters and sensitivity to outliers). In psychological and mental health research, model-based clustering (e.g., LCA) may also be a strong candidate BC algorithm, as it can accommodate subgroups that plausibly arise from underlying generative processes. Multiple BC algorithms can also be used and tested to improve the robustness of the modelling approach.

The choice of ensemble methods can also depend on the features of the data. Majority voting is the earliest and most straightforward approach. However, it is most appropriate when BCs produce highly similar cluster assignment results. It also does not take full advantage of ensemble methods, i.e., integrating diverse results from base algorithms (weak learners) to achieve high performance (Polikar, 2006). The co-association matrix is the most commonly used method due to its interoperability and flexibility. When displayed using heatmaps, the co-association matrix can provide a direct visual presentation of cluster membership, which can be interpreted as relative stability when choosing the number of clusters (Monti et al., 2003). There are also flexible ways to decompose the co-association matrix for consensus (e.g., as a distance matrix or graph). However, it can be difficult to calculate with an increasing number of observations. This can be addressed by using the binary cluster association matrix with big data.

**Fig. 5:**
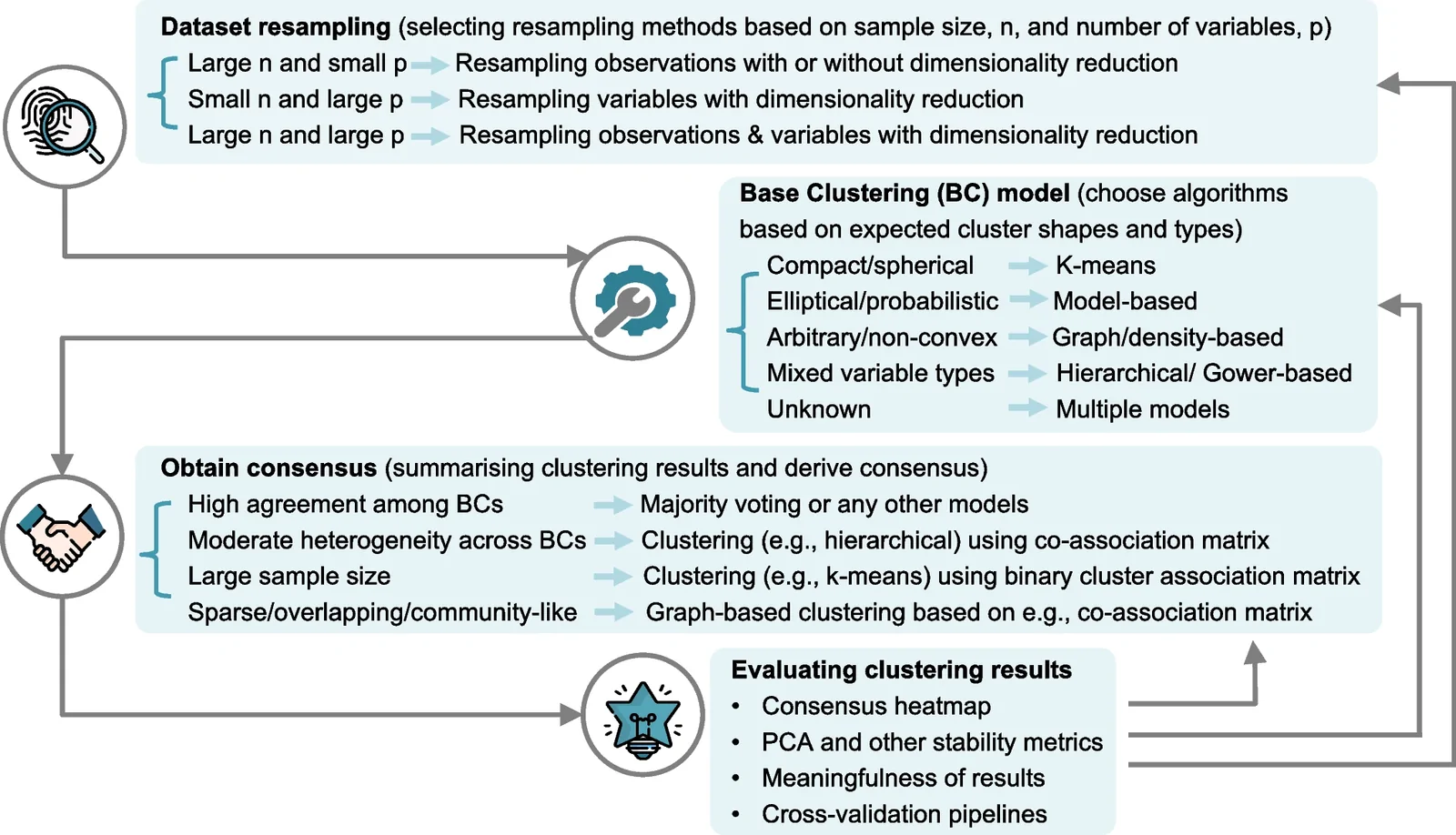
Simple illustration of advisory ensemble clustering workflow with general modelling considerations and recommendations

Although the literature is sparse on benchmarking different ensemble clustering algorithms, limited evidence suggests that the accuracy and robustness of most of these techniques are high and that differences may be due to specific characteristics (e.g., dimensionality, distribution, and size) of the data (Ghaemi et al., 2009; Wu et al., 2018). We provide high-level considerations and recommendations for establishing the whole ensemble clustering workflow in Fig. 5, with possible cross-validation (CV, a method that repeatedly applies a model to resampled or partitioned data to improve its generalisability) procedures outlined in Fig. S3.

### Optimising parameters

Substantial research effort has been focused on improving the performance of ensemble models, particularly by improving the quality and diversity of BCs (Abbasi et al., 2019; Berikov, 2014; Zhang, 2022). Diversity of BCs can be achieved through the sampling processes or different clustering models. For BC models requiring specifying the number of clusters (k), diversity can also be achieved by specifying a larger k or ***random-k.*** In these cases, k is recommended to be equal to $\sqrt{sample\, size}$ or randomly selected between 2 and $\sqrt{sample\, size}$ (Fred & Jain, 2005; Iam-On et al., 2011). However, the computational cost or complexity may increase for some models with increasing k in large datasets (e.g., the dimensions and sparseness of the binary association matrix). Hence, random sampling and projection methods are often preferred for generating BCs. In such scenarios, it is advisable to use the same k for BCs that is required for the final clustering results (Monti et al., 2003). Improving the quality of BCs can be achieved by selecting or weighting BCs with high quality as well as retaining diversity (Golalipour et al., 2021).

The number of BCs (ensemble size) may also impact clustering performance depending on the features of the data. Generally, increasing ensemble size is associated with higher performance, particularly with diverse BCs (Fern & Brodley, 2003; Wu et al., 2018). Among the limited benchmarking studies, clustering quality normally plateaus for ensemble sizes between 50 and 100 (Fern & Brodley, 2003; Wu et al., 2015, 2018).

In single-run clustering models, a range of internal validity indices, e.g., ***Silhouette index***, ***Calinski–Harabasz (CH) index***, ***Dunn index,*** and ***Gap index,*** have been developed to facilitate the choice of the optimal number of clusters (Gao et al., 2023a, 2023b), which do not generally agree with each other. The advantage of ensemble clustering is that BC stability indicators (e.g., heatmap of co-association matrix) can be used (Dwyer et al., 2020; Monti et al., 2003; Șenbabaoğlu et al., 2014). The optimal number of clusters would ideally demonstrate higher stability, greater overall consensus between pairs of BCs, and better overall fitting performance in the final clusters (see detailed explanation in the implementation section below with examples). In studies with a high explorative purpose, selecting the optimal number of clusters should also take into consideration the meaningfulness of clusters (Gao et al., 2023a, 2023b).

### Implementation of ensemble clustering models

Traditional software packages, such as IBM® SPSS® Statistics and Stata, only allow for standard applications of clustering models, i.e., clustering models based on a single run of the algorithm. Although ensemble clustering models can be implemented via additional programming, they are substantially difficult due to the challenges in programming environments (e.g., operating multiple data objects simultaneously). As open-source statistical programming languages such as R and Python are becoming increasingly popular, the implementation of ensemble clustering models is now much more accessible.

To date, there have been a range of R packages developed for ensemble clustering models, such as diceR, ConsensusClusterPlus, and clue (see Table 1, with the popularity of these packages approximated by frequency of downloads between 2015 and 2025 estimated in Fig. S2). Many of the existing packages were inspired by and followed the design principle of the ensemble clustering model proposed by Monti et al. (2003). Here we have also included brief information regarding other related packages that were developed specifically for a certain type of data or multi-view clustering.

**Table 1:** Ensemble clustering implementation in R

Main R packages	References
**diceR** can perform all the steps on the original dataset, from generating base clusters to consensus formation. It includes a large number of methods for base clustering as well as methods for visualisation of results that inform hypothesis testing ***Main functions****:* dice(), consensus_cluster(), consensus_matrix(), consensus_combine(), consensus_evaluate() ***Methods for sampling***: Random sampling of observations ***Methods for base clusters***: Nonnegative matrix factorisation, hierarchical clustering, divisive analysis clustering, *k*-means, partition around medoids, affinity propagation, spectral clustering, Gaussian mixture model, co-clustering using multiple latent block model, self-organising map with hierarchical clustering, fuzzy c-means clustering, and hierarchical DBSCAN, as well as custom-defined clustering algorithms ***Ensemble methods:*** Majority voting, *k*-modes (categorical variable clustering of cluster label), cluster-based similarity partitioning algorithm (CSPA, graph partitioning of co-association matrix), link-based cluster ensembles (LCE, spectral graph partitioning of co-association matrix), and LCA (categorical variable clustering of cluster label). In the diceR package, CSPA and LCE applied hierarchical clustering instead of the original graph models proposed by Strehl and Ghosh (2002) and Iam-On and Garrett (2010)	(Chiu & Talhouk, 2018; Chiu et al., 2024)
**ConsensusClusterPlus** is a simple package based on two functions only, one generating the base clusters and implementing consensus clustering, and the other calculating cluster-consensus and item-consensus. Several graphical features are incorporated in these two functions ***Main functions***: calcICL(), ConsensusClusterPlus() ***Methods for sampling***: Random sampling of observations and/or variables ***Methods for base clusters***: Hierarchical clustering, partitioning around medoids, and *k*-means, as well as custom-defined clustering algorithms ***Ensemble methods***: Hierarchical consensus clustering based on the method developed by Monti et al. (2003), i.e., hierarchical clustering based on co-association matrix	(Wilkerson & Hayes, 2010; Wilkerson & Waltman, 2020)
**clue** forms consensus on a collection of partitions (soft or hard) or hierarchies (*n*-valued trees) by minimisation of dissimilarity between consensus candidates and base clusters (“optimisation approach”) ***Main functions***: cl_consensus(), cl_dissimilarity(), cl_ensemble() ***Methods for sampling***: Random sampling of observations and variation of parameters/algorithms ***Methods for base clusters***: Partitions or hierarchies must be created prior to using the package with the desired algorithm. Both soft/fuzzy and hard/crisp partitions are possible ***Ensemble methods***: Minimisation is done differently for partitions and hierarchies, with a range of possible methods. For partitions: fixed-point algorithm for soft/hard least squares Euclidian/median Manhattan consensus partitions, fixed-point algorithm for the “first model” from Gordon and Vichi (2001), greedy algorithm of Dimitriadou et al. (2002), sequential unconstrained minimisation techniques. For hierarchies: minimisation of Euclidean dissimilarity, sequential unconstrained minimisation technique with Manhattan dissimilarity, majority consensus tree of Margush and McMorris (1981). Custom-defined algorithms can be used for partitions and hierarchies	(Hornik, 2005; Hornik & Böhm, 2023)
**clusterCons** package performs all the steps on the original dataset, from generating base clusters to consensus formation. Results from different consensus clustering can be merged, considering a user-specified weighting. Visual comparisons can be performed between the number of clusters ***Main functions***: cluscomp(), clrob(), memrob() ***Methods for sampling***: Random sampling of observations and variation of parameters/algorithms ***Methods for base clusters***: Hierarchical clustering, divisive analysis, partitioning around medoids, and *k*-means, as well as custom-defined clustering algorithms ***Ensemble methods***: Hierarchical consensus clustering based on the method developed by Monti et al. (2003)	(Simpson, 2022; Simpson et al., 2010)
**M3C** package uses Monte Carlo simulation to simulate stability scores distribution, in order to inform the selection of optimal number of clusters (via hypothesis testing) ***Main functions***: M3C() ***Methods for sampling***: Random sampling of observations ***Methods for base clusters***: Partitioning around medoids, *k*-means, spectral clustering, hierarchical clustering **Ensemble methods**: Monte Carlo reference-based consensus clustering based on Monti et al. (2003), regularised consensus clustering (addition of penalty term to the estimation of the optimal K). This model was developed to address the issue of existing methods (e.g., PAC) in identifying optimal clusters, which allows comparisons with null distributions using hypothesis testing	(John & Watson, 2023; John et al., 2020)
**cola** package internally using *clue* for assigning consensus labels. Incorporates top-values methods to select variables with highest variability as part of the pre-processing stage. It can also perform hypothesis testing to identify specificity of variables in one or several clusters (e.g., using permutation) ***Main functions***: consensus_partition(), run_all_consensus_partition_methods() ***Methods for sampling***: Random sampling of observations or variables and variation of algorithms ***Methods for base clusters***: Hierarchical clustering, *k*-means, spherical *k*-means, partitioning around medoids, and model-based clustering, as well as custom-defined clustering algorithms ***Ensemble methods***: Internal use of cl_consensus() from the clue package	(Gu, 2023; Gu et al., 2021)
**Other related packages:**	
***coca***: an extension of ConsensusClusterPlus, which focuses on multi-view clustering, using a multi-cluster-of-clusters analysis (COCA). The individual results from individual views were combined using an unweighted approach	(Cabassi & Kirk, 2020a, 2020b)
***klic*:** multi-view clustering using the kernel learning integrative clustering (KLIC) method, which is similar to coca*.* klic uses the kernel *k*-means approach, which allows one to assign weights to different views, aimed at removing view-specific noise	(Cabassi & Kirk, 2020b; Cabassi et al., 2020)
***SC3***: specific consensus package for clustering single-cell RNA-Seq data. The consensus clustering approach is similar to the method proposed by Monti et al. (2003) except that BCs were generated from data with different methods of distance calculation and transformation	(Kiselev, 2023; Kiselev et al., 2017)
***clusterExperiment***: specific consensus package for clustering single-cell and other large gene expression data	(Purdom & Risso, 2023; Risso et al., 2018)
***BCClong:*** Bayesian consensus clustering for multi-view longitudinal data using a joint modelling approach	(Tan et al., 2022, 2023)

Not all packages are in CRAN, and some packages will not be maintained regularly. Please check detailed package documentation when using these packages

## Example of algorithm implementation

### Dataset

**Fig. 6:**
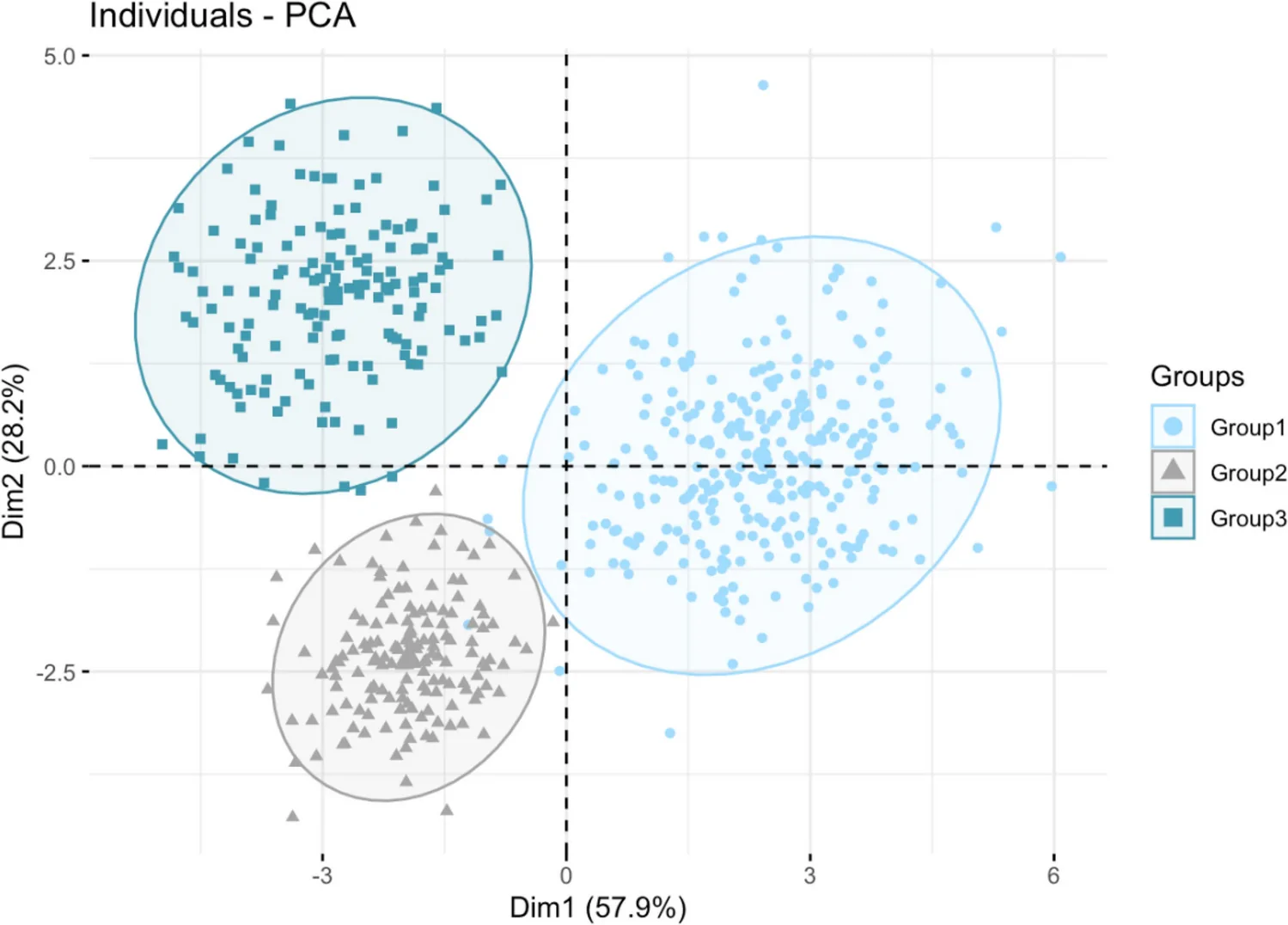
PCA of the three generated clusters in the simulated example dataset

For simplicity in demonstrating different algorithms, we have used a simulated dataset generated from multivariate normal distributions. This simplified example does not capture the complexity of real-world data structures and challenges (e.g., sparsity, skewed distributions, missing data, or high dimensionality). However, this relatively simple generative process enables readers to become familiar with the analytical procedures and underlying methodological concepts. The code for generating the data is provided in Supplementary Material II. Briefly, we applied three steps: (1) generated a mixture of three multivariate normal distributions (with different means and variances) on a three-dimensional space representing three clusters with *n* = 300, 150, and 150; (2) randomly projected the original three-dimensional data onto a 10-dimensional space, creating a dataset with 600 observations and 12 variables; and (3) added random errors to individual variables and additional outliers. The distributions of the three clusters are visualised using principal component analysis (PCA, see Fig. 6).

### Steps of ensemble clustering

In this section, the detailed procedures for implementing ensemble clustering models are presented. The R code for the step-by-step guide of modelling processes can be found in Supplementary Material III.

#### Developing the analysis plan

The development of an analysis plan before obtaining or analysing the data can be difficult for clustering analysis, as the choice of the appropriate model may depend on the characteristics of the data and the performance of different algorithms. However, it remains important to develop the framework for the analytical procedures and guidance for practical decisions. Ideally, this process should be pre-registered to support robust open science practices (Gao et al., 2023a, 2023b). A template analysis plan with the current tutorial analysis as an example is provided in Table S2 in Supplementary Material I.

#### Data assessment and pre-processing

Researchers should undertake in-depth examination of source data for characteristics such as dimensionality, distribution of variables, and level of missing data, which can be done using functions such as dfSummary from the summarytools package. This process should be followed by data pre-processing procedures (e.g., identification of outliers, normalisation, and dimensionality reduction, see Supplementary Material III). In this example, we employ ***principal component analysis (PCA)*** for dimensionality reduction. Many other dimensionality reduction methods can be used such as ***multidimensional scaling (MDS***), ***t-distributed stochastic neighbour embedding (t-SNE), nonnegative matrix factorisation (NMF)***, and ***uniform manifold approximation and projection (UMAP)***; see detailed comparisons of these models elsewhere (Ayesha et al., 2020; Jia et al., 2022; Van Der Maaten et al., 2009). NMF and related methods have been widely applied in the ensemble clustering framework, notably in the fields of genetics and neuroscience, to effectively manage high-dimensional and sparse data (Brunet et al., 2004; Gaujoux & Seoighe, 2010).

**Figure:**
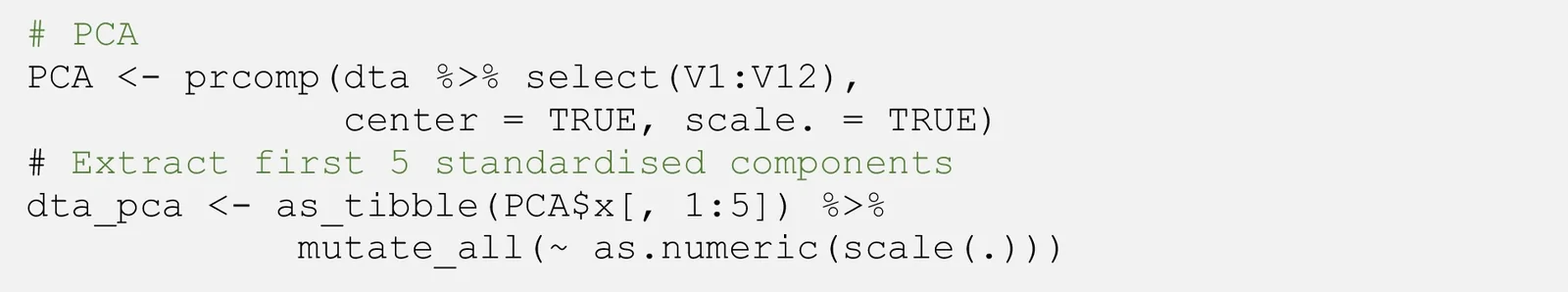

#### Ensemble clustering

Many existing packages provide individual functions for each step of e clustering (generating BCs and ensemble BCs). As methods involving random sampling of observations will introduce missing data, one additional imputation step is also required. The quality and diversity of BCs can also be examined. Poor-quality BCs (lower ranking across a range of internal validity indices) can be trimmed or removed. The detailed code for carrying out the two ensemble clustering steps is provided in Supplementary Material II using the diceR package. However, most users may choose to use the integrated approach with one function incorporating both steps. Using the dice function from the diceR package, this can be achieved using the following code; additional specifications (e.g., trim, reweight, and plot) can be found in the user manual (https://cran.r-project.org/web/packages/diceR/refman/diceR.html#dice).

**Figure:**
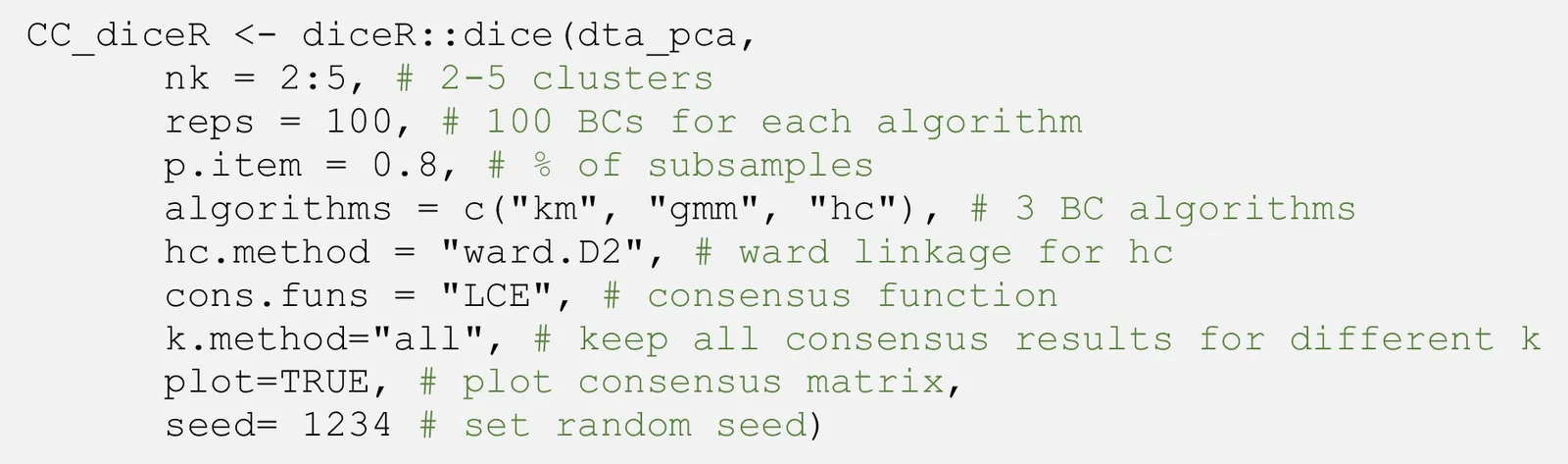

In this analysis, we created four solutions with cluster numbers varying from 2 to 5, utilising three BC algorithms, *k*-means ("km"), Gaussian mixture model (GMM, "gmm"), and hierarchical clustering ("hc") with Ward’s linkage method (hc.method = "ward.D2"), along with one consensus function, LCE (cons.funs = "LCE"). dice offers a few consensus functions, such as *k*-mode and majority voting, among which CSPA is consistent with the model specification proposed by Monti et al. (2003). The function is also set up to automatically calculate internal validity indices, which facilitate the evaluation of BCs, allowing for the automatic exclusion of those with low scores on internal indices (by setting trim = TRUE), and the reweighting of BCs based on their internal validity (by setting reweigh = TRUE). Specifying plot = TRUE will allow us to generate associated plots (e.g., consensus matrix, see Fig. 6, with interpretation provided in subsequent sections).

Similar code can also be achieved using the ConsensusClusterPlus function from the ConsensusClusterPlus package. In this package, we can perform both random sampling of variables and observations, in contrast to diceR, which only allows random sampling of observations.

**Figure:**
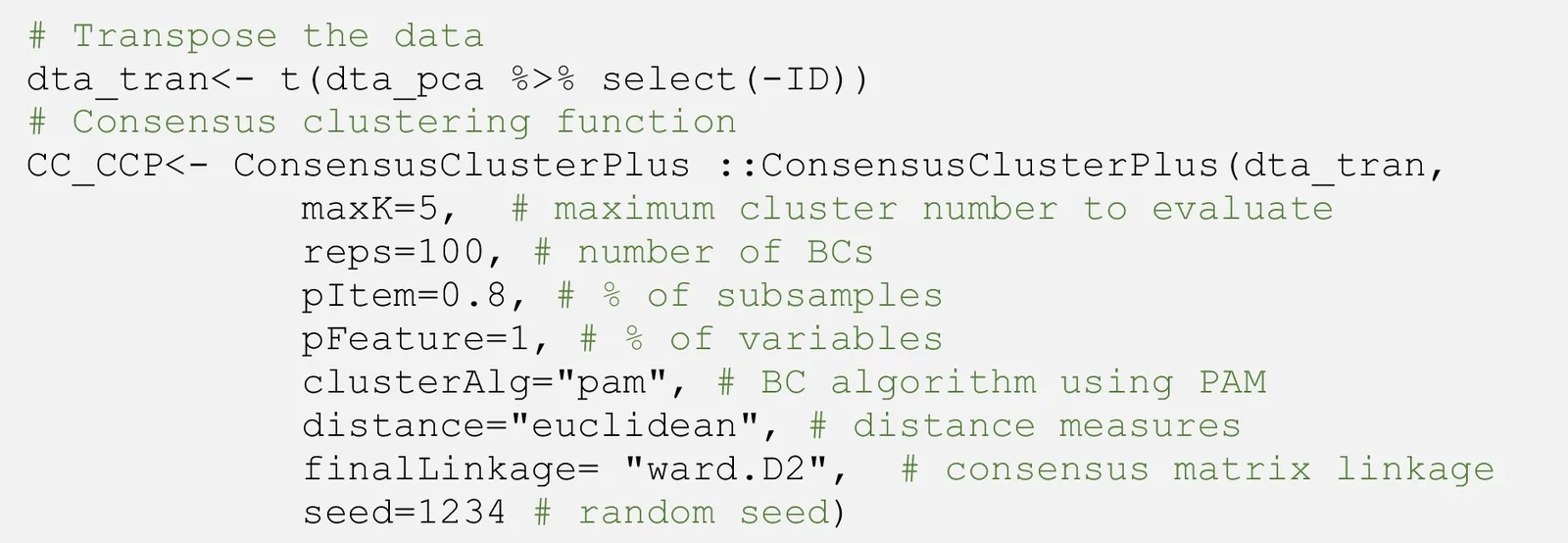

Users can also manually specify the two steps with the support of existing packages (e.g., to incorporate imputed datasets currently not directly embedded in packages such as diceR); see the example provided in Supplementary Material III.

#### Specifying and parameterising ensemble clustering models

Ensemble clustering models offer a variety of specification options and parameter settings, such as the selection of BC algorithms, consensus matrix types, the number of BCs, and the fraction of samples to utilise. Typically, a sampling proportion of 80% is recommended (Monti et al., 2003; Șenbabaoğlu et al., 2014) to capture most data characteristics while allowing for variations among BCs.

In this illustrative example, we use 100 BCs. In practice, the ensemble size can be determined by how diverse BCs are, as highlighted above. Users also have the option to explore how the results change with an increasing number of BCs.

#### Choosing the optimal parameters and accessing validity

**Fig. 7:**
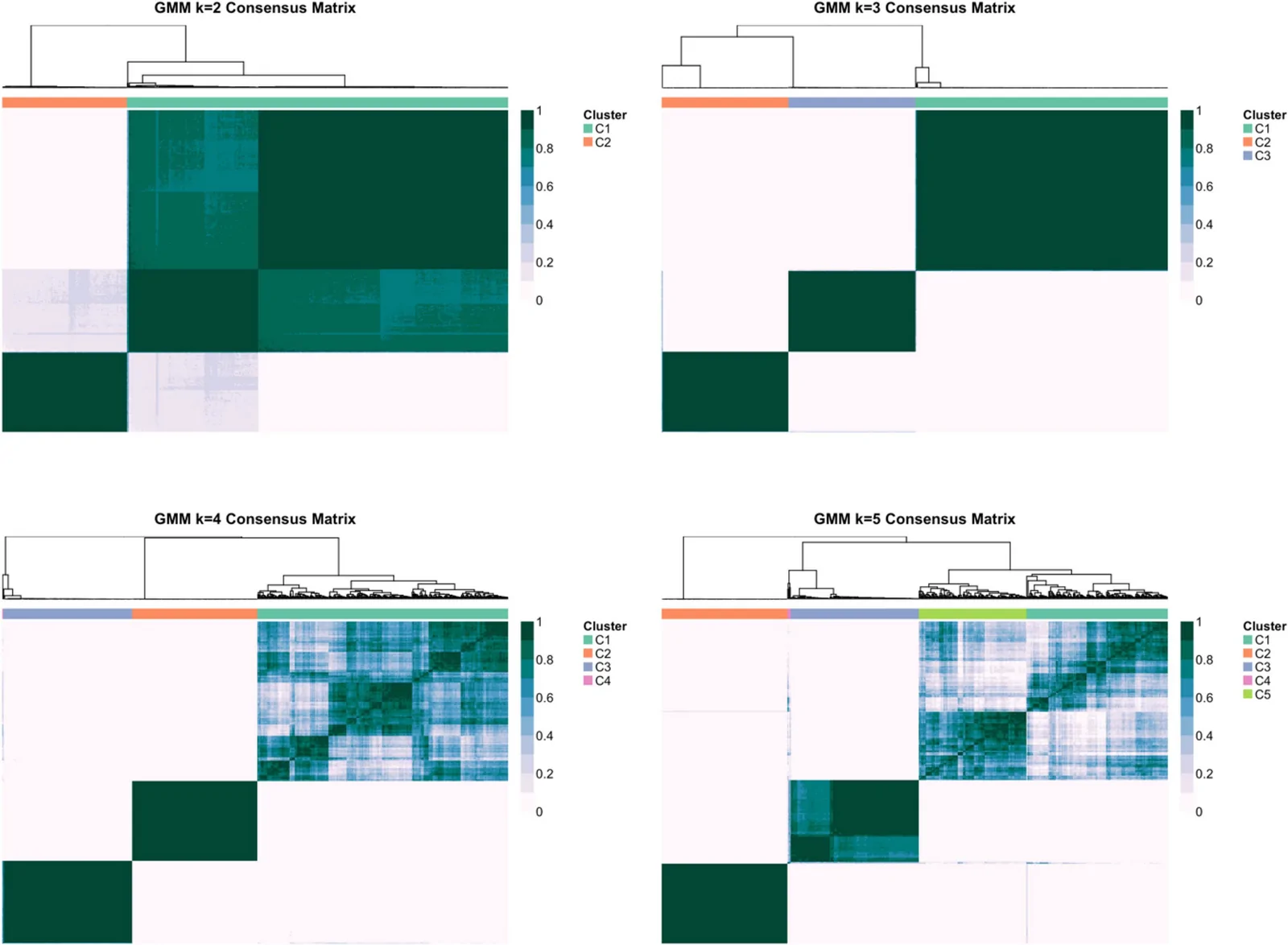
Consensus matrix constructed from 100 BCs estimated with GMM, with the number of clusters (*k*) ranging from 2 to 5, using the ***diceR*** function in the ***diceR*** package with the simulated example dataset

A critical and primary benefit of ensemble clustering is that the level of consensus between BCs can also be used to choose the optimal number of clusters based on stability, as discussed above. As shown in Fig. 6, when the number of clusters (k=3) was chosen based on the ground truth in the simulated data, the consensus matrix showed three clear square-shaped structures (with distinct boundaries) along the antidiagonal direction, suggesting that pairs of observations were commonly clustered into the same group (or different groups). When other numbers of clusters were chosen, there were no clear antidiagonal structures. To quantify this visual dispersion, Brunet et al. (2004) applied a measure based on the ***cophenetic correlation coefficient***, which measures the correlation between the consensus co-association matrix and the distance on the reordered co-association matrix in Fig. 7.

**Fig. 8:**
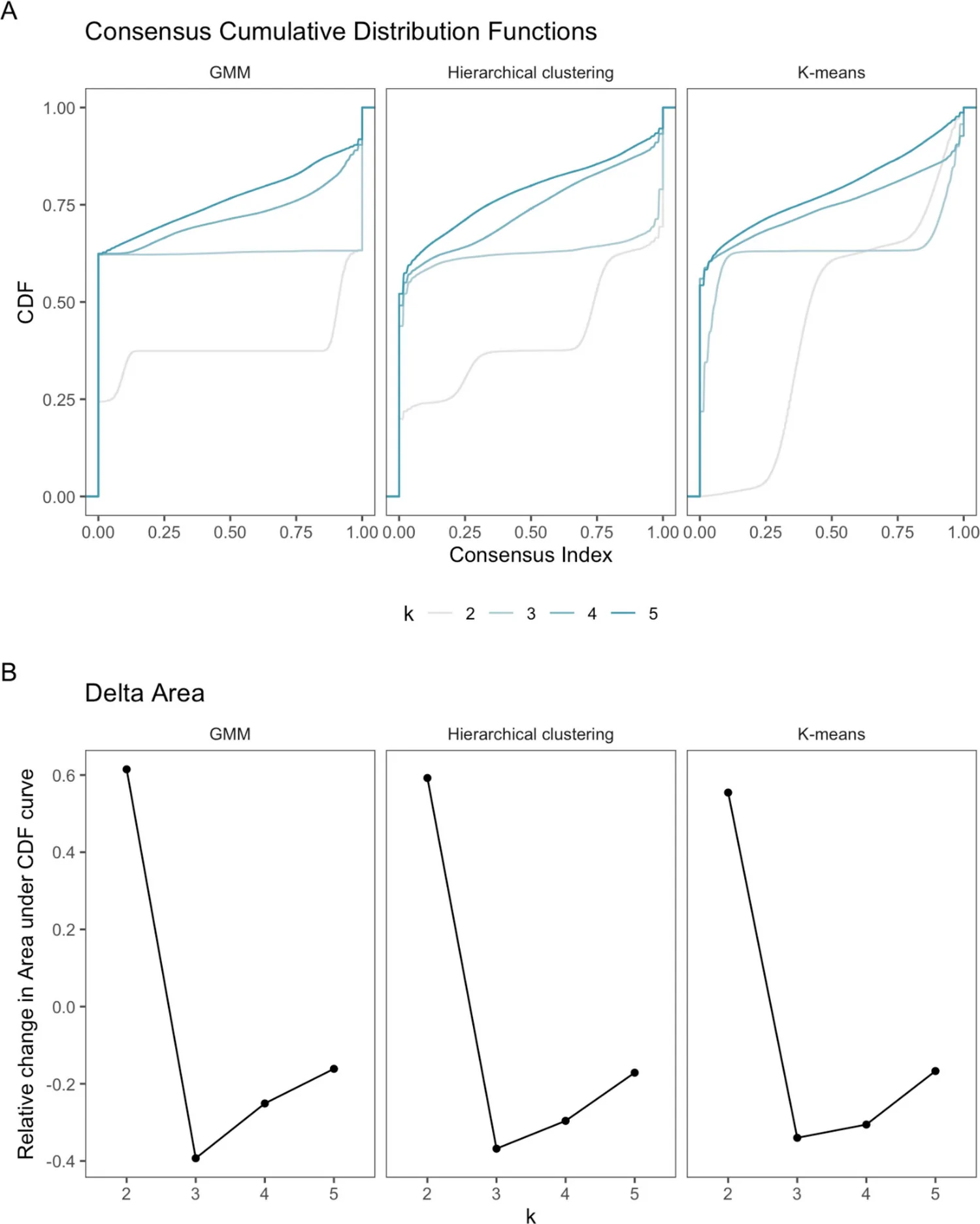
Cumulative distribution function (CDF) of consensus matrix indexes (**A**) and delta area plot representing changes in CDF (**B**) estimated from the diceR function in the diceR package using the simulated example dataset

Monti et al. (2003) further developed a methodology to facilitate choosing k using the distribution of consensus matrix index. An optimal number of k will commonly produce a consensus matrix index with a bimodal distribution, with a high number of indexes closer to 0 (always clustered to different groups) or 1 (always clustered to the same group). This distribution can be described using an empirical cumulative distribution function (CDF) and assessed by considering changes in the area under the CDF curve, known as the delta area (see Fig. 7). As suggested by Monti et al. (2003), the CDF derived from the optimal k typically exhibits three characteristics: a three-phase step function in the CDF plot, beginning with an initial step near 0 (indicative of the proportion of zeros in the matrix), followed by a flat line across the 0–1 range, and concluding with a final step near 1. This is reflected as an “elbow” effect seen in the relative change in the area under the delta area plot; see Fig. 8 (k=3 for all three BC algorithms). The CDF method can sometimes identify clusters in data where none exist (see examples provided by Șenbabaoğlu et al., 2014). To address, this issue, the authors introduced the proportion of ambiguous clustering (PAC) score (Șenbabaoğlu et al., 2014). This metric measures the proportion of sample pairs whose consensus indices fall within an indeterminate range (e.g., 0.05–0.95, which can be tuned) indicating observations that are more ambiguous in cluster membership. A lower PAC score indicates a flatter middle segment in the CDF, reflecting greater consistency across BCs. PAC scores are computed in the dice function, which also serves as the default method for selecting the optimal k (when k.method = “all” is not specified). However, PAC was also suggested to have an inherent bias towards higher values of k because the null reference distributions are not considered (John et al., 2020).

Apart from the number of clusters, an ensemble clustering pipeline also involves many other hyper‑parameters that must be set before the algorithm can be run—for example, the dimensionality retained, the covariance structure chosen on finite mixture models, the linkage criterion used in hierarchical agglomeration, and the neighbourhood radius in density‑based methods. In supervised prediction problems, hyper‑parameters are typically tuned by maximising performance on unseen test set(s) (e.g., by cross‑validation). However, as clustering is unsupervised, no direct signal is available; consequently, hyper‑parameters must be chosen on the basis of indirect evidence such as internal, external, predictive, and translational validity of the resulting groups (see explanation in Table S2). Internal validity can be evaluated using CV or nested CV framework to ensure that the decision-making process is not impacted by random fluctuations in the data. This can be evaluated using multiple stability indicators, including PAC and CDF, as well as other indicators such as the adjusted Rand index (Gao et al., 2023a, 2023b). This approach is detailed in the examples provided by Dwyer et al. (2022). One operational example is provided in Fig. S3 and Supplementary Material IV.

To confirm that the cluster solution did not occur by chance, permutation-based non-parametric tests can be incorporated (Dwyer et al., 2022). In a permutation test, an empirical distribution of a statistic under the null hypothesis is constructed by reshuffling the observed data many times and recalculating the statistic of interest for each reshuffled dataset. The statistic calculated for the ensemble cluster is then compared with this empirical distribution to obtain a *p*-value. An example of such a test can be found in Supplementary Material III, where the PAC score is calculated for 100 permutations of the BC classifications obtained through *k*-means, and compared with the PAC score of the original results. The *p*-value obtained was 0, irrespective of the bounds selected for the PAC score, due to the clear superiority of *k*-means clustering over random assignment of observations to clusters.

Assessing the ***external validity***, that is, the generalisability of the clustering results to external data (data from other settings, populations, or time periods), remains a significant challenge, primarily due to the absence of ground truth. While various methods have been proposed, no consensus on the processes and procedures for such validation has been established. This situation is largely attributed to the perception of clustering analysis as an exploratory technique (Ullmann et al., 2022). However, we believe that external validity is desirable to allow findings to be translated into practice. We acknowledge that not all studies can find a matched external validation dataset. In these cases, researchers can choose to publish a prediction algorithm (algorithm predicting cluster labels), enabling others to validate when such data are available. When the external validation data are available, the validation can either carry out two independent clustering analyses and directly compare the similarity of clustering results or apply a predictive algorithm (Brucar et al., 2023). For example, Ullmann et al. (2022) proposed an approach that compares multiple indexes and performance indicators across models, replicating the clustering findings (rerun of the identical model on validation data) or predicting clustering results (cluster labels in the validation data obtained from prediction models of clustering labels). When the two validation approaches yield comparable clusters (e.g., showing similar relationships with the input data), consistent correlations with external factors, and concordance between the two sets of results, this indicates a high level of external validity for the findings.

External variables not included in the clustering process (e.g., sociodemographic factors and other associated outcomes such as diagnoses and long-term trajectories) can be used to assess the predictive and translational validity (Brucar et al., 2023).

#### Reporting ensemble clustering studies

Studies employing ensemble clustering should follow the reporting guidelines of clustering studies (Gao et al., 2023a, 2023b). In addition, users should include detailed modelling procedures (e.g., BC algorithms, consensus function, the method for choosing optimal k) and choice of parameters (e.g., sampling proportion, number of BCs) as well as associated results (e.g., consensus matrix, CDF and Delta area plot, PAC scores).

## Important practical notes

Importantly, limitations of BC algorithms will be automatically carried forward in the consensus process (e.g., assumptions in the shapes of clusters, sensitivity to scale, outliers, and density differences between clusters, impacts of hyperparameter choices); see details discussed elsewhere (Gao et al., 2023a, 2023b). Clusters with arbitrary or non-convex shapes may not be recovered by BC algorithms that are designed to identify primarily convex cluster structures. In this case, the clustering solution may appear to be stable but fail to recover the true underlying heterogeneity. Therefore, it is important to evaluate whether the assumptions of BC algorithms can be met and test the impact of choosing different BC and ensemble algorithms. Users can take advantage of the diceR package, which allows for simultaneously employing and comparing multiple algorithms (see details in Supplementary Material II).

Stability and internal validity do not guarantee external validity; similarly, stability and internal and external validity do not translate directly to meaningfulness. Clustering solutions that are stable and describe the data well can fail to demonstrate external validity. Such failures may arise from many different factors, such as differences in the level of noise, underrepresented subgroups, variations in data collection and processing pipelines, and overfitting of the original clustering solutions (Wen et al., 2025). Conversely, stable, valid, and generalisable clustering solutions may nevertheless have very little practical utility. For example, the partition of “low”, “medium”, and “high” categories may provide little actionable insight. Therefore, the choice of modelling approaches and perimeters will need to be guided by the aim of the research and the potential implications of the findings, rather than purely relying on a data-driven approach.

## Comparison

To demonstrate the need for ensemble clustering, we conducted simulations using datasets from three published studies (Amiet et al., 2020; Cotton et al., 2022; Jakobson & Rigby, 2021) (see details of these studies in Table S3 in Supplementary Material I). The primary aim of these simulations was to examine the extent to which ensemble clustering improves the stability and robustness of clustering solutions relative to single-run models when data are subject to small perturbations. Meaningful clustering solutions should ideally remain stable under minor changes in the data, with similar observations consistently grouped into the same clusters. We therefore evaluated clustering stability by introducing varying levels of perturbation to the data. For each simulation dataset, we generated perturbed datasets by randomly removing 0.25–20% of the data and filled the omitted data with ***multivariate imputation by chained equations (MICE)***. This process ensures that the replacement values follow the same multivariate distribution as the original data. For each perturbation condition (e.g., 0.25% missing), imputation was repeated 100 times to generate multiple perturbed datasets. We then applied each clustering algorithm repeatedly to the perturbed datasets within a perturbation condition (e.g., 0.25% missing), and the stability of a clustering algorithm was characterised by the Rand index (RI, which measures levels of agreement between pairs of clustering results). The larger RI values suggest that the algorithm can produce more stable and generalisable results when there are minor changes in the data. Detailed procedures and results are provided in Supplementary Material V.

**Fig. 9:**
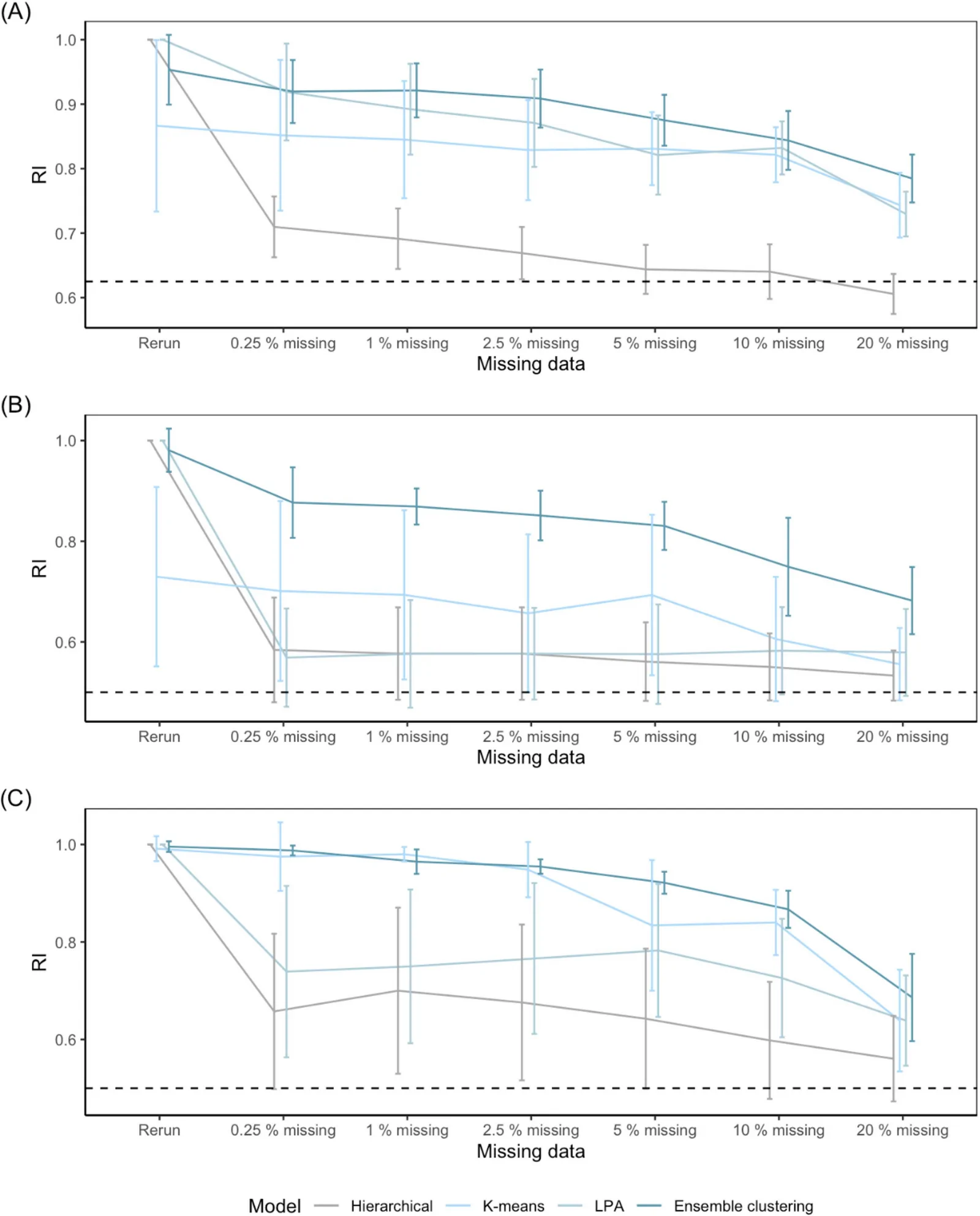
Changes in average clustering stability (measured using the pairwise Rand index, RI) across perturbed datasets (100 replications for each perturbation condition) using four types of clustering models (error bar: standard deviation). *Note:* The source datasets were obtained from **A** clustering of 20 AQoL-6D items (Cotton et al., 2022) with four clusters identified; **B** clustering of alexithymic traits (Jakobson & Rigby, 2021) with two clusters identified; and **C** clustering of cannabis expectancies (Amiet et al., 2020). In this demonstration, RI is chosen as the stability index due to its intuitive interpretation. However, RI is sensitive to the specified number of clusters (k). Therefore, we use a dashed line to represent the RI expected under random cluster assignment while preserving the number of clusters identified in the original study, providing a theoretical lower-bound (chance-level) benchmark for clustering agreement

Results from three studies are provided in Fig. 9. Minor changes in data (e.g., 0.25% of the data being different) introduced substantially different results. In some cases (e.g., hierarchical clustering), stability was only slightly better than random sampling (see Fig. 9A). Ensemble clustering, on the other hand, had the best performance with the slowest decay in stability with increasing perturbation. This is particularly the case in the example in Fig. 9B, where the source data have slightly higher dimensionality. Single-run models also showed substantially wider error bars for the pairwise RI across three simulation studies, indicating greater variability in agreement across perturbed dataset pairs. This again highlights the lack of stability of single-run clustering solutions and their greater sensitivity to data perturbations than ensemble clustering. This simple example, although demonstrating relative robustness and reproducibility of ensemble clustering solutions under data perturbation, does not imply that its solutions accurately reflect the underlying heterogeneity.

## Discussion

Clustering techniques designed in the twentieth century have contributed important insights in psychology and psychiatry; however, challenges related to reproducibility, stability, and clinical translation have limited their real-world impact. In this paper, we aimed to address this problem by translating ensemble clustering techniques to audiences in the field. As outlined by our tutorial, ensemble clustering is a flexible technique that can be relatively easy to implement with existing toolboxes to address related questions. We then demonstrated that minor perturbations of psychological/psychiatry data can lead to major differences in clustering solutions using traditional single-run methods, which are reduced with ensemble clustering. When combined, future research in the field will benefit from these techniques, and we advocate their widespread use.

In addition to a simulated demonstration example that serves as a step-by-step guide for the ensemble clustering technique, we provided a comparative analysis with different clustering methods and data missing rates to evaluate the performance of ensemble clustering in three complex real-world scenarios in psychology and psychiatry. The results demonstrated the significant reliability and application value of ensemble clustering technology in these fields. The resources presented will enable researchers to apply advanced ensemble clustering techniques to decompose heterogeneous and complex psychological data into stable subgroups.

Ensemble clustering, along with its extensions such as multi-view clustering (see one of the examples provided by Pfeifer et al., 2023), offers a robust, data-driven framework that enables the integration of high-dimensional, multimodal data and diverse clustering paradigms (Dwyer et al., 2022; Gao et al., 2023a, 2023b). These approaches support insights such as the understanding of the heterogeneous biological, psychological, and social mechanisms of mental health conditions and precision medicine approaches to treatment (Dwyer et al., 2018; Manchia et al., 2020; Wright & Woods, 2020). Additionally, ensemble clustering can increase confidence in clustering results by improving the stability and robustness of identified subgroups, thereby supporting more reliable interpretation and translation of the knowledge into real-world practice.

Psychological and psychiatric data present several methodological challenges that warrant consideration when applying ensemble clustering approaches. These data frequently comprise ordinal (e.g., Likert-scale) variables, latent constructs, and highly correlated variables. Treating highly corelated ordinal items, underpinned by latent constructs, as continuous variables and directly integrating them into distance-based clustering models will be problematic. These features can distort the distance matrix, producing stable clustering solutions that are a poor fit for the data (e.g., producing cluster results dominated by one overly represented domain among source variables). In these cases, the application of proper BC models (e.g., model-based clustering) or dimensionality reduction methods is necessary. Longitudinal data, with trajectory changes and dynamic relationships between variables, can introduce further complexity. The typical ensemble approach cannot incorporate the full features of advanced longitudinal models, such as growth mixture models, which model latent subgroups of longitudinal trajectory random effects within subgroup. In these cases, assembly is difficult, as disagreement between models may arise from many different parameters (e.g., trajectory shapes, variance structures), making it unclear which model components should be meaningfully combined into a consensus solution. In such cases, different ensemble pipelines are required, e.g., via Bayesian models (Xiao et al., 2025).

It is important to note that ensemble clustering techniques also come with several limitations. First, they demand considerable computational resources, especially when processing large datasets. This can be a major barrier for applying ensemble clustering models, which can be addressed by applying computationally efficient models (e.g., using a binary cluster association matrix) and implementing computation pipelines in high-performance computing clusters. Second, the flexibility in choosing different clustering methods, optimisations, and strategies may introduce additional complexities. These choices are often important because the limitations of the underlying BC algorithms can also be carried forward in the consensus approach. Despite the substantial advantages offered by ensemble clustering models, their deployment also necessitates validation in external samples. Further research and development will undoubtedly address some of these limitations, but these methods have already demonstrated their potential in other fields. Developing familiarity with the conceptual framework as well as practical skills for applying these models would be a great advantage for research in psychology and psychiatry.

## Supplementary Information

Below is the link to the electronic supplementary material.

ESM 1 Supplementary Material 1 (DOCX 352 KB)

ESM 2 Supplementary Material 2 (PDF 295 KB)

ESM 3 Supplementary Material 3 (PDF 2.80 MB)

ESM 4 Supplementary Material 4 (PDF 1.33 MB)

ESM 5 Supplementary Material 5 (PDF 1.04 MB)

## Data Availability

All data and analysis code are available at https://doi.org/10.17605/OSF.IO/THJDC. The pre-print version of the paper is available at https://doi.org/10.31234/osf.io/fq6e9.

## Footnotes

1 PubMed search using (“cluster analysis” OR “clustering analysis” OR “k-means” OR “hierarchical clustering” OR LCA OR “latent class analysis” OR “latent profile analysis” OR LPA OR “latent class trajectory analysis” OR “growth mixture modeling” OR “growth mixture modelling” OR “HDBSCAN” OR “density-based clustering” OR “fuzzy clustering” OR “graph clustering” OR “spectral clustering” OR “subspace clustering” or “multi-view clustering”) AND (depression OR anxiety OR personality OR psychosis OR bipolar OR anorexia OR MRI OR EEG OR “mental health” OR psychology OR psychiatry) on 21 November 2024.

2 Ensemble clustering and consensus clustering are often used interchangeably as umbrella terms. However, consensus clustering is also used frequently to describe a specific type of ensemble clustering, the model proposed by Monti et al. (2003). In this paper, we use ensemble clustering for all clustering ensemble methods and consensus clustering for specific models.

3 *Note:* Not all multi-view clustering algorithms require ensemble processes.

## Code availability

All analysis code is available at https://doi.org/10.17605/OSF.IO/THJDC.

## References

[CR1] Abbasi, S.-o, Nejatian, S., Parvin, H., Rezaie, V., & Bagherifard, K. (2019). Clustering ensemble selection considering quality and diversity. *Artificial Intelligence Review,**52*(2), 1311–1340. 10.1007/s10462-018-9642-2

[CR2] Alqurashi, T., & Wang, W. (2019). Clustering ensemble method. *International Journal of Machine Learning and Cybernetics,**10*(6), 1227–1246. 10.1007/s13042-017-0756-7

[CR3] Amiet, D., Youssef, G. J., Hagg, L. J., Lorenzetti, V., Parkes, L., Solowij, N., & Yücel, M. (2020). Young adults with higher motives and expectancies of regular cannabis use show poorer psychosocial functioning. *Frontiers in Psychiatry,**11*, Article 599365. 10.3389/fpsyt.2020.599365 PMC7771276 33384630

[CR4] Ayesha, S., Hanif, M. K., & Talib, R. (2020). Overview and comparative study of dimensionality reduction techniques for high dimensional data. *Information Fusion,**59*, 44–58. 10.1016/j.inffus.2020.01.005

[CR5] Beijers, L., Wardenaar, K. J., van Loo, H. M., & Schoevers, R. A. (2019). Data-driven biological subtypes of depression: Systematic review of biological approaches to depression subtyping. *Molecular Psychiatry,**24*(6), 888–900. 10.1038/s41380-019-0385-5 30824865

[CR6] Berikov, V. (2014). Weighted ensemble of algorithms for complex data clustering. *Pattern Recognition Letters,**38*, 99–106. 10.1016/j.patrec.2013.11.012

[CR7] Bondar, J., Caye, A., Chekroud, A. M., & Kieling, C. (2020). Symptom clusters in adolescent depression and differential response to treatment: A secondary analysis of the Treatment for Adolescents with Depression Study randomised trial. *The Lancet Psychiatry,**7*(4), 337–343. 10.1016/S2215-0366(20)30060-2 32199509

[CR8] Boongoen, T., & Iam-On, N. (2018). Cluster ensembles: A survey of approaches with recent extensions and applications. *Computer Science Review,**28*, 1–25. 10.1016/j.cosrev.2018.01.003

[CR9] Bradley, P. S., & Fayyad, U. M. (1998). Refining initial points for k-means clustering. ICML,

[CR10] Brière, G., Darbo, É., Thébault, P., & Uricaru, R. (2021). Consensus clustering applied to multi-omics disease subtyping. *BMC Bioinformatics,**22*(1), 361. 10.1186/s12859-021-04279-1 PMC8259015 34229612

[CR11] Brucar, L. R., Feczko, E., Fair, D. A., & Zilverstand, A. (2023). Current approaches in computational psychiatry for the data-driven identification of brain-based subtypes. *Biological Psychiatry,**93*(8), 704–716. 10.1016/j.biopsych.2022.12.020 PMC10038896 36841702

[CR12] Brunet, J.-P., Tamayo, P., Golub, T. R., & Mesirov, J. P. (2004). Metagenes and molecular pattern discovery using matrix factorization. *Proceedings of the National Academy of Sciences of the United States of America,**101*(12), 4164–4169. 10.1073/pnas.0308531101 PMC384712 15016911

[CR13] Cabassi, A., & Kirk, P. D. W. (2020a). *coca: Cluster-of-Clusters Analysis*. In (Version 1.1.0) R package. https://CRAN.R-project.org/package=coca

[CR14] Cabassi, A., & Kirk, P. D. W. (2020b). Multiple kernel learning for integrative consensus clustering of omic datasets. *Bioinformatics,**36*(18), 4789–4796. 10.1093/bioinformatics/btaa593 PMC7750932 32592464

[CR15] Cabassi, A., Kirk, P. D. W., & Gonen, M. (2020). *klic: Kernel Learning Integrative Clustering*. In (Version 1.0.4) R package. https://CRAN.R-project.org/package=klic

[CR16] Cai, J., Wang, S., & Guo, W. (2021). Unsupervised embedded feature learning for deep clustering with stacked sparse auto-encoder. *Expert Systems with Applications,**186*, Article 115729. 10.1016/j.eswa.2021.115729

[CR17] Case, M., Stauffer, V. L., Ascher-Svanum, H., Conley, R., Kapur, S., Kane, J. M., Kollack-Walker, S., Jacob, J., & Kinon, B. J. (2011). The heterogeneity of antipsychotic response in the treatment of schizophrenia. *Psychological Medicine,**41*(6), 1291–1300. 10.1017/S0033291710001893 PMC3080711 20925971

[CR18] Cattell, R. B. (1943). The description of personality: Basic traits resolved into clusters. *Journal of Abnormal and Social Psychology,**38*(4), 476–506. 10.1037/h0054116

[CR19] Chao, G., Sun, S., & Bi, J. (2021). A survey on multiview clustering. *IEEE Transactions on Artificial Intelligence,**2*(2), 146–168. 10.1109/TAI.2021.3065894 PMC8925043 35308425

[CR20] Chiu, D. S., & Talhouk, A. (2018). DiceR: An R package for class discovery using an ensemble driven approach. *BMC Bioinformatics,**19*(1), 11. 10.1186/s12859-017-1996-y PMC5769335 29334888

[CR21] Chiu, D. S., Talhouk, A., & Liu, J. (2024). *diceR: Diverse Cluster Ensemble in R*. In (Version 2.2.0) R package. https://CRAN.R-project.org/package=diceR

[CR22] Clatworthy, J., Buick, D., Hankins, M., Weinman, J., & Horne, R. (2005). The use and reporting of cluster analysis in health psychology: A review. *British Journal of Health Psychology,**10*(3), 329–358. 10.1348/135910705X25697 16238852

[CR23] Cotton, S. M., Hamilton, M. P., Filia, K., Menssink, J. M., Engel, L., Mihalopoulos, C., Rickwood, D., Hetrick, S. E., Parker, A. G., Herrman, H., Telford, N., Hickie, I., McGorry, P. D., & Gao, C. X. (2022). Heterogeneity of quality of life in young people attending primary mental health services. *Epidemiology and Psychiatric Sciences,**31*, Article e55. 10.1017/s2045796022000427 PMC9305730 35856272

[CR24] Dimitriadou, E., Weingessel, A., & Hornik, K. (2002). A combination scheme for fuzzy clustering. *International Journal of Pattern Recognition and Artificial Intelligence*, *16*(7), 901–912. 0.1142/S0218001402002052

[CR25] Dwyer, D. B., Buciuman, M.-O., Ruef, A., Kambeitz, J., Sen Dong, M., Stinson, C., Kambeitz-Ilankovic, L., Degenhardt, F., Sanfelici, R., Antonucci, L. A., Lalousis, P. A., Wenzel, J., Urquijo-Castro, M. F., Popovic, D., Oeztuerk, O. F., Haas, S. S., Weiske, J., Hauke, D., Neufang, S., … Consortium, P. (2022). Clinical, brain, and multilevel clustering in early psychosis and affective stages. *JAMA Psychiatry,**79*(7), 677–689. 10.1001/jamapsychiatry.2022.1163 PMC9118078 35583903

[CR26] Dwyer, D. B., Falkai, P., & Koutsouleris, N. (2018). Machine learning approaches for clinical psychology and psychiatry. *Annual Review of Clinical Psychology,**14*, 91–118. 10.1146/annurev-clinpsy-032816-045037 29401044

[CR27] Dwyer, D. B., Kalman, J. L., Budde, M., Kambeitz, J., Ruef, A., Antonucci, L. A., Kambeitz-Ilankovic, L., Hasan, A., Kondofersky, I., Anderson-Schmidt, H., Gade, K., Reich-Erkelenz, D., Adorjan, K., Senner, F., Schaupp, S., Andlauer, T. F. M., Comes, A. L., Schulte, E. C., Klöhn-Saghatolislam, F., … Koutsouleris, N. (2020). An investigation of psychosis subgroups with prognostic validation and exploration of genetic underpinnings: The PsyCourse study. *JAMA Psychiatry,**77*(5), 523–533. 10.1001/jamapsychiatry.2019.4910 PMC7042925 32049274

[CR28] Feczko, E., & Fair, D. A. (2020). Methods and challenges for assessing heterogeneity. *Biological Psychiatry,**88*(1), 9–17. 10.1016/j.biopsych.2020.02.015 PMC8404882 32386742

[CR29] Fern, X. Z., & Brodley, C. E. (2003). Random projection for high dimensional data clustering: A cluster ensemble approach. Proceedings of the 20th international conference on machine learning (ICML-03),

[CR30] Fern, X. Z., & Lin, W. (2008). Cluster ensemble selection. *Statistical Analysis and Data Mining: The ASA Data Science Journal,**1*(3), 128–141. 10.1002/sam.10008

[CR31] Forbes, M. K., Baillie, A., Batterham, P. J., Calear, A., Kotov, R., Krueger, R. F., Markon, K. E., Mewton, L., Pellicano, E., Roberts, M., Rodriguez-Seijas, C., Sunderland, M., Watson, D., Watts, A. L., Wright, A. G. C., & Clark, L. A. (2024). Reconstructing psychopathology: A data-driven reorganization of the symptoms in the Diagnostic and Statistical Manual of Mental Disorders. *Clinical Psychological Science*. 10.1177/21677026241268345 PMC12711323 41415680

[CR32] Forbes, M. K., Sunderland, M., Rapee, R. M., Batterham, P. J., Calear, A. L., Carragher, N., Ruggero, C., Zimmerman, M., Baillie, A. J., Lynch, S. J., Mewton, L., Slade, T., & Krueger, R. F. (2021). A detailed hierarchical model of psychopathology: From individual symptoms up to the general factor of psychopathology. *Clinical Psychological Science,**9*(2), 139–168. 10.1177/2167702620954799 PMC7983870 33758691

[CR33] Franklyn, S. I., Stewart, J., Beaurepaire, C., Thaw, E., & McQuaid, R. J. (2022). Developing symptom clusters: Linking inflammatory biomarkers to depressive symptom profiles. *Translational Psychiatry,**12*(1), Article 133. 10.1038/s41398-022-01900-6 PMC8971490 35361785

[CR34] Fred, A. L. N., & Jain, A. K. (2002). Data clustering using evidence accumulation. 2002 International Conference on Pattern Recognition,

[CR35] Fred, A. L. N., & Jain, A. K. (2005). Combining multiple clusterings using evidence accumulation. *IEEE Transactions on Pattern Analysis and Machine Intelligence,**27*(6), 835–850. 10.1109/TPAMI.2005.113 15943417

[CR36] Fu, L., Lin, P., Vasilakos, A. V., & Wang, S. (2020). An overview of recent multi-view clustering. *Neurocomputing,**402*, 148–161. 10.1016/j.neucom.2020.02.104

[CR37] Gao, C. X., Dwyer, D., Zhu, Y., Smith, C. L., Du, L., Filia, K. M., Bayer, J., Menssink, J. M., Wang, T., Bergmeir, C., Wood, S., & Cotton, S. M. (2023a). An overview of clustering methods with guidelines for application in mental health research. *Psychiatry Research,**327*, Article 115265. 10.1016/j.psychres.2023.115265 37348404

[CR38] Gao, C. X., Telford, N., Filia, K. M., Menssink, J., Albrecht, S., McGorry, P., Hamilton, M., Wang, M., Gan, D. Z., & Dwyer, D. (2023b). Capturing the clinical complexity in young people presenting to primary mental health services: A data-driven approach. *Epidemiology and Psychiatric Sciences*. 10.1017/s2045796024000386. Accepted. PMC11450420 39291560

[CR39] Gaujoux, R., & Seoighe, C. (2010). A flexible R package for nonnegative matrix factorization. *BMC Bioinformatics,**11*(1), 367. 10.1186/1471-2105-11-367 PMC2912887 20598126

[CR40] Ghaemi, R., Sulaiman, M. N., Ibrahim, H., & Mustapha, N. (2009). A survey: Clustering ensembles techniques. *International Journal of Computer and Information Engineering,**3*(2), 365–374.

[CR41] Gionis, A., Mannila, H., & Tsaparas, P. (2007). Clustering aggregation. *ACM Transactions on Knowledge Discovery from Data (TKDD),**1*(1), 4-es. 10.1145/1217299.1217303

[CR42] Golalipour, K., Akbari, E., Hamidi, S. S., Lee, M., & Enayatifar, R. (2021). From clustering to clustering ensemble selection: A review. *Engineering Applications of Artificial Intelligence,**104*, Article 104388. 10.1016/j.engappai.2021.104388

[CR43] Gordon, A. D., & Vichi, M. (2001). Fuzzy partition models for fitting a set of partitions. *Psychometrika,**66*(2), 229–247. 10.1007/Bf02294837

[CR44] Gu, Z. (2023). *cola: A Framework for Consensus Partitioning*. In (Version 2.8.0) R package. https://bioconductor.org/packages/cola/

[CR45] Gu, Z., Schlesner, M., & Hubschmann, D. (2021). Cola: An R/Bioconductor package for consensus partitioning through a general framework. *Nucleic Acids Research,**49*(3), Article e15. 10.1093/nar/gkaa1146 PMC7897501 33275159

[CR46] Hall, M.-H., Holton, K. M., Öngür, D., Montrose, D., & Keshavan, M. S. (2019). Longitudinal trajectory of early functional recovery in patients with first episode psychosis. *Schizophrenia Research,**209*, 234–244. 10.1016/j.schres.2019.02.003 PMC7003957 30826261

[CR47] Hornik, K. (2005). A clue for cluster ensembles. *Journal of Statistical Software,**14*(12), 1–25. 10.18637/jss.v014.i12

[CR48] Hornik, K., & Böhm, W. (2023). *clue: Cluster Ensembles*. In (Version 0.3–65) R package. https://CRAN.R-project.org/package=clue

[CR49] Huang, J. Z. (1997). A Fast Clustering Algorithm to Cluster Very Large Categorical Data Sets in Data Mining. Workshop on Research Issues on Data Mining and Knowledge Discovery,

[CR50] Iam-On, N., Boongoen, T., Garrett, S., & Price, C. (2011). A link-based approach to the cluster ensemble problem. *IEEE Transactions on Pattern Analysis and Machine Intelligence,**33*(12), 2396–2409. 10.1109/TPAMI.2011.84 21576752

[CR51] Iam-On, N., & Garrett, S. (2010). Linkclue: A MATLAB package for link-based cluster ensembles. *Journal of Statistical Software,**36*, 1–36. 10.18637/jss.v036.i09

[CR52] Jain, A. K. (2010). Data clustering: 50 years beyond K-means. *Pattern Recognition Letters,**31*(8), 651–666. 10.1016/j.patrec.2009.09.011

[CR53] Jain, B. J. (2018). The mean partition theorem in consensus clustering. *Pattern Recognition,**79*, 427–439. 10.1016/j.patcog.2018.01.030

[CR54] Jakobson, L. S., & Rigby, S. N. (2021). Alexithymia and sensory processing sensitivity: Areas of overlap and links to sensory processing styles. *Frontiers in Psychology*. 10.3389/fpsyg.2021.583786 PMC8182761 34108902

[CR55] Jia, W., Sun, M., Lian, J., & Hou, S. (2022). Feature dimensionality reduction: A review. *Complex & Intelligent Systems,**8*(3), 2663–2693. 10.1007/s40747-021-00637-x

[CR56] John, C. R., & Watson, D. (2023). *M3C: Monte Carlo Reference-based Consensus Clustering*. In (Version 1.24.0) R package. https://bioconductor.org/packages/M3C/10.1038/s41598-020-58766-1 PMC7000518 32020004

[CR57] John, C. R., Watson, D., Russ, D., Goldmann, K., Ehrenstein, M., Pitzalis, C., Lewis, M., & Barnes, M. (2020). M3C: Monte Carlo reference-based consensus clustering. *Scientific Reports,**10*(1), 1816. 10.1038/s41598-020-58766-1 PMC7000518 32020004

[CR58] Kiselev, V. Y. (2023). *SC3: Single-Cell Consensus Clustering*. In (Version 1.30.0) R package. https://bioconductor.org/packages/SC3/

[CR59] Kiselev, V. Y., Kirschner, K., Schaub, M. T., Andrews, T., Yiu, A., Chandra, T., Natarajan, K. N., Reik, W., Barahona, M., Green, A. R., & Hemberg, M. (2017). SC3: Consensus clustering of single-cell RNA-seq data. *Nature Methods,**14*(5), 483–486. 10.1038/nmeth.4236 PMC5410170 28346451

[CR60] Kleinberg, J. (2002). An impossibility theorem for clustering. *Advances in neural information processing systems*, *15*.

[CR61] Kuncheva, L. I., & Hadjitodorov, S. T. (2004). Using diversity in cluster ensembles. Conference Proceedings - IEEE International Conference on Systems, Man and Cybernetics,

[CR62] Li, F., Qian, Y., Wang, J., Dang, C., & Jing, L. (2019). Clustering ensemble based on sample’s stability. *Artificial Intelligence,**273*, 37–55. 10.1016/j.artint.2018.12.007

[CR63] Lin, H., Liu, H., Wu, J., Li, H., & Günnemann, S. (2023). Algorithm 1038: KCC: A MATLAB package for k-means-based consensus clustering. *ACM Transactions on Mathematical Software*. 10.1145/3616011

[CR64] Liu, H., Liu, T., Wu, J., Tao, D., & Fu, Y. (2015). Spectral ensemble clustering. *Proceedings of the 21th ACM SIGKDD international conference on knowledge discovery and data mining* (pp. 715–724). 10.1145/2783258.2783287

[CR65] Ma, T., Wang, F., Cheng, J., Yu, Y., & Chen, X. (2016). A hybrid spectral clustering and deep neural network ensemble algorithm for intrusion detection in sensor networks. *Sensors,**16*(10), Article 1701. 10.3390/s16101701 PMC5087489 27754380

[CR66] Manchia, M., Pisanu, C., Squassina, A., & Carpiniello, B. (2020). Challenges and future prospects of precision medicine in psychiatry. *Pharmacogenomics and Personalized Medicine,**13*, 127–140. 10.2147/pgpm.S198225 PMC7186890 32425581

[CR67] Margush, T., & McMorris, F. R. (1981). Consensusn-trees. *Bulletin of Mathematical Biology,**43*(2), 239–244. 10.1007/bf02459446

[CR68] Marquand, A. F., Wolfers, T., Mennes, M., Buitelaar, J., & Beckmann, C. F. (2016). Beyond lumping and splitting: A review of computational approaches for stratifying psychiatric disorders. *Biological Psychiatry: Cognitive Neuroscience and Neuroimaging,**1*(5), 433–447. 10.1016/j.bpsc.2016.04.002 PMC5013873 27642641

[CR69] Milligan, G. W. (1980). An examination of the effect of six types of error perturbation on fifteen clustering algorithms. *Psychometrika,**45*(3), 325–342. 10.1007/BF02293907

[CR70] Milligan, G. W., & Cooper, M. C. (1987). Methodology review: Clustering methods. *Applied Psychological Measurement,**11*(4), 329–354. 10.1177/014662168701100401

[CR71] Monti, S., Tamayo, P., Mesirov, J., & Golub, T. (2003). Consensus clustering: A resampling-based method for class discovery and visualization of gene expression microarray data. *Machine Learning,**52*(1), 91–118. 10.1023/A:1023949509487

[CR72] Nie, X., et al. (2023). Clustering ensemble in scRNA-seq data analysis: Methods, applications and challenges. *Computers in Biology and Medicine,**159*, Article 106939. 10.1016/j.compbiomed.2023.106939 37075602

[CR73] Oladottir, K., Wolf-Arehult, M., Ramklint, M., & Isaksson, M. (2022). Cluster analysis of personality traits in psychiatric patients with borderline personality disorder. *Borderline Personality Disorder and Emotion Dysregulation,**9*(1), Article 7. 10.1186/s40479-022-00178-w PMC8822819 35130981

[CR74] Pfeifer, B., Bloice, M. D., & Schimek, M. G. (2023). Parea: Multi-view ensemble clustering for cancer subtype discovery. *Journal of Biomedical Informatics,**143*, Article 104406. 10.1016/j.jbi.2023.104406 37257630

[CR75] Pierce, M., McManus, S., Hope, H., Hotopf, M., Ford, T., Hatch, S. L., John, A., Kontopantelis, E., Webb, R. T., Wessely, S., & Abel, K. M. (2021). Mental health responses to the COVID-19 pandemic: A latent class trajectory analysis using longitudinal UK data. *The Lancet Psychiatry,**8*(7), 610–619. 10.1016/S2215-0366(21)00151-6 PMC9764381 33965057

[CR76] Polikar, R. (2006). Ensemble based systems in decision making. *IEEE Circuits and Systems Magazine,**6*(3), 21–45. 10.1109/MCAS.2006.1688199

[CR77] Purdom, E., & Risso, D. (2023). *clusterExperiment: Compare Clusterings for Single-Cell Sequencing*. In (Version 2.22.0) R package. https://bioconductor.org/packages/clusterExperiment

[CR78] Rappoport, N., & Shamir, R. (2018). Multi-omic and multi-view clustering algorithms: Review and cancer benchmark. *Nucleic Acids Research,**46*(20), 10546–10562. 10.1093/nar/gky889 PMC6237755 30295871

[CR79] Risso, D., Purvis, L., Fletcher, R. B., Das, D., Ngai, J., Dudoit, S., & Purdom, E. (2018). ClusterExperiment and RSEC: A Bioconductor package and framework for clustering of single-cell and other large gene expression datasets. *PLoS Computational Biology,**14*(9), Article e1006378. 10.1371/journal.pcbi.1006378 PMC6138422 30180157

[CR80] Sæther, L. S., Ueland, T., Haatveit, B., Maglanoc, L. A., Szabo, A., Djurovic, S., Aukrust, P., Roelfs, D., Mohn, C., Ormerod, M. B. E. G., Lagerberg, T. V., Steen, N. E., Melle, I., Andreassen, O. A., & Ueland, T. (2023). Inflammation and cognition in severe mental illness: Patterns of covariation and subgroups. *Molecular Psychiatry,**28*(3), 1284–1292. 10.1038/s41380-022-01924-w PMC10005942 36577840

[CR81] Șenbabaoğlu, Y., Michailidis, G., & Li, J. Z. (2014). Critical limitations of consensus clustering in class discovery. *Scientific Reports,**4*(1), Article 6207. 10.1038/srep06207 PMC4145288 25158761

[CR82] Shireman, E. M., Steinley, D., & Brusco, M. J. (2016). Local optima in mixture modeling. *Multivariate Behavioral Research,**51*(4), 466–481. 10.1080/00273171.2016.1160359 PMC5534344 27494191

[CR83] Simpson, T. I. (2022). *clusterCons: Consensus Clustering using Multiple Algorithms and Parameters*. In (Version 1.2) R package. https://CRAN.R-project.org/package=clusterCons

[CR84] Simpson, T. I., Armstrong, J. D., & Jarman, A. P. (2010). Merged consensus clustering to assess and improve class discovery with microarray data. *BMC Bioinformatics,**11*(1), Article 590. 10.1186/1471-2105-11-590 PMC3002369 21129181

[CR85] Steinley, D. (2003). Local optima in K-means clustering: What you don’t know may hurt you. *Psychological Methods,**8*(3), 294–304. 10.1037/1082-989x.8.3.294 14596492

[CR86] Strehl, A., & Ghosh, J. (2002). Cluster ensembles - a knowledge reuse framework for combining multiple partitions. *Journal of machine learning research*,* 3*(Dec), 583–617. 10.1162/153244303321897735

[CR87] Tan, Z., Lu, Z., & Shen, C. (2023 ). *BCClong: Bayesian Consensus Clustering for Multiple Longitudinal Features*. In (Version 1.0.1) R package. https://CRAN.R-project.org/package=BCClong

[CR88] Tan, Z., Shen, C., Subbarao, P., Lou, W., & Lu, Z. (2022). A Joint Modeling Approach for Clustering Mixed-Type Multivariate Longitudinal Data: Application to the CHILD Cohort Study. *arXiv*. 10.48550/arXiv.2210.08385

[CR89] Thorndike, R. L. (1953). Who belongs in the family? *Psychometrika,**18*(4), 267–276. 10.1007/BF02289263

[CR90] Topchy, A., Jain, A. K., & Punch, W. (2004). A mixture model for clustering ensembles. Proceedings of the 2004 SIAM international conference on data mining,

[CR91] Tryon, R. C. (1939). *Cluster analysis; correlation profiles and orthometric (factor) analysis for the isolation of unities in mind and personality* (p. 122). Edwards.

[CR92] Ullmann, T., Hennig, C., & Boulesteix, A.-L. (2022). Validation of cluster analysis results on validation data: A systematic framework. *Wires Data Mining and Knowledge Discovery,**12*(3), Article e1444. 10.1002/widm.1444

[CR93] Van Der Maaten, L., Postma, E. O., & van den Herik, H. J. (2009). Dimensionality reduction: A comparative review. *Journal of Machine Learning Research,**10*(66–71), 13.

[CR94] Vega-Pons, S., & Ruiz-Shulcloper, J. (2011). A survey of clustering ensemble algorithms. *International Journal of Pattern Recognition and Artificial Intelligence,**25*(03), 337–372. 10.1142/S0218001411008683

[CR95] Wen, Z., Hammoud, M. Z., Siegel, C. E., Laska, E. M., Abu-Amara, D., Etkin, A., Milad, M. R., & Marmar, C. R. (2025). Neuroimaging-based variability in subtyping biomarkers for psychiatric heterogeneity. *Molecular Psychiatry,**30*(5), 1966–1975. 10.1038/s41380-024-02807-y PMC12015113 39511450

[CR96] Wilkerson, M. D., & Hayes, D. N. (2010). ConsensusClusterPlus: A class discovery tool with confidence assessments and item tracking. *Bioinformatics,**26*(12), 1572–1573. 10.1093/bioinformatics/btq170 PMC2881355 20427518

[CR97] Wilkerson, M. D., & Waltman, P. (2020). *ConsensusClusterPlus*. In (Version 1.66.0) R package. https://bioconductor.org/packages/ConsensusClusterPlus/

[CR98] Wolpert, D. H., & Macready, W. G. (1997). No free lunch theorems for optimization. *IEEE Transactions on Evolutionary Computation,**1*(1), 67–82. 10.1109/4235.585893

[CR99] Wright, A. G. C., & Woods, W. C. (2020). Personalized models of psychopathology. *Annual Review of Clinical Psychology,**16*, 49–74. 10.1146/annurev-clinpsy-102419-125032 32070120

[CR100] Wu, J., Liu, H., Xiong, H., Cao, J., & Chen, J. (2015). K-means-based consensus clustering: A unified view. *IEEE Transactions on Knowledge and Data Engineering,**27*(1), 155–169. 10.1109/TKDE.2014.2316512

[CR101] Wu, X., Ma, T., Cao, J., Tian, Y., & Alabdulkarim, A. (2018). A comparative study of clustering ensemble algorithms. *Computers & Electrical Engineering,**68*, 603–615. 10.1016/j.compeleceng.2018.05.005

[CR102] Xanthopoulos, P. (2014). A review on consensus clustering methods. In T. M. Rassias, C. A. Floudas, & S. Butenko (Eds.), *Optimization in science and engineering: In honor of the 60th birthday of Panos M. Pardalos* (pp. 553–566). Springer New York. 10.1007/978-1-4939-0808-0_26

[CR103] Xiao, X., Rabe-Hesketh, S., & Skrondal, A. (2025). Bayesian identification and estimation of growth mixture models. *Psychometrika,**90*(2), 442–475. 10.1017/psy.2025.11 PMC12483708 40190064

[CR104] Xu, C., Tao, D., & Xu, C. (2013). A survey on multi-view learning. *arXiv preprint *arXiv:1304.5634.

[CR105] Yang, Y., & Wang, H. (2018). Multi-view clustering: A survey. *Big Data Mining and Analytics,**1*(2), 83–107.

[CR106] Yoon, C. K. (2009). *Naming nature: The clash between instinct and science*. WW Norton & Company.

[CR107] Yu, Y., Ge, R., & Frangou, S. (2026). Neuroimaging-based subgroups in schizophrenia: A critical appraisal of clustering studies. *Biological Psychiatry: Global Open Science*. 10.1016/j.bpsgos.2026.100745 PMC13272521 42318040

[CR108] Yu, Z., Li, L., Wong, H.-S., You, J., Han, G., Gao, Y., & Yu, G. (2014). Probabilistic cluster structure ensemble. *Information Sciences,**267*, 16–34. 10.1016/j.ins.2014.01.030

[CR109] Yu, Z., Wong, H. S., & Wang, H. (2007). Graph-based consensus clustering for class discovery from gene expression data. *Bioinformatics,**23*(21), 2888–2896. 10.1093/bioinformatics/btm463 17872912

[CR110] Zhang, M. (2022). Weighted clustering ensemble: A review. *Pattern Recognition,**124*, Article 108428. 10.1016/j.patcog.2021.108428

[CR111] Zhou, Z.-H., & Tang, W. (2006). Clusterer ensemble. *Knowledge-Based Systems,**19*(1), 77–83. 10.1016/j.knosys.2005.11.003

