# Supplementary material for: Ensemble clustering: A practical tutorial
Source: Behav Res Methods. 2026 Sep 3;58(10):286. doi: 10.3758/s13428-026-03158-y (PMC13541929; doi:10.3758/s13428-026-03158-y)
Supplement: Supplementary file 1 [file 13428_2026_3158_MOESM2_ESM.pdf]

Load libraries
Generate source data
Simulated data
Visualise clusters
Randomly order the data
Save data

Ensemble clustering: A practical tutorial

Supplementary Material II: Data generation process

Load libraries

```
library(pacman)
p_load("tidyverse", "ggplot2", "Rdimtools", "ggthemes","mice","mnorm")
## Provides detailed information and environment settings for the current R sess
ion
sessionInfo()
```

```
## R version 4.2.2 (2022-10-31 ucrt)
## Platform: x86_64-w64-mingw32/x64 (64-bit)
## Running under: Windows 10 x64 (build 19044)
##
## Matrix products: default
##
## locale:
## [1] LC_COLLATE=English_Australia.utf8  LC_CTYPE=English_Australia.utf8
## [3] LC_MONETARY=English_Australia.utf8 LC_NUMERIC=C
## [5] LC_TIME=English_Australia.utf8
##
## attached base packages:
## [1] stats     graphics  grDevices utils     datasets  methods   base
##
## other attached packages:
##  [1] mnorm_1.2.2     mice_3.15.0     ggthemes_5.1.0  Rdimtools_1.1.2
##  [5] lubridate_1.9.2 forcats_1.0.0   stringr_1.5.0   dplyr_1.1.0
##  [9] purrr_1.0.1     readr_2.1.4     tidyr_1.3.0     tibble_3.1.8
## [13] ggplot2_3.4.1   tidyverse_2.0.0 pacman_0.5.1
##
## loaded via a namespace (and not attached):
##  [1] bit64_4.0.5          doParallel_1.0.17    tools_4.2.2
##  [4] backports_1.4.1      bslib_0.4.2          utf8_1.2.3
##  [7] R6_2.5.1             colorspace_2.1-0     withr_2.5.0
## [10] tidyselect_1.2.0     maotai_0.2.5         bit_4.0.5
## [13] compiler_4.2.2       cli_3.6.0            sass_0.4.5
## [16] RcppDE_0.1.7         scales_1.2.1         digest_0.6.31
## [19] dbscan_1.1-12        rmarkdown_2.20       base64enc_0.1-3
## [22] pkgconfig_2.0.3      htmltools_0.5.4      fastmap_1.1.0
## [25] htmlwidgets_1.6.1    rlang_1.0.6          rstudioapi_0.14
## [28] jquerylib_0.1.4      generics_0.1.3       jsonlite_1.8.4
## [31] magrittr_2.0.3       Matrix_1.5-1         Rcpp_1.0.10
## [34] munsell_0.5.0        fansi_1.0.4          lifecycle_1.0.3
## [37] CVXR_1.0-12          scatterplot3d_0.3-42 stringi_1.7.12
## [40] yaml_2.3.7           mclustcomp_0.3.3     MASS_7.3-58.1
## [43] Rtsne_0.17           grid_4.2.2           parallel_4.2.2
## [46] lattice_0.20-45      hms_1.1.2            knitr_1.42
## [49] pillar_1.9.0         fastcluster_1.2.6    ADMM_0.3.3
## [52] codetools_0.2-18     glue_1.6.2           evaluate_0.20
## [55] RcppParallel_5.1.6   vctrs_0.5.2          tzdb_0.3.0
## [58] Rdpack_2.6           foreach_1.5.2        gtable_0.3.1
## [61] RANN_2.6.1           cachem_1.0.6         xfun_0.37
## [64] rbibutils_2.2.16     broom_1.0.3          Rmpfr_0.9-5
## [67] pracma_2.4.4         RSpectra_0.16-1      minpack.lm_1.2-4
## [70] iterators_1.0.14     shapes_1.2.7         hpa_1.3.3
## [73] cluster_2.1.4        rgl_1.3.1            gmp_0.7-4
## [76] timechange_0.2.0     ellipsis_0.3.2
```

Generate source data

Simulated data

```
# Prepare multivariate normal vector parameters
# expected value
mean <- c(2.5, 2.5, 3)
n_dim <- length(mean)

# correlation matrix
cor <- c( 1, 0.3, 0.1,
0.3, 1, 0.2,
0.1, 0.2, 1)
cor <- matrix(cor, ncol = n_dim, nrow = n_dim, byrow = TRUE)

# covariance matrix
sd_mat <- diag(c(1.2, 1.2, 1))
sigma <- sd_mat %*% cor %*% t(sd_mat)

# Generate data from this distribution
d1<-rmnorm(n = 300, mean = mean, sigma = sigma)


# Prepare multivariate normal vector parameters
# expected value
mean <- c(0, 0, -1)
n_dim <- length(mean)
# correlation matrix
cor <- c( 1, 0.3, 0.1,
0.3, 1, 0.2,
0.1, 0.2, 1)
cor <- matrix(cor, ncol = n_dim, nrow = n_dim, byrow = TRUE)

# covariance matrix
sd_mat <- diag(c(0.7, 0.7, 0.5))
sigma <- sd_mat %*% cor %*% t(sd_mat)

# Generate data from this distribution
d2<-rmnorm(n = 150, mean = mean, sigma = sigma)


# Prepare multivariate normal vector parameters
# expected value
mean <- c(5, -1, -3)
n_dim <- length(mean)

# correlation matrix
cor <- c( 1, 0.3, 0.1,
0.3, 1, 0.2,
0.1, 0.2, 1)
cor <- matrix(cor, ncol = n_dim, nrow = n_dim, byrow = TRUE)

# covariance matrix
sd_mat <- diag(c(1, 1, 0.7))
sigma <- sd_mat %*% cor %*% t(sd_mat)

# Generate data from this distribution
```

```
d3<-rmnorm(n = 150, mean = mean, sigma = sigma)

 #Merge d1, d2, and d3 along the rows
source <- rbind(d1,d2,d3)
names(source)<-c("X1","X2","X3")

## Establish group class for each observation
group <- c(rep("Group1",300),rep("Group2",150),rep("Group3",150))


# Random projection into 10 variables
set.seed(1234)
dta <- lapply(1:3, function(x) do.rndproj(source,ndim = 3)$Y)

## Combine the data in the generated list 'dta' into a data frame
dta <- rlist::list.cbind(dta)
dta<-base::cbind(source,dta)
names(dta)<-paste("V",1:12)


## Convert 'dta' to a tibble data frame
dta <- as_tibble(dta)

## Add a column named 'group' name to data
dta$group <- group

## Add additional noise and outliers
# Add noise
set.seed(1234)
for (i in 1:10) {
    sd <- runif(1, 0.1, 1)
    dta[,i] <- dta[,i] + rnorm(nrow(dta), 0, sd )
}

## Add a few outliers
dta[30,1] <- 15
dta[60,4] <- -9
dta[250,6] <- 15
dta[556,10] <- 18
```

Visualise clusters

The generated clusters can be visualised by PCA

```
## Perform Principal Component Analysis (PCA) on the first 10 columns of 'dta'
res.pca <- FactoMineR::PCA(dta %>%  select(V1:V12),
                           scale.unit = TRUE,
                           ncp = 3,
                           graph = FALSE)

factoextra::fviz_pca_ind(res.pca,
             geom.ind = "point",
             col.ind = dta$group, # color by groups
             palette = c("#A0DCFF", "#A6A6A6", "#419AB0"),
             addEllipses = TRUE, # Concentration ellipses
             legend.title = "Groups",
             axes=c(1,2))
```

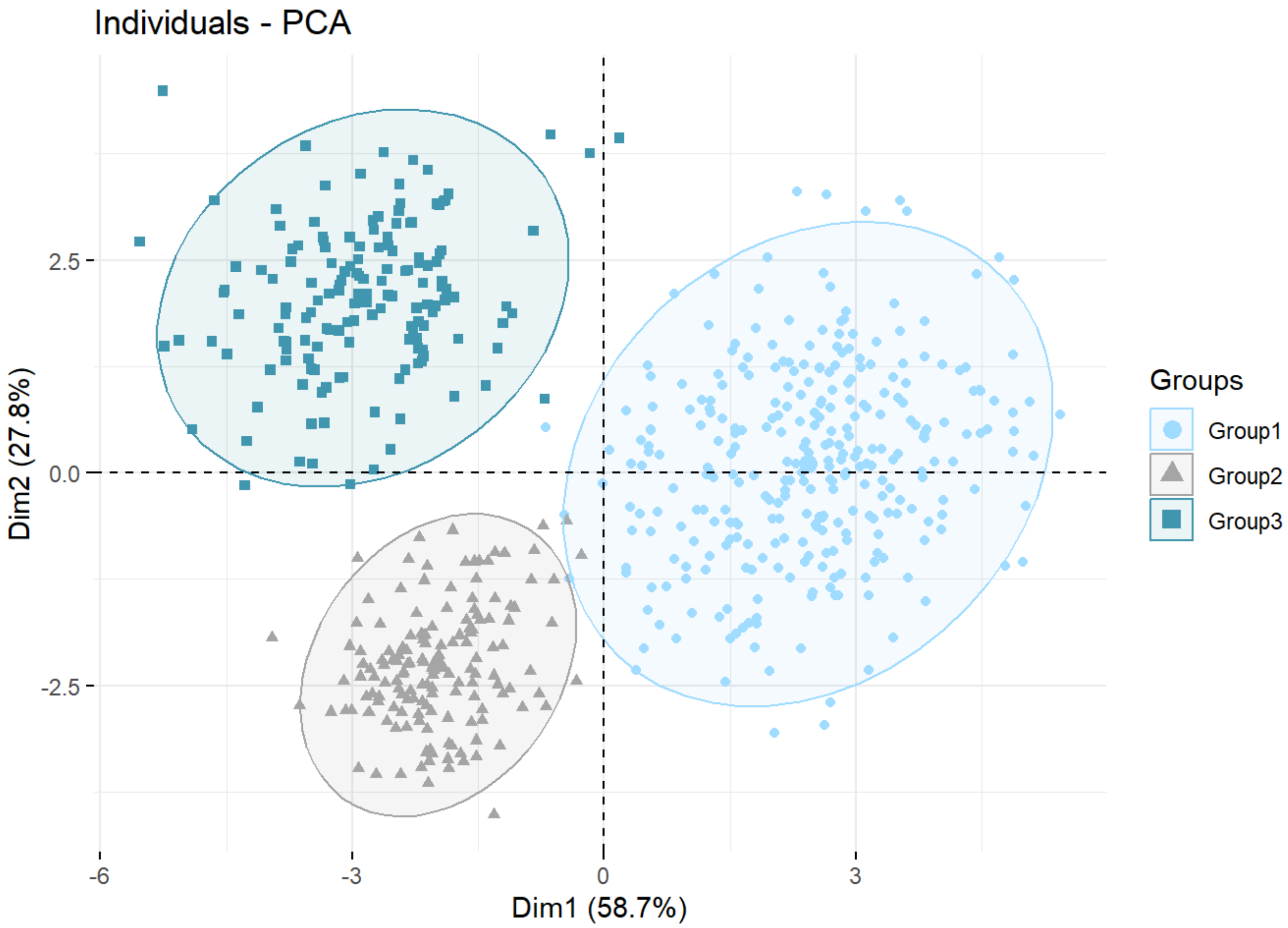


```
factoextra::fviz_pca_ind(res.pca,
             geom.ind = "point",
             col.ind = dta$group, # color by groups
             palette = c("#A0DCFF", "#A6A6A6", "#419AB0"),
             addEllipses = TRUE, # Concentration ellipses
             legend.title = "Groups",
             axes=c(1,3))
```

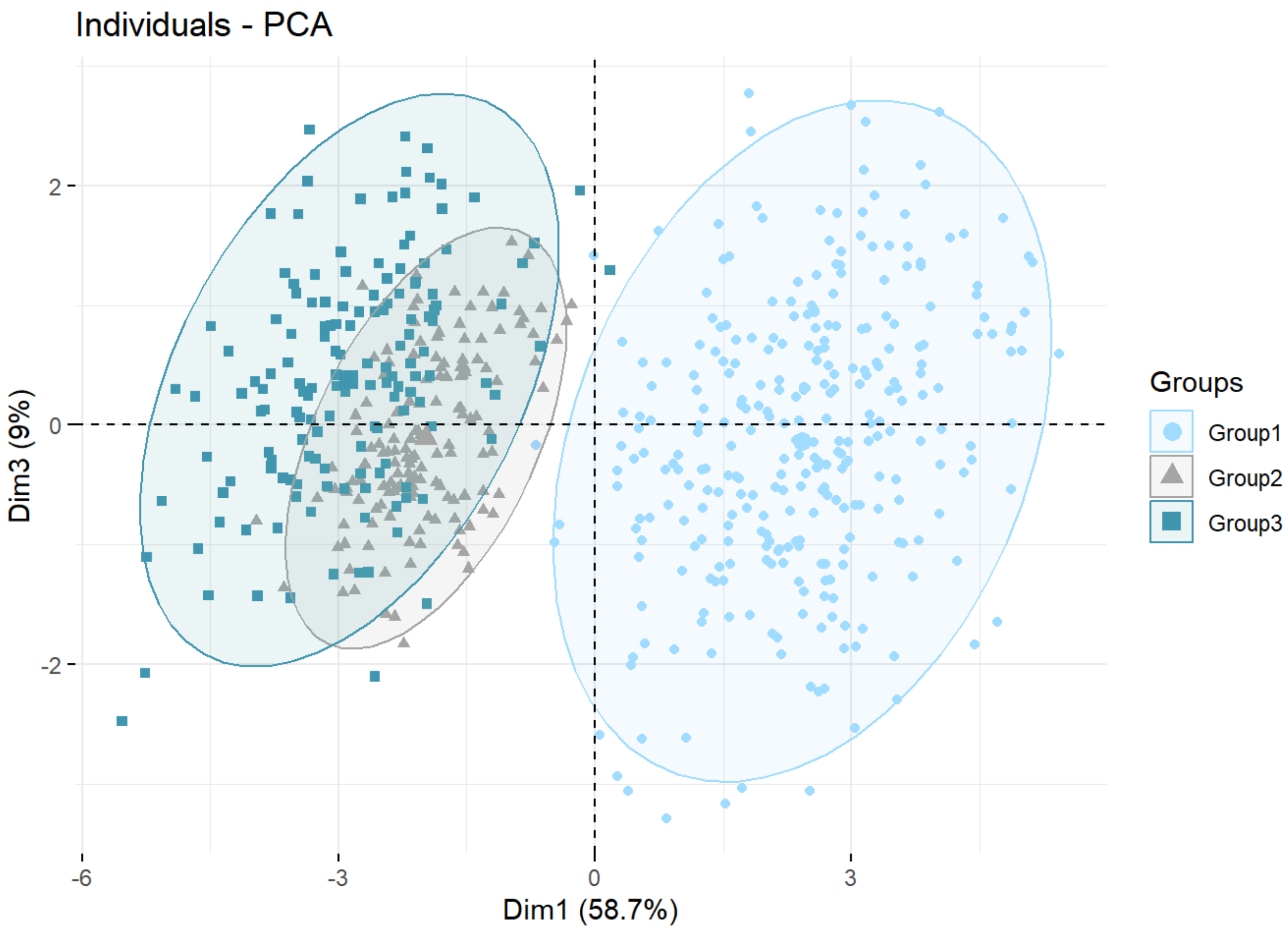


```
factoextra::fviz_pca_ind(res.pca,
             geom.ind = "point",
             col.ind = dta$group, # color by groups
             palette = c("#A0DCFF", "#A6A6A6", "#419AB0"),
             addEllipses = TRUE, # Concentration ellipses
             legend.title = "Groups",
             axes=c(2,3))
```

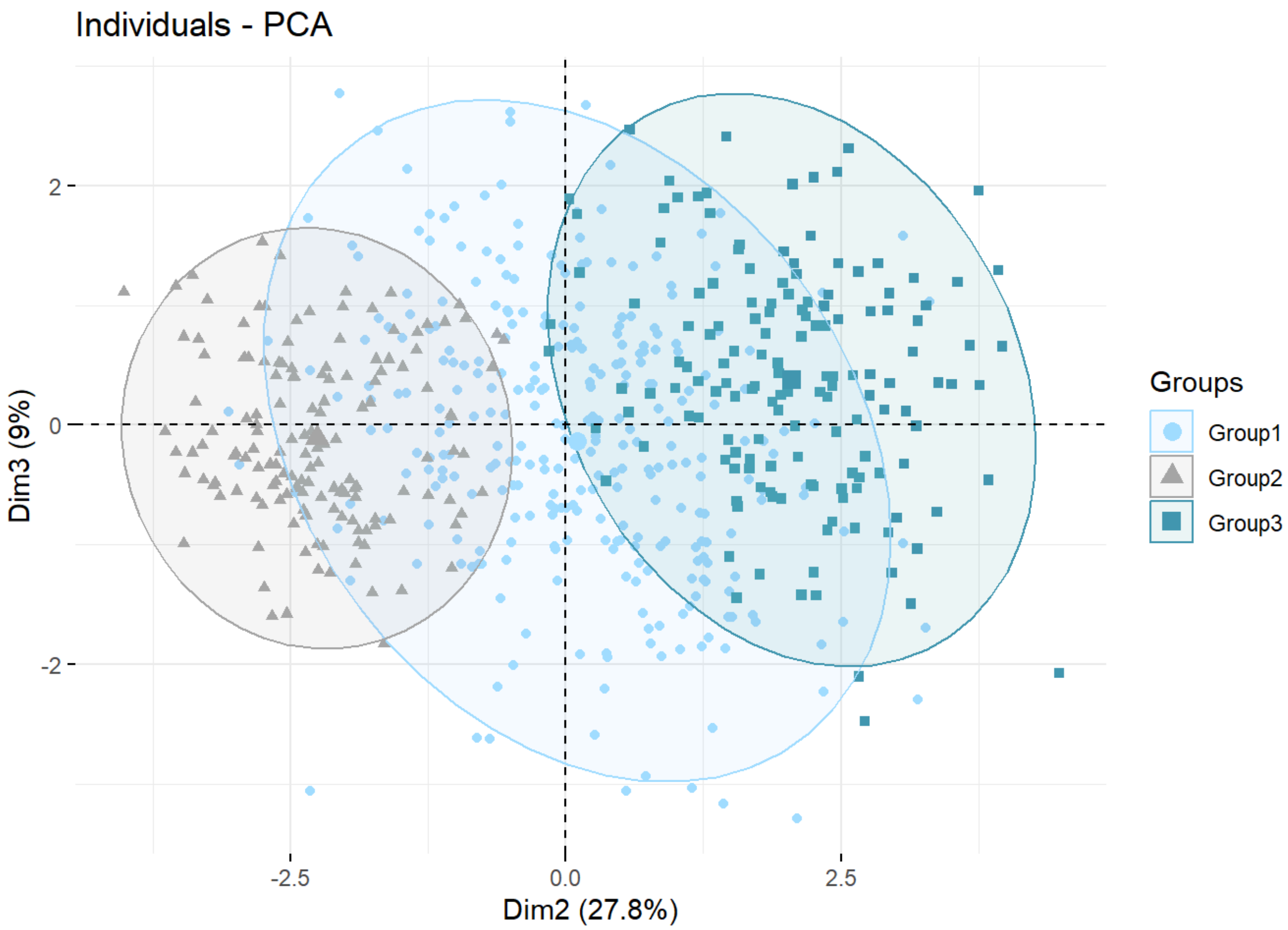

Randomly order the data

```
## Random order the rows of 'dta'
set.seed(12345)
dta <- dta[sample(1:nrow(dta)), ]

## Add "ID"
dta <- tibble::rownames_to_column(dta,var = "ID")  %>%
  mutate(ID=as.numeric(ID))

## Store group name separately
group <- dta %>% select(ID, group)


## Keep clustering variables
dta <- dta %>% select(ID,V1:V12)
```

Save data

```
saveRDS(dta,here::here("source_data.rds"))
```

```
saveRDS(group,here::here("source_data_with_label.rds"))
```
