# Supplementary material for: Ensemble clustering: A practical tutorial
Source: Behav Res Methods. 2026 Sep 3;58(10):286. doi: 10.3758/s13428-026-03158-y (PMC13541929; doi:10.3758/s13428-026-03158-y)
Supplement: Supplementary file 2 [file 13428_2026_3158_MOESM3_ESM.pdf]

Load libraries
Import data
Inspecting Data
Data Frame Summary
Pre-processing
Outliers
Correlations
Dimensionality reduction
Normalise data
Dimensionality reduction
Generate base clusterings (BCs)
Use diceR package
Missing data imputation
Manually generate BCs
Establish consensus
Majority voting
Consensus matrix
Binary encoding clustering
Integrated functions
Choosing optimal k
Permutation test
Missing data

Ensemble clustering: A practical tutorial

Supplementary Material III: Step by step guide

Load libraries

```
library(pacman)
p_load(
  "tidyverse", "ggplot2", "gridExtra", "kableExtra", "knitr", "here",
  "qgraph", "arsenal", "stringr", "Hmisc", "tidyselect", "readxl", "gdata",
  "ggthemes", "scales", "FactoMineR", "smacof", "networktools", "Rtsne",
  "factoextra", "ConsensusClusterPlus", "NMF", "diceR", "RclusTool",
  "data.table", "aricode","dummy_cols" ,"VIM","poLCA", "parallel", "patchwork")
## Provides detailed information and environment settings for the current R session
sessionInfo()
```

```
## R version 4.5.1 (2025-06-13)
## Platform: aarch64-apple-darwin20
## Running under: macOS Tahoe 26.4.1
## 
## Matrix products: default
## BLAS:   /Library/Frameworks/R.framework/Versions/4.5-arm64/Resources/lib/libRblas.0.dylib 
## LAPACK: /Library/Frameworks/R.framework/Versions/4.5-arm64/Resources/lib/libRlapack.dylib;  LAPACK version 3.12.1
## 
## locale:
## [1] en_US.UTF-8/en_US.UTF-8/en_US.UTF-8/C/en_US.UTF-8/en_US.UTF-8
## 
## time zone: Australia/Melbourne
## tzcode source: internal
## 
## attached base packages:
##  [1] parallel  grid      tcltk     stats     graphics  grDevices utils    
##  [8] datasets  methods   base     
## 
## other attached packages:
##  [1] patchwork_1.3.2            poLCA_1.6.0.2             
##  [3] MASS_7.3-65                scatterplot3d_0.3-44      
##  [5] VIM_7.0.0                  aricode_1.0.3             
##  [7] data.table_1.18.2.1        RclusTool_0.91.61         
##  [9] tkrplot_0.0-32             tcltk2_1.6.1              
## [11] diceR_3.1.0                NMF_0.28                  
## [13] Biobase_2.70.0             BiocGenerics_0.56.0       
## [15] generics_0.1.4             cluster_2.1.8.1           
## [17] rngtools_1.5.2             registry_0.5-1            
## [19] ConsensusClusterPlus_1.74.0 factoextra_1.0.7          
## [21] Rtsne_0.17                 networktools_1.6.0        
## [23] smacof_2.1-7               e1071_1.7-16              
## [25] colorspace_2.1-2           plotrix_3.8-4             
## [27] FactoMineR_2.12            scales_1.4.0              
## [29] ggthemes_5.1.0             gdata_3.0.1               
## [31] readxl_1.4.5               tidyselect_1.2.1          
## [33] Hmisc_5.2-4                arsenal_3.6.3             
## [35] qgraph_1.9.8               here_1.0.2                
## [37] knitr_1.50                 kableExtra_1.4.0          
## [39] gridExtra_2.3              lubridate_1.9.4           
## [41] forcats_1.0.1              stringr_1.5.2             
## [43] dplyr_1.2.1                purrr_1.1.0               
## [45] readr_2.1.5                tidyr_1.3.2               
## [47] tibble_3.3.0               ggplot2_4.0.2             
## [49] tidyverse_2.0.0            pacman_0.5.1              
## 
## loaded via a namespace (and not attached):
##   [1] RColorBrewer_1.1-3   doParallel_1.0.17    tools_4.5.1         
##   [4] mlr3learners_0.14.0  backports_1.5.0      R6_2.6.1            
##   [7] DT_0.34.0            jomo_2.7-6           withr_3.0.2         
##  [10] sp_2.2-0             fdrtool_1.2.18       cli_3.6.5           
##  [13] textshaping_1.0.4    flashClust_1.01-2    sandwich_3.1-1      
##  [16] sass_0.4.10          nnls_1.6             mvtnorm_1.3-3       
##  [19] S7_0.2.0             robustbase_0.99-6    randomForest_4.7-1.2
##  [22] proxy_0.4-27         pbapply_1.7-4        pbivnorm_0.6.0      
##  [25] mlr3tuning_1.5.1     systemfonts_1.3.1    foreign_0.8-90      
##  [28] svglite_2.2.2        R.utils_2.13.0       paradox_1.0.1       
##  [31] parallelly_1.46.1    rstudioapi_0.17.1    shape_1.4.6.1       
##  [34] gtools_3.9.5         car_3.1-3            leaps_3.2           
##  [37] Matrix_1.7-3         abind_1.4-8          R.methodsS3_1.8.2   
##  [40] weights_1.1.2        lifecycle_1.0.5      multcomp_1.4-29     
##  [43] yaml_2.3.10          carData_3.0-5        lavaan_0.6-20       
##  [46] crayon_1.5.3         mitml_0.4-5          lattice_0.22-7      
##  [49] pillar_1.11.1        boot_1.3-31          estimability_1.5.1  
##  [52] corpcor_1.6.10       codetools_0.2-20     pan_1.9             
##  [55] glue_1.8.0           vcd_1.4-13           vctrs_0.7.1         
##  [58] png_0.1-8            Rdpack_2.6.4         cellranger_1.1.0    
##  [61] gtable_0.3.6         cachem_1.1.0         xfun_0.56           
##  [64] rbibutils_2.3        coda_0.19-4.1        reformulas_0.4.1    
##  [67] survival_3.8-3       iterators_1.0.14     TH.data_1.1-5       
##  [70] nlme_3.1-168         bbotk_1.8.1          rprojroot_2.1.1     
##  [73] mlr3pipelines_0.10.0 bslib_0.9.0          mlr3_1.3.0          
##  [76] rpart_4.1.24         nnet_7.3-20          mlr3misc_0.19.0     
##  [79] mnormt_2.1.1         emmeans_2.0.0        compiler_4.5.1      
##  [82] mda_0.5-5            glmnet_4.1-10        htmlTable_2.4.3     
##  [85] lgr_0.5.2            mice_3.19.0          xml2_1.4.0          
##  [88] checkmate_2.3.3      lmtest_0.9-40        DEoptimR_1.1-4      
##  [91] psych_2.5.6          quadprog_1.5-8       multcompView_0.1-10 
##  [94] palmerpenguins_0.1.1 digest_0.6.37        minqa_1.2.8         
##  [97] rmarkdown_2.30       htmltools_0.5.8.1    pkgconfig_2.0.3     
## [100] jpeg_0.1-11          base64enc_0.1-3      lme4_1.1-37         
## [103] fastmap_1.2.0        rlang_1.1.7          htmlwidgets_1.6.4   
## [106] farver_2.1.2         jquerylib_0.1.4      zoo_1.8-15          
## [109] jsonlite_2.0.0       mmand_1.7.0          mclust_6.1.2        
## [112] eigenmodel_1.11      R.oo_1.27.1          magrittr_2.0.4      
```

```
## [115] polynom_1.4-1       Formula_1.2-5       wordcloud_2.6
## [118] Rcpp_1.1.0          stringi_1.8.7       plyr_1.8.9
## [121] conclust_1.1        listenv_0.10.0      ggrepel_0.9.6
## [124] splines_4.5.1       hms_1.1.3           igraph_2.2.0
## [127] uuid_1.2-1          ranger_0.18.0       reshape2_1.4.4
## [130] stats4_4.5.1        evaluate_1.0.5      BiocManager_1.30.27
## [133] laeken_0.5.3        nloptr_2.2.1        tzdb_0.5.0
## [136] foreach_1.5.2       future_1.69.0       reshape_0.8.10
## [139] gridBase_0.4-7      broom_1.0.10        xtable_1.8-4
## [142] glasso_1.11         viridisLite_0.4.2   class_7.3-23
## [145] SearchTrees_0.5.5   ellipse_0.5.0       corrplot_0.95
## [148] timechange_0.3.0    globals_0.19.0
```

```
# ensure select and filter function always sourced from dplyr package
conflicted::conflict_prefer("select", "dplyr")
```

```
## [conflicted] Will prefer dplyr::select over any other package.
```

```
conflicted::conflict_prefer("filter", "dplyr")
```

```
## [conflicted] Will prefer dplyr::filter over any other package.
```

```
conflicted::conflict_prefer("combine", "dplyr")
```

```
## [conflicted] Will prefer dplyr::combine over any other package.
```

Import data

```
# Import source data
dta <- readRDS(here::here("source_data.rds"))
# Import group labels for source data
dta_group <- readRDS(here::here("source_data_with_label.rds"))
```

Inspecting Data

We first produce statistical summaries for each variable within the 'dta' dataset, detailing the total number of variables, the count of observations, and the properties of each variable such as data types, distribution patterns, presence of missing values, average values, standard deviations, medians, and other relevant metrics.

```
print(summarytools::dfSummary(dta, graph.magnif = 0.75), method = "render")
```

Data Frame Summary

dta

Dimensions: 600 x 13
Duplicates: 0

No	Variable	Stats / Values	Freqs (% of Valid)	Graph	Valid	Missing
1	ID [numeric]	Mean (sd) : 300.5 (173.3)<br>min ≤ med ≤ max:<br>1 ≤ 300.5 ≤ 600<br>IQR (CV) : 299.5 (0.6)	600 distinct values	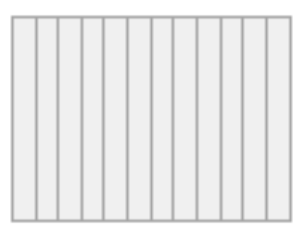	600 (100.0%)	0 (0.0%)
2	V1 [numeric]	Mean (sd) : 2.5 (2.1)<br>min ≤ med ≤ max:<br>-1.8 ≤ 2.7 ≤ 15<br>IQR (CV) : 3.2 (0.8)	600 distinct values	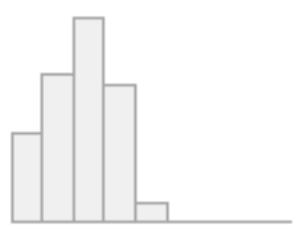	600 (100.0%)	0 (0.0%)
3	V2 [numeric]	Mean (sd) : 1 (2)<br>min ≤ med ≤ max:<br>-4.8 ≤ 0.9 ≤ 5.8<br>IQR (CV) : 3.1 (1.9)	600 distinct values	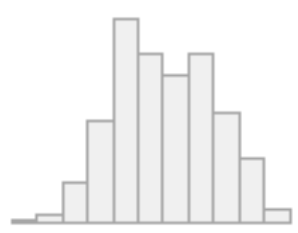	600 (100.0%)	0 (0.0%)
4	V3 [numeric]	Mean (sd) : 0.5 (2.9)<br>min ≤ med ≤ max:<br>-6 ≤ 0.4 ≤ 6.8<br>IQR (CV) : 4.9 (5.7)	600 distinct values	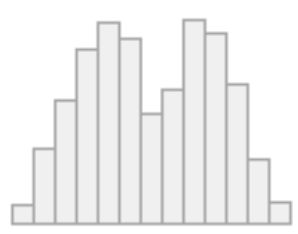	600 (100.0%)	0 (0.0%)
5	V4 [numeric]	Mean (sd) : 1.4 (3.2)<br>min ≤ med ≤ max:<br>-9 ≤ 1 ≤ 7.9<br>IQR (CV) : 5.5 (2.3)	600 distinct values	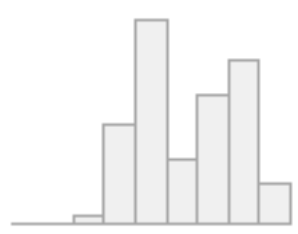	600 (100.0%)	0 (0.0%)

No	Variable	Stats / Values	Freqs (% of Valid)	Graph	Valid	Missing
6	V5 [numeric]	Mean (sd) : 2.7 (2.2)<br>min ≤ med ≤ max:<br>-3 ≤ 3 ≤ 7.5<br>IQR (CV) : 3.2 (0.8)	600 distinct values	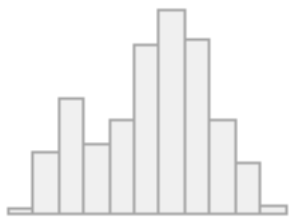	600 (100.0%)	0 (0.0%)
7	V6 [numeric]	Mean (sd) : 2.3 (2.2)<br>min ≤ med ≤ max:<br>-2.8 ≤ 2.2 ≤ 15<br>IQR (CV) : 3.2 (0.9)	600 distinct values	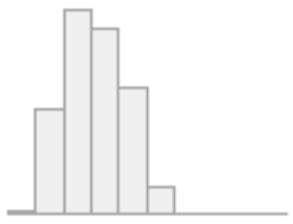	600 (100.0%)	0 (0.0%)
8	V7 [numeric]	Mean (sd) : 2.2 (2.8)<br>min ≤ med ≤ max:<br>-3 ≤ 1.9 ≤ 8.8<br>IQR (CV) : 5 (1.3)	600 distinct values	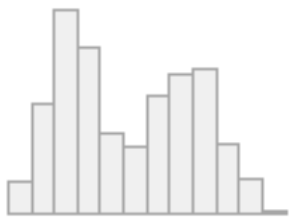	600 (100.0%)	0 (0.0%)
9	V8 [numeric]	Mean (sd) : -0.5 (1.1)<br>min ≤ med ≤ max:<br>-4.3 ≤ -0.4 ≤ 2.2<br>IQR (CV) : 1.6 (-2.1)	600 distinct values	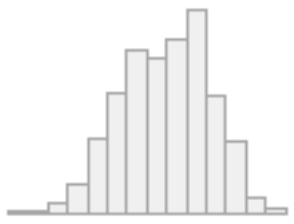	600 (100.0%)	0 (0.0%)
10	V9 [numeric]	Mean (sd) : 0.1 (1.6)<br>min ≤ med ≤ max:<br>-4.4 ≤ 0.1 ≤ 4.2<br>IQR (CV) : 2.3 (28.4)	600 distinct values	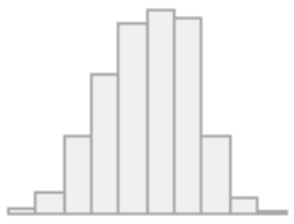	600 (100.0%)	0 (0.0%)
11	V10 [numeric]	Mean (sd) : 2 (3)<br>min ≤ med ≤ max:<br>-3.4 ≤ 1.6 ≤ 18<br>IQR (CV) : 5.5 (1.5)	600 distinct values	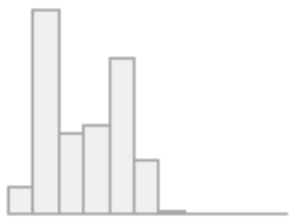	600 (100.0%)	0 (0.0%)
12	V11 [numeric]	Mean (sd) : -1.7 (3.1)<br>min ≤ med ≤ max:<br>-8.2 ≤ -1.3 ≤ 3.7<br>IQR (CV) : 5.7 (-1.8)	600 distinct values	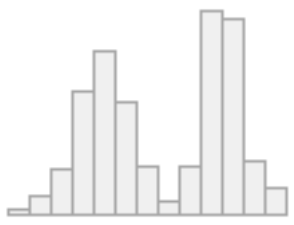	600 (100.0%)	0 (0.0%)
13	V12 [numeric]	Mean (sd) : -0.1 (1.6)<br>min ≤ med ≤ max:<br>-5.7 ≤ 0.2 ≤ 3.5<br>IQR (CV) : 2 (-22.3)	600 distinct values	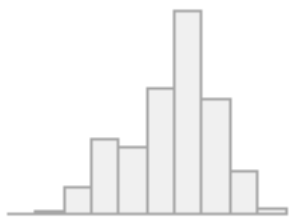	600 (100.0%)	0 (0.0%)

Generated by summarytools (https://github.com/dcomtois/summarytools) 1.1.5 (R (https://www.r-project.org/) version 4.5.1)
2026-05-23

```
psych::describe(dta)
```

```
##     vars   n   mean     sd median trimmed    mad   min    max  range  skew
## ID     1 600 300.50 173.35 300.50  300.50 222.39  1.00 600.00 599.00  0.00
## V1     2 600   2.52   2.09   2.72    2.50   2.31 -1.78  15.00  16.78  0.31
## V2     3 600   1.04   1.99   0.93    1.03   2.31 -4.81   5.85  10.66  0.06
## V3     4 600   0.52   2.95   0.41    0.53   3.64 -5.99   6.84  12.83 -0.01
## V4     5 600   1.42   3.23   0.99    1.43   4.17 -9.00   7.91  16.91 -0.01
## V5     6 600   2.71   2.21   3.04    2.77   2.33 -3.00   7.50  10.50 -0.30
## V6     7 600   2.30   2.17   2.21    2.24   2.37 -2.80  15.00  17.80  0.45
## V7     8 600   2.23   2.79   1.87    2.17   3.62 -2.98   8.78  11.76  0.16
## V8     9 600  -0.53   1.12  -0.44   -0.50   1.19 -4.31   2.18   6.49 -0.21
## V9    10 600   0.06   1.58   0.15    0.09   1.68 -4.40   4.25   8.65 -0.17
## V10   11 600   2.03   3.05   1.61    1.94   4.02 -3.38  18.00  21.38  0.35
## V11   12 600  -1.72   3.06  -1.34   -1.66   4.05 -8.16   3.75  11.91 -0.13
## V12   13 600  -0.07   1.57   0.17    0.01   1.38 -5.71   3.47   9.18 -0.49
##     kurtosis   se
## ID     -1.21 7.08
## V1      1.05 0.09
## V2     -0.73 0.08
## V3     -1.06 0.12
## V4     -1.14 0.13
## V5     -0.75 0.09
## V6      0.99 0.09
## V7     -1.26 0.11
## V8     -0.44 0.05
## V9     -0.50 0.06
## V10    -0.30 0.12
## V11    -1.45 0.12
## V12    -0.20 0.06
```

The distribution of data can be assessed by analyzing the box plots of the variables.

```
# Reshape data long
long_df <- pivot_longer(dta, cols = V1:V12,
                        names_to = "variable",
                        values_to = "value")

# box plot printed in panels
ggplot(long_df, aes(x = variable, y = value)) +
  geom_boxplot() +
  facet_wrap(~variable, scales = "free_x", nrow = 1) +
  theme_minimal()
```

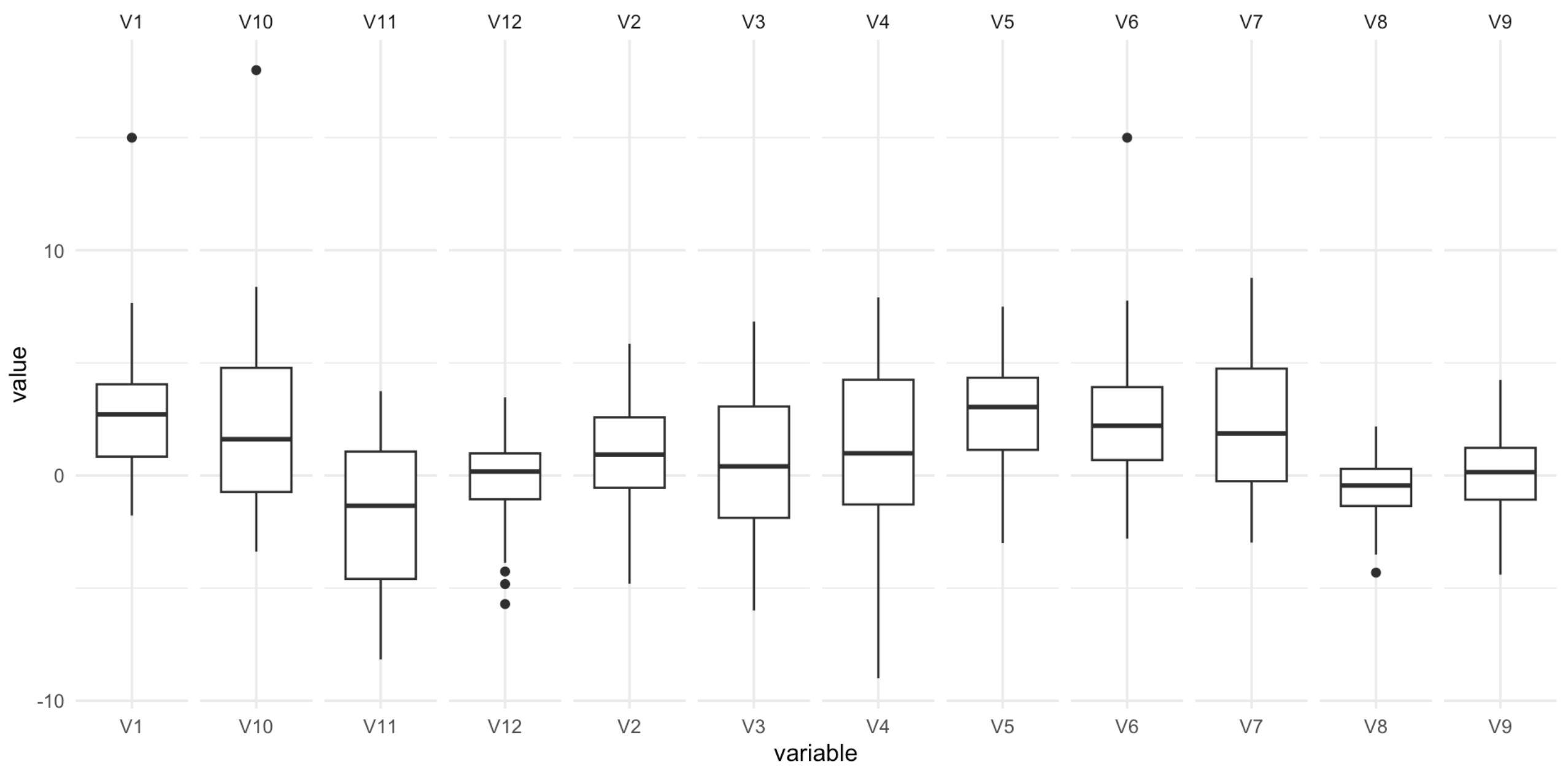

Pre-processing

Outliers

The presence of several significant outliers has been observed, which could markedly affect the effectiveness of clustering algorithms like k-means. Consequently, we have decided to exclude these outliers from our analysis, applying a threshold of three times the interquartile range (IQR) as the criterion for removal.

```
nrow(dta)
```

```
## [1] 600
```

```
# loop all variables
for (var in paste0("V", 1:12)) {
    # Obtain IQR
    Q <- quantile(dta[[var]], probs = c(.25, .75), na.rm = TRUE)
    iqr <- IQR(dta[[var]], na.rm = TRUE) # Calculate IQR
    lower_bound <- Q[2] - 3 * iqr # Define lower boundary
    upper_bound <- Q[1] + 3 * iqr # Define upper boundary

    # remove outlier if there is any in the variable
    if (any(dta[[var]] < lower_bound | dta[[var]] > upper_bound)) {
        dta <-
            dta %>% filter(dta[[var]] > lower_bound & dta[[var]] < upper_bound)
    }
}
nrow(dta)
```

```
## [1] 596
```

```
# Remove the same observations in data labels
dta_group <- dta_group %>%
    filter(ID %in% dta$ID)
```

A few records were removed.

Correlations

In the final step, we will assess the pairwise correlations among the variables.

```
library(pheatmap)
# Choose colours and breaks for the plot
paletteLength <- 30
cols <- colorRampPalette(c("#045a8d", "white", "#cb181d"))(paletteLength)
brks <- seq(-1, 1, length.out = paletteLength)

# Plot the heatmap
plot <-
    pheatmap(
        cor(dta %>% select(V1:V12)),
        color = cols,
        breaks = brks,
        display_numbers = TRUE
    )
```

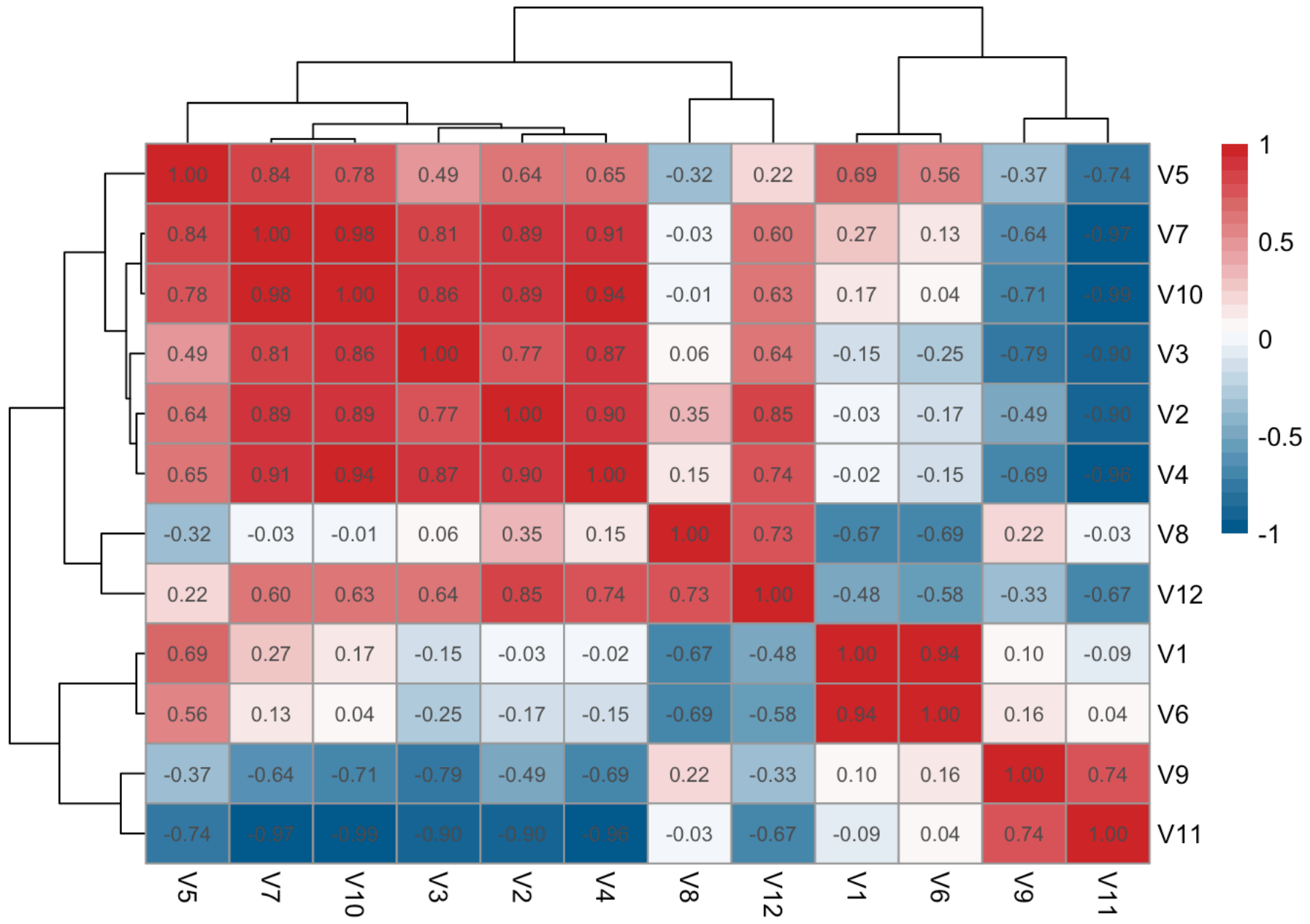

The variables exhibited a high degree of correlation, which is attributed to the nature of the random projection model employed. This strong correlation underpins the justification for implementing dimensionality reduction before proceeding with clustering.

Dimensionality reduction

Normalise data

If only normalisation is needed, without dimensionality reduction, the following code can be used.

```
## The 'scale(.)' function normalizes each column by setting the mean to 0 and the standard deviation to 1,
## The 'as.numeric' converts the scaled values to numerical data.
dta_norm <- dta %>%
  dplyr::mutate_at(vars(V1:V12), ~ as.numeric(scale(.)))

# Check data
psych::describe(dta_norm)
```

```
##     vars   n   mean     sd median trimmed    mad   min    max  range  skew
## ID     1 596 299.65 173.11 299.50  299.49 222.39  1.00 600.00 599.00  0.00
## V1     2 596   0.00   1.00   0.11    0.00   1.15 -2.11   2.57   4.68 -0.02
## V2     3 596   0.00   1.00  -0.06   -0.01   1.17 -2.97   2.43   5.40  0.09
## V3     4 596   0.00   1.00  -0.04    0.00   1.23 -2.21   2.15   4.36 -0.01
## V4     5 596   0.00   1.00  -0.14    0.00   1.29 -3.25   2.02   5.26  0.00
## V5     6 596   0.00   1.00   0.15    0.03   1.05 -2.58   2.17   4.75 -0.29
## V6     7 596   0.00   1.00  -0.03   -0.02   1.12 -2.41   2.62   5.03  0.15
## V7     8 596   0.00   1.00  -0.13   -0.02   1.30 -1.87   2.35   4.21  0.16
## V8     9 596   0.00   1.00   0.08    0.02   1.07 -2.69   2.43   5.12 -0.18
## V9    10 596   0.00   1.00   0.06    0.02   1.06 -2.81   2.64   5.45 -0.17
## V10   11 596   0.00   1.00  -0.17   -0.03   1.31 -1.81   2.14   3.95  0.15
## V11   12 596   0.00   1.00   0.12    0.02   1.32 -2.11   1.79   3.90 -0.14
## V12   13 596   0.00   1.00   0.15    0.05   0.89 -3.08   2.28   5.36 -0.44
##     kurtosis   se
## ID     -1.20 7.09
## V1     -0.84 0.04
## V2     -0.78 0.04
## V3     -1.06 0.04
## V4     -1.14 0.04
## V5     -0.76 0.04
## V6     -0.71 0.04
## V7     -1.26 0.04
## V8     -0.56 0.04
## V9     -0.50 0.04
## V10    -1.42 0.04
## V11    -1.45 0.04
## V12    -0.36 0.04
```

```
saveRDS(dta_norm, "cleaned_data.rds")
```

Dimensionality reduction

We employ PCA for dimensionality reduction.

```
# Random seed to ensure reproducibility of the results
set.seed(1234)
# Use prcomp function for PCA
PCA <- prcomp(dta %>% select(V1:V12),
              center = TRUE,
              scale. = TRUE) # standardise data

# Plot variances of dimensions
fviz_eig(PCA)
```

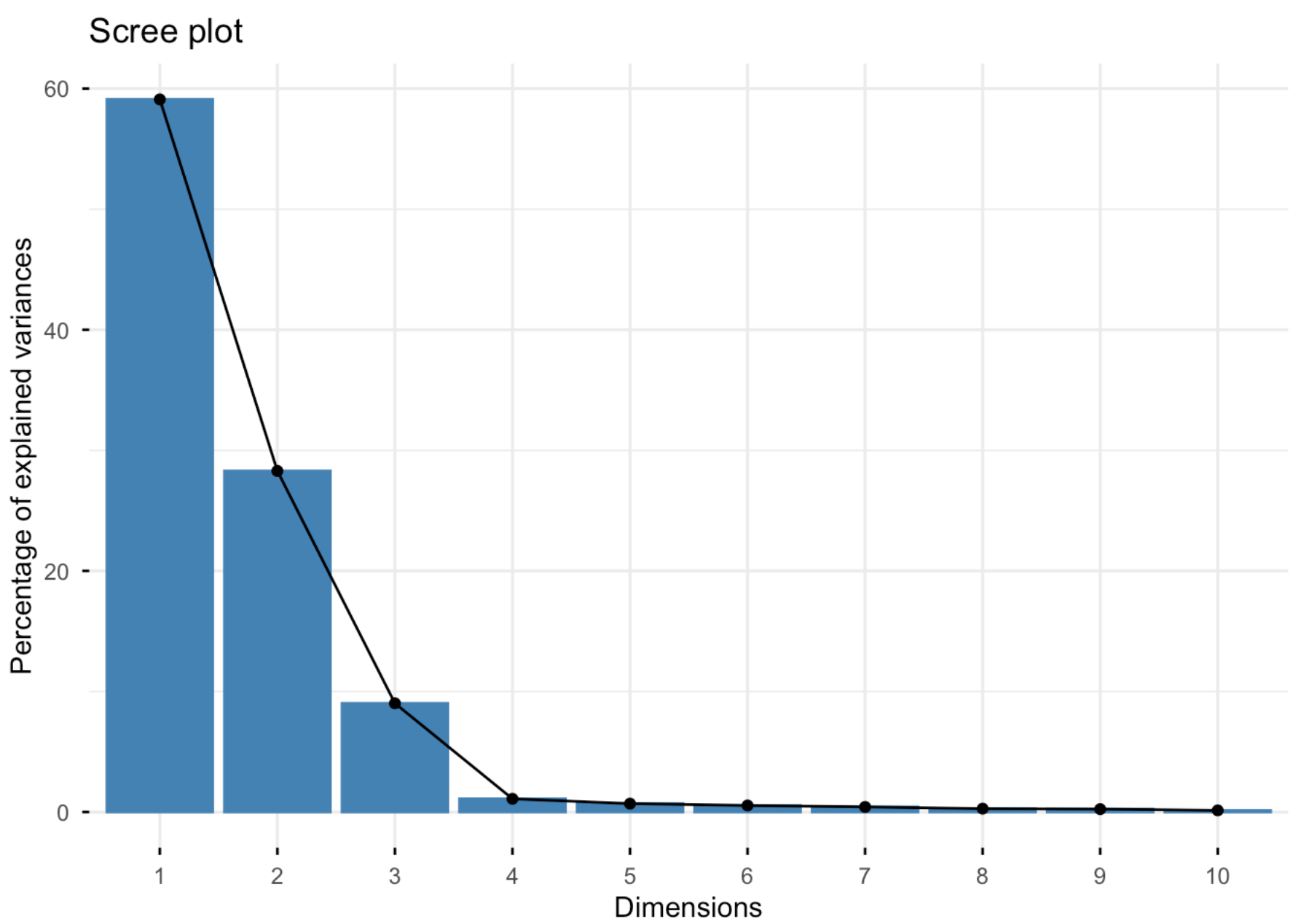

The initial three dimensions captured the majority of the variance, a result of the data generation method. The optimal number of dimensions can be selected in several ways, for example, by applying the elbow method and then retaining one or two additional components beyond the elbow point to ensure that all meaningful variation is captured, or by relying on cross-validation performance metrics. In this case, we will retain the first five dimensions in the subsequent analysis.

```
# prcomp package do not standardise extracted components and we will do this manually. This step is not necessary if using pri
ncipal function in psych package
dta_pca <- as_tibble(PCA$x[, 1:5]) %>%
    mutate_all( ~ as.numeric(scale(.)))

# Add ID and keep it the first column
dta_pca <- dta_pca %>%
    mutate(ID = dta$ID) %>%
    relocate(ID)

psych::describe(dta_pca)
```

```
##     vars   n   mean     sd median trimmed    mad   min    max  range  skew
## ID     1 596 299.65 173.11 299.50  299.49 222.39  1.00 600.00 599.00  0.00
## PC1    2 596   0.00   1.00  -0.12   -0.01   1.31 -2.09   2.05   4.14  0.10
## PC2    3 596   0.00   1.00   0.07    0.00   1.17 -2.21   2.20   4.41 -0.04
## PC3    4 596   0.00   1.00   0.01   -0.01   0.97 -2.69   3.18   5.88  0.16
## PC4    5 596   0.00   1.00  -0.01    0.00   0.98 -2.81   3.32   6.13  0.00
## PC5    6 596   0.00   1.00   0.04    0.02   0.98 -4.18   2.93   7.11 -0.25
##     kurtosis   se
## ID     -1.20 7.09
## PC1    -1.37 0.04
## PC2    -0.96 0.04
## PC3     0.16 0.04
## PC4     0.05 0.04
## PC5     0.26 0.04
```

Similarly, we can use the cmdscale function from the stats package for dimensionality reduction.

```
# Random seed to ensure reproducibility of the results
set.seed(123456)
## 'dist' is used to calculate the distance of variables; 'cmdscale' is used to perform multidimensional scaling analysis
dta_mds <- stats::cmdscale(dist(dta %>% select(V1:V12)),
                           k = 5, # specifies the number of dimensions to reduce to
                           add = FALSE # Not add input matrix to the the results
                           )

# Standardise extracted data
dta_mds <- as_tibble(dta_mds) %>%
    mutate_all(~ as.numeric(scale(.)))

# Check data
psych::describe(dta_mds)
```

```
##    vars   n mean sd median trimmed  mad   min   max range  skew kurtosis   se
## V1    1 596    0  1   0.16    0.02 1.30 -2.04  1.94  3.98 -0.13    -1.41 0.04
## V2    2 596    0  1  -0.02    0.01 1.19 -2.50  2.01  4.51 -0.08    -0.87 0.04
## V3    3 596    0  1  -0.03   -0.03 0.97 -2.80  3.39  6.19  0.30     0.35 0.04
## V4    4 596    0  1  -0.03    0.01 1.02 -5.52  2.78  8.29 -0.28     1.01 0.04
## V5    5 596    0  1   0.01   -0.02 0.80 -2.54 14.13 16.67  4.69    65.22 0.04
```

Many other dimensionality reduction models can be used:

- Non-negative matrix factorization using nmf function from NMF package
- Kernel PCA using kpca from kernlab package
- t-distributed stochastic neighbor embedding (t-SNE) using Rtsne function from Rtsne package
- Multiple correspondence analysis (MCA) for categorical variables using MCA fom FactoMineR package
- Categorical Principal Components Analysis (CATPCA) using princals function from Gifi package
- Autoencoder using autoencode from ruta package

Generate base clusterings (BCs)

Use diceR package

We can use consensus_cluster from diceR package go generate BCs, which includes diverse methods: * “nmf”: Nonnegative Matrix Factorization (using Kullback-Leibler Divergence or Euclidean distance; See Note for specifications.) * “hc”: Hierarchical Clustering * “diana”: DIvisive ANAlysis Clustering * “km”: K-Means Clustering * “pam”: Partition Around Medoids * “ap”: Affinity Propagation * “sc”: Spectral Clustering using Radial-Basis kernel function * “gmm”: Gaussian Mixture Model using Bayesian Information Criterion on EM algorithm * “block”: Co-clustering using a multiple latent block model * “som”: Self-Organizing Map (SOM) with Hierarchical Clustering * “cmeans”: Fuzzy C-Means Clustering * “hdbscan”: Hierarchical Density-based Spatial Clustering of Applications with Noise (HDBSCAN)

Initially, we experiment with the k-means as the BC algorithm, using 100 random samples comprising 80% of the original data to create BCs (the consensus_cluster function exclusively samples observations). In this particular example, the k-means algorithm faces a greater challenge due to the non-spherical shapes of clusters, uneven distribution of cluster sizes, and diverse cluster densities. Individual BCs generated from k-means may presented with higher diversity with the data, therefore we use a larger number of BCs. In scenarios with lower diversity, fewer BCs would be adequate.

```
## BCs can be generated using consensus_cluster function.
set.seed(1234567)
BCs_diceR_km <- diceR::consensus_cluster(
    dta_pca %>% select(-ID),
    nk = 3, # Number of clusters for BCs
    p.item = 0.8,  # Randomly selected 80% of the original data
    reps = 100, # Generate 100 BCs,
    algorithms = c("km") # BC algorithms we use k-means
)

# consensus_cluster store data in a 4-dimensional array: rows are samples, columns are different bootstrap subsample replicate
s,the 3th dimension is for algorithms, and the 4th dimension is for different k.
str(BCs_diceR_km)
```

```
##  int [1:596, 1:100, 1, 1] 3 3 3 1 2 3 3 3 NA 1 ...
##  - attr(*, "dimnames")=List of 4
##   ..$ : chr [1:596] "1" "2" "3" "4" ...
##   ..$ : chr [1:100] "R1" "R2" "R3" "R4" ...
##   ..$ : chr "KM"
##   ..$ : chr "3"
```

Diversity among BCs can be evaluated using the Rand Index (RI) or the Adjusted Rand Index (ARI). The RI quantifies the agreement proportion for all observation pairs between two clustering outcomes, while the ARI, as a chance-corrected version of the RI, adjusts for random agreement.

```
# Function calculate ARI for the ith and jth column of the data. If users interested in calculating RI, aricode::RI function c
an be used instead.
cal_ARI_ij<-function(i,j,data){
    return<-1
    if(i!=j){
        #
        data<-data[,c(i,j)] %>% na.omit()
        return<-aricode::ARI(data[,1],data[,2])
    }
    return
}

# Function calculating RI for all pairs of columns in the data
ARI<-function(cluster_results){
  # Calculate pairwise Rand index
  cal_ARI <- Vectorize(cal_ARI_ij, vectorize.args=list("i","j"))
  ARI <- outer(1:length(cluster_results),1:length(cluster_results),
              cal_ARI,data=cluster_results)
  diag(ARI) <- NA
  return(ARI)
}

agreement<-ARI(as.data.frame(BCs_diceR_km))
hist(as.vector(agreement))
```

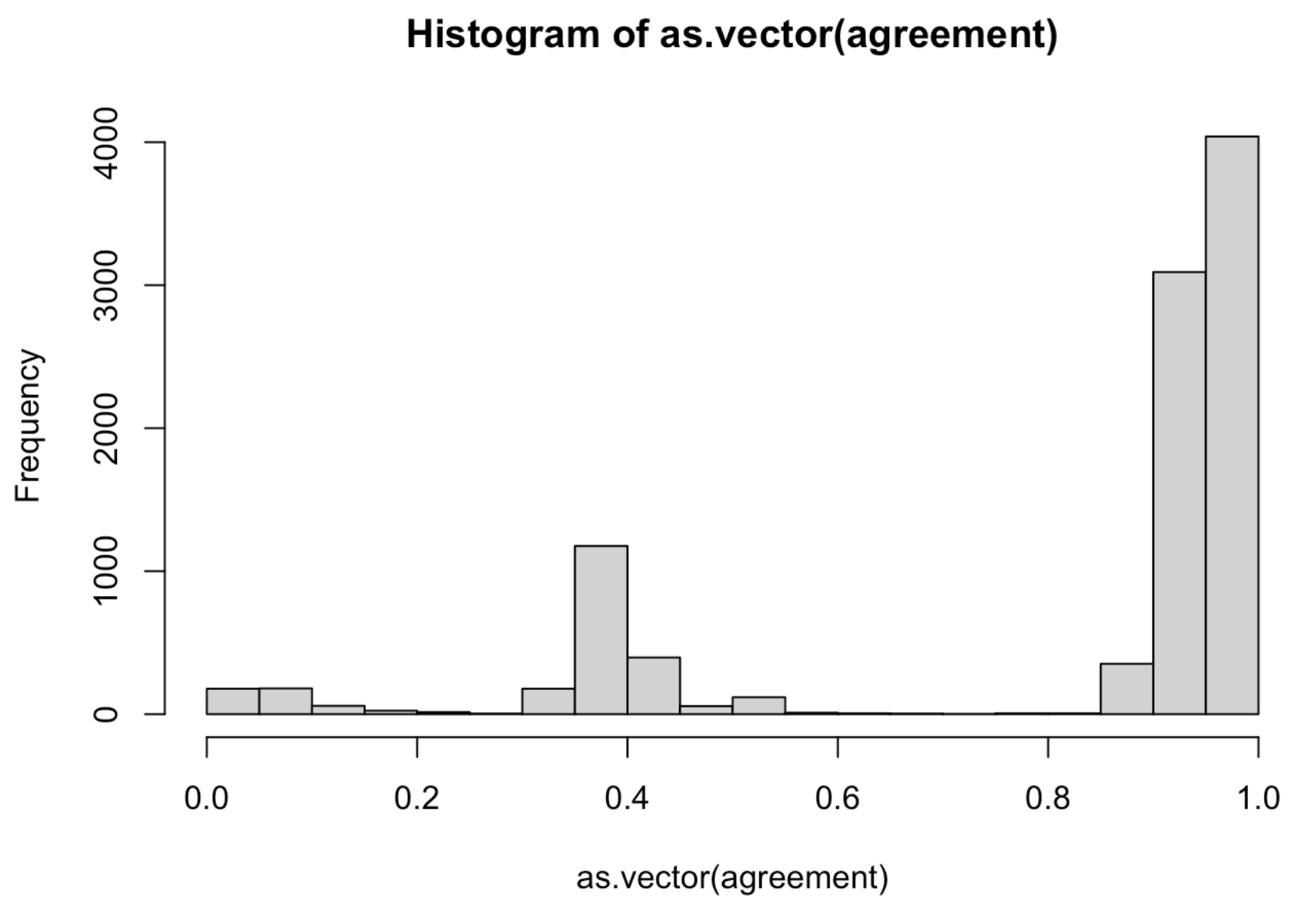

In this specific data, employing the Gaussian Mixture Model (GMM) will result in BCs that exhibit near-perfect agreement, as shown in the demonstration below. This high level of agreement is attributed to the fact that the data was generated from multivariate normal distributions.

```
set.seed(1234)
BCs_diceR_gmm <- diceR::consensus_cluster(dta_pca %>% select(-ID),
  nk = 3, # Number of clusters for BCs
  p.item = 0.8, # Randomly selected 80% of the original data for constructing base clusters;
  reps = 100,  # Generate 100 BCs,
  algorithms = c("gmm") # BC algorithms we use gmm
)

agreement<-ARI(as.data.frame(BCs_diceR_gmm))
hist(as.vector(agreement))
```

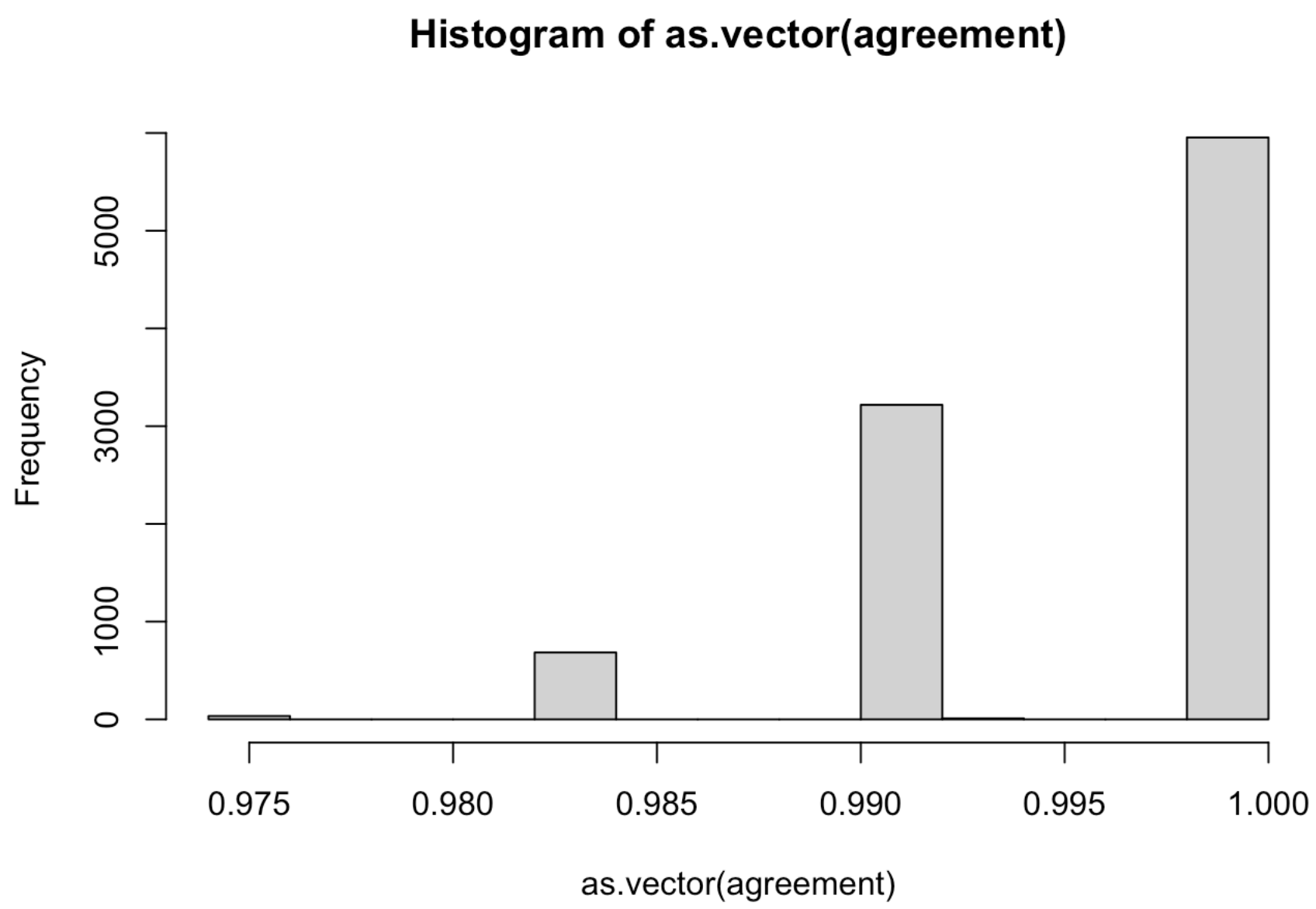

In practical applications, GMM and various other BC algorithms might offer more benefits compared to k-means, e.g., not assuming spherical clusters. However, k-means stands out as the fastest algorithm, especially for handling large datasets.

Missing data imputation

BCs include missing data as a part of sampling observations, which can be addressed with imputation. The diceR package utilizes K-Nearest Neighbours (KNN) for imputation. However, this method doesn't ensure that all missing values are filled, necessitating an additional step of majority voting for complete imputation.

```
# KNN imputation
BCs_imputed<- apply(BCs_diceR_km,
                    2:4,
                    diceR::impute_knn,
                    data = dta_pca %>% select(-ID),
                    seed = 1234)
# majority voting
BCs_imputed <- impute_missing(BCs_imputed, dta_pca %>% select(-ID), nk = 3) # nk represents number of clusters
```

Other imputation packages, e.g., mice, missRanger can also be used to impute the missing cluster labels (as factor variable).

Manually generate BCs

The BC generation process can also be programmed manually. We create a function for this task as followed.

```
# here we create a user defined function to make calling this part of the code easier.
gen_base_clustering<-function(data, n){
  # data structure to store the results
  BCs <-data[,"ID"]
  # loop individual BCs
  for(i in 1:n) {
    # Sample observations and variables
    sample_rows <- sample(1:nrow(data), size = round(nrow(data) * 0.8))
    sample_cols <- sample(2:ncol((data)), size = round((ncol(data)-1) * 0.8)) # exclude ID
    sample_data<-data[sample_rows, c(1,sample_cols)]

    # obtain PCA
    pca<-prcomp(sample_data %>% select(-ID),
                center = TRUE,
                scale. = TRUE)
    pca<-as_tibble(pca$x[, 1:4]) %>%
        mutate_all( ~ as.numeric(scale(.)))

    # K-means clustering with 3 clusters
    model<-kmeans(pca,  centers=3,nstart=10)
    # Store results and merge back to main data
    cluster<-as_tibble(base::cbind(sample_data$ID,model$cluster))
    names(cluster)<-c("ID", paste0("C",i) )
    # Join BC back to results
    BCs<-BCs %>%
      left_join(cluster,by = join_by(ID))
  }
  #remove ID
  BCs<-BCs %>%
    select(-ID) %>%
    mutate_all(as.numeric)
}

# Generate BCs
set.seed(12345)
BCs_manual<-gen_base_clustering(dta_pca,n=100)
```

The ‘gen_base_clustering’ function can be modified in various ways to align with the specific objectives of the users, e.g., different number of clusters for BCs, different proportion of observations and variables to be sampled, different algorithms etc.

```
agreement<-ARI(as.data.frame(BCs_manual))
hist(as.vector(agreement))
```

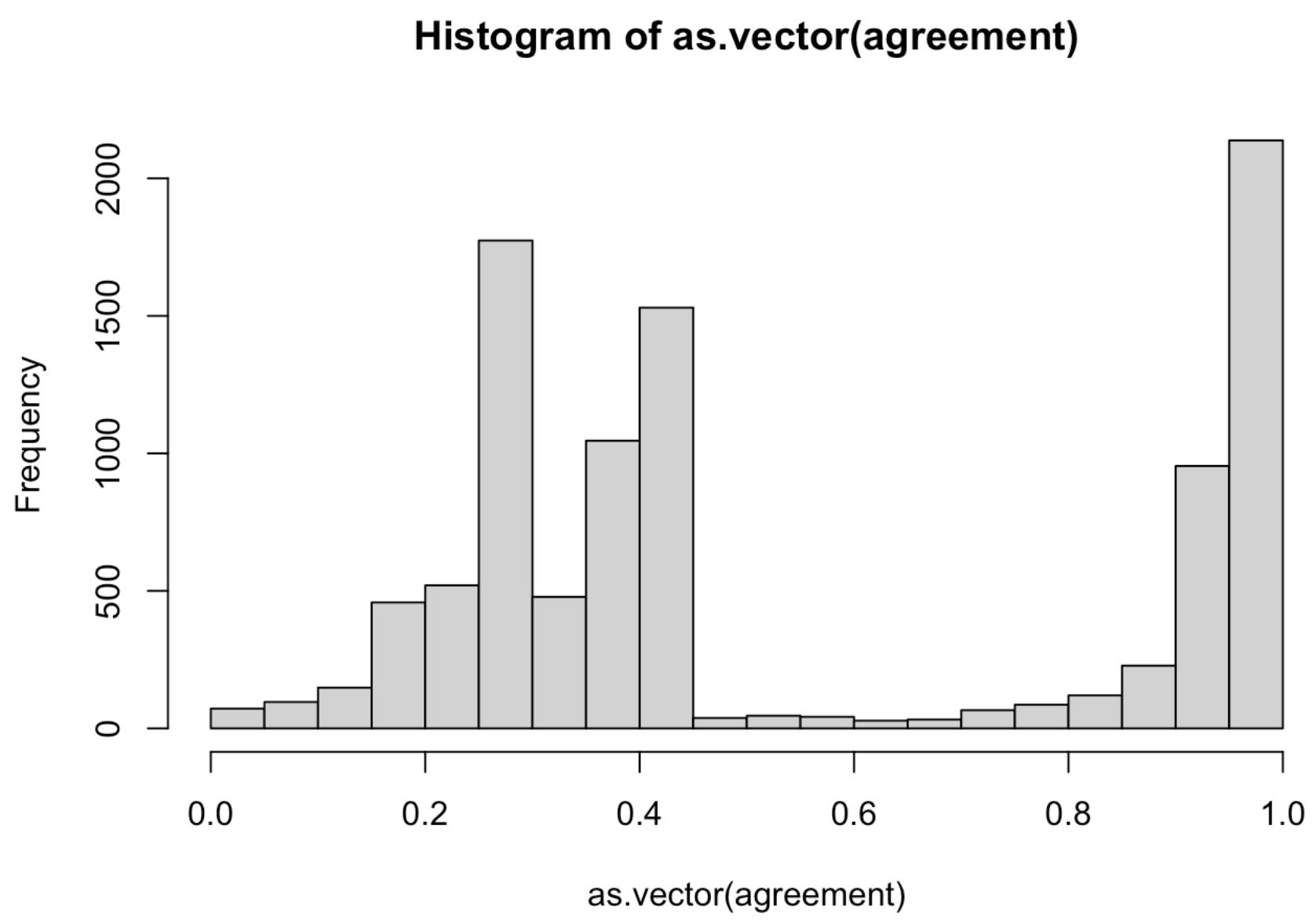

This provide similarly results with diceR package with using k-means, but slightly more diverse as both variables and observations were sampled.

Establish consensus

Here we demonstrate three main methods for obtaining consensus:

- Majority voting;
- Consensus matrix; and
- Clustering with binary encoding data

Majority voting

Majority Voting can be implemented using ‘majority_voting’ function in diceR package.

```
##'is.relabelled = TRUE' means to re-label the clustering results
dta_group$majority_voting_km <- diceR::majority_voting(BCs_diceR_km, is.relabelled = FALSE)

# Comparing with true label
table(dta_group$group,dta_group$majority_voting_km)
```

```
##
##            1   2   3
##   Group1   1  21 276
##   Group2   0 150   0
##   Group3 148   0   0
```

A fraction of observations in the large cluster were miss-classified, which may due to the slightly higher level of disagreement between BCs. See example below showing slightly better results with BCs generated using GMM.

```
##'is.relabelled = TRUE' means to re-label the clustering results
dta_group$majority_voting_gmm <- diceR::majority_voting(BCs_diceR_gmm, is.relabelled = FALSE)

a<-as_tibble(BCs_diceR_gmm)
# Comparing with true label
table(dta_group$group,dta_group$majority_voting_gmm)
```

```
##
##            1   2   3
##   Group1   0   0 298
##   Group2   0 150   0
##   Group3 148   0   0
```

Consensus matrix

Consensus matrix can be easily constructed from BCs using consensus_matrix function from diceR package.

```
# Extract consensus matrix
cm <- diceR::consensus_matrix(BCs_diceR_km)

# Visualise using heatmap
plot<- diceR::graph_heatmap(BCs_diceR_km)
```

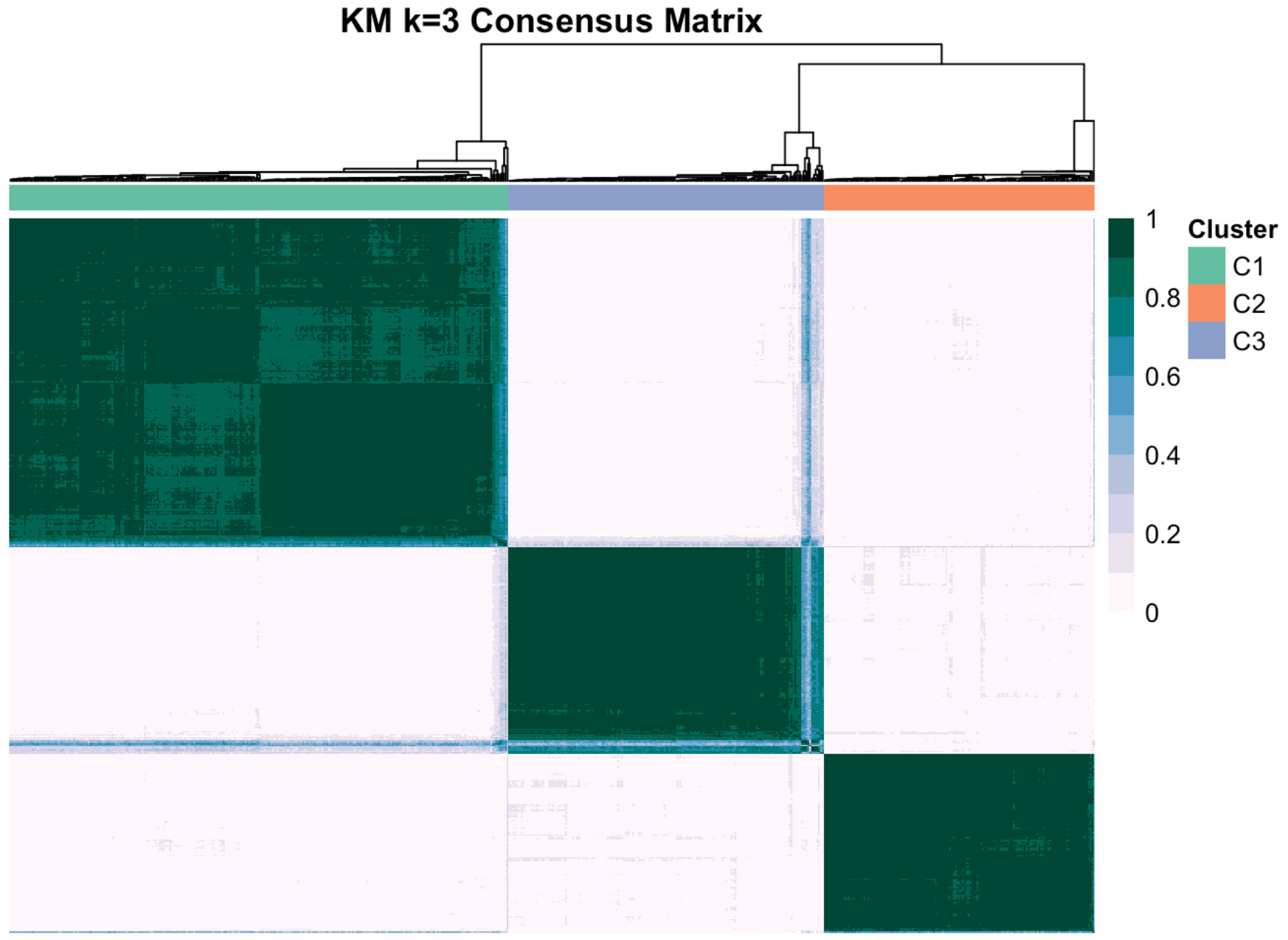


```
# Extract using hierarchical clustering which is saved in the plot
dta_group$cm_hierarchical<-plot[[2]][[1]][[1]]

# Comparing with true label
table(dta_group$group,dta_group$cm_hierarchical)
```

```
##
##           C1  C2  C3
##   Group1 274   1  23
##   Group2   0   0 150
##   Group3   0 148   0
```

The consensus matrix can be used in other clustering models, e.g., spectral clustering.

```
# Use spectralClustering function from RclusTool package.
res <- RclusTool::spectralClustering(cm, K=3)
dta_group$spectral<-res$label
table(dta_group$group,dta_group$spectral)
```

```
##
##          1   2   3
##   Group1   1 275  22
##   Group2   0   0 150
##   Group3 148   0   0
```

LCE from diceR can also be used. In diceR package, LCE applied hierarchical clustering instead of the original graph models proposed by Iam-On and Garrett (2010).

```
# Use LCE function from diceR package
dta_group$spectral<- LCE(BCs_imputed,k=3,sim.mat = "cts")
table(dta_group$group,dta_group$spectral)
```

```
##
##          1   2   3
##   Group1 278   1  19
##   Group2   0   0 150
##   Group3   0 148   0
```

There were around 10 records commonly incorrectly clustered. This is due to the fact that the clusters are non-spherical and have difference variance. GMM function is slighly better suited with this data.

```
# Extract consensus matrix from GMM
cm <- diceR::consensus_matrix(BCs_diceR_gmm)
res <- RclusTool::spectralClustering(cm, K=3)
dta_group$spectral<-res$label
table(dta_group$group,dta_group$spectral)
```

```
##
##          1   2   3
##   Group1   0 298   0
##   Group2 150   0   0
##   Group3   0   0 148
```

Binary encoding clustering

Here we provide another example using binary cluster association matrix using the manually generated clustering results. The missing data can be imputed first and then converted to binary cluster association matrix using dummy variable coding.

```
# Missing data can be filled in with imputation here we use missRanger for fast imputation
set.seed(1234)
BCs_manual <- missRanger::missRanger( BCs_manual %>% mutate_all(as.factor),
                            pmm.k = 3)  # using predictive mean matching

## Extract binary encoding matrix
Bi_BCs <- fastDummies::dummy_cols(BCs_manual,
           select_columns = names(BCs_manual),
           ignore_na = TRUE, # ignoring NA values in BCs
           remove_selected_columns = TRUE  # removing the original data columns
           )
# Bi_BCs is a 497 x 300 matrix
```

Then k-means can be applied to the binary cluster association matrix.

```
##  Clustering using k-means
set.seed(1234)
clustering_results <- kmeans(Bi_BCs, 3,nstart = 20)

## Add binary encoding clustering results in 'dta' frame
dta_group$Bin_pam <- clustering_results$cluster
table(dta_group$group,dta_group$Bin_pam)
```

```
##
##          1   2   3
##   Group1  15 283   0
##   Group2 150   0   0
##   Group3   0   0 148
```

Integrated functions

Existing functions can integrate these individual steps into a combined function. Here we first use the dice function in diceR package.

```
CC_diceR <- diceR::dice(dta_pca %>% select(-ID),
                        nk = 2:5, # 2-5 clusters
                        reps = 100, # 100 BCs
                        p.item=0.8, # % of subsamples
                        algorithms = c("km", "gmm", "hc"), # three algorithms for BCs
                        hc.method = "ward.D2", # use ward linkage method for hierarchical Clustering
                        cons.funs = c("LCE"), # one consensus function
                        k.method="all", # keep all consensus results for different k
                        plot=TRUE, # plot consensus matrix,
                        seed= 1234 # set random seed
                        )
```

```
## Selecting k and imputing non-clustered cases
## Computing consensus functions
## Evaluating output with consensus function results
```

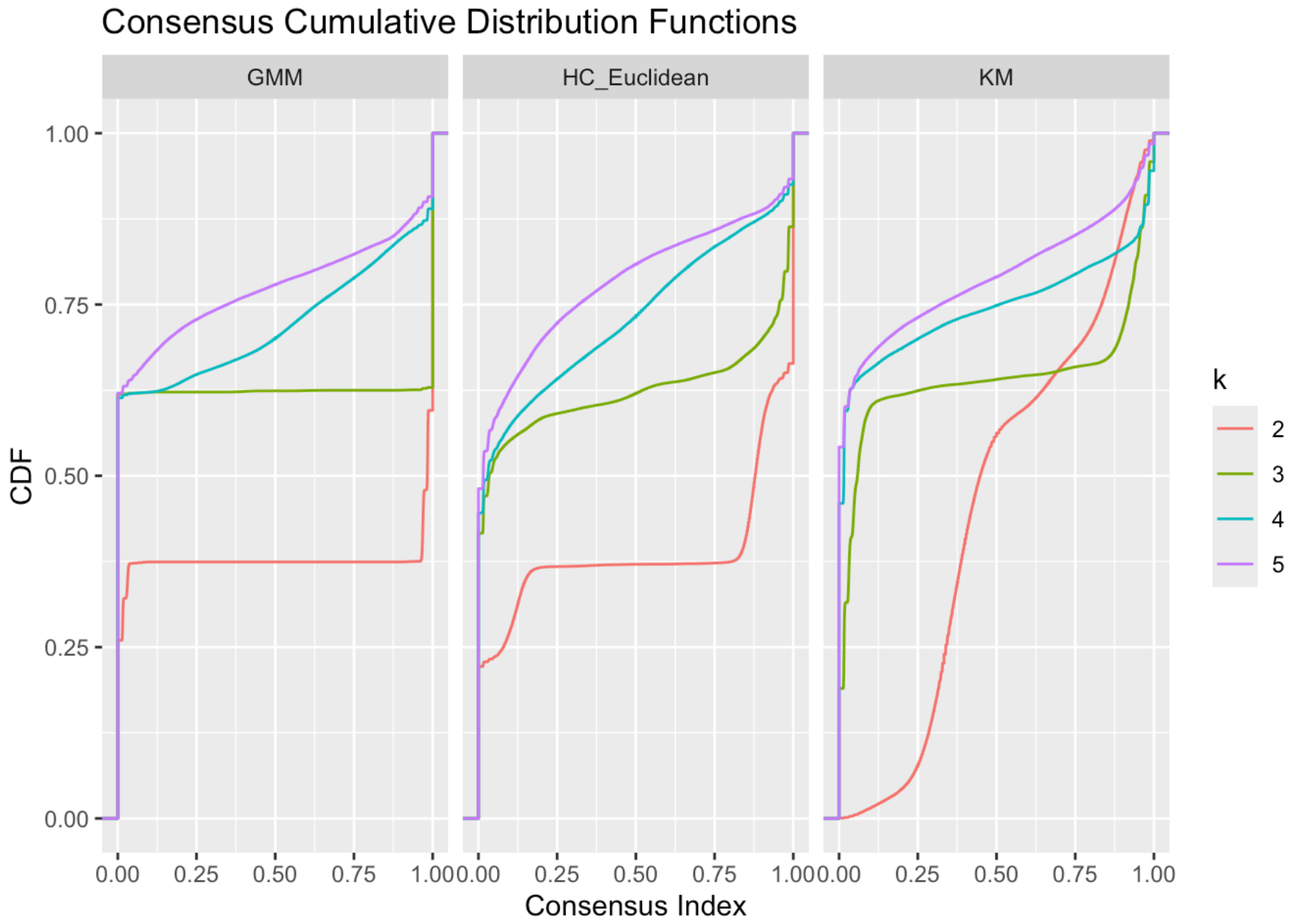
Consensus Cumulative Distribution Functions
GMM
HC_Euclidean
KM
CDF
1.00
0.75
0.50
0.25
0.00
0.00 0.25 0.50 0.75 1.00
Consensus Index
k
2
3
4
5

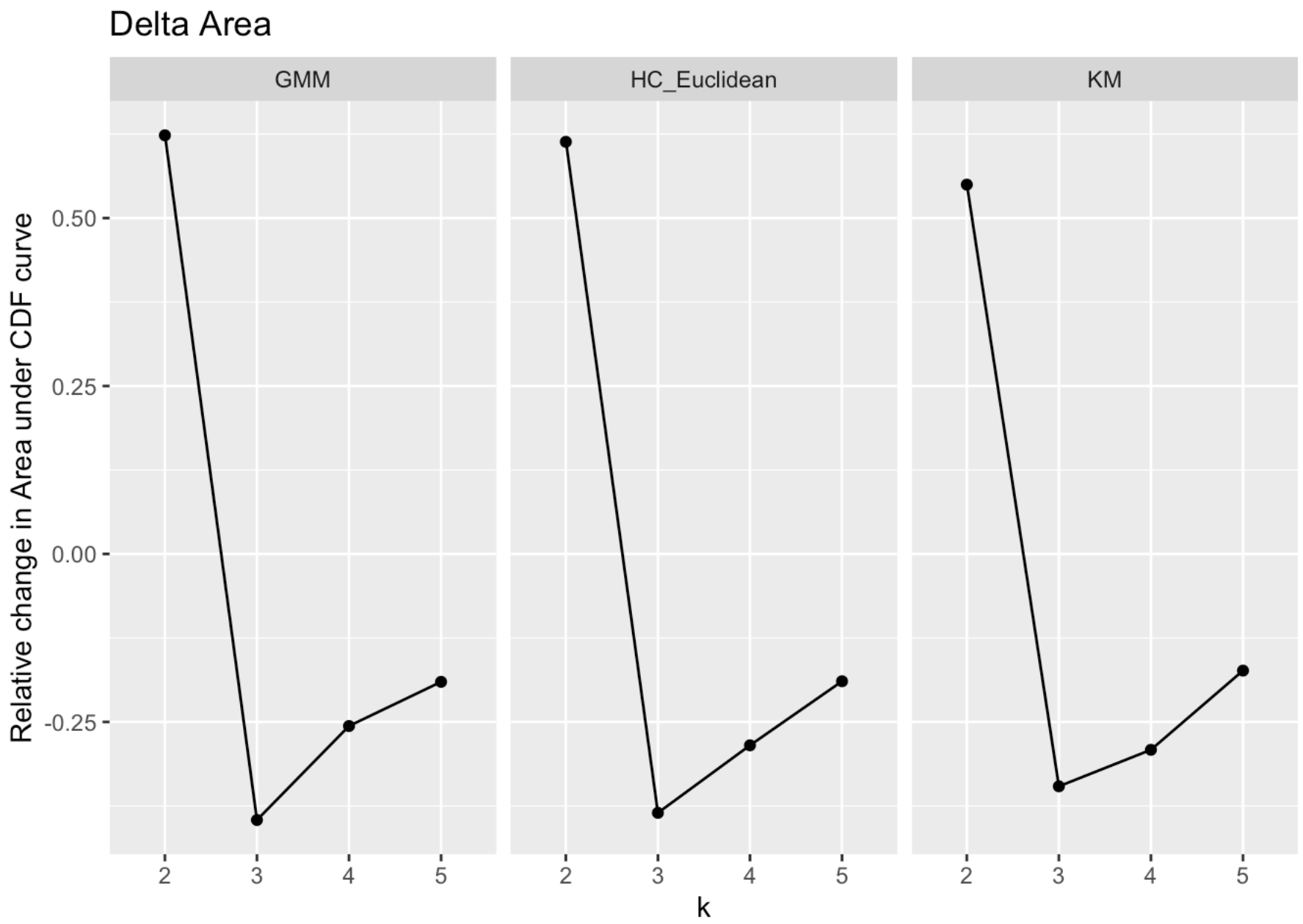
Delta Area
GMM
HC_Euclidean
KM
Relative change in Area under CDF curve
0.50
0.25
0.00
-0.25
2 3 4 5
k

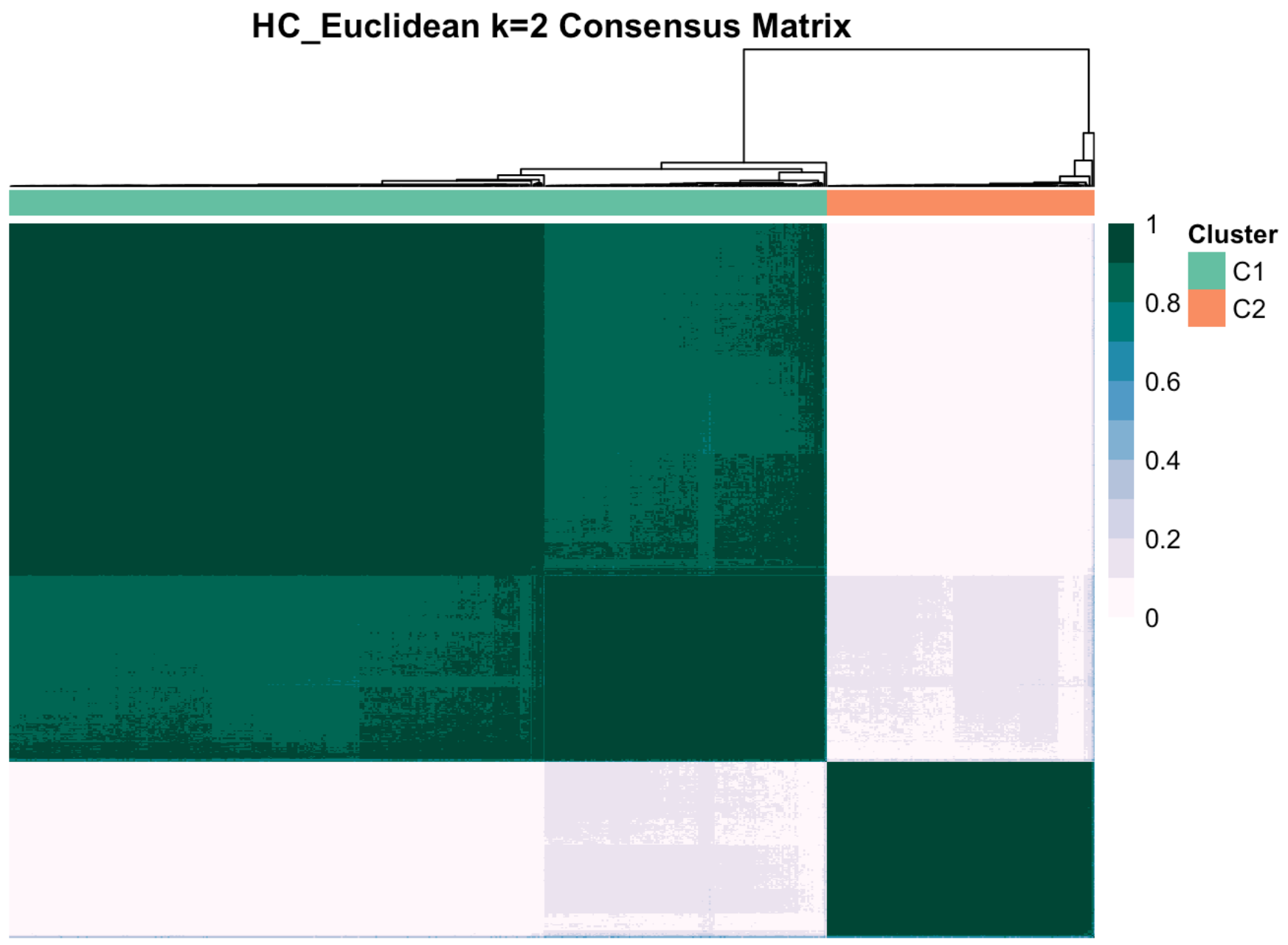
HC_Euclidean k=2 Consensus Matrix
1
0.8
0.6
0.4
0.2
0
Cluster
C1
C2

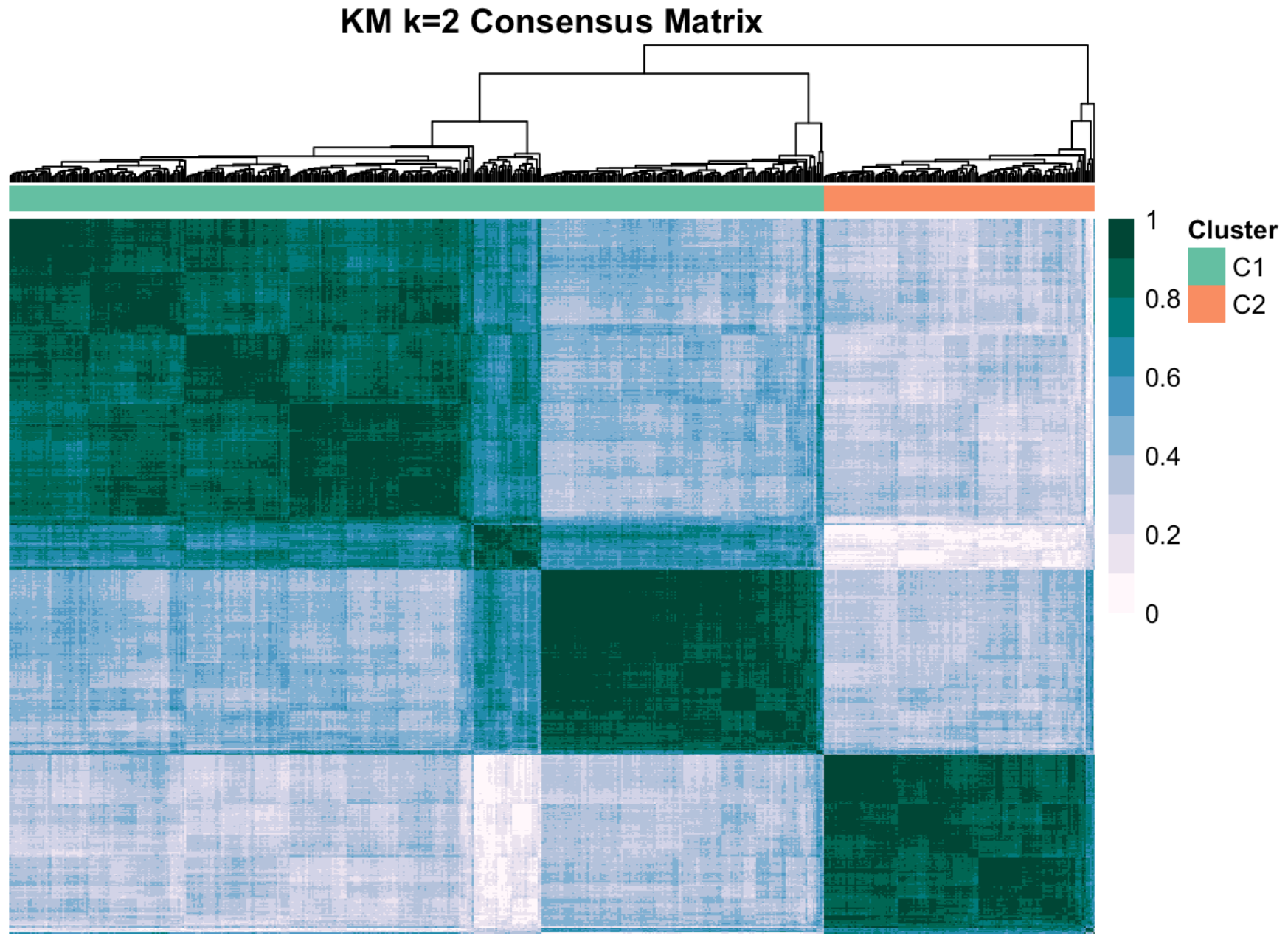
KM k=2 Consensus Matrix
Cluster
C1
C2
1
0.8
0.6
0.4
0.2
0

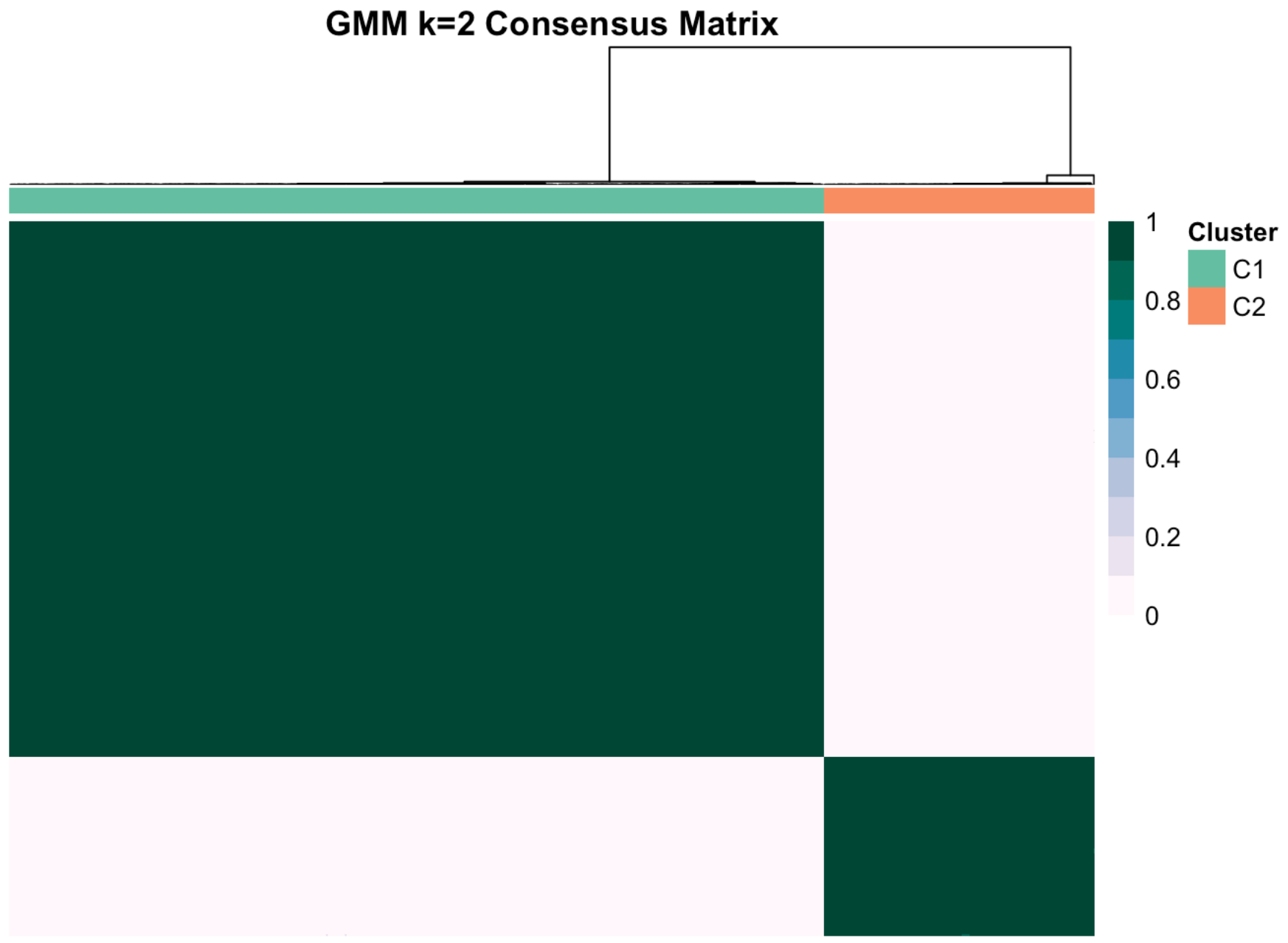
GMM k=2 Consensus Matrix
Cluster
C1
C2
1
0.8
0.6
0.4
0.2
0

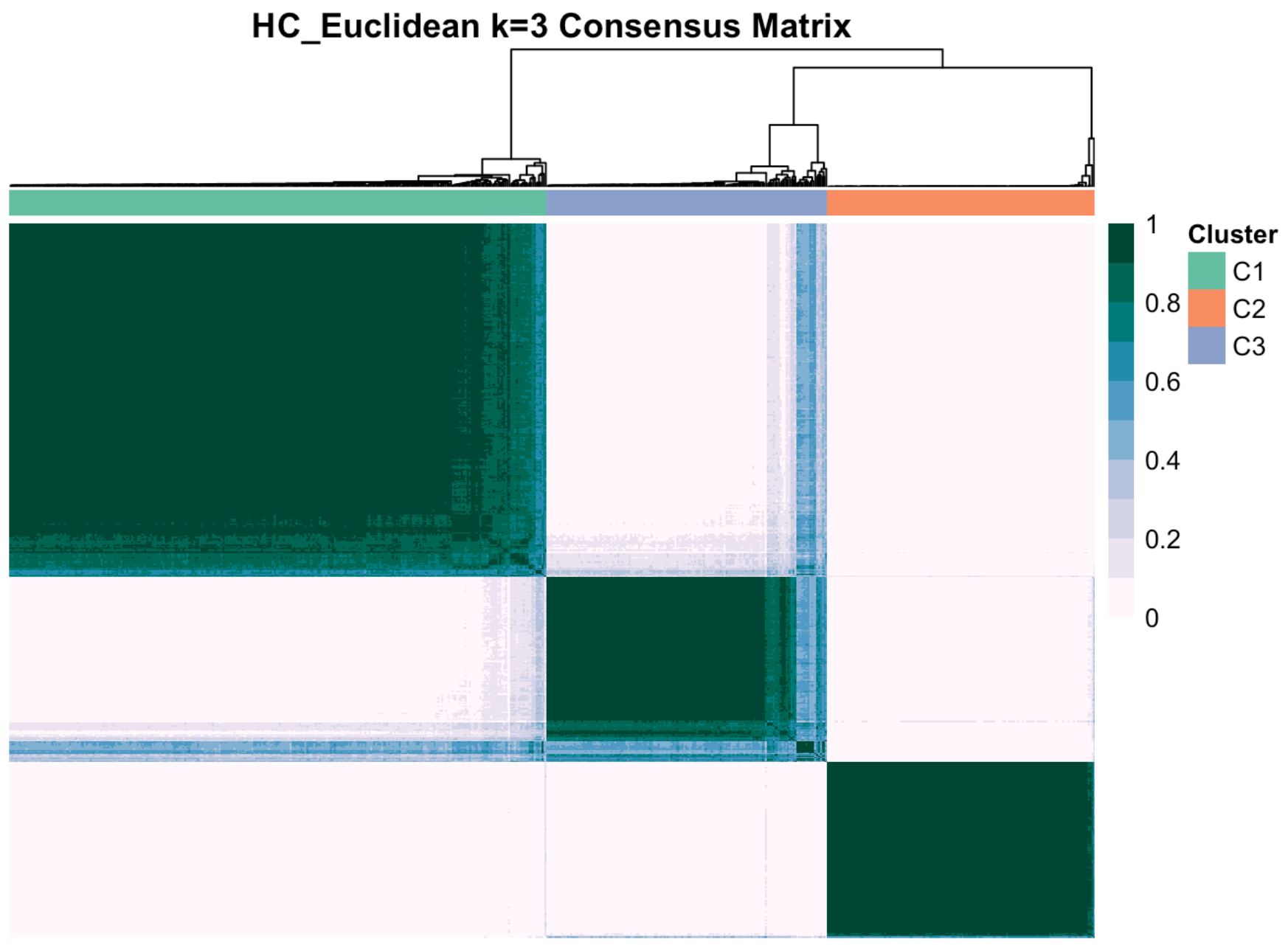
HC_Euclidean k=3 Consensus Matrix
Cluster
C1
C2
C3
1
0.8
0.6
0.4
0.2
0

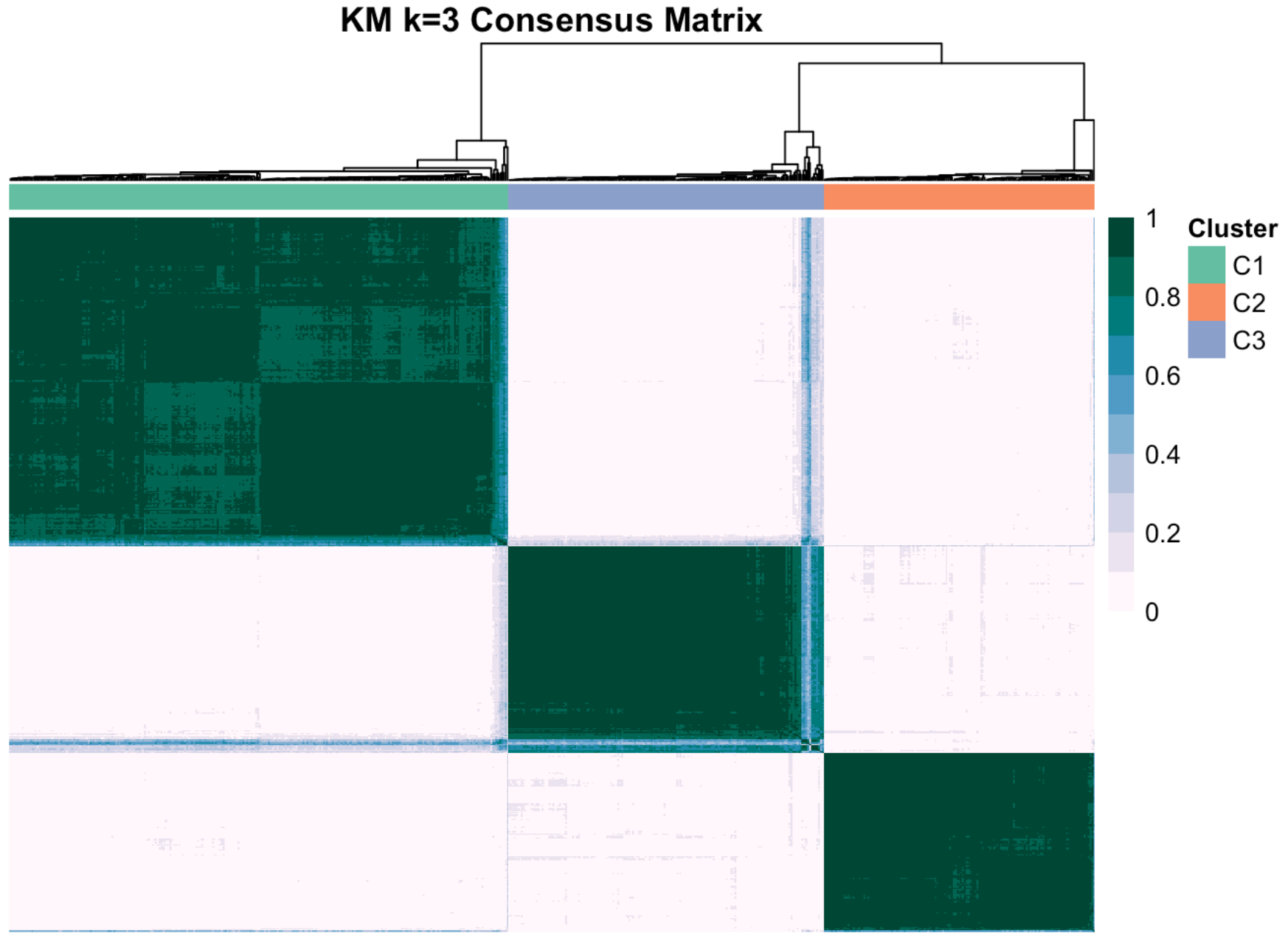
KM k=3 Consensus Matrix
1
0.8
0.6
0.4
0.2
0
Cluster
C1
C2
C3

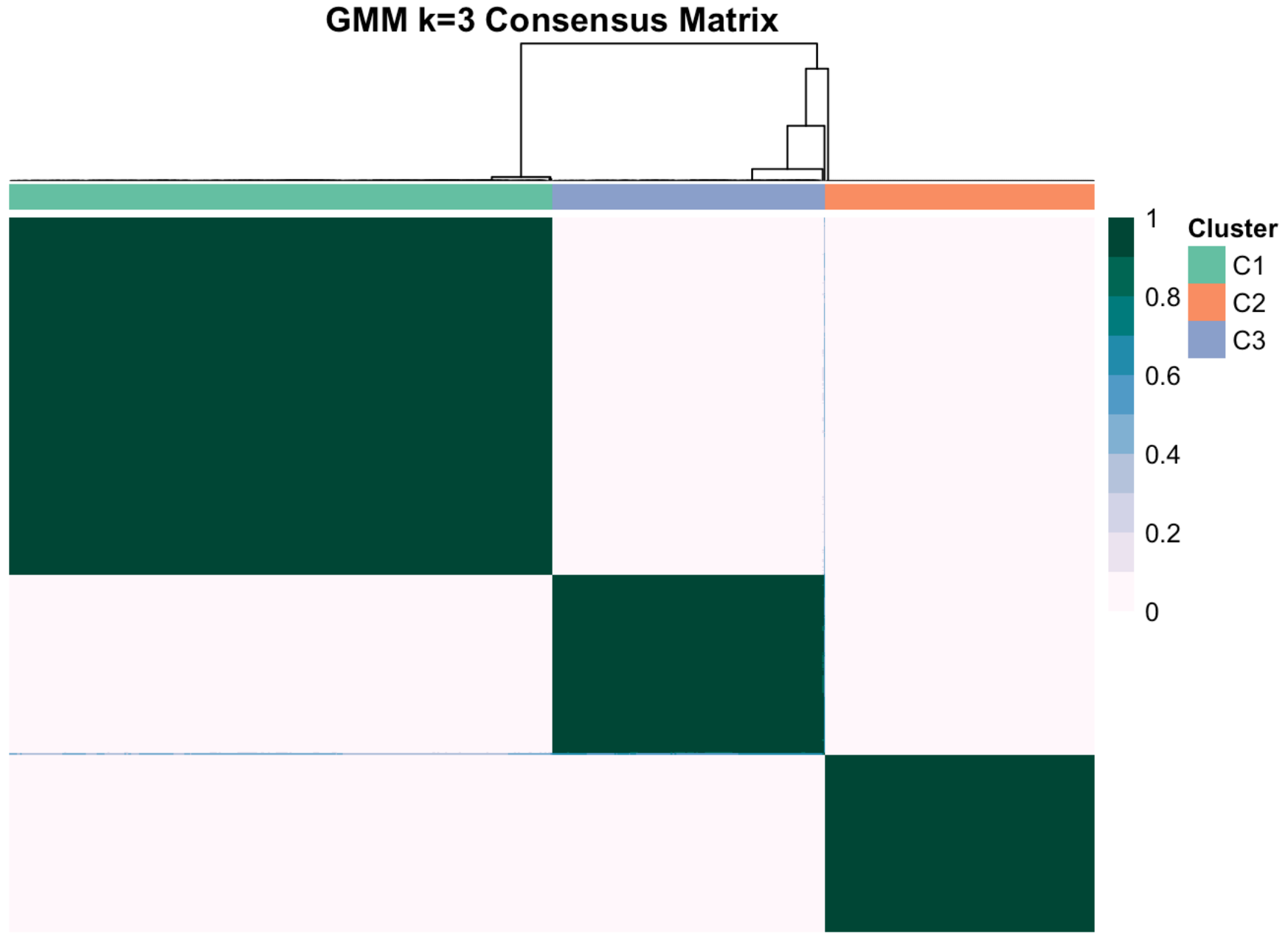
GMM k=3 Consensus Matrix
1
0.8
0.6
0.4
0.2
0
Cluster
C1
C2
C3

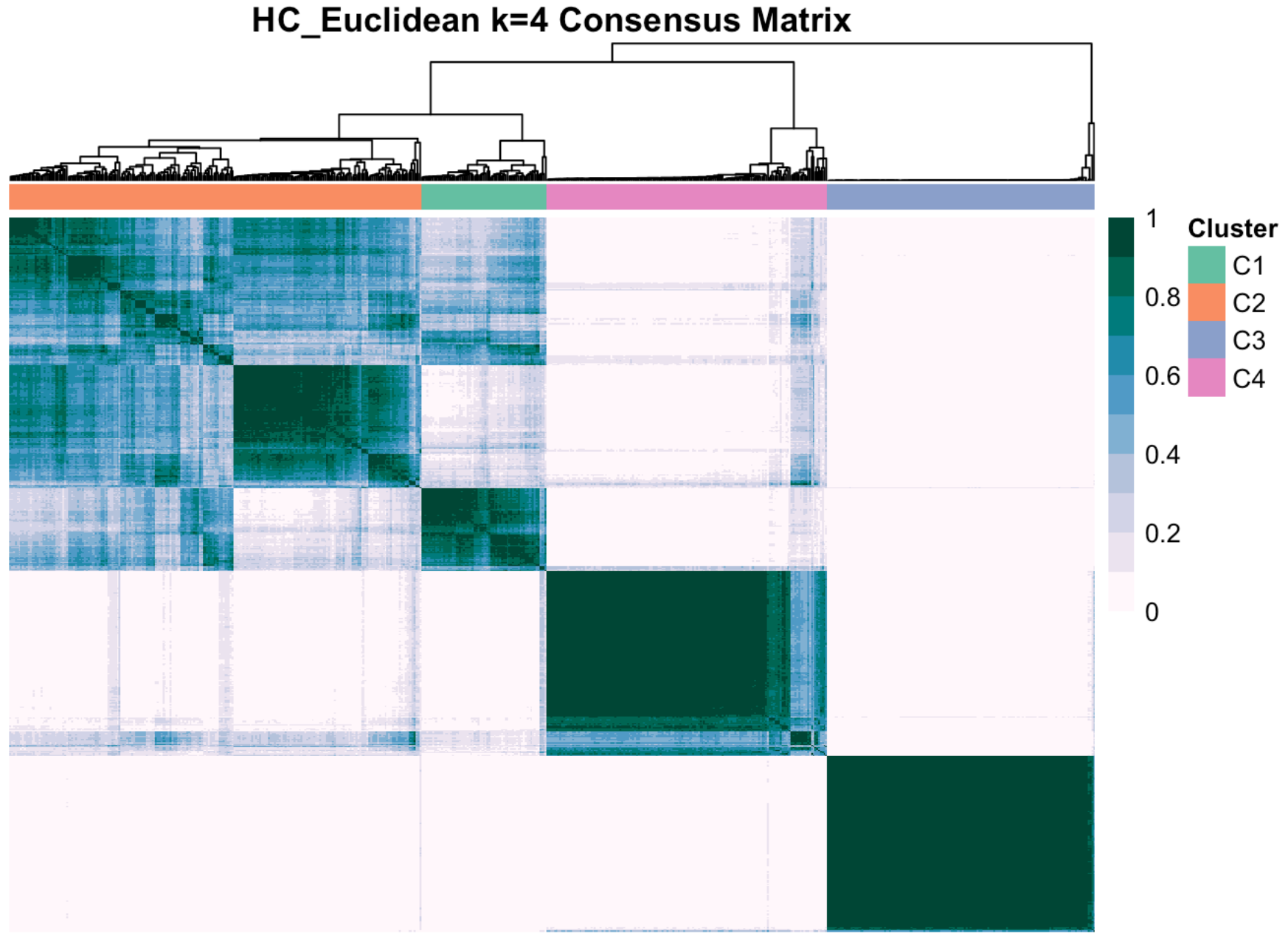
HC_Euclidean k=4 Consensus Matrix
1
0.8
0.6
0.4
0.2
0
Cluster
C1
C2
C3
C4

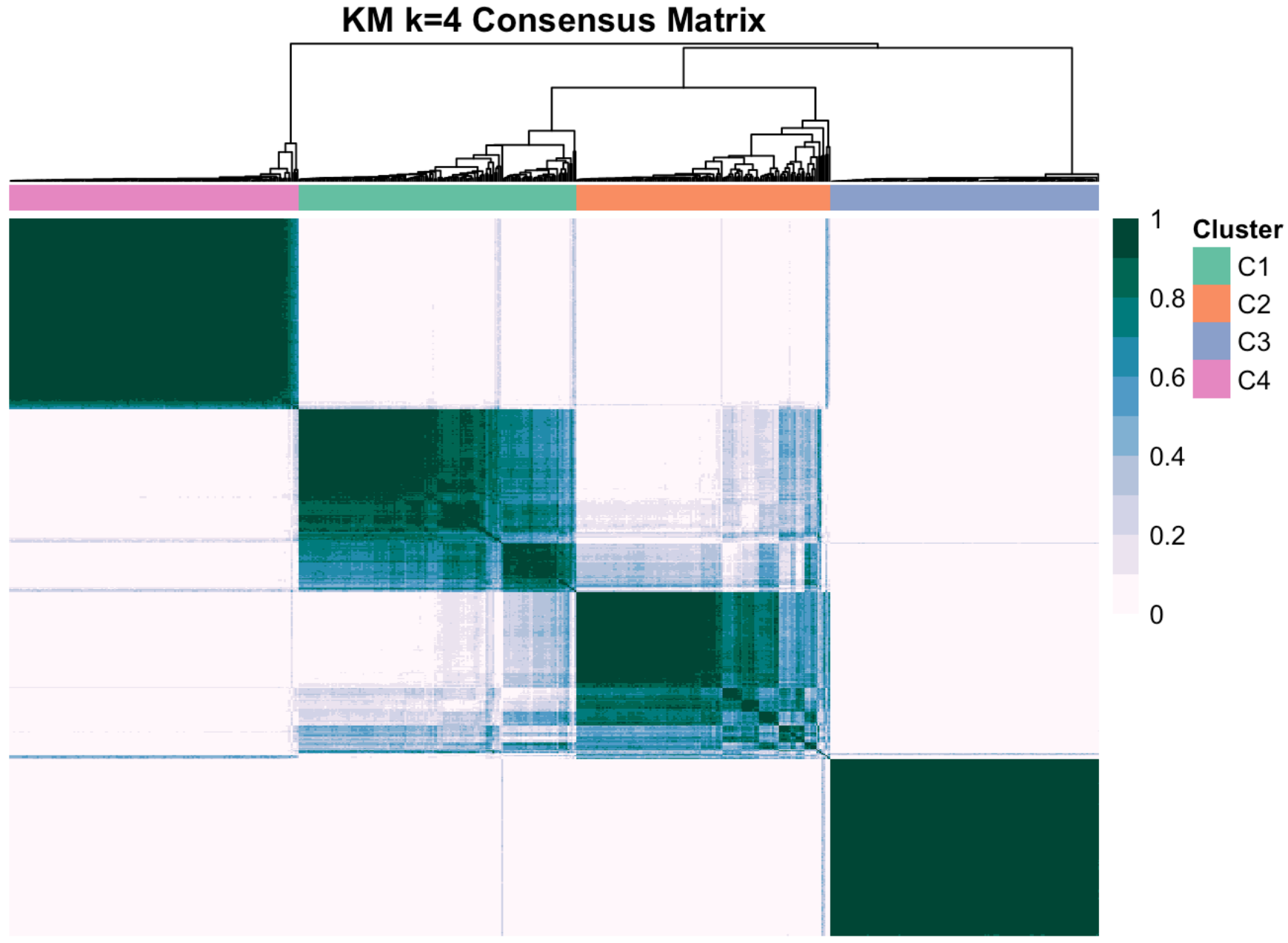
KM k=4 Consensus Matrix
1
0.8
0.6
0.4
0.2
0
Cluster
C1
C2
C3
C4

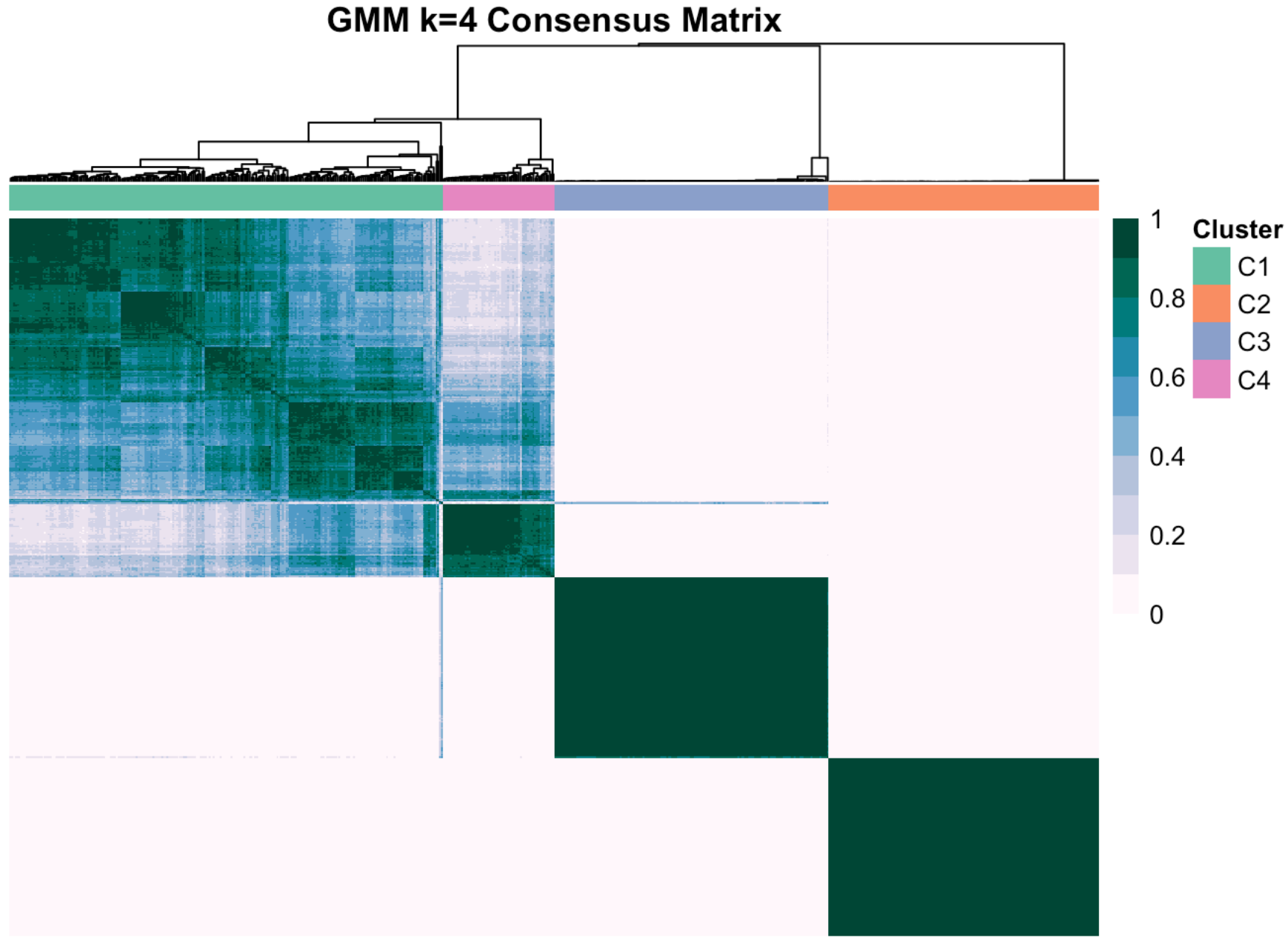
GMM k=4 Consensus Matrix
1
0.8
0.6
0.4
0.2
0
Cluster
C1
C2
C3
C4

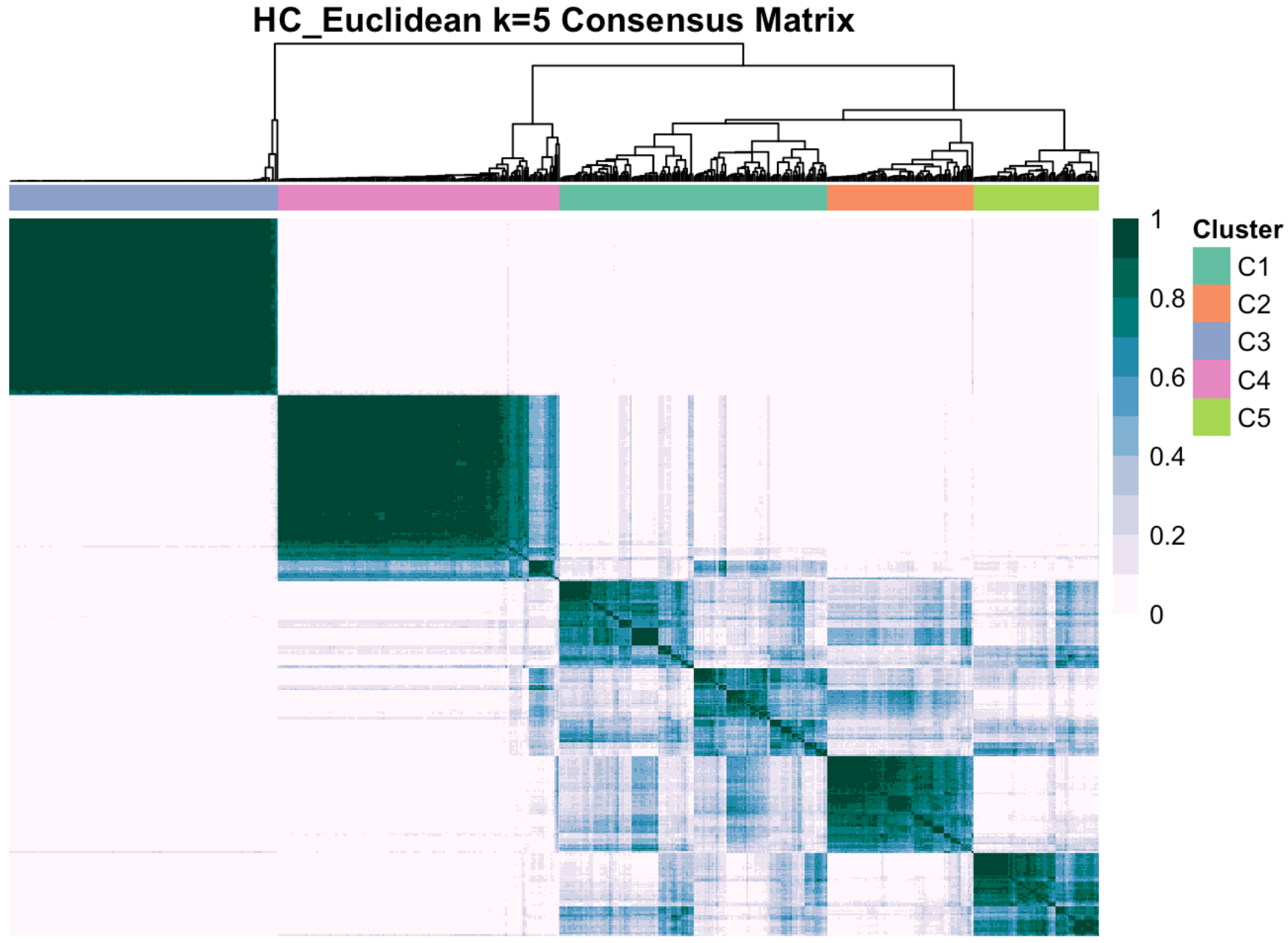
HC_Euclidean k=5 Consensus Matrix
1
0.8
0.6
0.4
0.2
0
Cluster
C1
C2
C3
C4
C5

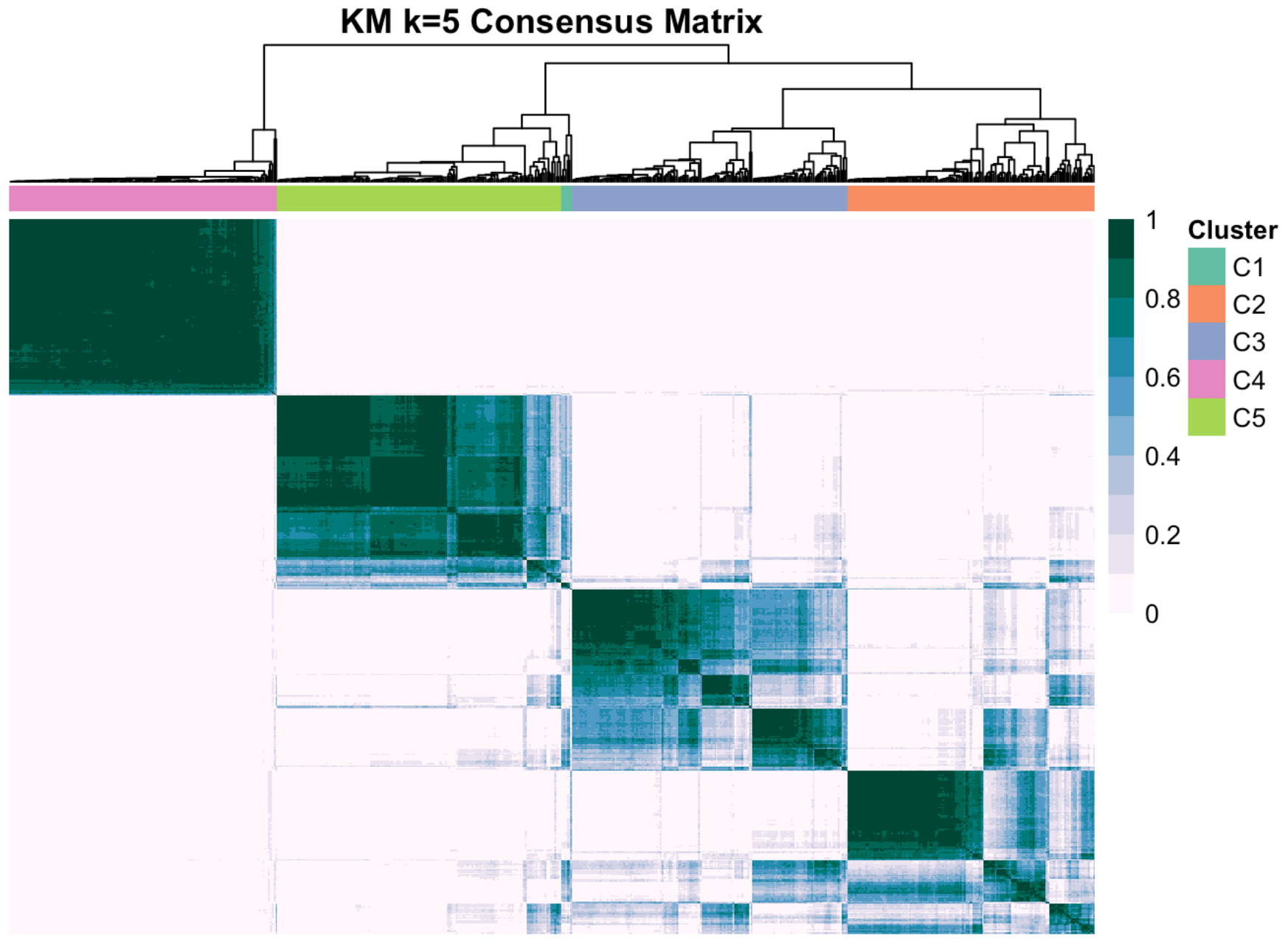

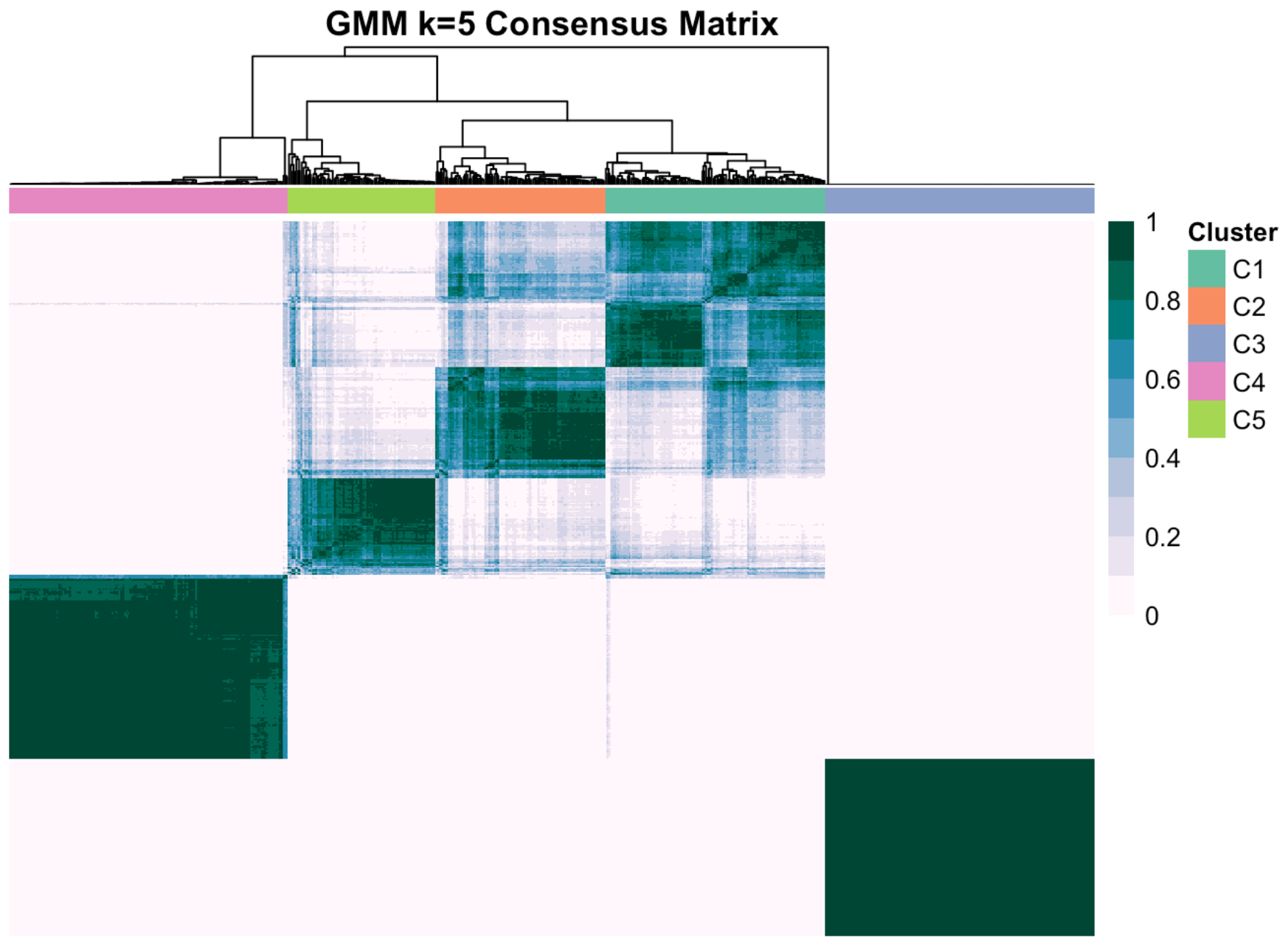

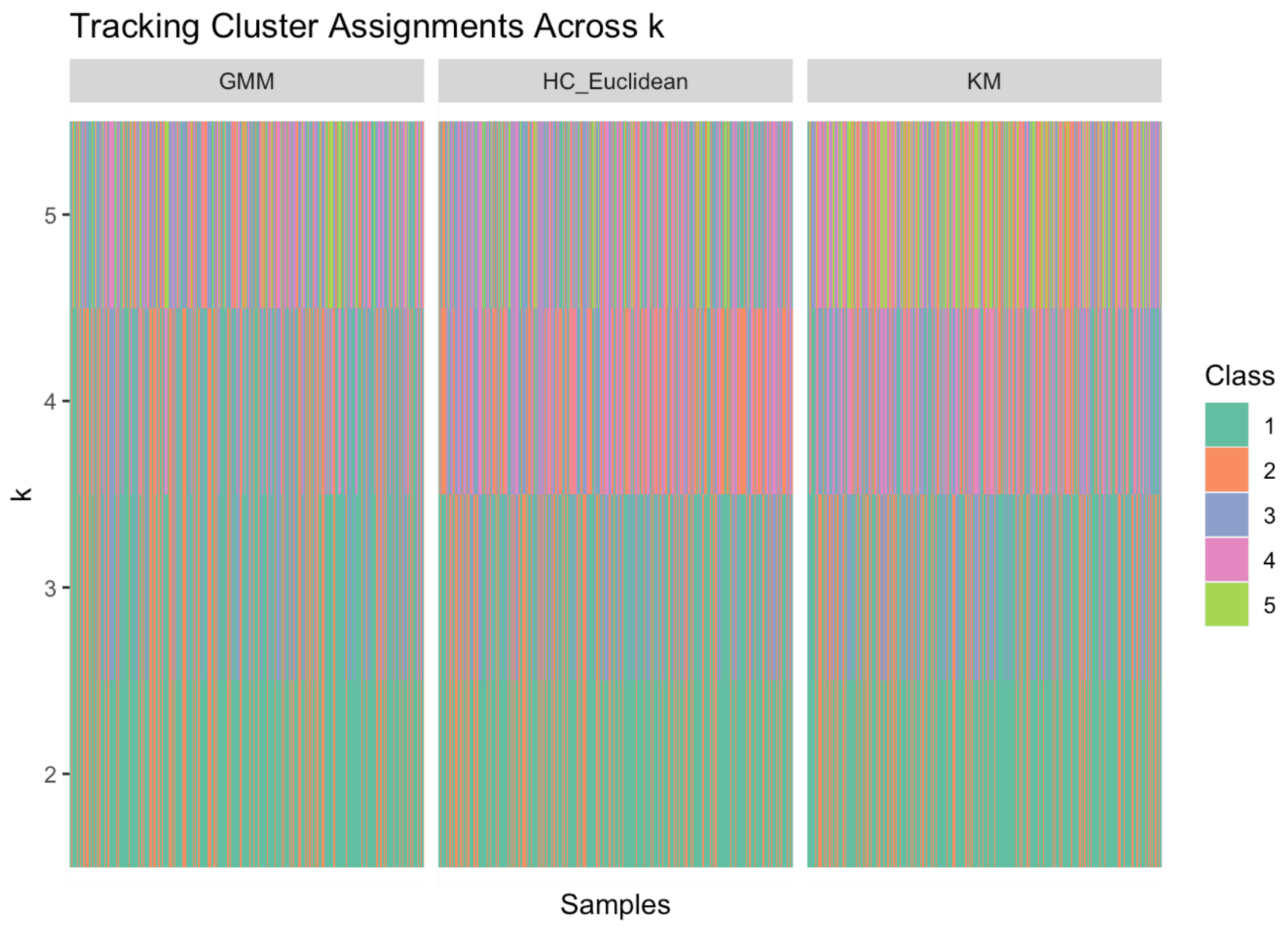


```
## Did not converge after  1000  iterations.
```

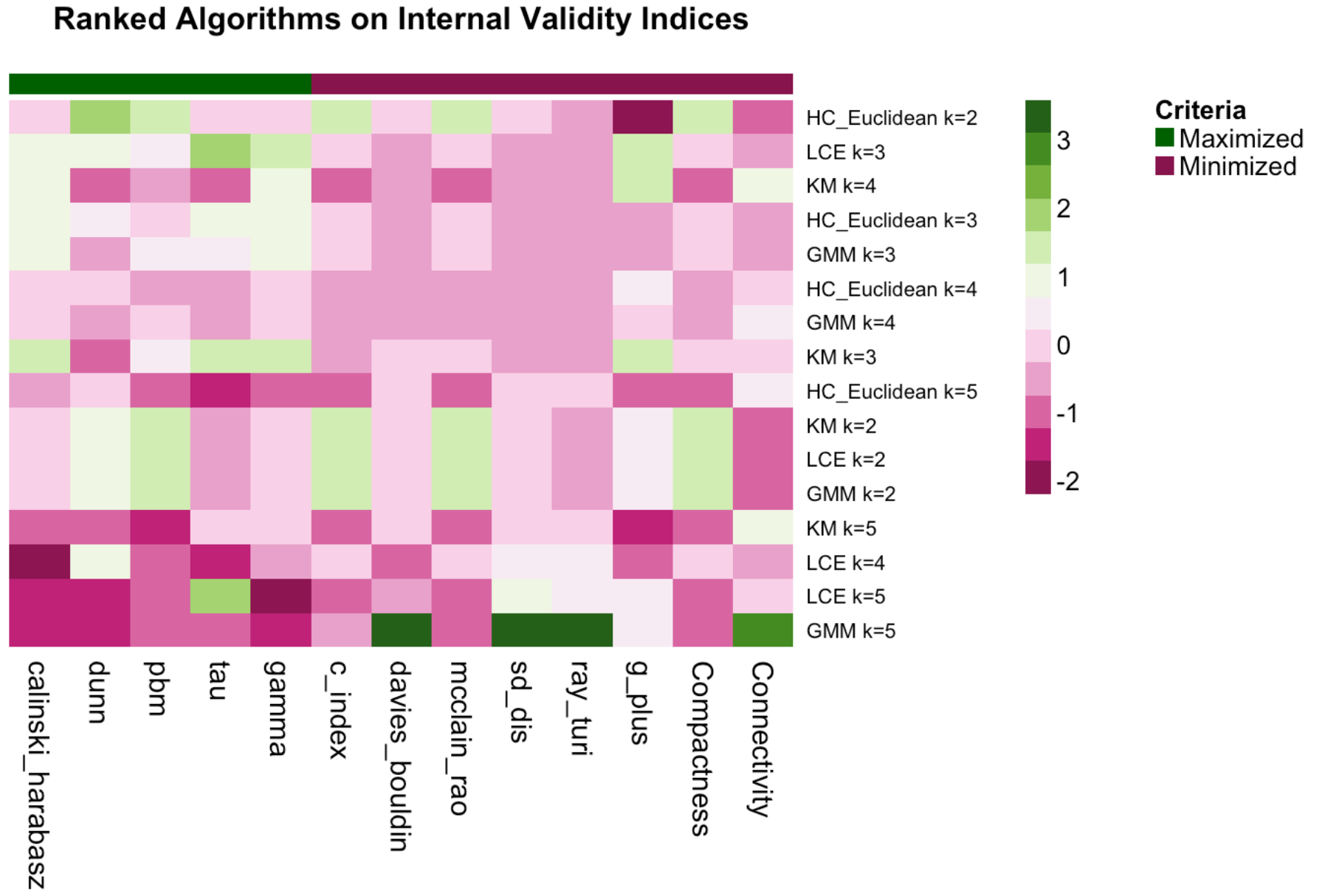

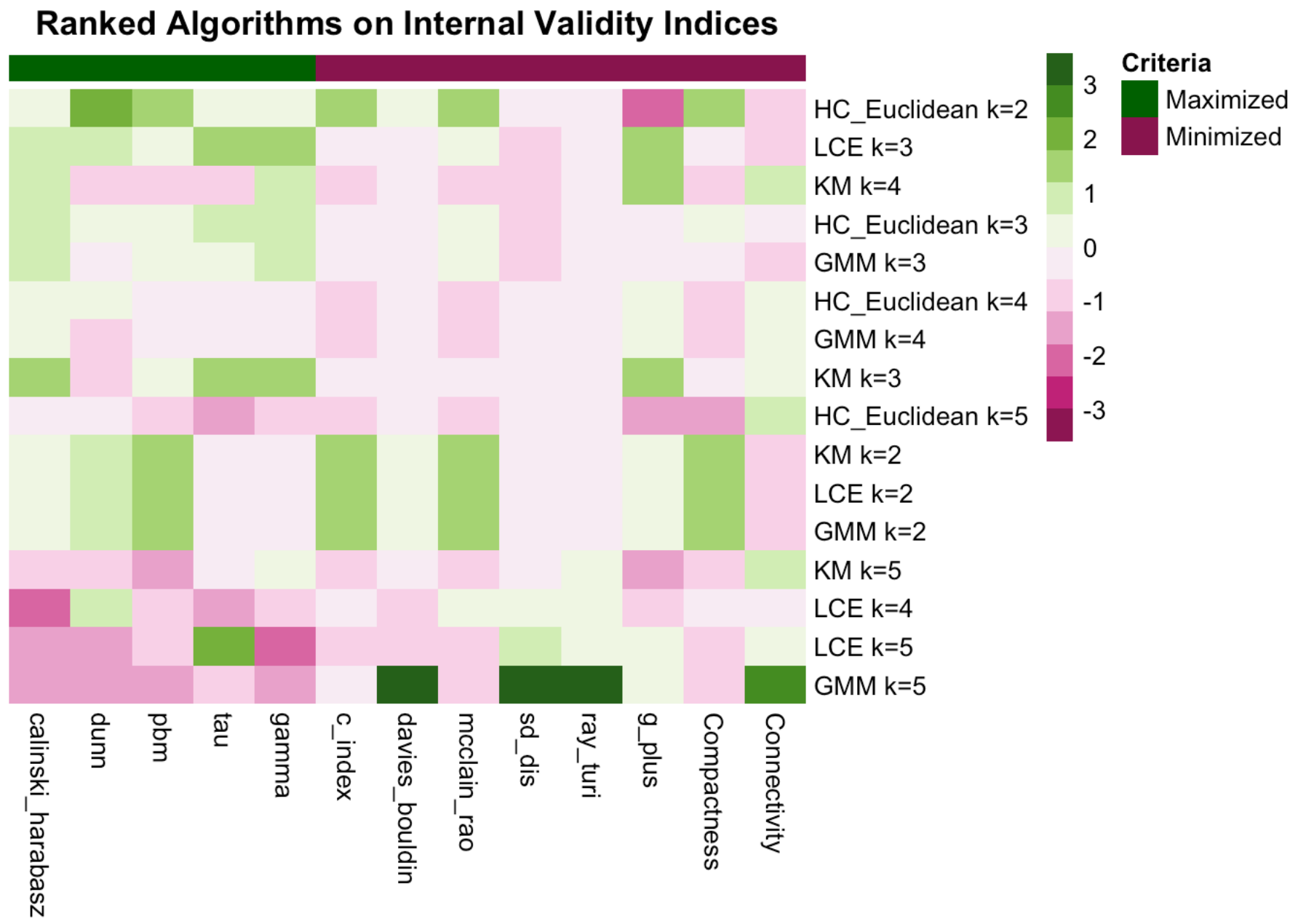


```
## Diverse Cluster Ensemble Completed
```

The cdf plot and delta area plot can be extracted for publication quality figure.

```
#sel-define lables and colours
labels<- c(  `GMM` = "GMM",
             `HC_Euclidean` = "Hierarchical clustering",
             `KM` = "K-means"   )
colfunc <- colorRampPalette(c("#E2E2E2", "#419AB0"))
colours<-colfunc(4)

# CDF
p1 <- diceR::graph_cdf(CC_diceR$E) +
  theme_few() +
  theme(legend.position = "bottom") +
  facet_grid( ~ Method,labeller = as_labeller(labels)) +
  scale_colour_manual(values=colours)
```

```
# CDF
p2 <- diceR::graph_delta_area(CC_diceR$E) +
  theme_few() +
  facet_grid( ~ Method,labeller = as_labeller(labels))
```

```
p1 + p2 +
  plot_layout(ncol = 1) +
  plot_annotation(tag_levels = 'A')
```

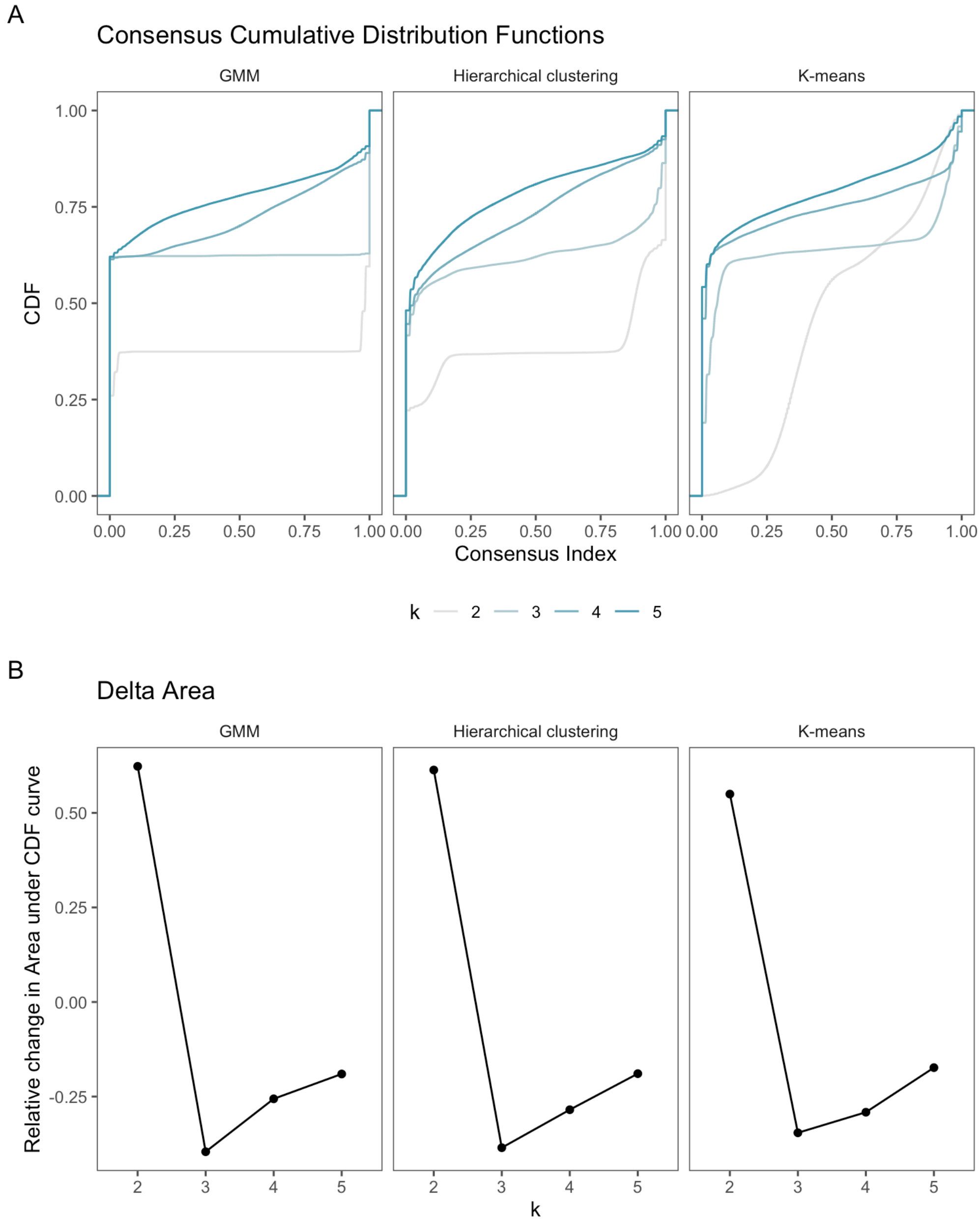

Users can also use CSPA function, which performs hierarchical clustering utilizing the co-association matrix. CSPA retains the original name as proposed by Strehl & Ghosh (2002). However, it diverges from the original methodology by replacing the graph-based partitioning model METIS with hierarchical clustering. This makes the consensus function consistent with model introduced by Monti (2003).

Check results

```
head(CC_diceR$clusters)
```

```
##   LCE k=2 LCE k=3 LCE k=4 LCE k=5
## 1       1       1       1       1
## 2       1       1       1       1
## 3       1       1       1       1
## 4       2       2       2       2
## 5       1       3       3       3
## 6       1       1       1       1
```

```
# Compare with ture label
table(dta_group$group,CC_diceR$clusters[,2])
```

```
##         
##            1   2   3
##   Group1 297   0   1
##   Group2   0   0 150
##   Group3   0 148   0
```

We employed three BC algorithms and one consensus functions in our analysis. In this data example, different consensus functions generally yield comparable results. However, utilizing a variety of BC generation methods enhanced clustering accuracy compared to using just a single type. Users also have the option to refine the analysis by trimming and re-weighting individual BCs, which can be done by setting “trim=TRUE” and “reweigh=TRUE”.

Similarly this process can be achieved using consensus ConsensusClusterPlus function from ConsensusClusterPlus package. It allows BCs to generated with: * ‘hc’ hierarchical (hclust); * ‘pam’ for partitioning around medoids: * ‘km’ for k-means upon data matrix; * or other manually specified function that returns a clustering.

```
## Transpose the data 'dta' to meet the data requirements for 'ConsensusClusterPlus' clustering
dta_tran<- t(dta_pca %>% select(-ID))
set.seed(1234)
## Consensus clustering function
CC_CCP<-ConsensusClusterPlus(dta_tran,
                             maxK=5,  # maximum cluster number to evaluate
                             reps=100, # number of BCs
                             pItem=0.8, # % of subsamples
                             pFeature=1, # % of variables
                             clusterAlg="pam", # BC algorithm
                             distance="euclidean", # distance measures
                             finalLinkage= "ward.D2",  # linkage method for consensus matrix
                             seed=1234 # random seed
                             )
```

```
## end fraction
```

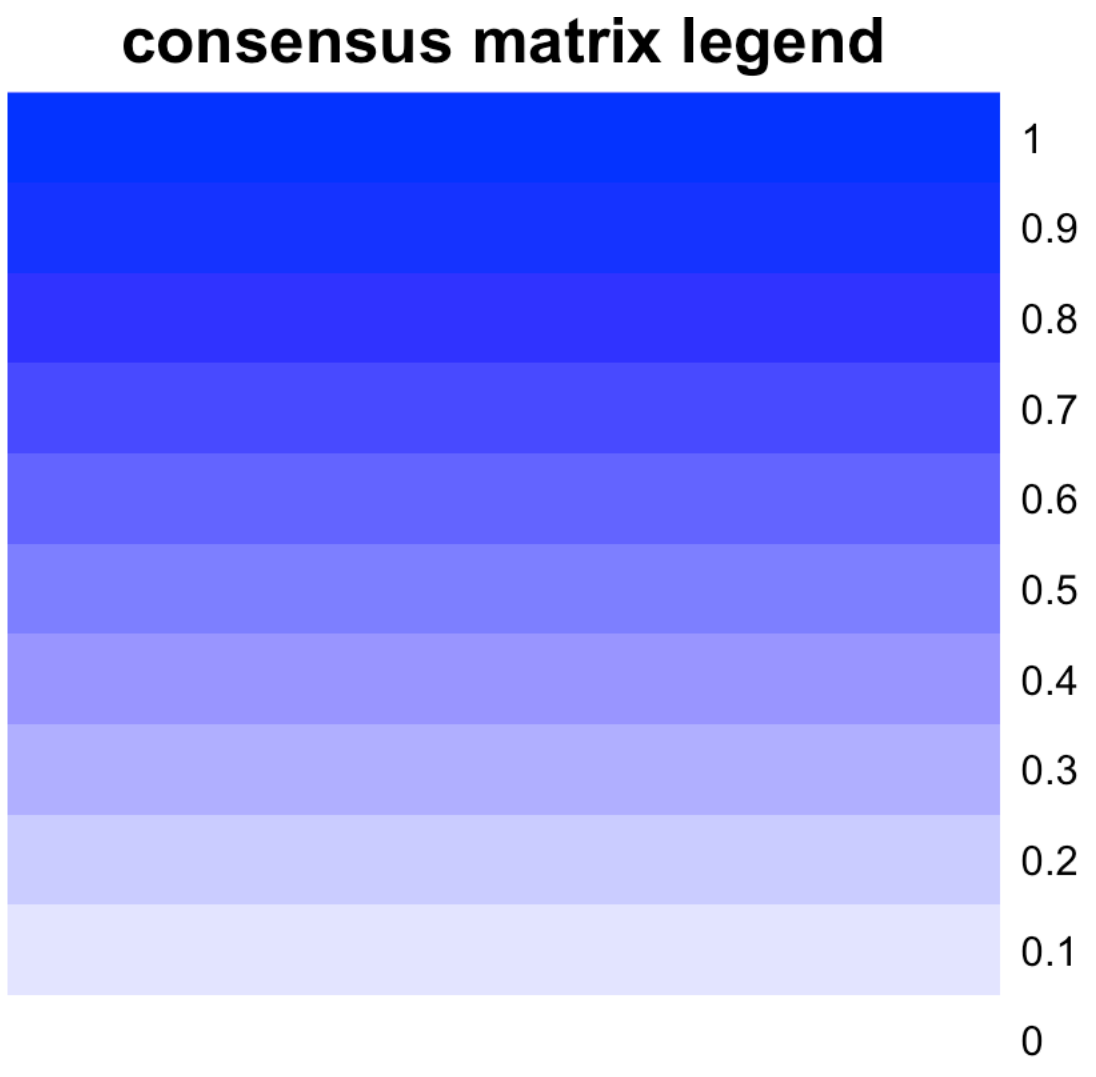


```
## clustered
```

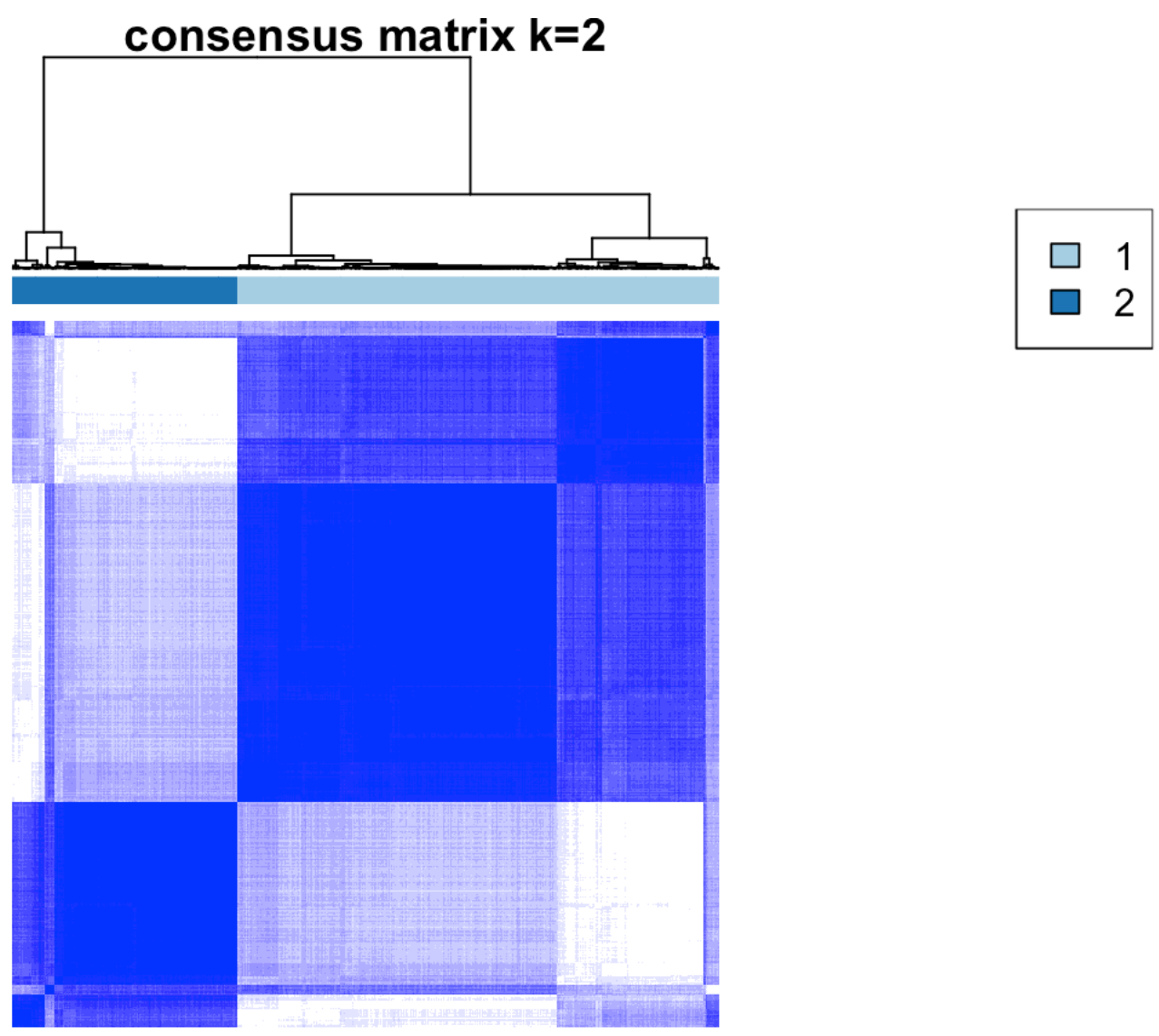


```
## clustered
```

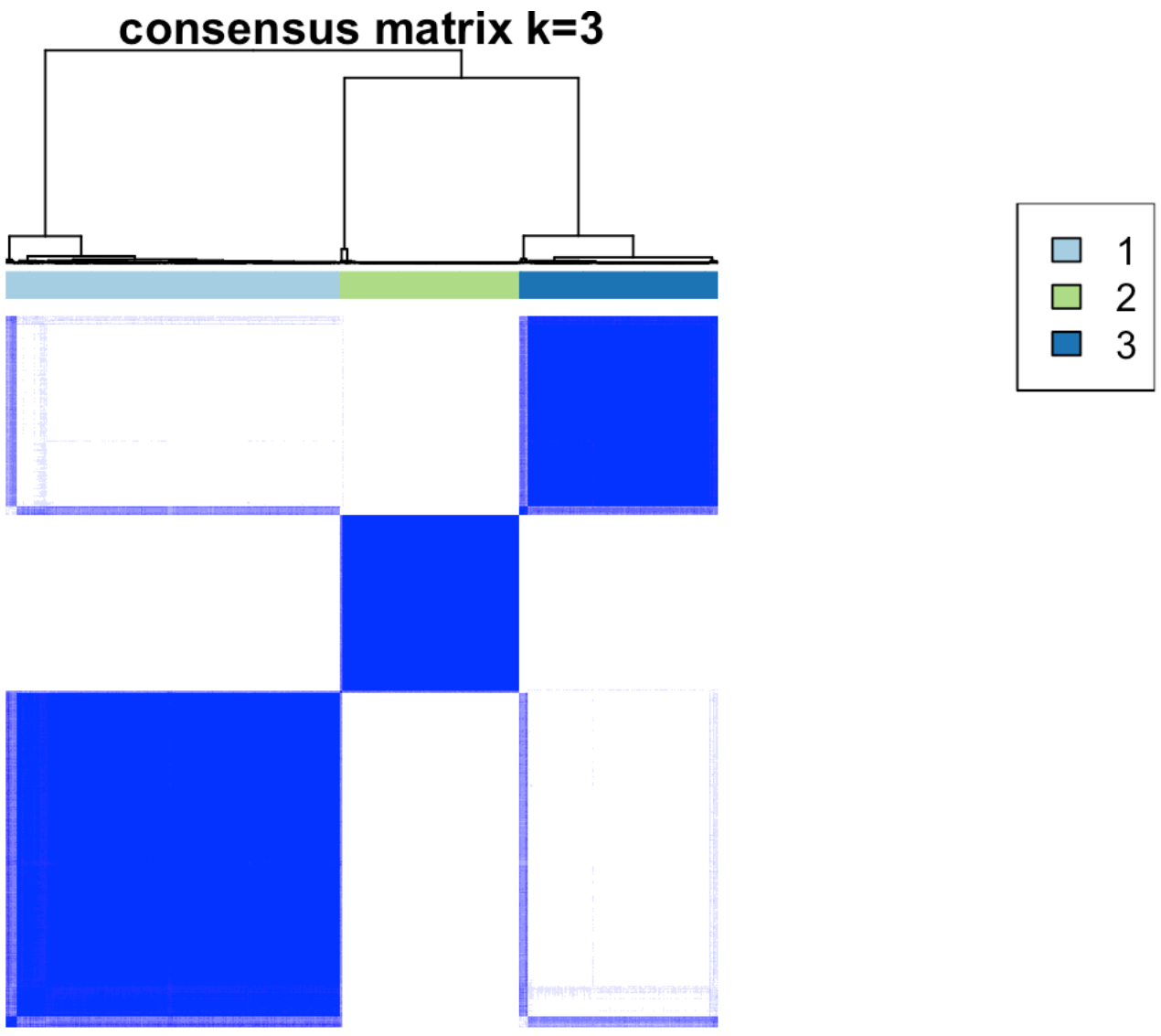


```
## clustered
```

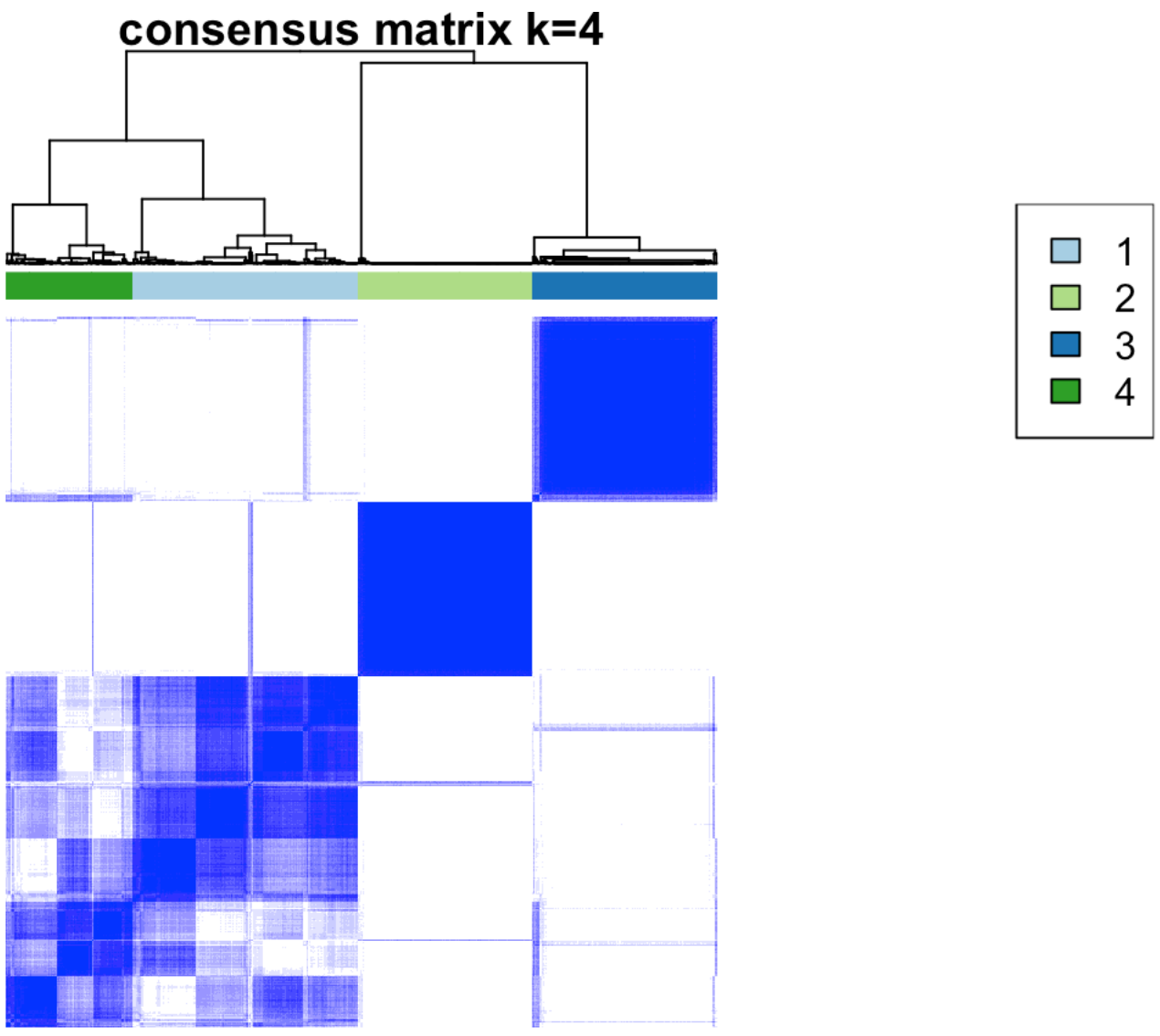


```
## clustered
```

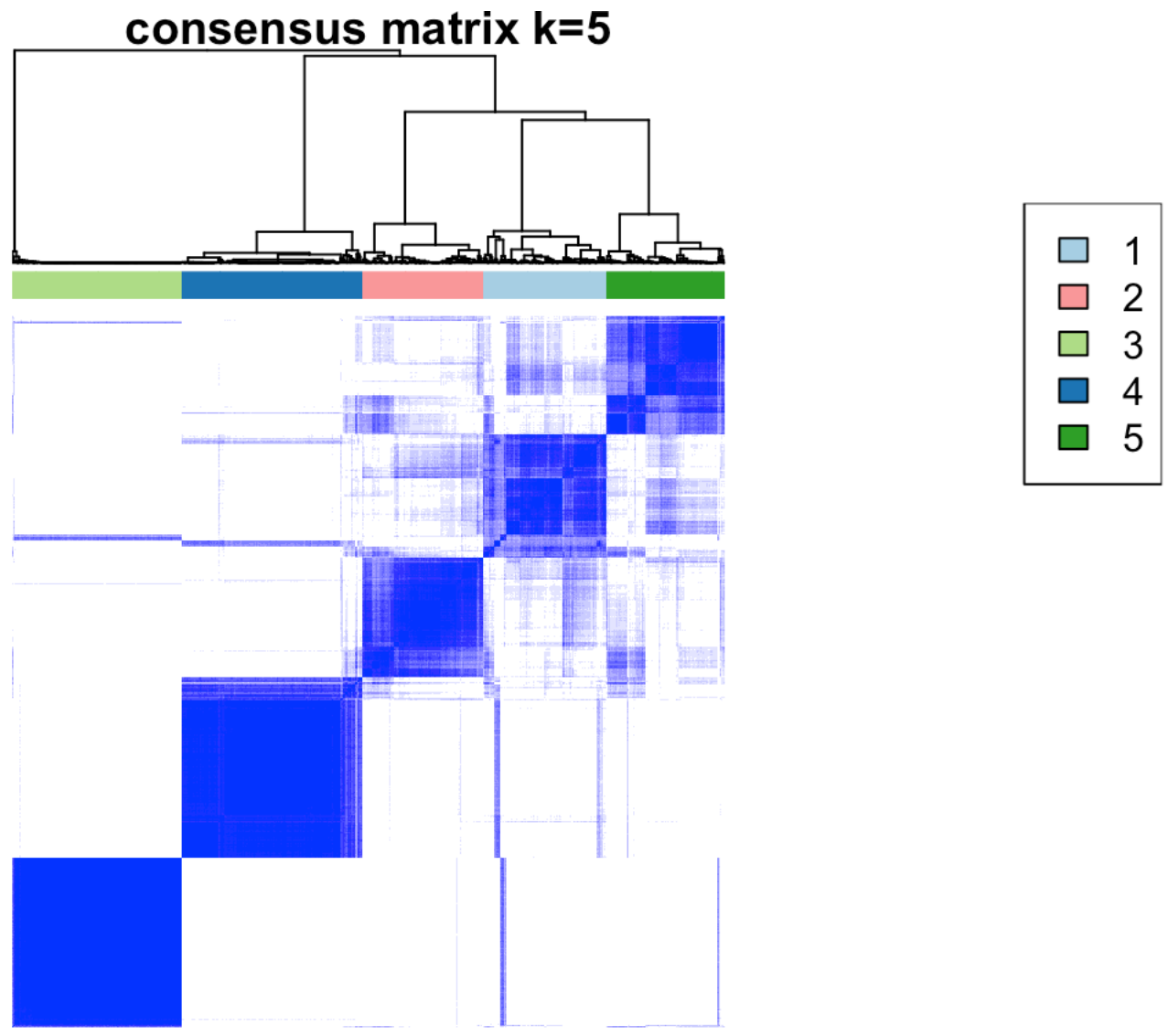
consensus matrix k=5
1
2
3
4
5

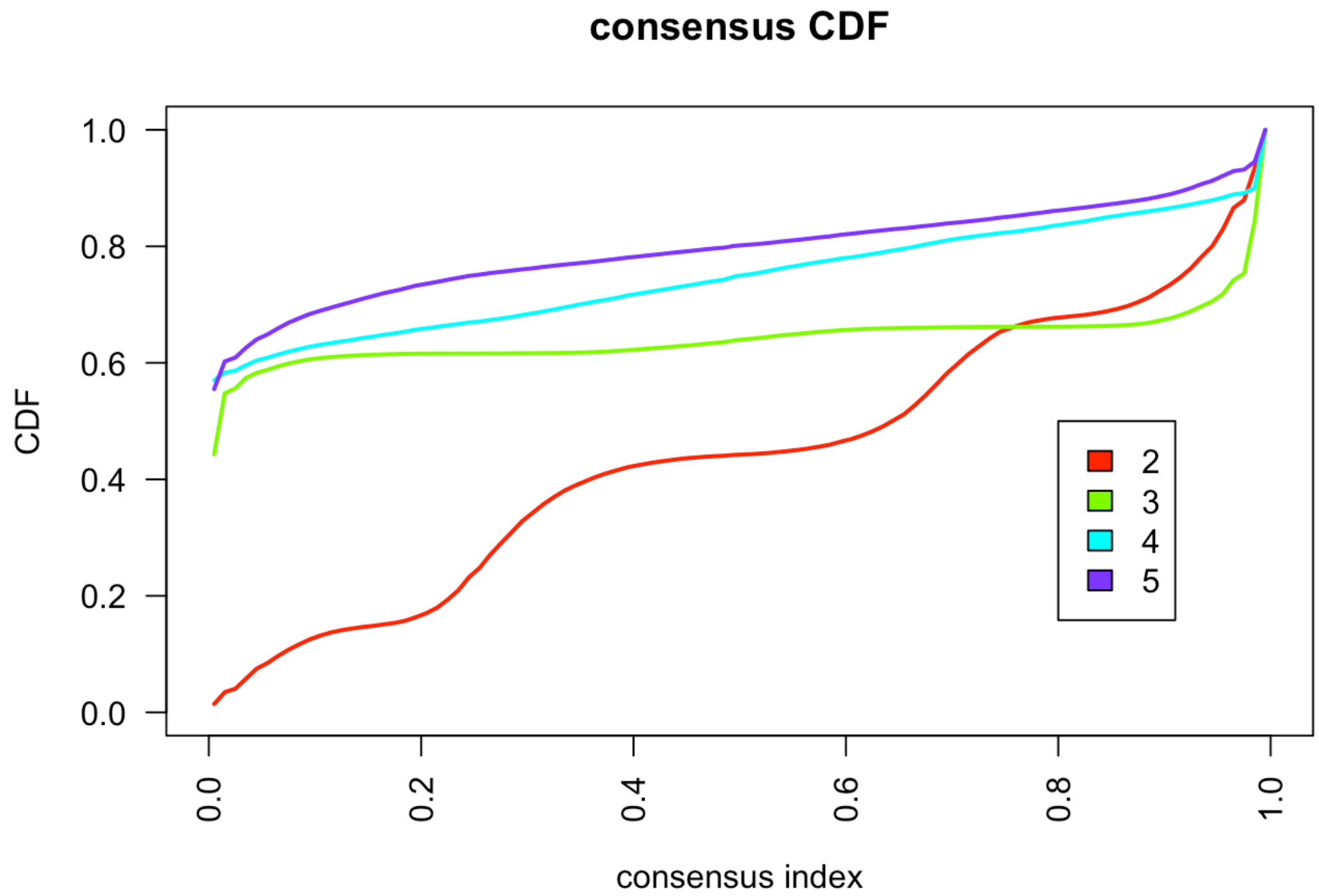
consensus CDF
CDF
consensus index
2
3
4
5

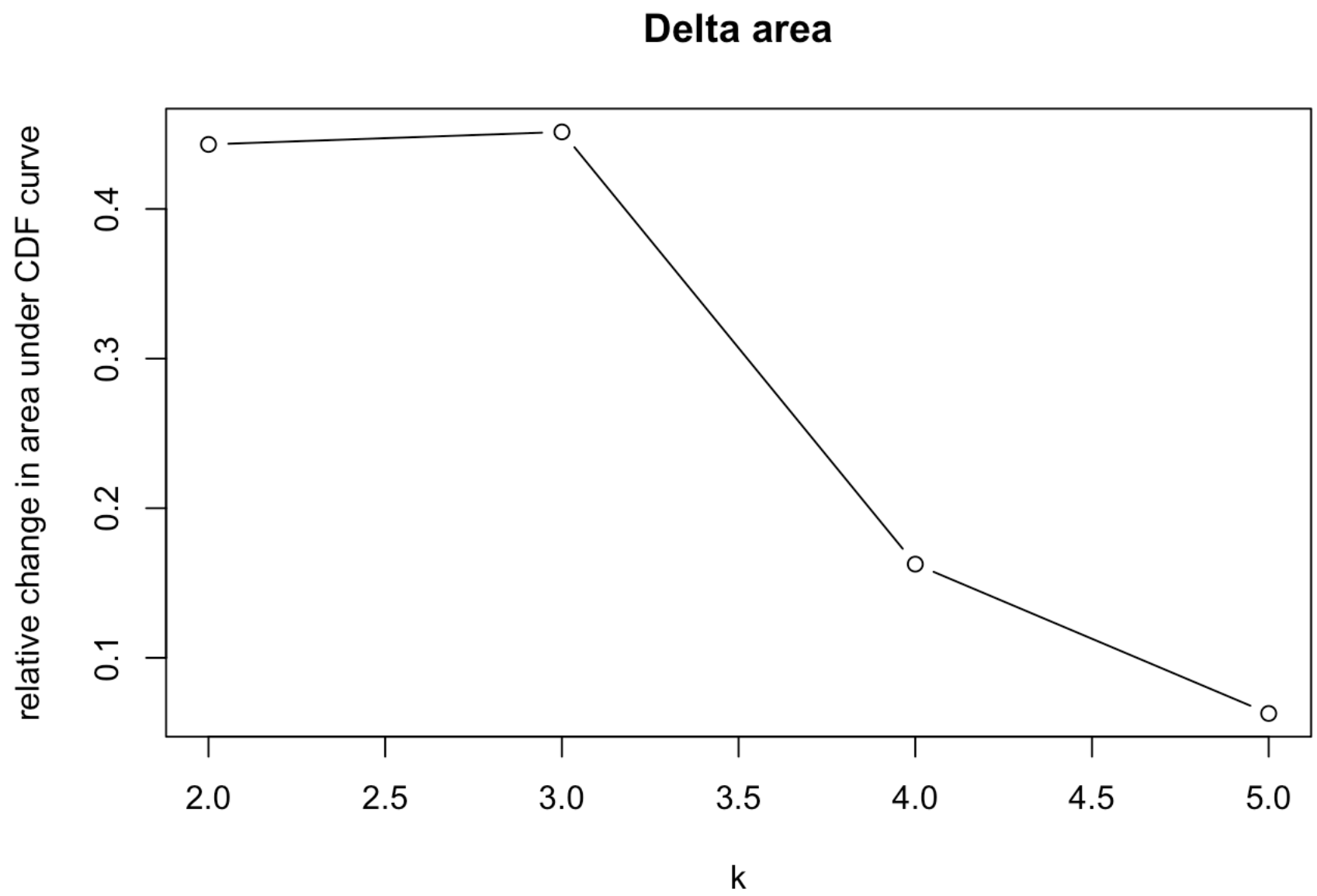
Delta area
relative change in area under CDF curve
k

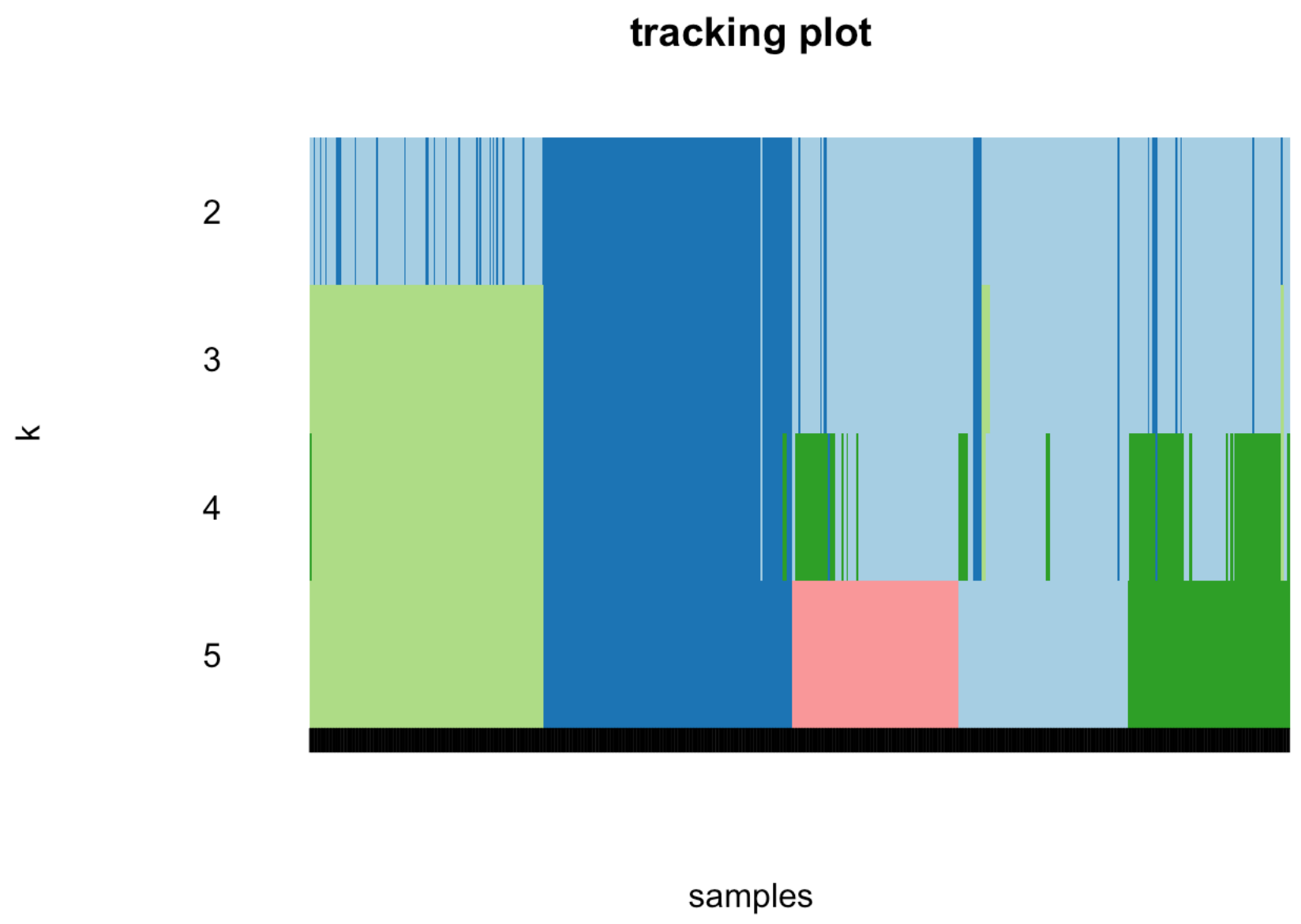

Choosing optimal k

Both diceR and ConsensusClusterPlus function enables a direct comparison of various cluster counts (k) by using consensus matrix plot and the empirical cumulative distribution function (CDF) of the consensus matrix. According to Monti (2003), the CDF derived from the optimal k typically exhibits three characteristics:

- clear square-shaped structure (with distinct boundaries) along the diagonal direction in the consensus plot, suggesting pairs of observations were consistently clustered to (or not clustered to) different groups;
- a three-phase step function in CDF plot, beginning with an initial step near 0 (indicative of the proportion of zeros in the matrix), followed by a flat line across the 0–1 range, and concluding with a final step near 1; and
- the delta area method, which is an 'elbow' effect seen in the relative change of the area under the CDF plot (delta area).

In our example, consensus plots and the CDFs from both packages accurately exhibit features of optimal number of clusters with k=3. However, the identification of k=3 as the optimal cluster count is uniquely discernible using the delta area method in the diceR package.

The CDF method sometimes identified clusters even in data where none existed. To address this, Şenbabaoğlu et al. (2012) introduced the Proportion of Ambiguous Clustering (PAC) score. The optimal k is determined by the lowest PAC score, thereby reducing ambiguity in the consensus. PAC scores are computed in the dice function, which also serves as the default method for selecting the optimal k. ConsensusClusterPlus function require additional calculation to estimate PAC scores. In our example, we set k.method = "all", enabling us to generate consensus results for all values of k and facilitate a manual comparison of different indices.

```
CC_diceR$indices$pac
```

```
##   k HC_Euclidean        KM         GMM
## 1 2    0.4435960 0.9919012 0.336580001
## 2 3    0.4486887 0.7712932 0.009880999
## 3 4    0.4807569 0.4868535 0.276786419
## 4 5    0.4534149 0.4439231 0.287631831
```

```
# 2 cluster
diceR::PAC(CC_CCP[[2]][[1]], lower = 0.05, upper = 0.95)
```

```
## [1] 0.7229767
```

```
# 3 cluster
diceR::PAC(CC_CCP[[3]][[1]], lower = 0.05, upper = 0.95)
```

```
## [1] 0.1216231
```

```
# 4 cluster
diceR::PAC(CC_CCP[[4]][[1]], lower = 0.05, upper = 0.95)
```

```
## [1] 0.2747899
```

```
# 5 cluster
diceR::PAC(CC_CCP[[5]][[1]], lower = 0.05, upper = 0.95)
```

```
## [1] 0.2716542
```

In our example, k=3 was identified correctly for hierarchical clustering and GMM as BC in dice function and ConsensusClusterPlus using Ward linkage method (other linkage methods suggest different results).

Relative criteria from different k were also computed as a part of dice package, which can be extracted as below.

```
# first three rows were indexes for BCs
knitr::kable(as_tibble(CC_diceR$indices$ii$`4`[4:7,]),
             digits = 3) %>%
  kable_styling(full_width = F)
```

Algorithms	calinski_harabasz	dunn	pbm	tau	gamma	c_index	davies_bouldin	mcclain_rao	sd_dis	ray_turi	g_plus	silhouette	s_dbw	C
LCE k=2	124.227	0.094	1.438	0	-0.333	0.299	1.705	0.822	0.932	0.897	0	0.242	0.873	
LCE k=3	140.710	0.095	1.039	0	1.186	0.225	1.579	0.750	0.759	0.763	0	0.257	1.092	
LCE k=4	93.888	0.091	0.595	0	-0.846	0.226	1.467	0.751	1.127	1.661	0	NaN	1.570	
LCE k=5	98.610	0.042	0.633	0	-2.292	0.196	1.479	0.708	1.162	1.470	0	NaN	1.049	

Optimal values:

- Calinski_harabasz: maximum value, k=3 in our example
- dunn: maximum value, k=2 in our example
- gamma: maximum value, k=3 in our example
- c_index: minimum value, k=3 in our example
- davies_bouldin: minimum value, k=3 in our example
- sd: minimum value , k=2 in our example
- s_dbw: minimum value , k=2 in our example
- silhouette: maximum value, k=3 in our example
- Compactness: minimum value, k=5 in our example
- Connectivity: minimum value , k=2 in our example

Internal criteria generated substantial inconsistency even for a simple example as illustrated, which highlighted the additional benefit of the consensus clustering approach.

Permutation test

Permutation testing can be a useful tool for evaluating validity of the consensus clustering results. In our example, the PAC score obtained for k=3 clusters generated through k-means is compared with the PAC scores obtained for 100 random permutations of the source data to derive a p-value (p). The p-value here represents the proportion of PAC scores that are lower than the one calculated in the initial data (observed PAC). ARI can also be used as a permutation marker.

```
# Observed PAC
cc<- diceR::dice(
    dta_pca %>% select(-ID),
    nk = 3, reps = 100, p.item = 0.8,
    algorithms = c("km"),cons.funs = c("kmodes"),
    k.method = "all", seed = 1234)
observed_pac<-cc$indices$pac$KM

# Generate 100 null datasets
set.seed(1234)
null_datasets <- replicate(100, dta_pca %>%  select(-ID)%>%
                                mutate(across(everything(), ~ sample(.))),
                            simplify = FALSE )

# Rerun clustering models
null_results <- NA
for (i in seq_along(null_datasets)) {
  cc<- diceR::dice(
      null_datasets[[i]],
      nk = 3,reps = 100, p.item = 0.8,
      algorithms = c("km"), cons.funs = c("kmodes"), seed = 1234 + i
  )
  null_results[i]<-cc$indices$pac$KM
}

p=mean(null_results<observed_pac)
null_results<-  as.data.frame(null_results)
names(null_results)<-"pac_permuted"
```

```
# Null distribution of PAC vs PAC observed
ggplot(null_results ,aes(x=pac_permuted)) +
        geom_histogram(fill='skyblue',bins=10) +
        geom_vline(aes(xintercept=observed_pac),col='red') +
        annotate("text",x = observed_pac, y= 20,
                 label = paste0("Observed PAC=",round(observed_pac,3),
                                ", p=",p), size=5, color = "red",hjust=-0.1) +
        scale_x_continuous(breaks=seq(0,1,by=0.1)) +
        labs(x="PAC",y="Number of permutations",title="PAC null distribution (n=100 permutations)") +
        theme_bw() +
        theme(plot.title=element_text(hjust=0.5,size=15),text=element_text(size=14),plot.margin = unit(c(20,0,20,0),"pt"))
```

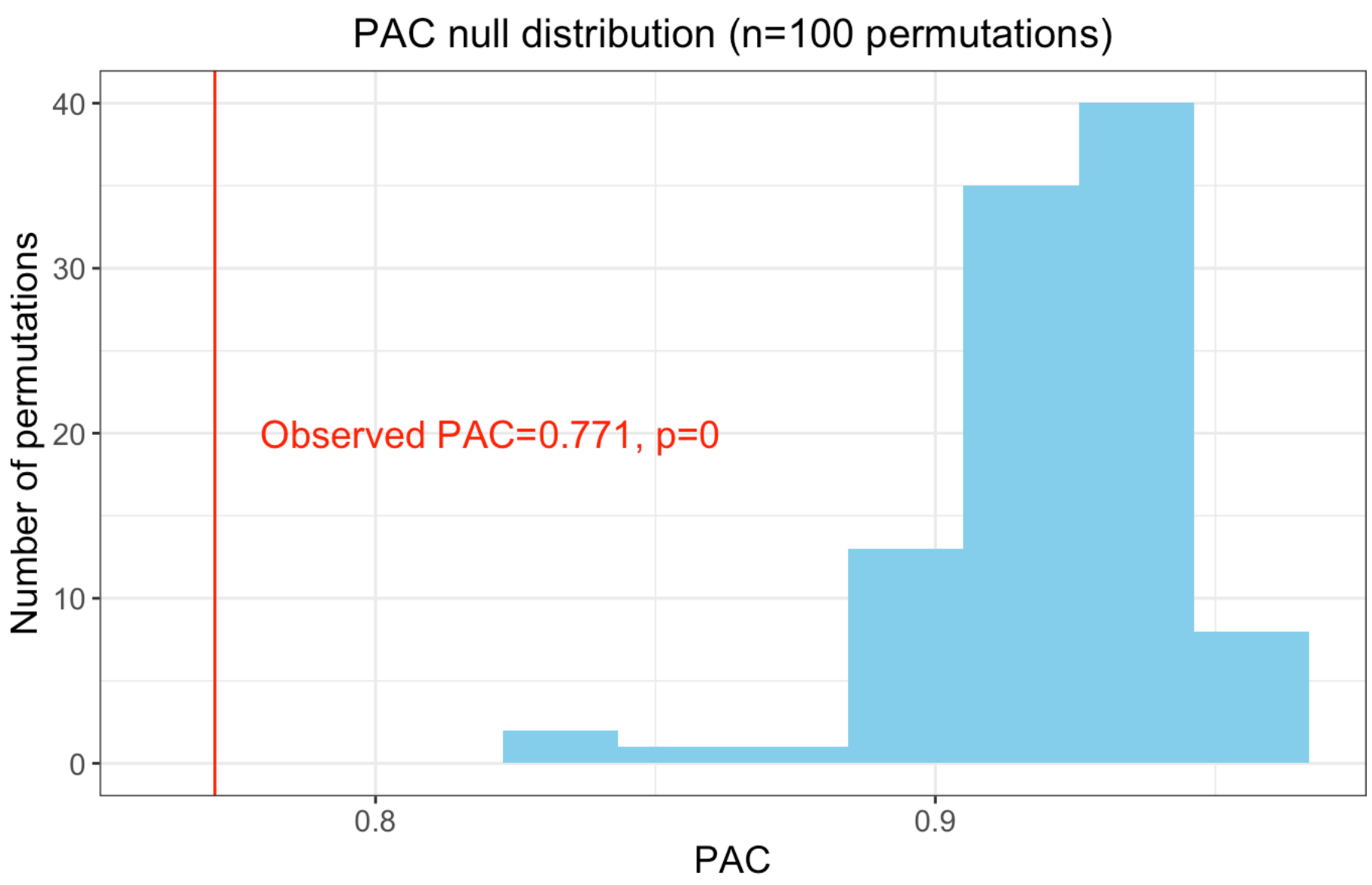

This is particularly useful when no underlying clustering truly presented. Below code shows permutation test results when we use a data set with underlying clustering structure removed.

```
# Observed PAC
set.seed(1234)
dta_pca_dis <- dta_pca %>%  select(-ID)%>%
      mutate(across(everything(), ~ sample(.)))

cc<- diceR::dice(
    dta_pca_dis,
    nk = 3, reps = 100, p.item = 0.8,
    algorithms = c("km"),cons.funs = c("kmodes"),
    k.method = "all", seed = 1234)
observed_pac<-cc$indices$pac$KM

# Generate 100 null datasets
set.seed(1234)
null_datasets <- replicate(100, dta_pca_dis %>%
                                mutate(across(everything(), ~ sample(.))),
                            simplify = FALSE )

# Rerun clustering models
null_results <- NA
for (i in seq_along(null_datasets)) {
  cc<- diceR::dice(
      null_datasets[[i]],
      nk = 3,reps = 100, p.item = 0.8,
      algorithms = c("km"), cons.funs = c("kmodes"), seed = 1234 + i
  )
  null_results[i]<-cc$indices$pac$KM
}

p=mean(null_results<observed_pac)
null_results<-  as.data.frame(null_results)
names(null_results)<-"pac_permuted"
```

```
# Null distribution of PAC vs PAC observed
ggplot(null_results ,aes(x=pac_permuted)) +
        geom_histogram(fill='skyblue',bins=10) +
        geom_vline(aes(xintercept=observed_pac),col='red') +
        annotate("text",x = observed_pac, y= 20,
                 label = paste0("Observed PAC=",round(observed_pac,3),
                                ", p=",p), size=5, color = "red",hjust=-0.1) +
        scale_x_continuous(breaks=seq(0,1,by=0.1)) +
        labs(x="PAC",y="Number of permutations",title="PAC null distribution (n=100 permutations)") +
        theme_bw() +
        theme(plot.title=element_text(hjust=0.5,size=15),text=element_text(size=14),plot.margin = unit(c(20,0,20,0),"pt"))
```

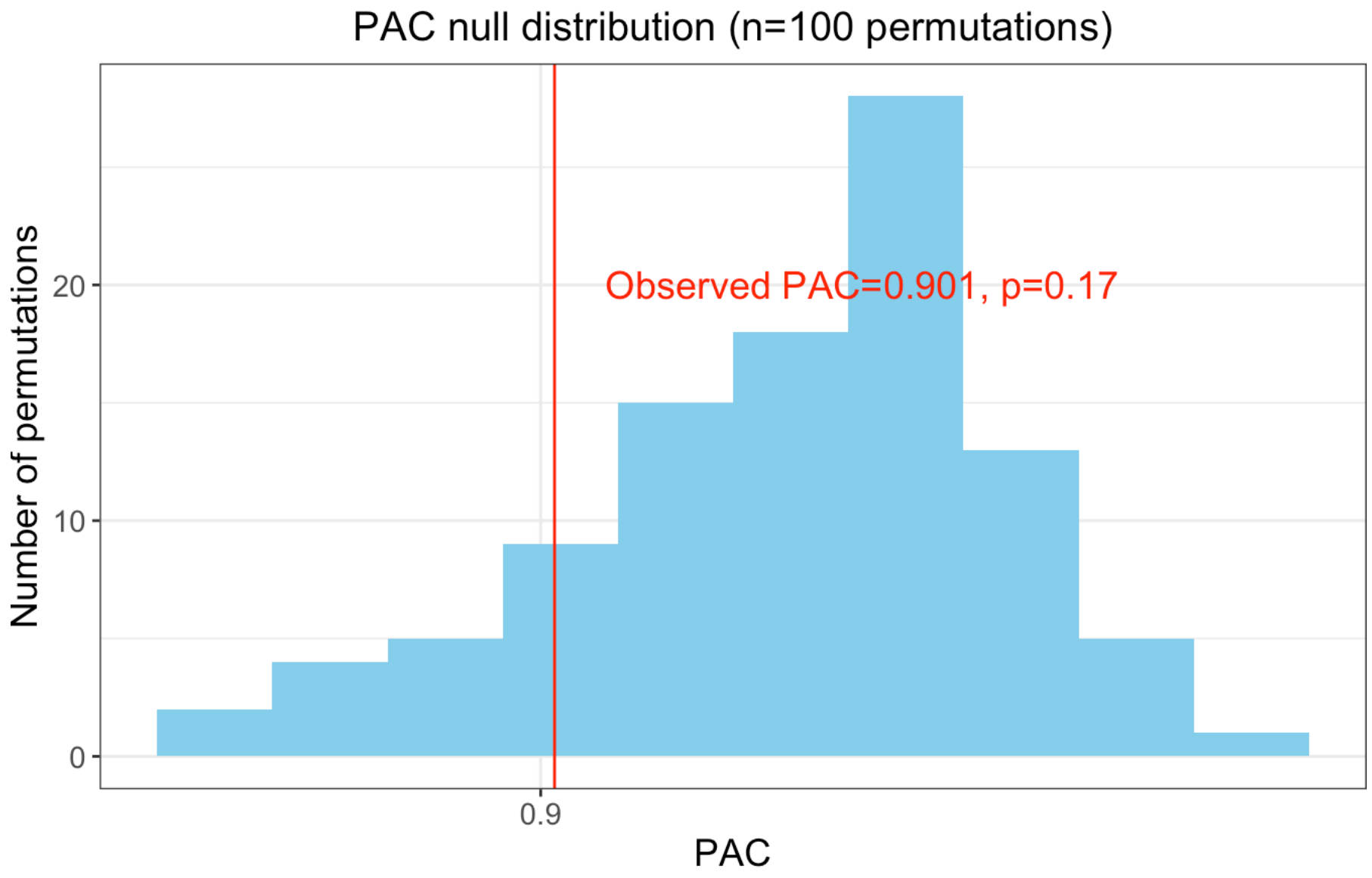

Missing data

Missing data poses extra challenges for ensemble clustering, an issue not directly handled by existing packages. Nonetheless, data imputation can be incorporated into available packages. In this demonstration, we show how to combine multiple imputation with ensemble clustering.

```
# randomly remove 5% data
random_missing<-function(x,p){
  # x=data; p=percentage missing
  x[sample(1:length(x), round(length(x)*p), replace = FALSE)]<-NA
  return(x)
}
dta_missing<-dta %>% mutate_all(random_missing,0.05)
```

For this analysis, we employed 10 imputed datasets. A higher number of imputed datasets may be required if there is a considerable amount of missing data or if there is significant variation in the consensus clustering results among the imputed datasets.

```
# Impute data
dta_imp <- lapply(1:10, function(x) missRanger::missRanger(dta_missing))

# Extract PCA
extract_PCA<-function(data,c){
   PCA <- prcomp(data,center = TRUE, scale. = TRUE )
   data <- as_tibble(PCA$x[, 1:c]) %>% mutate_all(~ as.numeric(scale(.)))
}
dta_pca<-lapply(dta_imp, extract_PCA,5)
```

```
# Consensus Clustering using k=3
extract_CC<-function(x){
  CC_results<-diceR::dice(x,  nk = 3, reps = 50,  algorithms = c("hc"),
                         hc.method="ward.D2",
                         cons.funs = c("kmodes"),  seed= 1234 )
  CC_results$clusters[,1]
}
# Split this job across 5 cores using parallel computation
CC_diceR_imp <- parallel::mclapply( dta_pca,  extract_CC, mc.cores = 5)  # mc.cores must be set at 1 if using Windows
CC_diceR_imp<-do.call(cbind, CC_diceR_imp)
```

The final clustering results can be extracted as an additional consensus process. Here we use the LCE function from diceR.

```
dta_group$impute_diceR<- diceR::LCE(CC_diceR_imp, k=3)
table(dta_group$group, dta_group$impute_diceR)
```

```
##
##            1   2   3
##   Group1 297   1   0
##   Group2   0   0 150
##   Group3   0 148   0
```

Alternatively, users can modify our code in the section “Manually generate BCs” to incorporate the missing data imputation process. This approach can allow the consensus function to directly account for both imputed and sampled data.
