# Supplementary material for: Ensemble clustering: A practical tutorial
Source: Behav Res Methods. 2026 Sep 3;58(10):286. doi: 10.3758/s13428-026-03158-y (PMC13541929; doi:10.3758/s13428-026-03158-y)
Supplement: Supplementary file 3 [file 13428_2026_3158_MOESM4_ESM.pdf]

- Load libraries
- Overview of the cross-validation pipeline
- Functions
- Import data
- Functions
  - Stability measure
  - Inner-loop resampling
  - Outer-loop resampling
  - Running ensemble clustering across multiple k values
  - Co-association matrix
- Example: obtaining clustering results of k=2-5 using k-means as BC.
  - Plot co-association matrix
  - CDF and Delta plot
  - Distribution of ARIs
- Example: obtaining clustering results of k=2-5 using hierarchical clutering as BC.
  - Plot co-association matrix
  - CDF and Delta plot
  - View distribution of ARI
- Example: obtaining clustering results of k=2-5 using mixed k-means and hierarchical clutering as BC.
  - Plot co-association matrix
  - CDF and Delta plot
  - View distribution of ARI

Ensemble clustering: A practical tutorial

Supplementary Material IV: Cross-Validation pipeline

Load libraries

```
library(pacman)
p_load(
  "tidyverse", "ggplot2", "gridExtra", "kableExtra", "knitr", "here",
  "qgraph", "arsenal", "stringr", "Hmisc", "tidyselect", "readxl", "gdata",
  "ggthemes", "scales", "FactoMineR", "smacof", "networktools", "Rtsne",
  "factoextra", "ConsensusClusterPlus", "NMF", "diceR", "RclusTool",
  "data.table", "aricode","dummy_cols" ,"VIM","poLCA", "parallel", "patchwork")
## Provides detailed information and environment settings for the current R sess
ion
sessionInfo()
```

```
## R version 4.5.1 (2025-06-13)
## Platform: aarch64-apple-darwin20
## Running under: macOS Tahoe 26.4.1
##
## Matrix products: default
## BLAS:   /Library/Frameworks/R.framework/Versions/4.5-arm64/Resources/lib/libR
blas.0.dylib
## LAPACK: /Library/Frameworks/R.framework/Versions/4.5-arm64/Resources/lib/libR
lapack.dylib;  LAPACK version 3.12.1
##
## locale:
## [1] en_US.UTF-8/en_US.UTF-8/en_US.UTF-8/C/en_US.UTF-8/en_US.UTF-8
##
## time zone: Australia/Melbourne
## tzcode source: internal
##
## attached base packages:
##  [1] parallel  grid      tcltk     stats     graphics  grDevices utils
##  [8] datasets  methods   base
##
## other attached packages:
##  [1] patchwork_1.3.2             poLCA_1.6.0.2
##  [3] MASS_7.3-65                 scatterplot3d_0.3-44
##  [5] VIM_7.0.0                   aricode_1.0.3
##  [7] data.table_1.18.2.1         RclusTool_0.91.61
##  [9] tkrplot_0.0-32              tcltk2_1.6.1
## [11] diceR_3.1.0                 NMF_0.28
## [13] Biobase_2.70.0              BiocGenerics_0.56.0
## [15] generics_0.1.4              cluster_2.1.8.1
## [17] rngtools_1.5.2              registry_0.5-1
## [19] ConsensusClusterPlus_1.74.0 factoextra_1.0.7
## [21] Rtsne_0.17                  networktools_1.6.0
## [23] smacof_2.1-7                e1071_1.7-16
## [25] colorspace_2.1-2            plotrix_3.8-4
## [27] FactoMineR_2.12             scales_1.4.0
## [29] ggthemes_5.1.0              gdata_3.0.1
## [31] readxl_1.4.5                tidyselect_1.2.1
## [33] Hmisc_5.2-4                 arsenal_3.6.3
## [35] qgraph_1.9.8                here_1.0.2
## [37] knitr_1.50                  kableExtra_1.4.0
## [39] gridExtra_2.3               lubridate_1.9.4
## [41] forcats_1.0.1               stringr_1.5.2
## [43] dplyr_1.2.1                 purrr_1.1.0
## [45] readr_2.1.5                 tidyr_1.3.2
## [47] tibble_3.3.0                ggplot2_4.0.2
## [49] tidyverse_2.0.0             pacman_0.5.1
##
## loaded via a namespace (and not attached):
##   [1] RColorBrewer_1.1-3   doParallel_1.0.17    tools_4.5.1
##   [4] mlr3learners_0.14.0  backports_1.5.0      R6_2.6.1
##   [7] DT_0.34.0            jomo_2.7-6           withr_3.0.2
##  [10] sp_2.2-0             fdrtool_1.2.18       cli_3.6.5
##  [13] textshaping_1.0.4    flashClust_1.01-2    sandwich_3.1-1
##  [16] sass_0.4.10          nnls_1.6             mvtnorm_1.3-3
##  [19] S7_0.2.0             robustbase_0.99-6    randomForest_4.7-1.2
```

```
##  [22] proxy_0.4-27          pbapply_1.7-4         pbivnorm_0.6.0
##  [25] mlr3tuning_1.5.1      systemfonts_1.3.1     foreign_0.8-90
##  [28] svglite_2.2.2         R.utils_2.13.0        paradox_1.0.1
##  [31] parallelly_1.46.1     rstudioapi_0.17.1     shape_1.4.6.1
##  [34] gtools_3.9.5          car_3.1-3             leaps_3.2
##  [37] Matrix_1.7-3          abind_1.4-8           R.methodsS3_1.8.2
##  [40] weights_1.1.2         lifecycle_1.0.5       multcomp_1.4-29
##  [43] yaml_2.3.10           carData_3.0-5         lavaan_0.6-20
##  [46] crayon_1.5.3          mitml_0.4-5           lattice_0.22-7
##  [49] pillar_1.11.1         boot_1.3-31           estimability_1.5.1
##  [52] corpcor_1.6.10        codetools_0.2-20      pan_1.9
##  [55] glue_1.8.0            vcd_1.4-13            vctrs_0.7.1
##  [58] png_0.1-8             Rdpack_2.6.4          cellranger_1.1.0
##  [61] gtable_0.3.6          cachem_1.1.0          xfun_0.56
##  [64] rbibutils_2.3         coda_0.19-4.1         reformulas_0.4.1
##  [67] survival_3.8-3        iterators_1.0.14      TH.data_1.1-5
##  [70] nlme_3.1-168          bbotk_1.8.1           rprojroot_2.1.1
##  [73] mlr3pipelines_0.10.0 bslib_0.9.0           mlr3_1.3.0
##  [76] rpart_4.1.24          nnet_7.3-20           mlr3misc_0.19.0
##  [79] mnormt_2.1.1          emmeans_2.0.0         compiler_4.5.1
##  [82] mda_0.5-5             glmnet_4.1-10         htmlTable_2.4.3
##  [85] lgr_0.5.2             mice_3.19.0           xml2_1.4.0
##  [88] checkmate_2.3.3       lmtest_0.9-40         DEoptimR_1.1-4
##  [91] psych_2.5.6           quadprog_1.5-8        multcompView_0.1-10
##  [94] palmerpenguins_0.1.1 digest_0.6.37         minqa_1.2.8
##  [97] rmarkdown_2.30        htmltools_0.5.8.1     pkgconfig_2.0.3
## [100] jpeg_0.1-11           base64enc_0.1-3       lme4_1.1-37
## [103] fastmap_1.2.0         rlang_1.1.7           htmlwidgets_1.6.4
## [106] farver_2.1.2          jquerylib_0.1.4       zoo_1.8-15
## [109] jsonlite_2.0.0        mmand_1.7.0           mclust_6.1.2
## [112] eigenmodel_1.11       R.oo_1.27.1           magrittr_2.0.4
## [115] polynom_1.4-1         Formula_1.2-5         wordcloud_2.6
## [118] Rcpp_1.1.0            stringi_1.8.7         plyr_1.8.9
## [121] conclust_1.1          listenv_0.10.0        ggrepel_0.9.6
## [124] splines_4.5.1         hms_1.1.3             igraph_2.2.0
## [127] uuid_1.2-1            ranger_0.18.0         reshape2_1.4.4
## [130] stats4_4.5.1          evaluate_1.0.5        BiocManager_1.30.27
## [133] laeken_0.5.3          nloptr_2.2.1          tzdb_0.5.0
## [136] foreach_1.5.2         future_1.69.0         reshape_0.8.10
## [139] gridBase_0.4-7        broom_1.0.10          xtable_1.8-4
## [142] glasso_1.11           viridisLite_0.4.2     class_7.3-23
## [145] SearchTrees_0.5.5     ellipse_0.5.0         corrplot_0.95
## [148] timechange_0.3.0      globals_0.19.0
```

```
# ensure select and filter function always sourced from dplyr package
conflicted::conflict_prefer("select", "dplyr")
```

```
## [conflicted] Will prefer dplyr::select over any other package.
```

```
conflicted::conflict_prefer("filter", "dplyr")
```

```
## [conflicted] Will prefer dplyr::filter over any other package.
```

```
conflicted::conflict_prefer("combine", "dplyr")
```

```
## [conflicted] Will prefer dplyr::combine over any other package.
```

```
RNGkind("L'Ecuyer-CMRG")
```

Overview of the cross-validation pipeline

Functions

This pipeline implements a two-level resampling framework for evaluating the stability and reproducibility of ensemble clustering solutions across different numbers of clusters (k). This structure is chosen to reduce simulation time as imputation in the inner-loop can be resource intensive.

The framework contains: * Inner-loop: repeatedly samples variables and generates ensemble clustering solutions * Outer-loop: repeatedly samples observations * Integration of inner- and outer- loop clustering results * Obtain stability indexes of the inner-loop and outer-loop

The implementation is modular so that different clustering algorithms can easily be substituted into the framework.

Import data

```
# Import source data
dta <- readRDS(here::here("cleaned_data.rds"))
dta_group <- readRDS(here::here("source_data_with_label.rds"))
dta_group <- dta_group %>%
    filter(ID %in% dta$ID)
```

Functions

Stability measure

The Adjusted Rand Index (ARI) is used to quantify agreement between clustering solutions. Pairwise ARI is calculated across all clustering repetitions to estimate clustering stability. For any ensembelling stage, we can obtain a distribution of ARIs.

```
# Calculate ARI between two clustering solutions
cal_ARI_ij<-function(i,j,data){
    return<-1
    if(i!=j){
        #
        data<-data[,c(i,j)] %>% na.omit()
        return<-aricode::ARI(data[,1],data[,2])
    }
    return
}

# Calculate pairwise ARI across all clustering repetitions
ARI<-function(cluster_results){
  cal_ARI <- Vectorize(cal_ARI_ij, vectorize.args=list("i","j"))
  ARI <- outer(1:length(cluster_results),1:length(cluster_results),
               cal_ARI,data=cluster_results)
  diag(ARI) <- NA
  return(ARI)
}
```

Inner-loop resampling

The inner loop evaluates the stability of clustering solutions under repeated feature perturbation.

At each iteration: 1. A subset of variables is randomly sampled 2. Principal component analysis (PCA) is performed 3. A base clustering (BC) algorithm is applied 4. Clustering agreement across repetitions is quantified using ARI

The resulting BC solutions are then aggregated into an ensemble clustering solution.

```
# Inner loop function
inner_loop <- function(data, k = 3, model ="km",
                       reps = 50, p.col = 0.8, npc = 5, seed = 1234) {

    set.seed(seed)
    ids <- data$ID
    # storage
    BCs <- tibble(ID = ids)

    # Generate BCs
    for(i in 1:reps){
        # sample columns only
        cols <- sample(
            names(data %>% select(-ID)),
            size = round((ncol(data) - 1) * p.col)
        )
        sample_data <- data %>%
            select(ID, all_of(cols))

        # PCA
        pca <- prcomp(
            sample_data %>% select(-ID),
            center = TRUE,
            scale. = TRUE
        )
        pca <- as_tibble(pca$x[, 1:npc]) %>%
            mutate_all(~ as.numeric(scale(.)))
        # BC
        if(model == "km"){
            results <- kmeans( pca, centers = k, nstart = 20)
            cluster <- results$cluster
        }else if (model == "hc"){
            results <- hclust(dist(pca), method = "ward.D2")
            cluster <- cutree(results, k)
        }else if (model == "mixed") {
            if (i< reps/2){
                results <- kmeans( pca, centers = k, nstart = 20)
                cluster <- results$cluster
            }else {
                results <- hclust(dist(pca), method = "ward.D2")
                cluster <- cutree(results, k)
            }
        }

         BCs[[paste0("Cluster", i)]] <- cluster
    }
    BCs<-as.data.frame(BCs)

    # remove ID
    BCs_mat <- BCs %>%
        select(-ID)

    # ARI between BCs
    ari_matrix <- ARI(BCs_mat)
    ari_mean <- mean(ari_matrix, na.rm = TRUE)
```

```
    ari_sd <- sd(ari_matrix, na.rm = TRUE)


    # Binary association matrix
    binary_mat <- fastDummies::dummy_cols(
        BCs_mat,
        select_columns = names(BCs_mat),
        remove_selected_columns = TRUE,
        ignore_na = TRUE
    )

    # Consensus clustering
    ensemble <- kmeans( binary_mat,centers = k,nstart = 50 )
    final_cluster <- ensemble$cluster
    list(
        cluster = final_cluster,
        ari_mean = ari_mean,
        ari_sd = ari_sd
    )
}
```

Outer-loop resampling

The outer loop evaluates the reproducibility of ensemble clustering solutions under repeated observation resampling.

At each iteration: 1. A subset of observation is sampled 2. The full inner-loop ensemble clustering procedure is performed for each subset 3. Cluster assignments are merged back into the full sample 4. Agreement across outer-loop solutions is quantified using ARI

This framework evaluates clustering robustness across both feature-level and observation-level perturbations.

```
# Outer loop function
outer_loop <- function(data, k = 3, model ="km", outer_reps = 50,  inner_reps =
50,
                       p.row = 0.8, p.col = 0.8, npc = 5, seed = 1234){
    set.seed(seed)
    # Storage
    outer_clusters <- tibble(ID = data$ID)
    inner_ari_mean <- c()
    inner_ari_sd <- c()
    # Outer resampling
    for(i in 1:outer_reps){
        # Sample rows
        rows <- sample( 1:nrow(data), size = round(nrow(data) * p.row))
        sample_data <- data[rows, ]

        # Run inner loop
        results <- inner_loop(
            data = sample_data,
            k = k,
            model = model,
            reps = inner_reps,
            p.col = p.col,
            npc = npc,
            seed = seed + i
        )

        # Store ARI from inner loop
        inner_ari_mean[i] <- results$ari_mean
        inner_ari_sd[i] <- results$ari_sd


        # Store cluster labels
        cluster <- tibble(
            ID = sample_data$ID,
            cluster = results$cluster
        )

        names(cluster)[2] <- paste0("Outer", i)

        # Merge back
        outer_clusters <- outer_clusters %>%
            left_join(cluster, by = "ID")
    }
    # Remove ID
    outer_clusters <- outer_clusters %>% select(-ID)

    # Outer-loop ARI
    ari_outer <- ARI(as.data.frame(outer_clusters))
    ari_outer_mean <- mean(ari_outer, na.rm = TRUE )
    ari_outer_sd <- sd( ari_outer, na.rm = TRUE)

    # Imputation, missRanger is used to impute missing cluster assignments
    outer_clusters <- missRanger::missRanger( outer_clusters %>% mutate_all(as.f
actor),
                                  verbose = FALSE, pmm.k = 3)  # using predictive mean
```

```
matching
    outer_clusters<- outer_clusters %>% mutate_all(as.numeric)
    # Co-association matrix
    cm <- diceR::consensus_matrix( as.matrix(outer_clusters))

    # Final hierarchical clustering
    hc <- hclust( dist(1 - cm), method = "ward.D2")
    final_cluster <- cutree(hc, k = k)

    # PAC
    pac <- diceR::PAC(cm, lower = 0.05, upper = 0.95 )

    # Return results
    list(
        cluster = final_cluster,
        cm = cm,
        bc=outer_clusters,
        pac = pac,
        inner_ari_mean = mean(inner_ari_mean, na.rm = TRUE),
        inner_ari_sd = mean(inner_ari_sd, na.rm = TRUE),
        outer_ari_mean = ari_outer_mean,
        outer_ari_sd = ari_outer_sd
    )
}
```

Running ensemble clustering across multiple k values

The following wrapper function applies the full nested resampling framework across multiple candidate cluster solutions (k). Parallel computation is used to reduce computational burden, which can be used in macOS or linux systems. If using Windows system, set cores = 1.

```r
# Run different k using parallel computation
run_ensemble_cv <- function(data,
                            k_range = 2:5,
                            model = "km",
                            outer_reps = 50,
                            inner_reps = 50,
                            p.row = 0.8,
                            p.col = 0.8,
                            npc = 5,
                            seed = 1234,
                            cores = 5) {

    results <- parallel::mclapply( k_range,
        function(k){
            outer_loop(
                data = data,
                k = k,
                model= model,
                outer_reps = outer_reps,
                inner_reps = inner_reps,
                p.row = p.row,
                p.col = p.col,
                npc = npc,
                seed = seed + k
            )
        },
        mc.cores = cores,
        mc.set.seed = TRUE,
        mc.preschedule = TRUE,
        mc.cleanup = TRUE

    )

    names(results) <- paste0("k_", k_range)
    results
}
```

Co-association matrix

The Co-association matrix is extracted for the outerloop.

```
plot_cm <- function(results, k){
    cm <- results[[paste0("k_", k)]]$cm
    cols <- colorRampPalette(
        c("white", "#67A9CF", "#2166AC")
    )(100)

    pheatmap::pheatmap(
        cm,
        color = cols,
        clustering_distance_rows = as.dist(1 - cm),
        clustering_distance_cols = as.dist(1 - cm),
        clustering_method = "ward.D2",
        main = paste0(
            "Outer-loop consensus matrix (k = ",
            k,
            ")"
        )
    )
}
```

Example: obtaining clustering results of k=2-5 using k-means as BC.

```
ensemble_cv<-run_ensemble_cv(dta,  npc=5, seed = 1111)
```

Plot co-association matrix

```
plot_cm(ensemble_cv, 2)
```

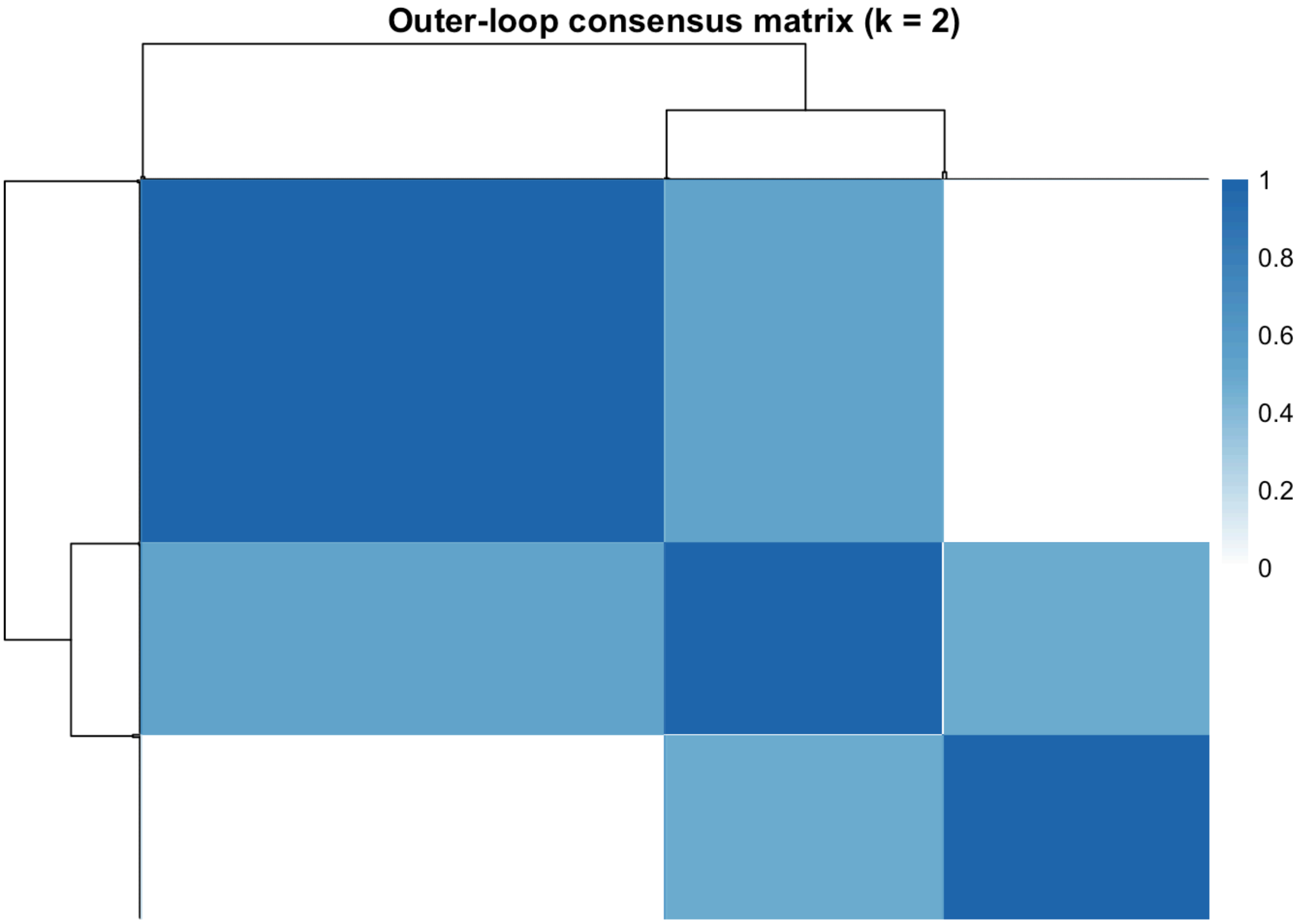


```
plot_cm(ensemble_cv, 3)
```

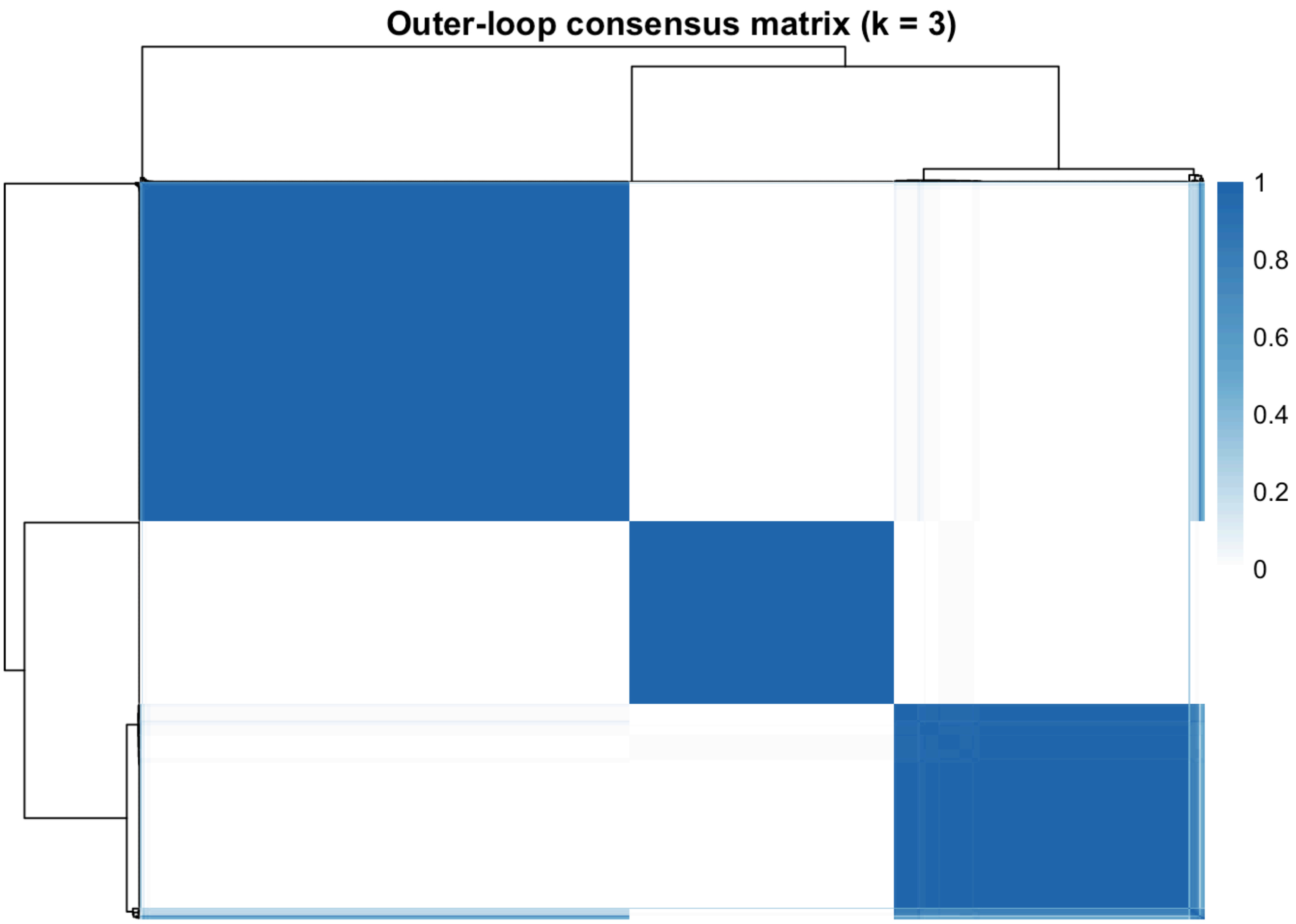


```
plot_cm(ensemble_cv, 4)
```

Outer-loop consensus matrix (k = 4)

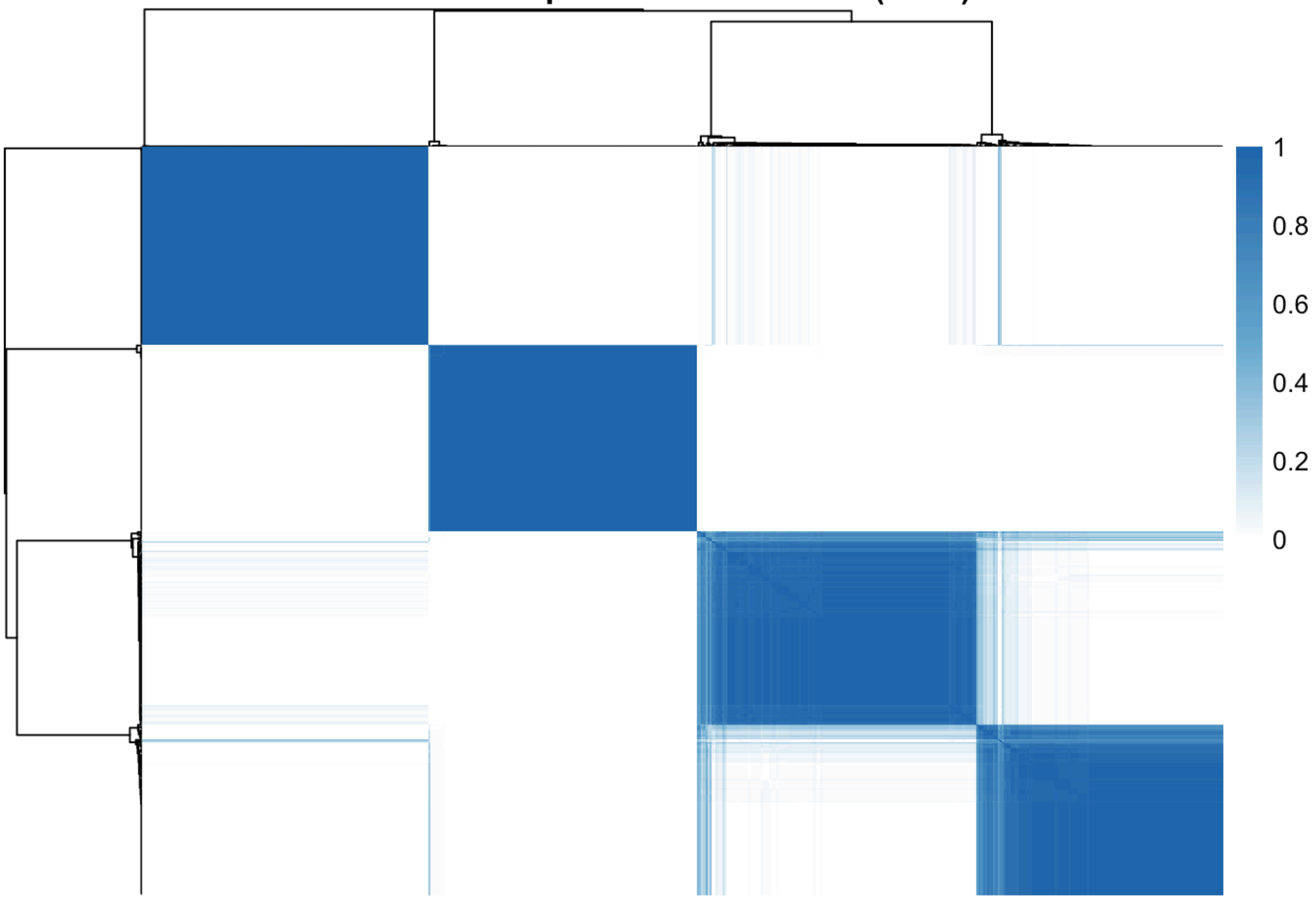


```
plot_cm(ensemble_cv, 5)
```

Outer-loop consensus matrix (k = 5)

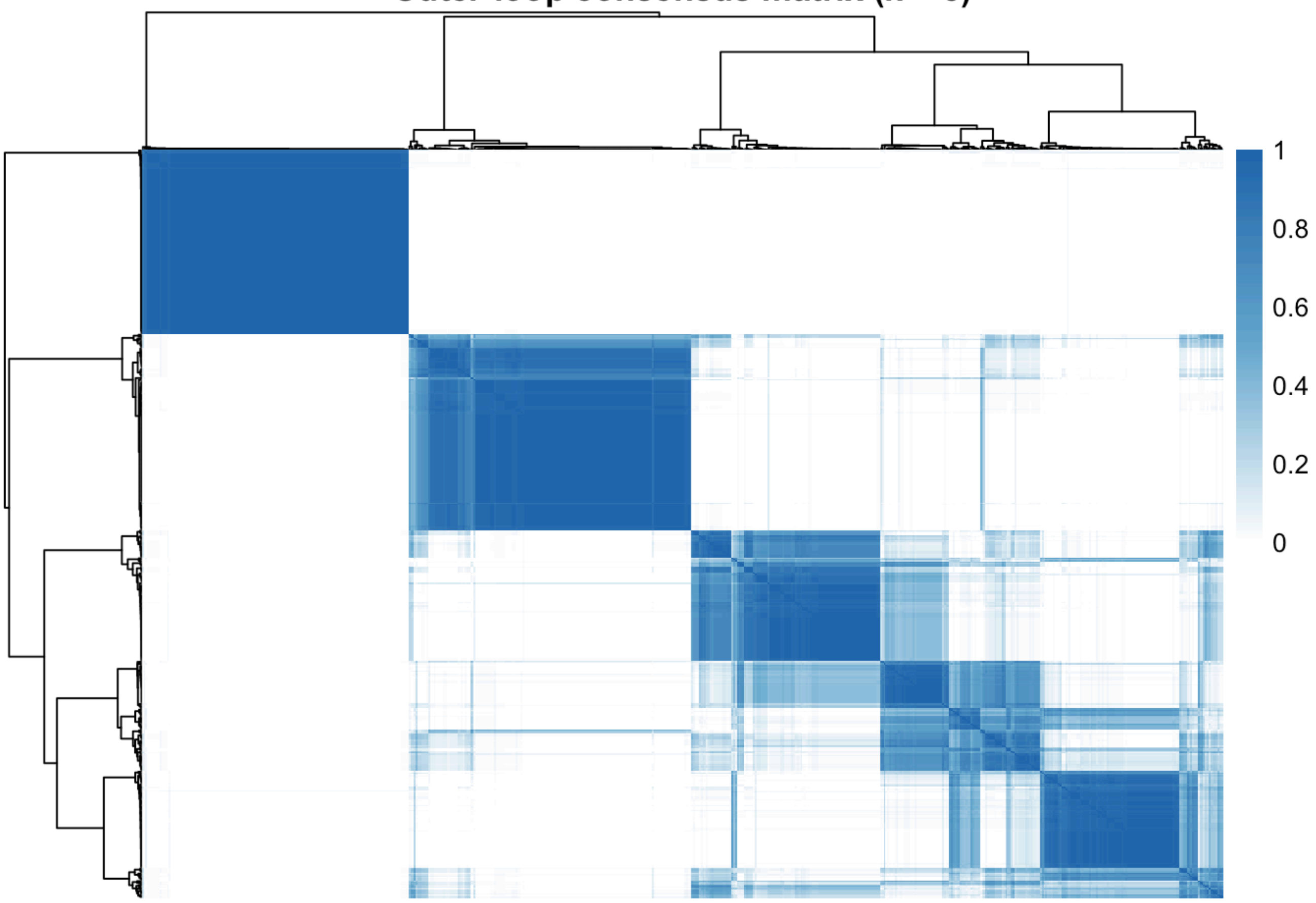

CDF and Delta plot

CDF and Delta plot obtained for outerloop.

```
ks_names<- c("2","3","4","5")
# extract consensus matrices
cms <- lapply(ensemble_cv, function(x) x$bc)
n <- nrow(cms[[1]])
r <- ncol(ensemble_cv[[1]]$bc)

# build fake diceR ensemble array
cm_array <- array(
  NA,
  dim = c(n, r, 1, length(cms)),
  dimnames = list(
    paste0("ID", seq_len(n)),
    paste0("R", seq_len(r)),
    "KM",
    ks_names
  )
)

for(i in seq_along(cms)){
  cm_array[, , 1, i] <- as.matrix(cms[[i]])
}

# run plots
cdf<-diceR::graph_cdf(cm_array)
```

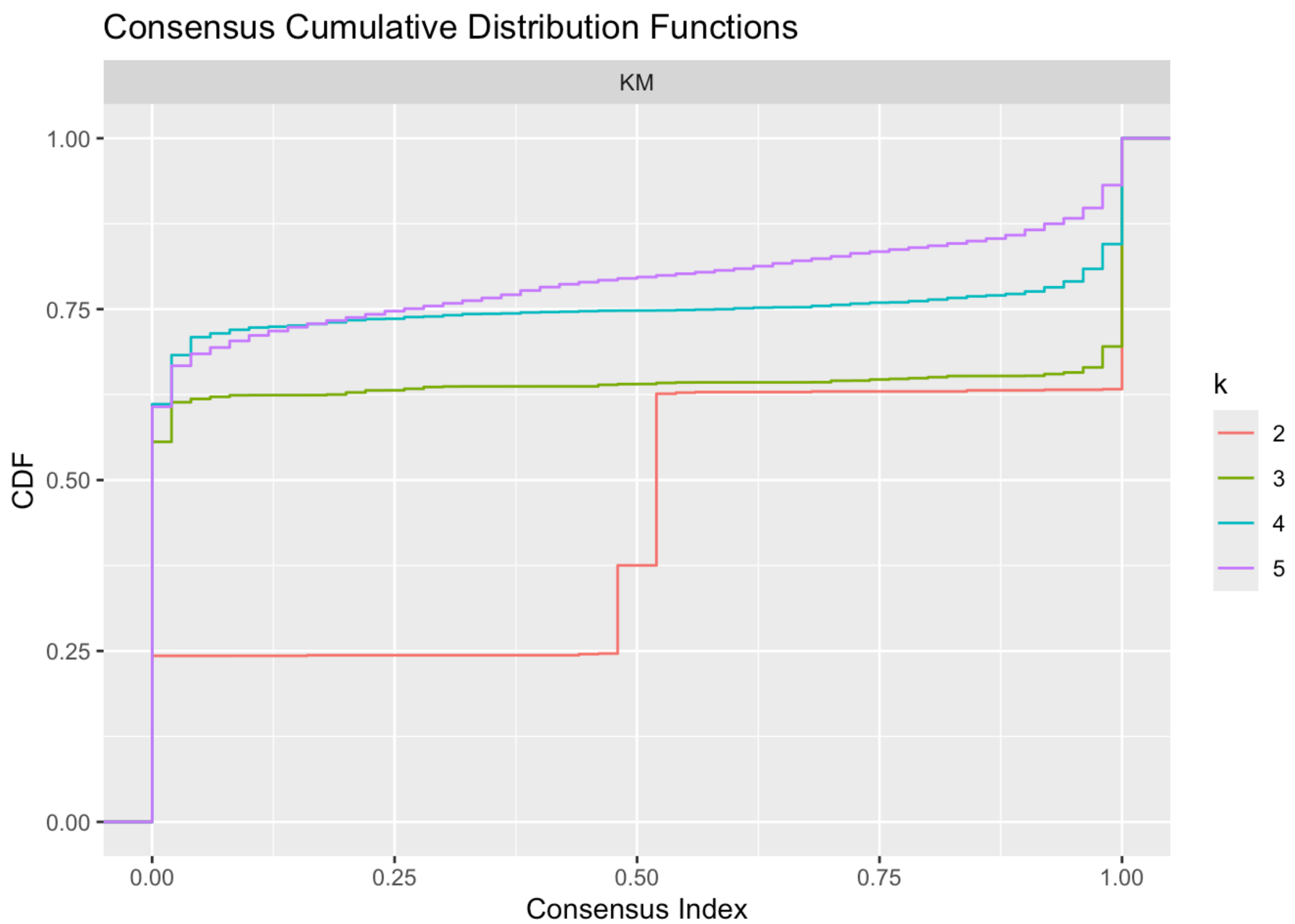

```
delta<-diceR::graph_delta_area(cm_array)
```

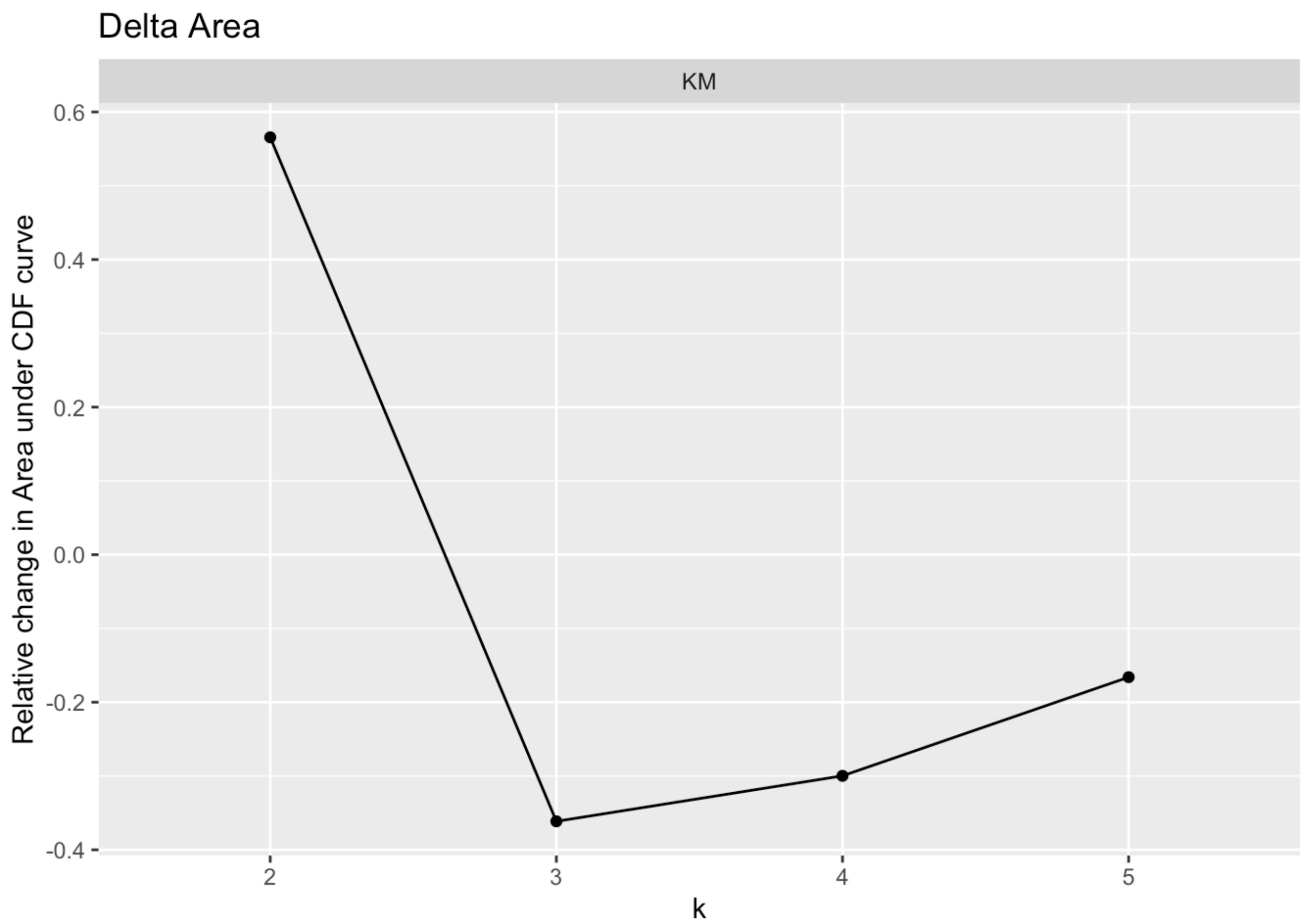

`

Distribution of ARIs

Distributions of ARIs are plotted for both inner and outer loop.

```
# Extract ARI results
ari_plot <- map_df(
    names(ensemble_cv),
    ~ tibble(
        k = str_remove(.x, "k_") %>% as.numeric(),
        `Inner loop` = ensemble_cv[[.x]]$inner_ari_mean,
        `Outer loop` = ensemble_cv[[.x]]$outer_ari_mean,
        Inner_sd = ensemble_cv[[.x]]$inner_ari_sd,
        Outer_sd = ensemble_cv[[.x]]$outer_ari_sd  )) %>%
    pivot_longer(
        cols = c(`Inner loop`, `Outer loop`),
        names_to = "Type",
        values_to = "ARI"
    ) %>%
    mutate(
        SD = ifelse(
            Type == "Inner loop",
            Inner_sd,
            Outer_sd
        )
    )

# plot AIRs
pd <- position_dodge(width = 0.2)
ggplot(ari_plot, aes( x = k, y = ARI,  color = Type, group = Type )) +
    geom_line(linewidth = 1,position = pd) +
    geom_point(size = 3,position = pd) +
    geom_errorbar( aes( ymin = ARI - SD, ymax = ARI + SD),
        position = pd,
        width = 0.15,
        linewidth = 0.8) +
    scale_x_continuous(  breaks = ari_plot$k %>% unique()) +
    scale_color_manual( values = c( "Inner loop" = "#67A9CF", "Outer loop" = "#2
166AC" )) +
    theme_classic() +
    labs(
        x = "Number of clusters (k)",
        y = "ARI",
        color = NULL
    )
```

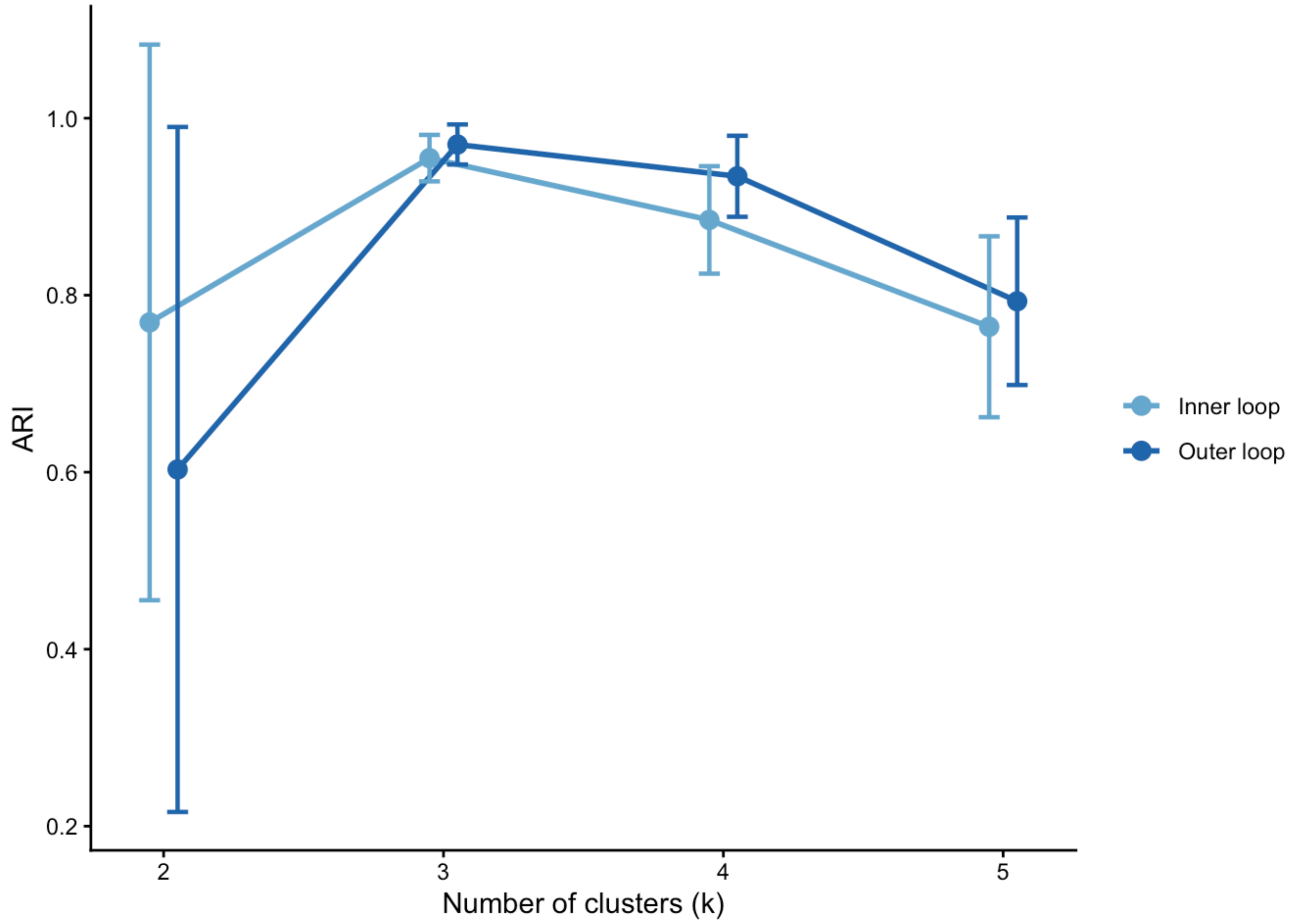


```
# Comparing with true label
table(dta_group$group,ensemble_cv$k_3$cluster)
```

```
##
##            1   2   3
##   Group1 278   0  20
##   Group2   0   0 150
##   Group3   0 148   0
```

Example: obtaining clustering results of k=2-5 using hierarchical clutering as BC.

```
ensemble_cv<-run_ensemble_cv(dta, model= "hc", npc = 5, seed = 1111)
```

Plot co-association matrix

```
plot_cm(ensemble_cv, 2)
```

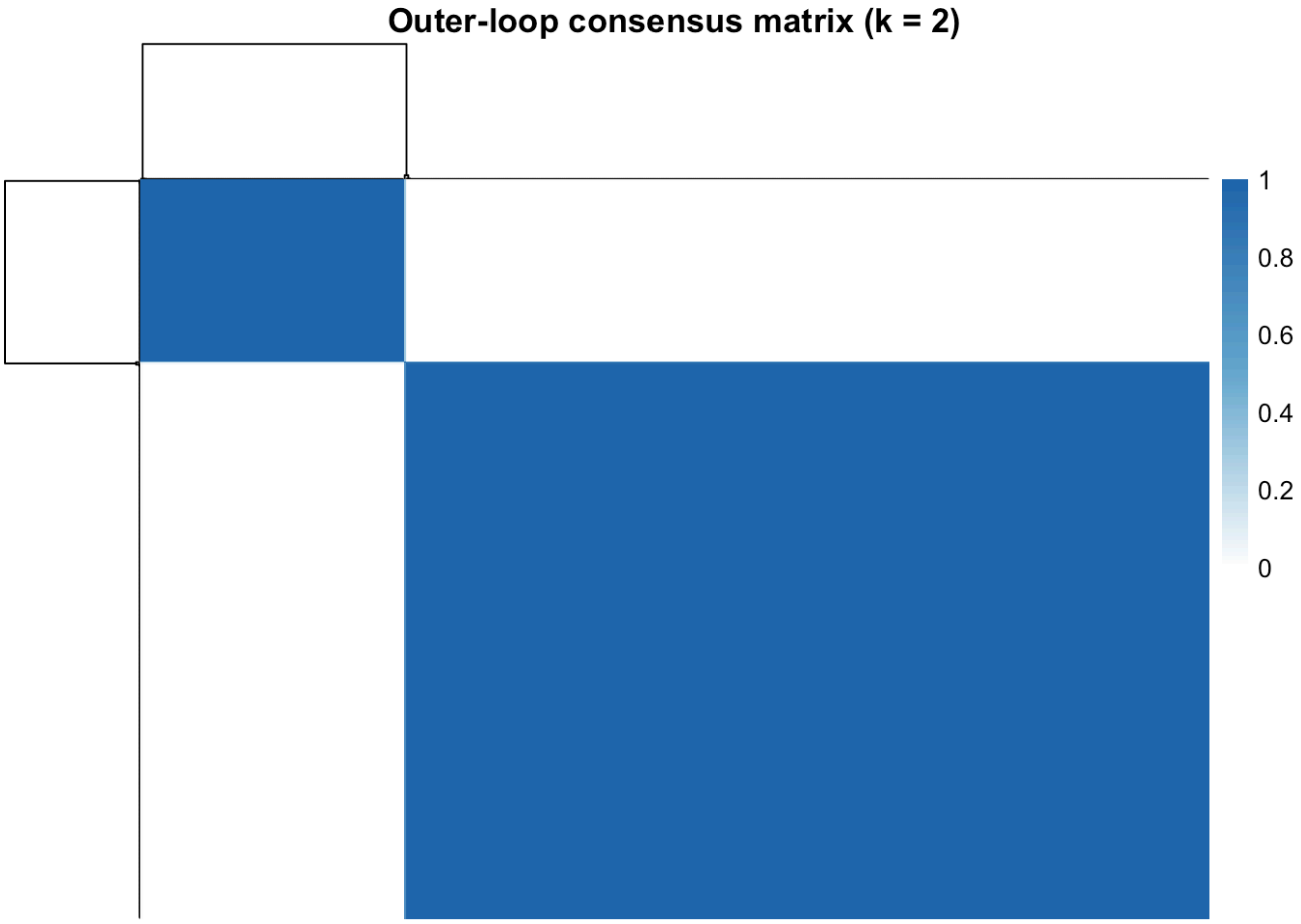


```
plot_cm(ensemble_cv, 3)
```

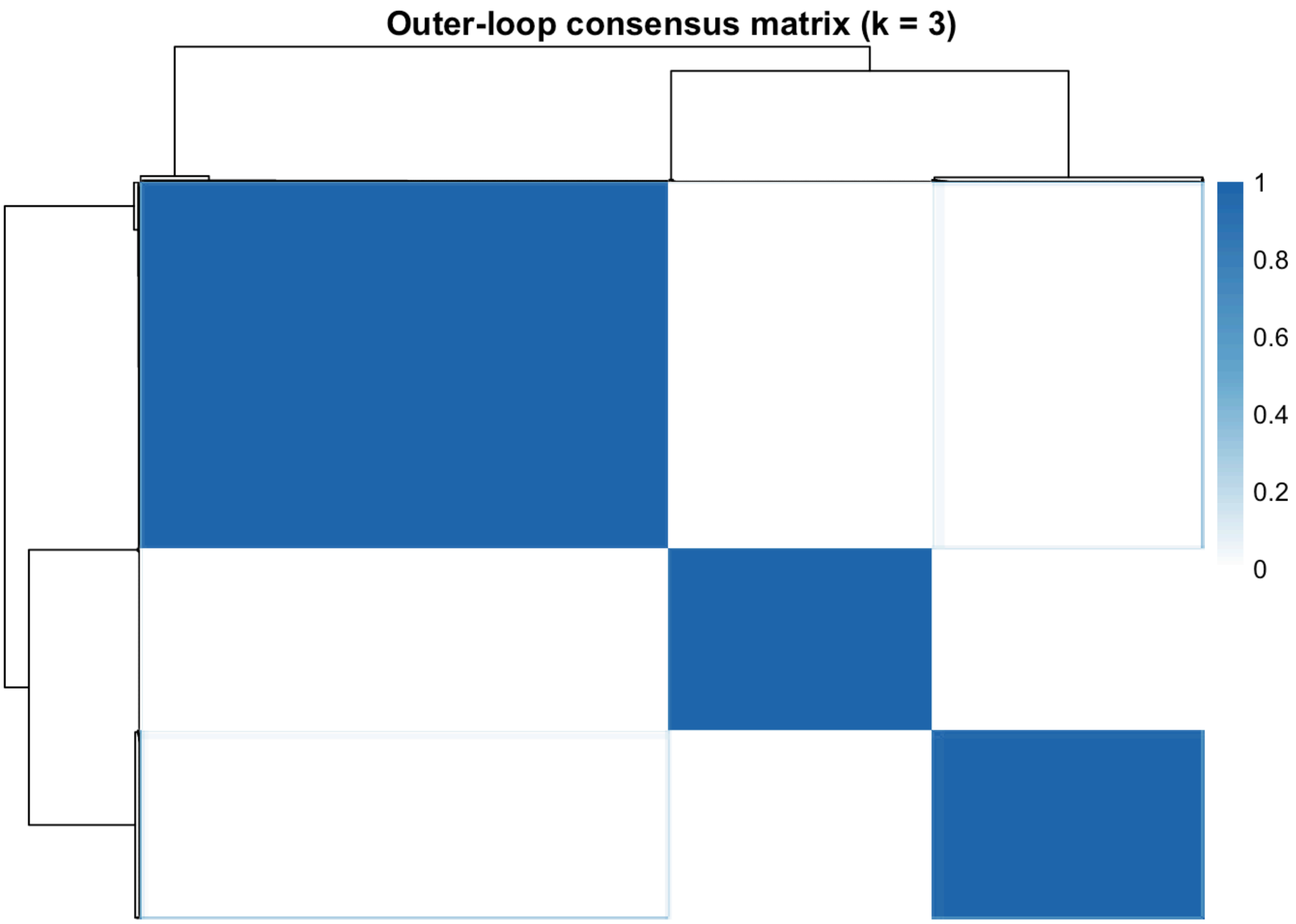


```
plot_cm(ensemble_cv, 4)
```

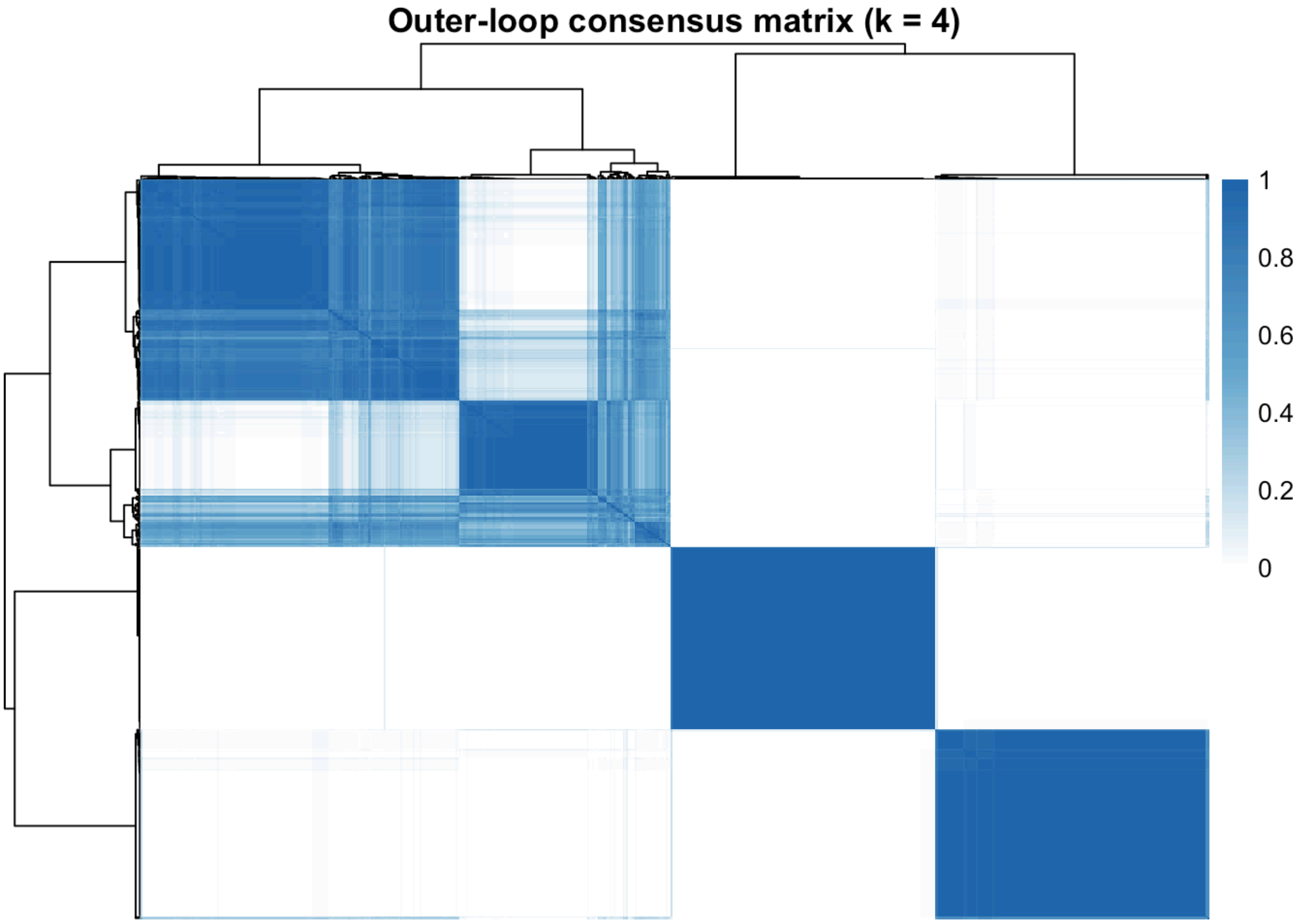


```
plot_cm(ensemble_cv, 5)
```

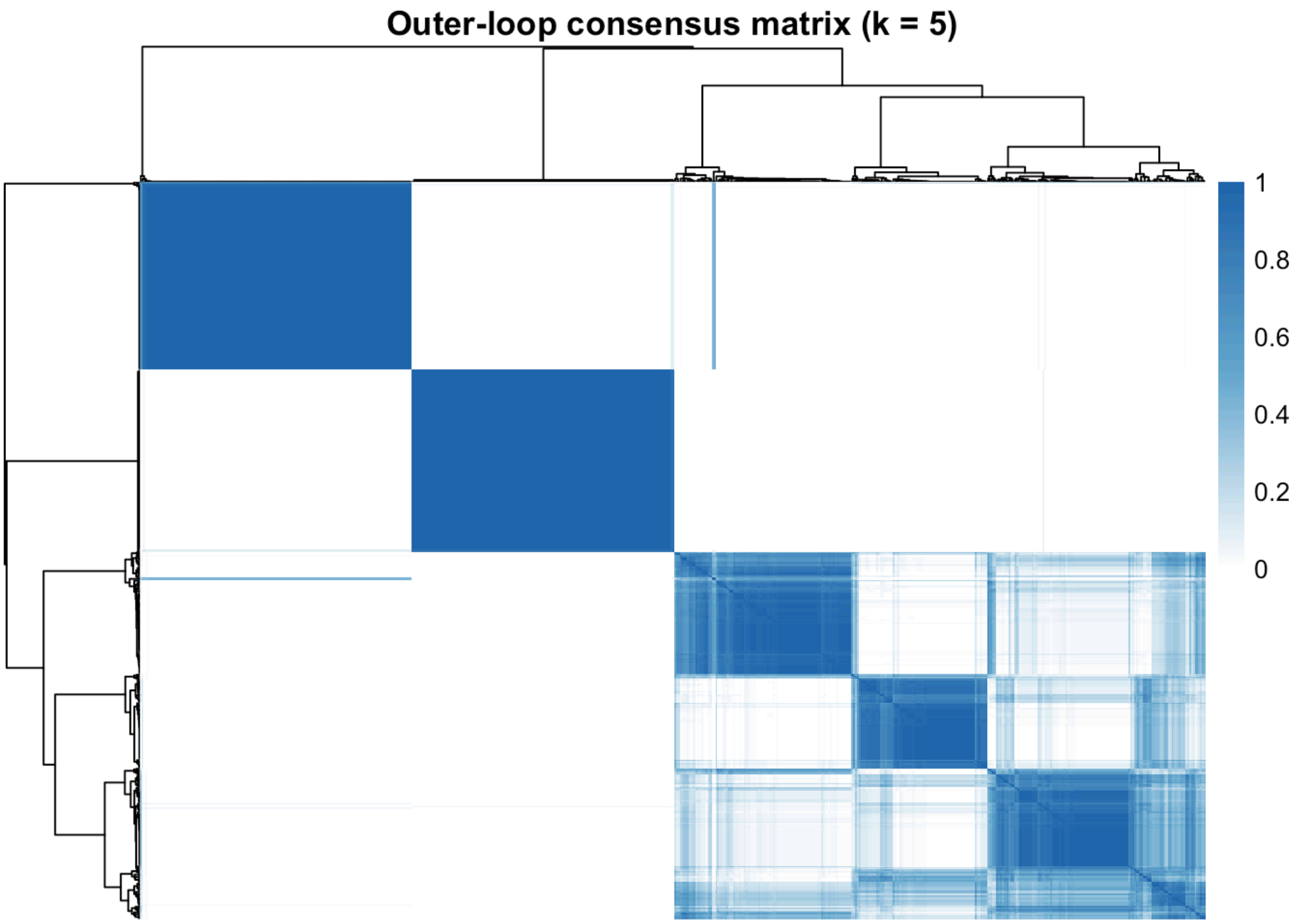

CDF and Delta plot

CDF and Delta plot obtained for outerloop.

```
# extract consensus matrices
cms <- lapply(ensemble_cv, function(x) x$bc)
n <- nrow(cms[[1]])
r <- ncol(ensemble_cv[[1]]$bc)

# build fake diceR ensemble array
cm_array <- array(
  NA,
  dim = c(n, r, 1, length(cms)),
  dimnames = list(
    paste0("ID", seq_len(n)),
    paste0("R", seq_len(r)),
    "KM",
    ks_names
  )
)

for(i in seq_along(cms)){
  cm_array[, , 1, i] <- as.matrix(cms[[i]])
}

# run plots
cdf<-diceR::graph_cdf(cm_array)
```

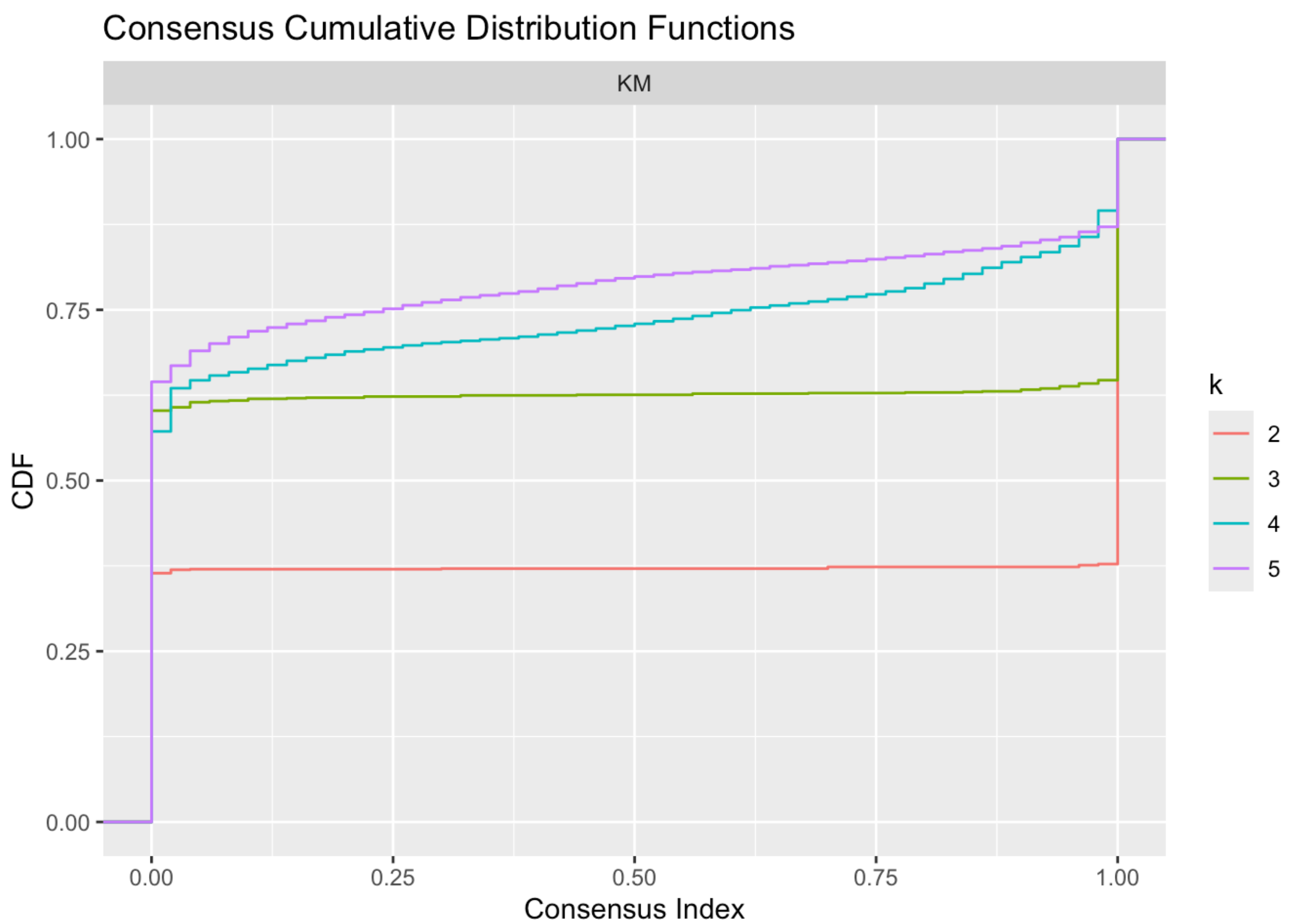


```
delta<-diceR::graph_delta_area(cm_array)
```

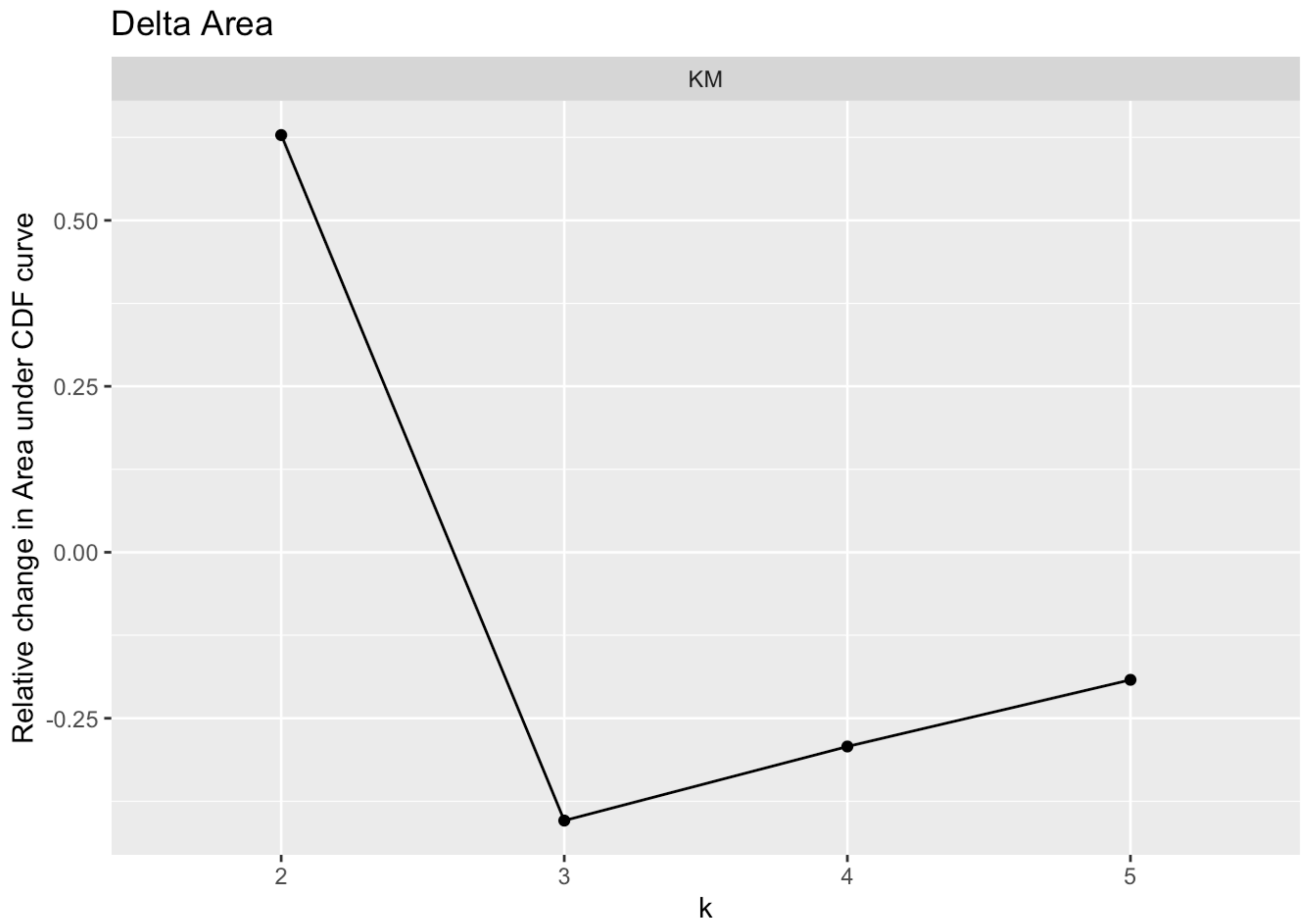

View distribution of ARI

Distributions of ARIs are plotted for both inner and outer loop.

```
# Extract ARI results
ari_plot <- map_df(
    names(ensemble_cv),
    ~ tibble(
        k = str_remove(.x, "k_") %>% as.numeric(),
        `Inner loop` = ensemble_cv[[.x]]$inner_ari_mean,
        `Outer loop` = ensemble_cv[[.x]]$outer_ari_mean,
        Inner_sd = ensemble_cv[[.x]]$inner_ari_sd,
        Outer_sd = ensemble_cv[[.x]]$outer_ari_sd  )) %>%
    pivot_longer(
        cols = c(`Inner loop`, `Outer loop`),
        names_to = "Type",
        values_to = "ARI"
    ) %>%
    mutate(
        SD = ifelse(
            Type == "Inner loop",
            Inner_sd,
            Outer_sd
        )
    )

# plot AIRs
pd <- position_dodge(width = 0.2)
ggplot(ari_plot, aes( x = k, y = ARI,  color = Type, group = Type )) +
    geom_line(linewidth = 1,position = pd) +
    geom_point(size = 3,position = pd) +
    geom_errorbar( aes( ymin = ARI - SD, ymax = ARI + SD),
        position = pd,
        width = 0.15,
        linewidth = 0.8) +
    scale_x_continuous(  breaks = ari_plot$k %>% unique()) +
    scale_color_manual( values = c( "Inner loop" = "#67A9CF", "Outer loop" = "#2
166AC" )) +
    theme_classic() +
    labs(
        x = "Number of clusters (k)",
        y = "ARI",
        color = NULL
    )
```

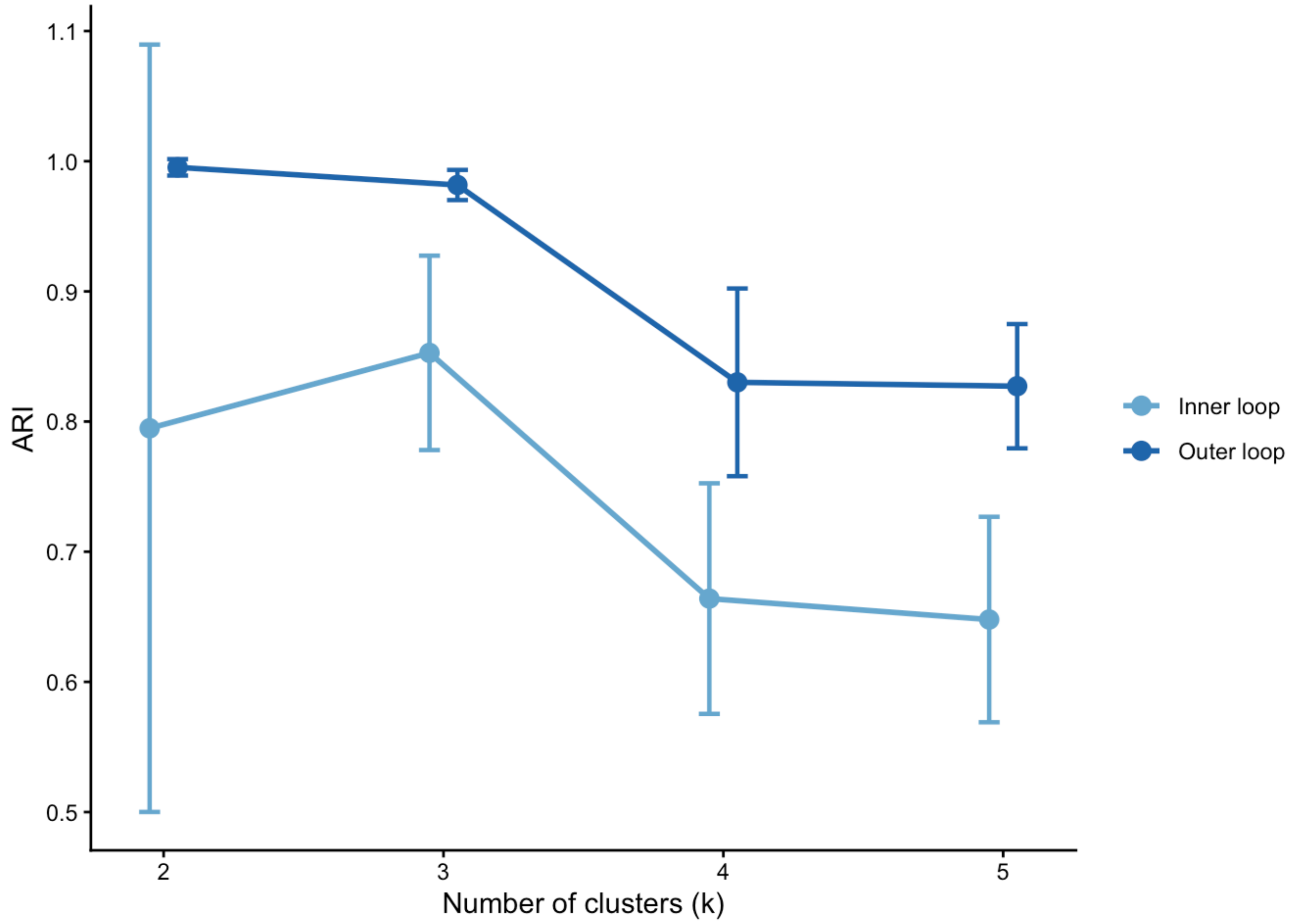


```
# Comparing with true label
table(dta_group$group,ensemble_cv$k_3$cluster)
```

```
##
##             1   2   3
##   Group1 295   0   3
##   Group2   0   0 150
##   Group3   0 147   1
```

Example: obtaining clustering results of k=2-5 using mixed k-means and hierarchical clutering as BC.

```
ensemble_cv<-run_ensemble_cv(dta, model= "mixed", npc = 5, seed = 1111)
```

Plot co-association matrix

```
plot_cm(ensemble_cv, 2)
```

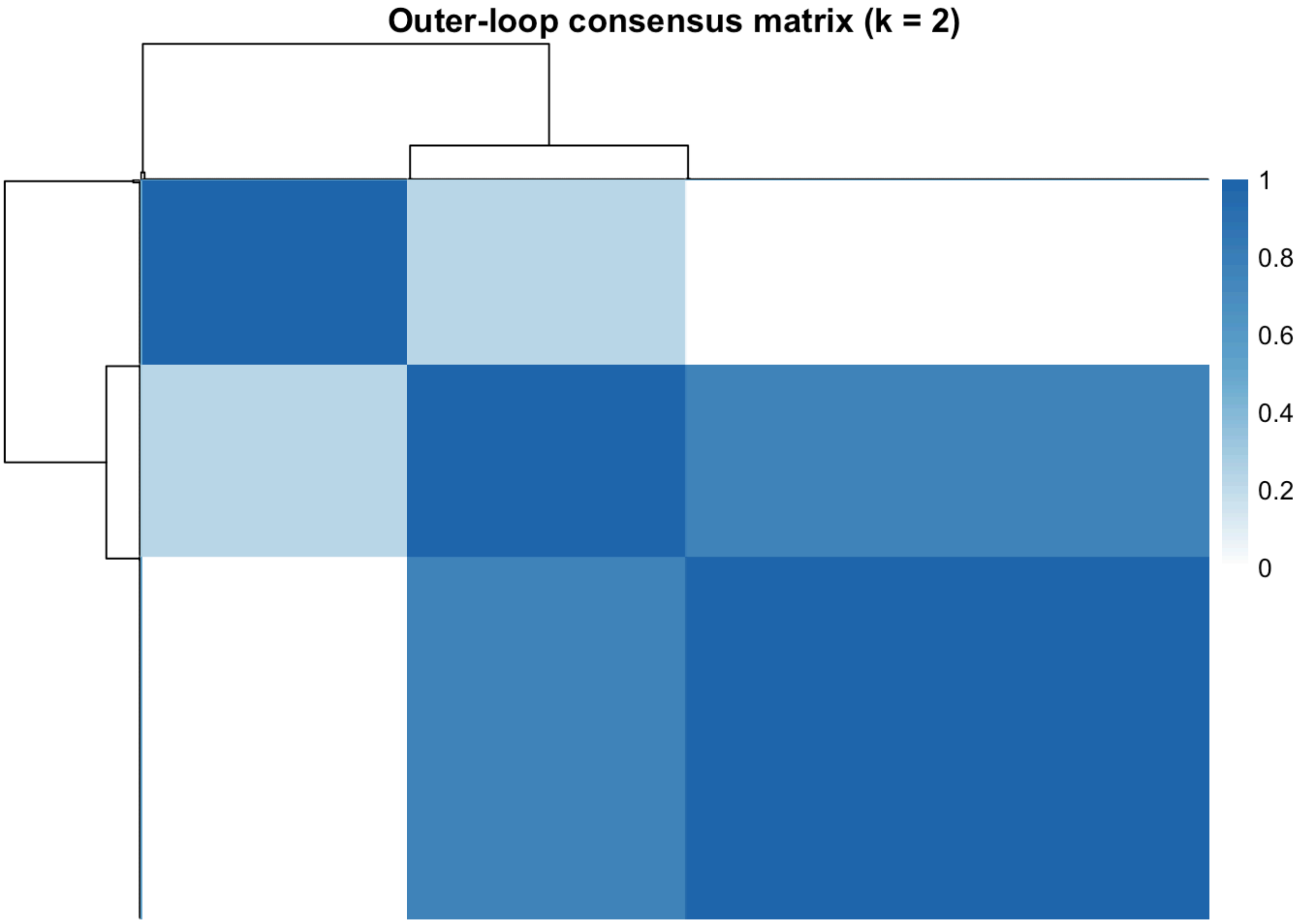


```
plot_cm(ensemble_cv, 3)
```

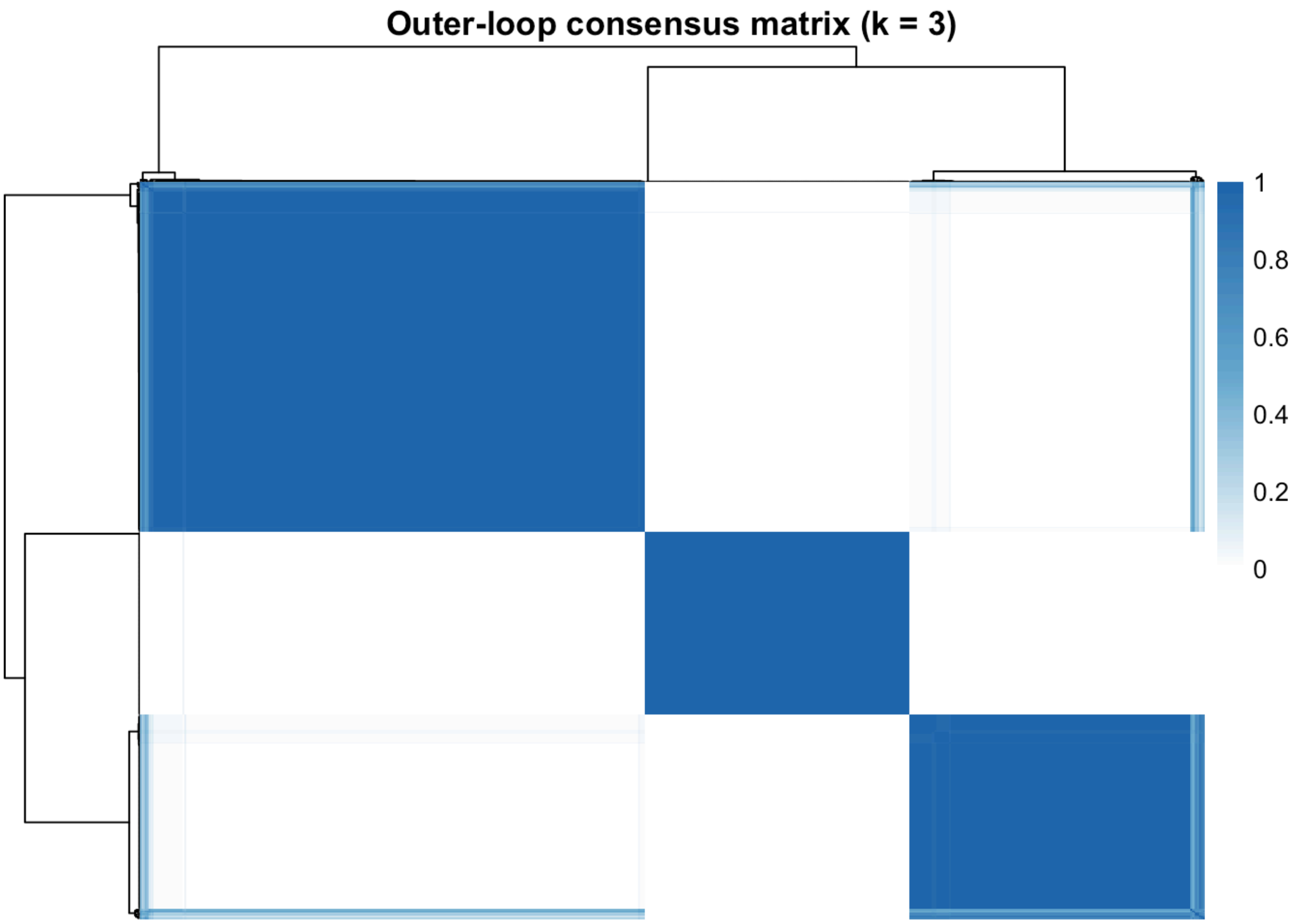


```
plot_cm(ensemble_cv, 4)
```

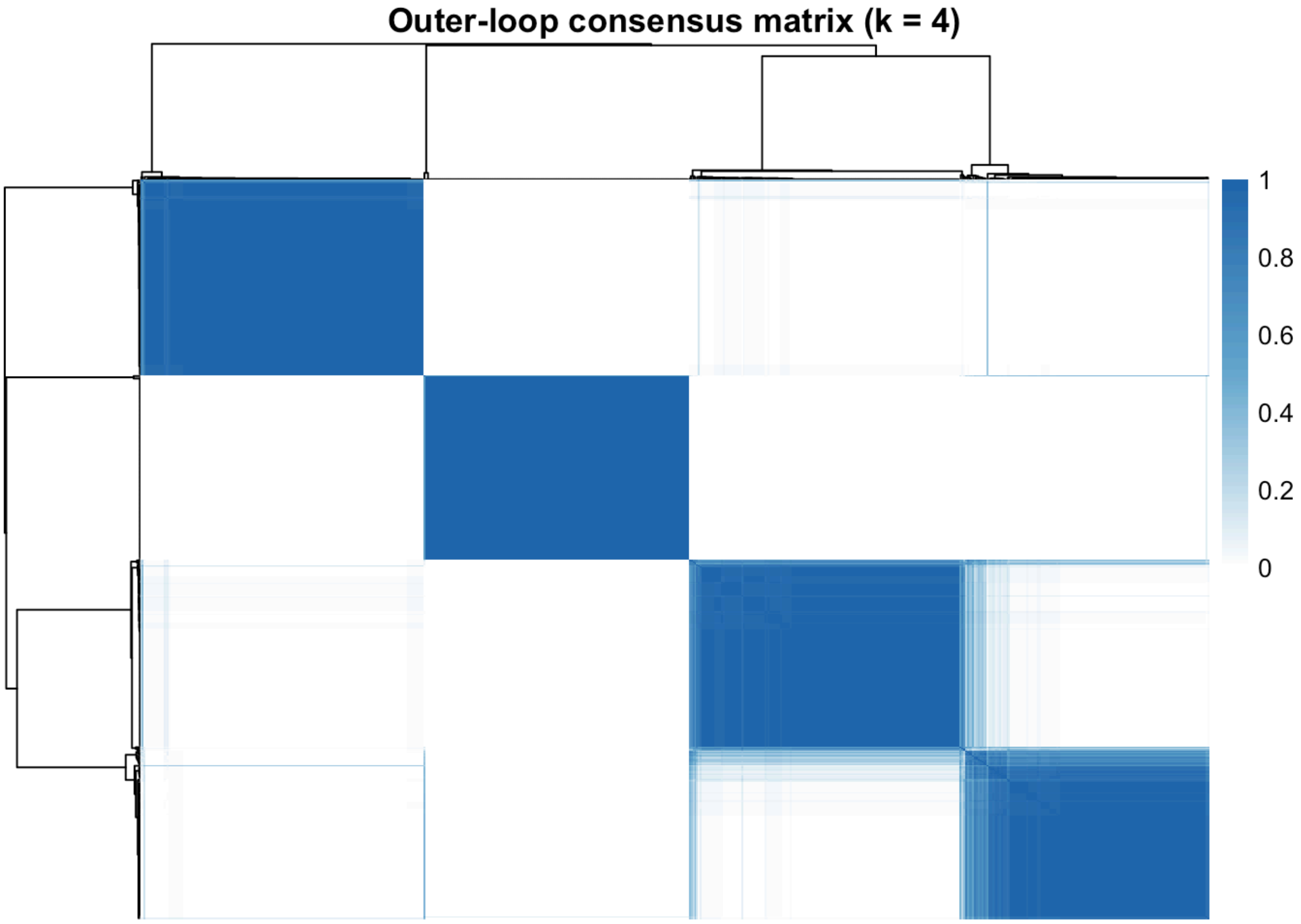


```
plot_cm(ensemble_cv, 5)
```

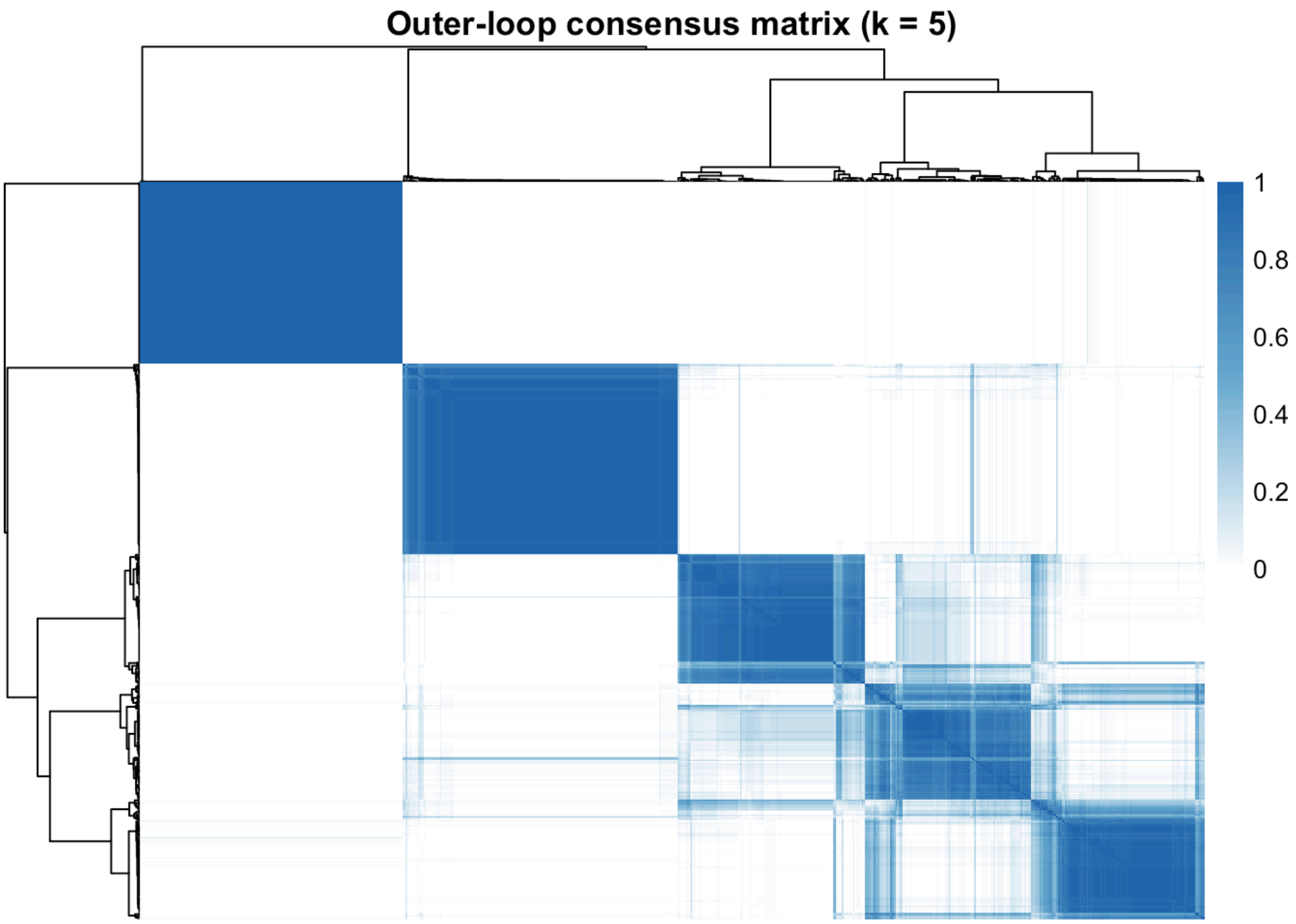

CDF and Delta plot

CDF and Delta plot obtained for outerloop.

```
# extract consensus matrices
cms <- lapply(ensemble_cv, function(x) x$bc)
n <- nrow(cms[[1]])
r <- ncol(ensemble_cv[[1]]$bc)

# build fake diceR ensemble array
cm_array <- array(
  NA,
  dim = c(n, r, 1, length(cms)),
  dimnames = list(
    paste0("ID", seq_len(n)),
    paste0("R", seq_len(r)),
    "KM",
    ks_names
  )
)

for(i in seq_along(cms)){
  cm_array[, , 1, i] <- as.matrix(cms[[i]])
}

# run plots
cdf<-diceR::graph_cdf(cm_array)
```

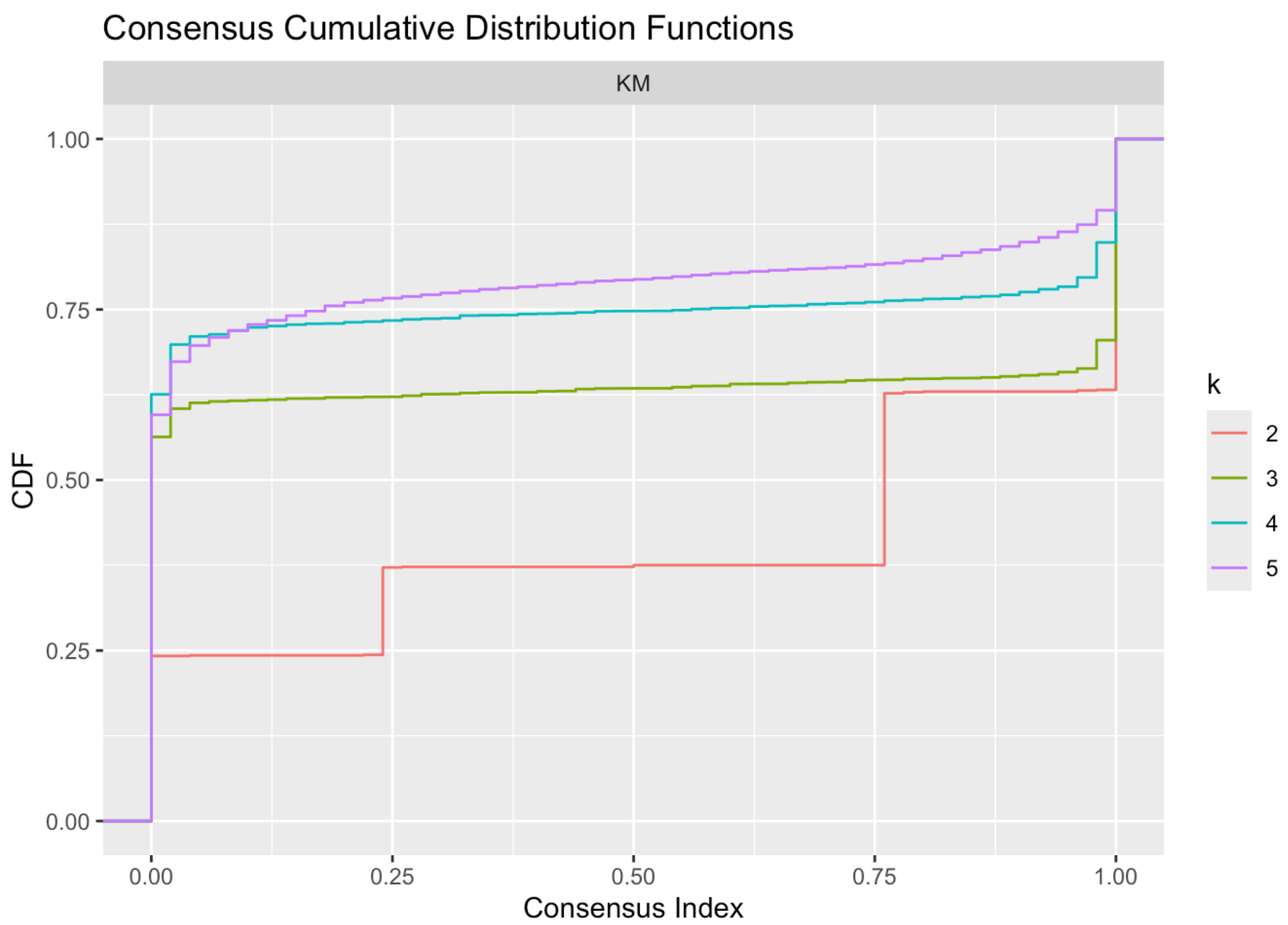


```
delta<-diceR::graph_delta_area(cm_array)
```

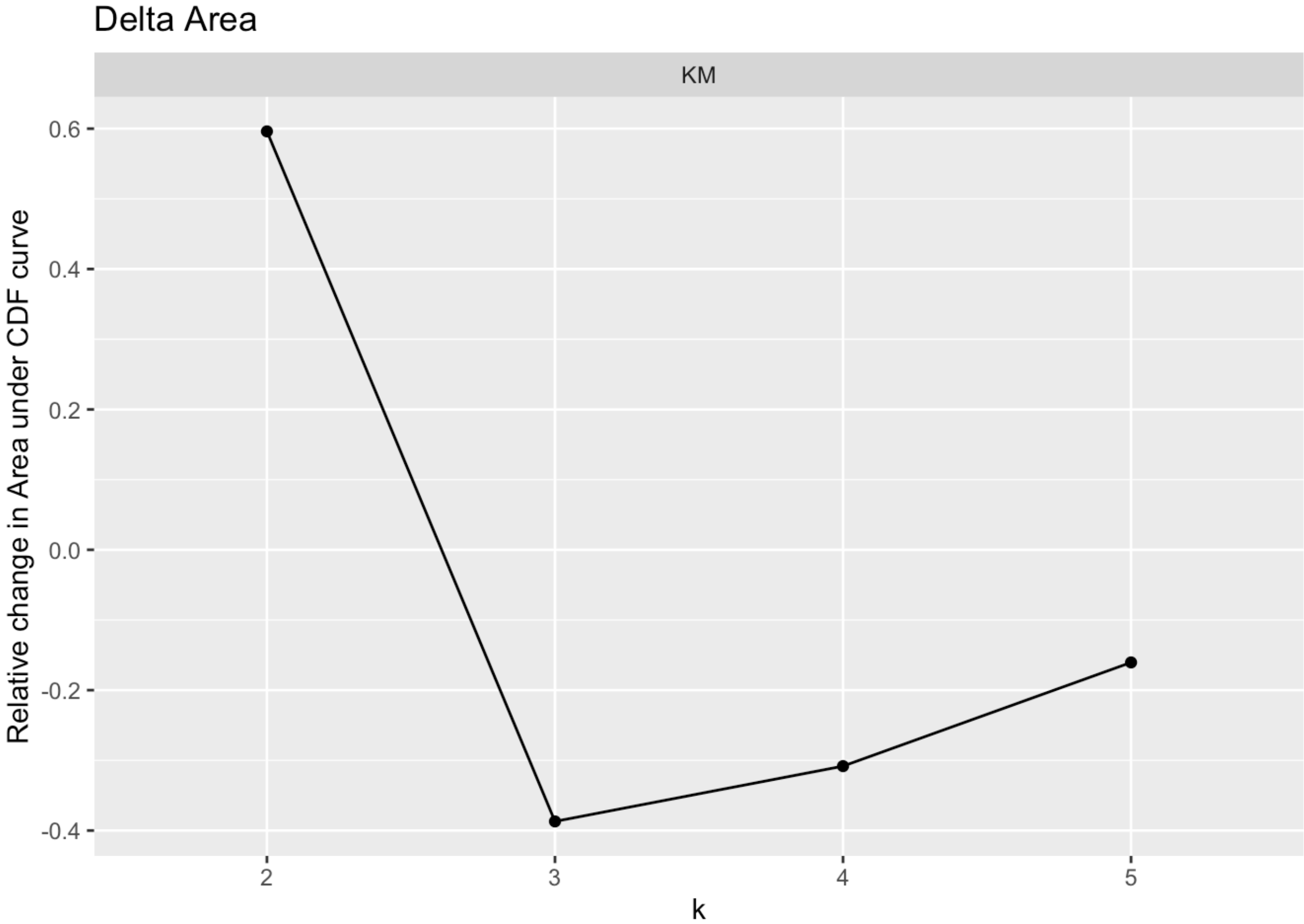

View distribution of ARI

Distributions of ARIs are plotted for both inner and outer loop.

```
# Extract ARI results
ari_plot <- map_df(
    names(ensemble_cv),
    ~ tibble(
        k = str_remove(.x, "k_") %>% as.numeric(),
        `Inner loop` = ensemble_cv[[.x]]$inner_ari_mean,
        `Outer loop` = ensemble_cv[[.x]]$outer_ari_mean,
        Inner_sd = ensemble_cv[[.x]]$inner_ari_sd,
        Outer_sd = ensemble_cv[[.x]]$outer_ari_sd  )) %>%
    pivot_longer(
        cols = c(`Inner loop`, `Outer loop`),
        names_to = "Type",
        values_to = "ARI"
    ) %>%
    mutate(
        SD = ifelse(
            Type == "Inner loop",
            Inner_sd,
            Outer_sd
        )
    )

# plot AIRs
pd <- position_dodge(width = 0.2)
ggplot(ari_plot, aes( x = k, y = ARI,  color = Type, group = Type )) +
    geom_line(linewidth = 1,position = pd) +
    geom_point(size = 3,position = pd) +
    geom_errorbar( aes( ymin = ARI - SD, ymax = ARI + SD),
        position = pd,
        width = 0.15,
        linewidth = 0.8) +
    scale_x_continuous(  breaks = ari_plot$k %>% unique()) +
    scale_color_manual( values = c( "Inner loop" = "#67A9CF", "Outer loop" = "#2
166AC" )) +
    theme_classic() +
    labs(
        x = "Number of clusters (k)",
        y = "ARI",
        color = NULL
    )
```

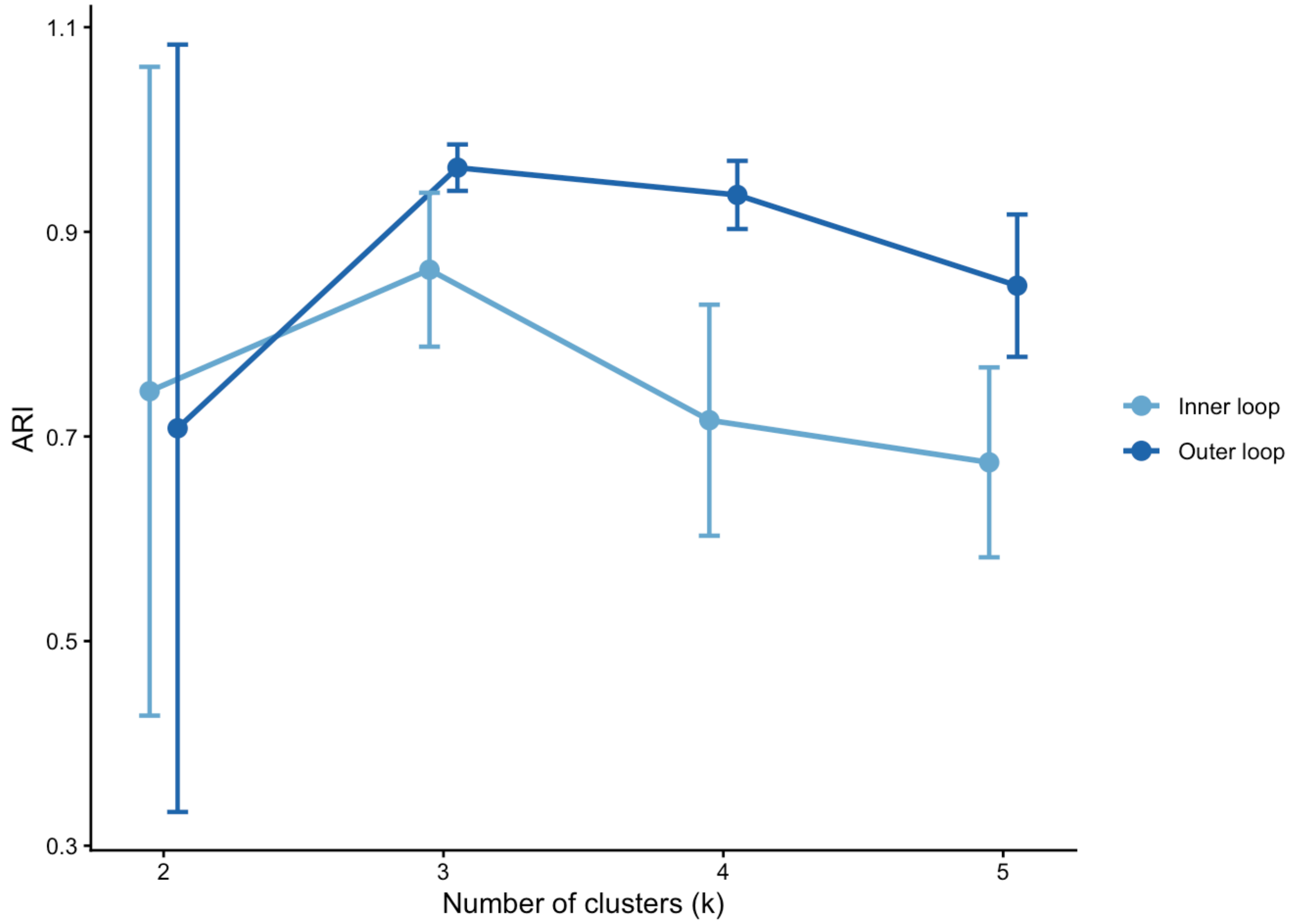


```
# Comparing with true label
table(dta_group$group,ensemble_cv$k_3$cluster)
```

```
##
##            1   2   3
##   Group1 278   0  20
##   Group2   0   0 150
##   Group3   0 148   0
```
