# Supplementary material for: Ensemble clustering: A practical tutorial
Source: Behav Res Methods. 2026 Sep 3;58(10):286. doi: 10.3758/s13428-026-03158-y (PMC13541929; doi:10.3758/s13428-026-03158-y)

Functions
random_missing
gen_data
extract_PCA
cluster_kmeans, cluster_hc, and cluster_lpa
cluster_cc
combine_cluster
extract_results
plot_results
Source data
Data Frame Summary
Data Frame Summary
Data Frame Summary
Generate simulated data
Simulation results

Ensemble clustering: A practical tutorial

Supplementary Material V: Simulation results

```
library(pacman)
p_load("tidyverse", "ggplot2", "gridExtra", "kableExtra", "knitr",
       "mice", "here", "ggthemes", "scales", "FactoMineR","diceR",
       "purrr", "mclust", "parallel","patchwork","factoextra")
## Provides detailed information and environment settings for the current R sess
ion
sessionInfo()
```

```
## R version 4.5.1 (2025-06-13)
## Platform: aarch64-apple-darwin20
## Running under: macOS Tahoe 26.4.1
##
## Matrix products: default
## BLAS:   /Library/Frameworks/R.framework/Versions/4.5-arm64/Resources/lib/libR
blas.0.dylib
## LAPACK: /Library/Frameworks/R.framework/Versions/4.5-arm64/Resources/lib/libR
lapack.dylib;  LAPACK version 3.12.1
##
## locale:
## [1] en_US.UTF-8/en_US.UTF-8/en_US.UTF-8/C/en_US.UTF-8/en_US.UTF-8
##
## time zone: Australia/Melbourne
## tzcode source: internal
##
## attached base packages:
## [1] parallel  stats     graphics  grDevices utils     datasets  methods
## [8] base
##
## other attached packages:
##  [1] factoextra_1.0.7 patchwork_1.3.2  mclust_6.1.2     diceR_3.1.0
##  [5] FactoMineR_2.12  scales_1.4.0     ggthemes_5.1.0   here_1.0.2
##  [9] mice_3.19.0      knitr_1.50       kableExtra_1.4.0 gridExtra_2.3
## [13] lubridate_1.9.4  forcats_1.0.1    stringr_1.5.2    dplyr_1.2.1
## [17] purrr_1.1.0      readr_2.1.5      tidyr_1.3.2      tibble_3.3.0
## [21] ggplot2_4.0.2    tidyverse_2.0.0  pacman_0.5.1
##
## loaded via a namespace (and not attached):
##  [1] Rdpack_2.6.4        sandwich_3.1-1      rlang_1.1.7
##  [4] magrittr_2.0.4      multcomp_1.4-29     compiler_4.5.1
##  [7] systemfonts_1.3.1   vctrs_0.7.1         pkgconfig_2.0.3
## [10] shape_1.4.6.1       fastmap_1.2.0       backports_1.5.0
## [13] rmarkdown_2.30      tzdb_0.5.0          nloptr_2.2.1
## [16] xfun_0.56           glmnet_4.1-10       jomo_2.7-6
## [19] cachem_1.1.0        jsonlite_2.0.0      flashClust_1.01-2
## [22] pan_1.9             broom_1.0.10        cluster_2.1.8.1
## [25] R6_2.6.1            bslib_0.9.0         stringi_1.8.7
## [28] RColorBrewer_1.1-3  boot_1.3-31         rpart_4.1.24
## [31] jquerylib_0.1.4     estimability_1.5.1  Rcpp_1.1.0
## [34] iterators_1.0.14    zoo_1.8-15          Matrix_1.7-3
## [37] splines_4.5.1       nnet_7.3-20         timechange_0.3.0
## [40] tidyselect_1.2.1    rstudioapi_0.17.1   yaml_2.3.10
## [43] codetools_0.2-20    lattice_0.22-7      withr_3.0.2
## [46] S7_0.2.0            coda_0.19-4.1       evaluate_1.0.5
## [49] survival_3.8-3      xml2_1.4.0          pillar_1.11.1
## [52] DT_0.34.0           foreach_1.5.2       reformulas_0.4.1
## [55] generics_0.1.4      rprojroot_2.1.1     hms_1.1.3
## [58] minqa_1.2.8         xtable_1.8-4        leaps_3.2
## [61] glue_1.8.0          emmeans_2.0.0       scatterplot3d_0.3-44
## [64] tools_4.5.1         lme4_1.1-37         mvtnorm_1.3-3
## [67] grid_4.5.1          rbibutils_2.3       nlme_3.1-168
## [70] cli_3.6.5           textshaping_1.0.4   viridisLite_0.4.2
## [73] svglite_2.2.2       gtable_0.3.6        sass_0.4.10
## [76] digest_0.6.37       ggrepel_0.9.6       TH.data_1.1-5
## [79] htmlwidgets_1.6.4   farver_2.1.2        htmltools_0.5.8.1
```

```
## [82] lifecycle_1.0.5      multcompView_0.1-10  mitml_0.4-5
## [85] MASS_7.3-65
```

```
# ensure select and filter function always sourced from dplyr package
conflicted::conflict_prefer("select", "dplyr")
```

```
## [conflicted] Will prefer dplyr::select over any other package.
```

```
conflicted::conflict_prefer("filter", "dplyr")
```

```
## [conflicted] Will prefer dplyr::filter over any other package.
```

Functions

random_missing

Data will be randomly selected and removed across all variables with a pre-specified proportion

```
# Function generate missing data
random_missing <- function(x, p) {
    # x=data; p=percentage missing
    x[sample(1:length(x), round(length(x) * p), replace = FALSE)] <- NA
    return(x)
}
```

gen_data

For demonstrating clustering results, this function will generated six lists of datasets with increasing level of perturbation:

- m imputed data sets with 0.25% of data randomly removed from source data
- m imputed data sets with 1% of data randomly removed from source data
- m imputed data sets with 2.5% of data randomly removed from source data
- m imputed data sets with 5% of data randomly removed from source data
- m imputed data sets with 10% of data randomly removed from source data
- m imputed data sets with 20% of data randomly removed from source data

Multiple Imputation by Chained Equations (MICE) will be used to capture multivariate associations with source data.

```
gen_data <- function(data, m, seed = 1234) {
    # Define the list
    combined_dta <- list()

    #generate missing 0.25, 1, 2.5, 5, 10, 20 %
    set.seed(1234)
    data_missing <- list()
    percent = c(0.0025, 0.01, 0.025, 0.05, 0.10, 0.20)
    for (i in 1:length(percent)) {
        set.seed(12457643)
        data_missing[[i]] <- data %>%
            mutate_all(random_missing, percent[i])
    }
    # Generate m imputed data for each level of missingness
    for (i in 1:length(percent)) {
        impute_data_ind <- mice(data_missing[[i]], m = m, method = "pmm")
        impute_data_ind <- complete(impute_data_ind, "all")
        name <- paste(percent[i] * 100, "% missing")
        combined_dta[[name]] <- impute_data_ind
    }
    combined_dta
}
```

extract_PCA

Extract pre-specified components and scale all the extracted variables

```
# conduct PCA
extract_PCA <- function(data, c = 5) {
    data <- data %>% mutate_all(as.numeric)
    PCA <- prcomp(data, center = TRUE, scale. = TRUE)
    data <- as_tibble(PCA$x[, 1:c]) %>%
        mutate_all( ~ as.numeric(scale(.)))
}
```

cluster_kmeans, cluster_hc, and cluster_lpa

```
# K-means
cluster_kmeans <- function(data, k = 2, c = 5) {
    kmeans(data, k, iter.max = 40, nstart = 20)$cluster
}

# Hierarchical clustering
cluster_hc <- function(data, k = 2, c = 5) {
    cluster <- hclust(dist(data), method = "ward.D2")
    cutree(cluster, k)
}

# LPA
cluster_lpa <- function(data, k = 2, c = 5) {
    mclust::Mclust(data, k, modelNames = "VVV")$classification
}
```

cluster_cc

Self-defined ensemble clustering function involves randomly sample variables to save computational cost. When both observations and/or variables were sampled, imputation models were often required to fill missing values of consensus results, which increases computational cost. In this self-defined function, we first estimate BCs using function gen_base_clustering, which sample 80% of variables. For each BC data, we conduct PCA followed by k-means clustering.

```
# Ensemble clustering, use self-defined function that samples variables to avoid
imputation to speed up simulation process.
cluster_cc <- function(data, k = 2, c = 5) {
    BC <- gen_base_clustering(data, k, c, reps = 100)
    diceR::k_modes(as.matrix(BC), is.relabelled = FALSE)

}

gen_base_clustering <- function(data, k = 2, c = 5, reps) {
    # data structure to store the results
    BCs <- as.data.frame(1:nrow(data))
    # loop individual BCs
    for (i in 1:reps) {
        # Sample variables
        sample_cols <-
            sample(1:ncol((data)), size = round(ncol(data) * 0.8))
        sample_data <- data[, sample_cols] %>%  mutate_all(as.numeric)
        # dimensionality reduction
        PCA <- prcomp(sample_data , center = TRUE, scale. = TRUE)
        PCA <- as_tibble(PCA$x[, 1:c]) %>% mutate_all( ~ as.numeric(scale(.)))
        # K-means clustering with 3 clusters
        model <- kmeans(PCA,  centers = k, nstart = 10)
        # Join BC back to results
        BCs[[i]] <- model$cluster
    }
    #remove ID
    BCs <- BCs %>%
        mutate_all(as.numeric)
}
```

combine_cluster

This function will obtain final clustering results (mean and SD of Rand Index) using a given model across all the datasets with a given level of perturbation (e.g., 100 imputed datasets from data with 1% of missing). The function will fist obtain clustering results of each dataset, combine them into a dataframe and calculate all pairwise RI. The function will return the mean and SD of RI.

```
# function integrate results
combine_cluster <- function(data, type = "cluster_kmeans", k = 2, c = 5) {
    cluster <- purrr::map(data, ~ parallel::mclapply(.x, type, k, c, mc.cores = 
4))
    cluster <- lapply(cluster, function(x)
            as.data.frame(do.call(cbind, x)))
    cluster_RI <- parallel::mclapply(cluster, RI, mc.cores = 4)
    cluster_RI <- do.call(rbind, cluster_RI)
    cluster_RI <- as.data.frame(cluster_RI) %>%
        mutate(data = rownames(cluster_RI))  %>%
        rename(RI_mean = V1, RI_SD = V2)
    cluster_RI
}

# Function calculating RI for all pairs of columns in the data
cal_RI_ij <- function(i, j, data) {
    return <- 1
    if (i != j) {
        data <- data[, c(i, j)] %>% na.omit()
        return <- aricode::RI(data[, 1], data[, 2])
    }
    return
}

RI <- function(cluster_results) {
    # Calculate pairwise Rand index
    cluster_results <- as.data.frame(cluster_results)
    cal_RI <- Vectorize(cal_RI_ij, vectorize.args = list("i", "j"))
    RI <- outer(1:length(cluster_results),
                1:length(cluster_results),
                cal_RI,
                data = cluster_results)
    RI[lower.tri(RI, diag = TRUE)] <- NA
    c(mean(RI, na.rm = TRUE), sd(RI, na.rm = TRUE))
}
```

Note: parallel::mclapply is only supported by macOS and Unix. fFor Windows, parallelization can be achived using parallel::parLapply() (https://dept.stat.lsa.umich.edu/~jerrick/courses/stat701/notes/parallel.html#using-sockets-with-parlapply)

extract_results

This function integrate all the steps across different models. It will repeat all the clustering models across perturbated data for each modelling method including, k-means, hierarchical clustering, LPA and ensemble clustering.

```
extract_results <- function(data, k, c) {
    # PCA
    PCA <- purrr::map (data, ~ lapply(.x, extract_PCA, c))

    # K-means results
    km <- combine_cluster(PCA, "cluster_kmeans", k)  %>%
        mutate(Model = "K-means")

    # Hierarchical results
    hc <- combine_cluster(PCA, "cluster_hc", k) %>%
        mutate(Model = "Hierarchical")

    # LPA results
    lpa <- combine_cluster(PCA, "cluster_lpa", k) %>%
        mutate(Model = "LPA")

    # Ensemble clustering results
    cc <- combine_cluster(data, "cluster_cc", k, c) %>%
        mutate(Model = "Ensemble clustering")

    # Combine and return
    as.data.frame(rbind(km, hc, lpa, cc))
}
```

plot_results

This function will plot the results.

```
plot_results <- function(results, base) {
  results %>%
    mutate(
      data = factor(
        data,
        levels = c(
          "Rerun",
          "0.25 % missing",
          "1 % missing",
          "2.5 % missing",
          "5 % missing",
          "10 % missing",
          "20 % missing"
        )
      ),
      Model = factor(
        Model,
        levels = c(
          "Hierarchical",
          "K-means",
          "LPA",
          "Ensemble clustering"
        )
      )
    ) %>%
    ggplot(aes(
      x = data,
      y = RI_mean,
      color = Model,
      group = Model
    )) +
    geom_line(position = position_dodge2(width = 0.2)) +
    geom_errorbar(
      aes(ymin = RI_mean - RI_SD, ymax = RI_mean + RI_SD),
      width = 0.2,
      position = position_dodge2(width = 0.2)
    ) +
    theme_classic() +
    geom_hline(yintercept = base, linetype = "dashed") +
    theme(
      strip.background = element_blank(),
      panel.border = element_rect(colour = "black", fill = NA)
    ) +
    scale_color_manual(values = c("#A9A9A9", "#ADDAFB", "#ACCCD7", "#5998AD")) +
    labs(x = "Missing data", y = "RI", color = "Model")
}
```

Source data

Here we use three datasets

```
dta1 <- readRDS(here::here("AQOL.rds")) %>%
    select(Q1:Q20) %>%
    mutate_all(as.numeric)
dta2 <- readRDS(here::here("Alexithymia.rds"))
dta3 <- readRDS(here::here("Cannabis.rds"))
```

```
print(summarytools::dfSummary(dta1, graph.magnif = 0.75), method = "render")
```

Data Frame Summary

dta1

Dimensions: 1067 x 20
Duplicates: 1

No	Variable	Stats / Values	Freqs (% of Valid)	Graph	Valid	Missing
1	Q1<br>[numeric]	Mean (sd) : 2 (1.1)<br>min ≤ med ≤ max:<br>1 ≤ 2 ≤ 6<br>IQR (CV) : 1 (0.6)	1 : 398 (37.3%)<br>2 : 425 (39.8%)<br>3 : 181 (17.0%)<br>4 : 18 (1.7%)<br>5 : 7 (0.7%)<br>6 : 38 (3.6%)		1067<br>(100.0%)	0<br>(0.0%)
2	Q2<br>[numeric]	Mean (sd) : 2.3 (1.2)<br>min ≤ med ≤ max:<br>1 ≤ 2 ≤ 6<br>IQR (CV) : 2 (0.5)	1 : 282 (26.4%)<br>2 : 424 (39.7%)<br>3 : 195 (18.3%)<br>4 : 105 (9.8%)<br>5 : 30 (2.8%)<br>6 : 31 (2.9%)		1067<br>(100.0%)	0<br>(0.0%)
3	Q3<br>[numeric]	Mean (sd) : 1.3 (0.6)<br>min ≤ med ≤ max:<br>1 ≤ 1 ≤ 4<br>IQR (CV) : 0 (0.5)	1 : 821 (76.9%)<br>2 : 188 (17.6%)<br>3 : 36 (3.4%)<br>4 : 22 (2.1%)		1067<br>(100.0%)	0<br>(0.0%)
4	Q4<br>[numeric]	Mean (sd) : 1.4 (0.7)<br>min ≤ med ≤ max:<br>1 ≤ 1 ≤ 4<br>IQR (CV) : 1 (0.5)	1 : 712 (66.7%)<br>2 : 250 (23.4%)<br>3 : 94 (8.8%)<br>4 : 11 (1.0%)		1067<br>(100.0%)	0<br>(0.0%)

No	Variable	Stats / Values	Freqs (% of Valid)	Graph	Valid	Missing
5	Q5<br>[numeric]	Mean (sd) : 2 (1)<br>min ≤ med ≤ max:<br>1 ≤ 2 ≤ 5<br>IQR (CV) : 1 (0.5)	1 : 324 (30.4%)<br>2 : 519 (48.6%)<br>3 : 136 (12.7%)<br>4 : 52 (4.9%)<br>5 : 36 (3.4%)	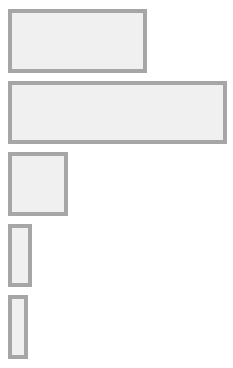	1067<br>(100.0%)	0<br>(0.0%)
6	Q6<br>[numeric]	Mean (sd) : 1.9 (0.8)<br>min ≤ med ≤ max:<br>1 ≤ 2 ≤ 4<br>IQR (CV) : 1 (0.4)	1 : 350 (32.8%)<br>2 : 492 (46.1%)<br>3 : 181 (17.0%)<br>4 : 44 (4.1%)	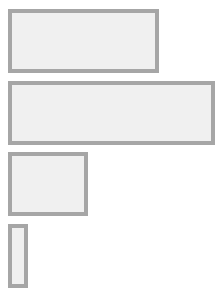	1067<br>(100.0%)	0<br>(0.0%)
7	Q7<br>[numeric]	Mean (sd) : 1.9 (1)<br>min ≤ med ≤ max:<br>1 ≤ 2 ≤ 4<br>IQR (CV) : 1 (0.5)	1 : 499 (46.8%)<br>2 : 308 (28.9%)<br>3 : 120 (11.2%)<br>4 : 140 (13.1%)	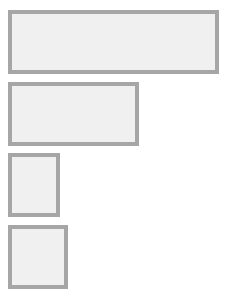	1067<br>(100.0%)	0<br>(0.0%)
8	Q8<br>[numeric]	Mean (sd) : 2.7 (1.2)<br>min ≤ med ≤ max:<br>1 ≤ 3 ≤ 5<br>IQR (CV) : 2 (0.5)	1 : 204 (19.1%)<br>2 : 308 (28.9%)<br>3 : 225 (21.1%)<br>4 : 241 (22.6%)<br>5 : 89 (8.3%)	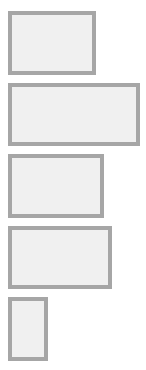	1067<br>(100.0%)	0<br>(0.0%)
9	Q9<br>[numeric]	Mean (sd) : 3.2 (1.2)<br>min ≤ med ≤ max:<br>1 ≤ 3 ≤ 5<br>IQR (CV) : 2 (0.4)	1 : 90 (8.4%)<br>2 : 243 (22.8%)<br>3 : 215 (20.1%)<br>4 : 373 (35.0%)<br>5 : 146 (13.7%)	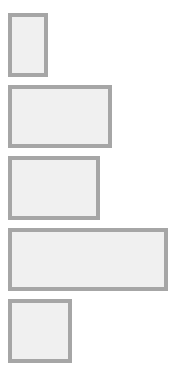	1067<br>(100.0%)	0<br>(0.0%)
10	Q10<br>[numeric]	Mean (sd) : 3.3 (1)<br>min ≤ med ≤ max:<br>1 ≤ 3 ≤ 5<br>IQR (CV) : 1 (0.3)	1 : 53 (5.0%)<br>2 : 177 (16.6%)<br>3 : 416 (39.0%)<br>4 : 277 (26.0%)<br>5 : 144 (13.5%)	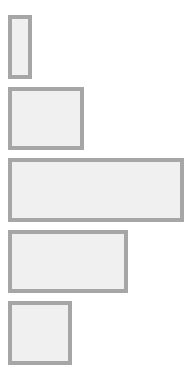	1067<br>(100.0%)	0<br>(0.0%)

No	Variable	Stats / Values	Freqs (% of Valid)	Graph	Valid	Missing
11	Q11<br>[numeric]	Mean (sd) : 3.1 (0.9)<br>min ≤ med ≤ max:<br>1 ≤ 3 ≤ 5<br>IQR (CV) : 1 (0.3)	1 : 40 (3.7%)<br>2 : 180 (16.9%)<br>3 : 508 (47.6%)<br>4 : 279 (26.1%)<br>5 : 60 (5.6%)		1067<br>(100.0%)	0<br>(0.0%)
12	Q12<br>[numeric]	Mean (sd) : 3.3 (1.1)<br>min ≤ med ≤ max:<br>1 ≤ 3 ≤ 5<br>IQR (CV) : 2 (0.3)	1 : 70 (6.6%)<br>2 : 232 (21.7%)<br>3 : 271 (25.4%)<br>4 : 347 (32.5%)<br>5 : 147 (13.8%)		1067<br>(100.0%)	0<br>(0.0%)
13	Q13<br>[numeric]	Mean (sd) : 3.1 (1.1)<br>min ≤ med ≤ max:<br>1 ≤ 3 ≤ 5<br>IQR (CV) : 2 (0.3)	1 : 65 (6.1%)<br>2 : 266 (24.9%)<br>3 : 332 (31.1%)<br>4 : 310 (29.1%)<br>5 : 94 (8.8%)		1067<br>(100.0%)	0<br>(0.0%)
14	Q14<br>[numeric]	Mean (sd) : 3 (1)<br>min ≤ med ≤ max:<br>1 ≤ 3 ≤ 5<br>IQR (CV) : 2 (0.3)	1 : 60 (5.6%)<br>2 : 326 (30.6%)<br>3 : 343 (32.1%)<br>4 : 281 (26.3%)<br>5 : 57 (5.3%)		1067<br>(100.0%)	0<br>(0.0%)
15	Q15<br>[numeric]	Mean (sd) : 1.7 (0.9)<br>min ≤ med ≤ max:<br>1 ≤ 1 ≤ 4<br>IQR (CV) : 1 (0.5)	1 : 581 (54.5%)<br>2 : 316 (29.6%)<br>3 : 107 (10.0%)<br>4 : 63 (5.9%)		1067<br>(100.0%)	0<br>(0.0%)
16	Q16<br>[numeric]	Mean (sd) : 1.6 (0.7)<br>min ≤ med ≤ max:<br>1 ≤ 2 ≤ 4<br>IQR (CV) : 1 (0.4)	1 : 512 (48.0%)<br>2 : 462 (43.3%)<br>3 : 72 (6.7%)<br>4 : 21 (2.0%)		1067<br>(100.0%)	0<br>(0.0%)

No	Variable	Stats / Values	Freqs (% of Valid)	Graph	Valid	Missing
17	Q17 [numeric]	Mean (sd) : 2 (1.1)<br>min ≤ med ≤ max:<br>1 ≤ 2 ≤ 5<br>IQR (CV) : 2 (0.5)	1 : 452 (42.4%)<br>2 : 329 (30.8%)<br>3 : 185 (17.3%)<br>4 : 66 (6.2%)<br>5 : 35 (3.3%)	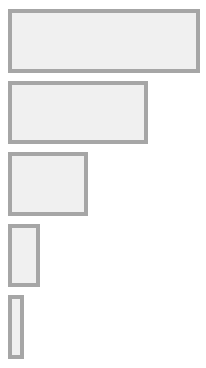	1067 (100.0%)	0 (0.0%)
18	Q18 [numeric]	Mean (sd) : 2 (0.9)<br>min ≤ med ≤ max:<br>1 ≤ 2 ≤ 4<br>IQR (CV) : 2 (0.4)	1 : 339 (31.8%)<br>2 : 417 (39.1%)<br>3 : 258 (24.2%)<br>4 : 53 (5.0%)	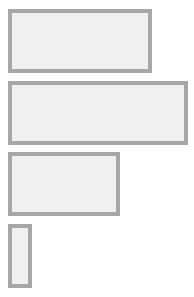	1067 (100.0%)	0 (0.0%)
19	Q19 [numeric]	Mean (sd) : 1.9 (0.8)<br>min ≤ med ≤ max:<br>1 ≤ 2 ≤ 4<br>IQR (CV) : 1 (0.4)	1 : 373 (35.0%)<br>2 : 457 (42.8%)<br>3 : 200 (18.7%)<br>4 : 37 (3.5%)	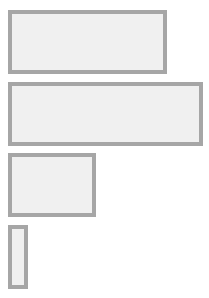	1067 (100.0%)	0 (0.0%)
20	Q20 [numeric]	Mean (sd) : 1.4 (0.6)<br>min ≤ med ≤ max:<br>1 ≤ 1 ≤ 3<br>IQR (CV) : 1 (0.4)	1 : 711 (66.6%)<br>2 : 282 (26.4%)<br>3 : 74 (6.9%)	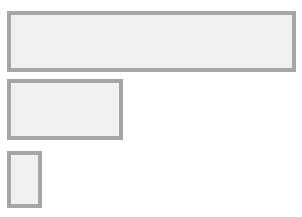	1067 (100.0%)	0 (0.0%)

Generated by summarytools (https://github.com/dcomtois/summarytools) 1.1.5 (R (https://www.r-project.org/) version 4.5.1)
2026-05-24

```
print(summarytools::dfSummary(dta2, graph.magnif = 0.75), method = "render")
```

Data Frame Summary

dta2

Dimensions: 201 x 18
Duplicates: 0

No	Variable	Stats / Values	Freqs (% of Valid)	Graph	Valid	Missing
1	ZTAS_Tot<br>[numeric]	Mean (sd) : 0 (1)<br>min ≤ med ≤ max:<br>-2.3 ≤ 0.1 ≤ 2.6<br>IQR (CV) : 1.4<br>(-1.787654e+15)	45 distinct values	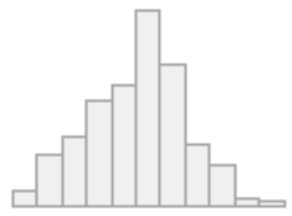	201<br>(100.0%)	0<br>(0.0%)
2	ZTAS_DIF<br>[numeric]	Mean (sd) : 0 (1)<br>min ≤ med ≤ max:<br>-2 ≤ -0.1 ≤ 2.6<br>IQR (CV) : 1.4<br>(1.347059e+15)	27 distinct values	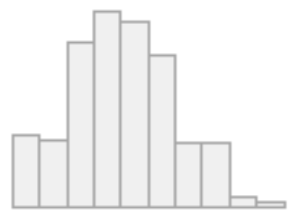	201<br>(100.0%)	0<br>(0.0%)
3	ZTAS_DDF<br>[numeric]	Mean (sd) : 0 (1)<br>min ≤ med ≤ max:<br>-2.3 ≤ -0.1 ≤ 2.1<br>IQR (CV) : 1.5<br>(-5.32015e+14)	21 distinct values	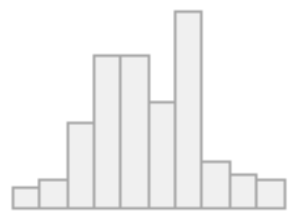	201<br>(100.0%)	0<br>(0.0%)
4	ZTAS_EOT<br>[numeric]	Mean (sd) : 0 (1)<br>min ≤ med ≤ max:<br>-2.9 ≤ 0 ≤ 2.7<br>IQR (CV) : 1.4<br>(-2.596554e+15)	22 distinct values	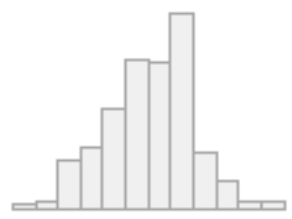	201<br>(100.0%)	0<br>(0.0%)
5	ZHSPS_Tot<br>[numeric]	Mean (sd) : 0 (1)<br>min ≤ med ≤ max:<br>-3 ≤ 0.1 ≤ 3.2<br>IQR (CV) : 1.3<br>(-4.264893e+14)	81 distinct values	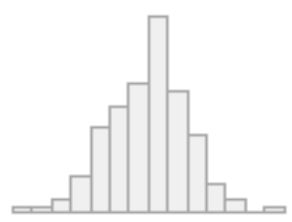	201<br>(100.0%)	0<br>(0.0%)
6	ZHSPS_EOE<br>[numeric]	Mean (sd) : 0 (1)<br>min ≤ med ≤ max:<br>-3.1 ≤ 0.1 ≤ 2.6<br>IQR (CV) : 1.3<br>(-7.37453e+14)	50 distinct values	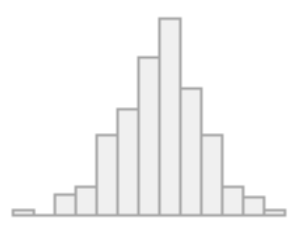	201<br>(100.0%)	0<br>(0.0%)
7	ZHSPS_AES<br>[numeric]	Mean (sd) : 0 (1)<br>min ≤ med ≤ max:<br>-2.3 ≤ 0.1 ≤ 2.3<br>IQR (CV) : 1.3<br>(1.917339e+15)	30 distinct values	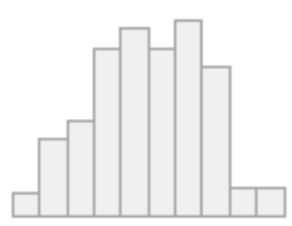	201<br>(100.0%)	0<br>(0.0%)
8	ZHSPS_LST<br>[numeric]	Mean (sd) : 0 (1)<br>min ≤ med ≤ max:<br>-1.7 ≤ 0 ≤ 3<br>IQR (CV) : 1.4<br>(-3.303735e+15)	35 distinct values	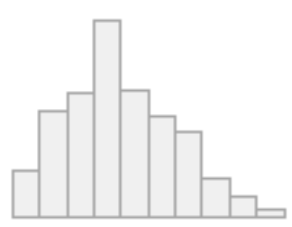	201<br>(100.0%)	0<br>(0.0%)

No	Variable	Stats / Values	Freqs (% of Valid)	Graph	Valid	Missing
9	ZOS_Tot<br>[numeric]	Mean (sd) : 0 (1)<br>min ≤ med ≤ max:<br>-2.6 ≤ 0 ≤ 2.8<br>IQR (CV) : 1.4<br>(-1.55004e+14)	47 distinct values	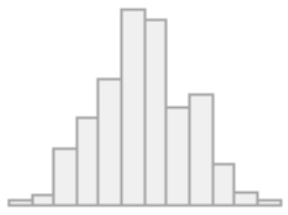	201<br>(100.0%)	0<br>(0.0%)
10	ZOS_NPS<br>[numeric]	Mean (sd) : 0 (1)<br>min ≤ med ≤ max:<br>-2.8 ≤ -0.1 ≤ 2.7<br>IQR (CV) : 1.7<br>(-1.56073e+15)	22 distinct values	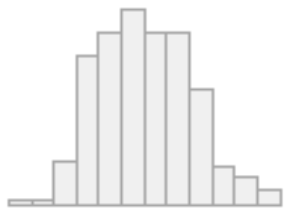	201<br>(100.0%)	0<br>(0.0%)
11	ZOS_APS<br>[numeric]	Mean (sd) : 0 (1)<br>min ≤ med ≤ max:<br>-3 ≤ 0 ≤ 2.1<br>IQR (CV) : 1.3<br>(-1.131529e+16)	26 distinct values	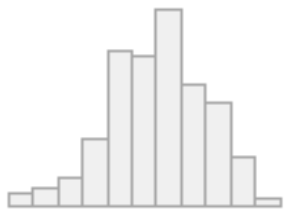	201<br>(100.0%)	0<br>(0.0%)
12	ZOS_AS<br>[numeric]	Mean (sd) : 0 (1)<br>min ≤ med ≤ max:<br>-2.8 ≤ -0.1 ≤ 2.1<br>IQR (CV) : 1.4<br>(5.134563e+14)	24 distinct values	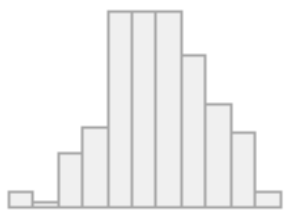	201<br>(100.0%)	0<br>(0.0%)
13	ZAASP_Seek<br>[numeric]	Mean (sd) : 0 (1)<br>min ≤ med ≤ max:<br>-2.5 ≤ 0.1 ≤ 2.4<br>IQR (CV) : 1.5<br>(5.176773e+14)	35 distinct values	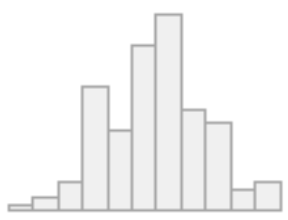	201<br>(100.0%)	0<br>(0.0%)
14	ZAASP_Sen<br>[numeric]	Mean (sd) : 0 (1)<br>min ≤ med ≤ max:<br>-2.6 ≤ -0.1 ≤ 3.2<br>IQR (CV) : 1.2<br>(-8.408951e+14)	38 distinct values	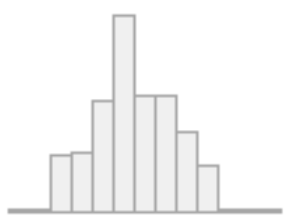	201<br>(100.0%)	0<br>(0.0%)
15	ZAASP_SA<br>[numeric]	Mean (sd) : 0 (1)<br>min ≤ med ≤ max:<br>-2.3 ≤ 0 ≤ 2.6<br>IQR (CV) : 1.4<br>(-7.996674e+14)	36 distinct values	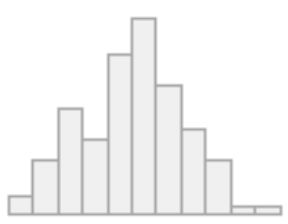	201<br>(100.0%)	0<br>(0.0%)
16	ZDASS_Dep<br>[numeric]	Mean (sd) : 0 (1)<br>min ≤ med ≤ max:<br>-1.4 ≤ -0.2 ≤ 3.4<br>IQR (CV) : 1.4<br>(-1.01997e+16)	20 distinct values	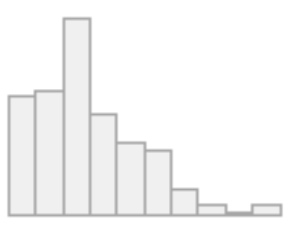	201<br>(100.0%)	0<br>(0.0%)

No	Variable	Stats / Values	Freqs (% of Valid)	Graph	Valid	Missing
17	ZDASS_Anx [numeric]	Mean (sd) : 0 (1)<br>min ≤ med ≤ max:<br>-1.6 ≤ -0.1 ≤ 3.1<br>IQR (CV) : 1.6 (-1.386253e+15)	19 distinct values	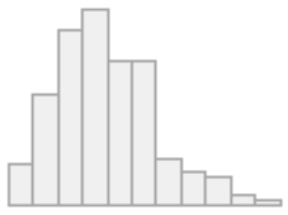	201 (100.0%)	0 (0.0%)
18	ZDASS_Str [numeric]	Mean (sd) : 0 (1)<br>min ≤ med ≤ max:<br>-1.9 ≤ 0 ≤ 2.7<br>IQR (CV) : 1.4 (-3.365534e+15)	18 distinct values	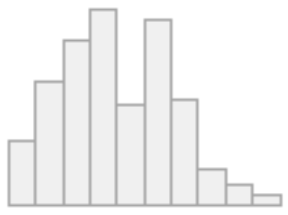	201 (100.0%)	0 (0.0%)

Generated by summarytools (https://github.com/dcomtois/summarytools) 1.1.5 (R (https://www.r-project.org/) version 4.5.1)
2026-05-24

```
print(summarytools::dfSummary(dta3, graph.magnif = 0.75), method = "render")
```

Data Frame Summary

dta3

Dimensions: 327 x 8
Duplicates: 0

No	Variable	Stats / Values	Freqs (% of Valid)	Graph	Valid	Missing
1	zCEQExpectancy_Positive [numeric]	Mean (sd) : 0 (0.9)<br>min ≤ med ≤ max:<br>-3.1 ≤ 0.1 ≤ 2.3<br>IQR (CV) : 1.1 (36.1)	57 distinct values	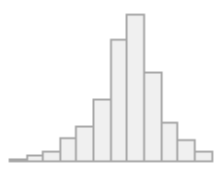	327 (100.0%)	0 (0.0%)
2	zCEQExpectancy_Negative [numeric]	Mean (sd) : 0 (1)<br>min ≤ med ≤ max:<br>-1.7 ≤ -0.1 ≤ 2.7<br>IQR (CV) : 1.4 (-1125363921)	61 distinct values	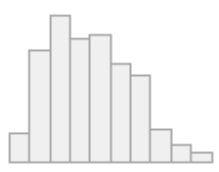	327 (100.0%)	0 (0.0%)

No	Variable	Stats / Values	Freqs (% of Valid)	Graph	Valid	Missing
3	zMMM_Coping [numeric]	Mean (sd) : 0 (1)<br>min ≤ med ≤ max:<br>-1.6 ≤ 0 ≤ 2.1<br>IQR (CV) : 1.4 (286.1)	17 distinct values	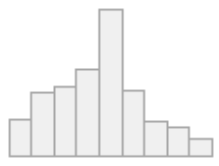	327 (100.0%)	0 (0.0%)
4	zMMM_Enhancement [numeric]	Mean (sd) : 0 (1)<br>min ≤ med ≤ max:<br>-3.1 ≤ 0.1 ≤ 1.2<br>IQR (CV) : 1.6 (78.8)	17 distinct values	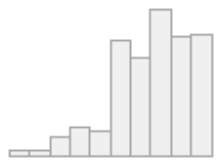	327 (100.0%)	0 (0.0%)
5	zMMM_Social [numeric]	Mean (sd) : 0 (1)<br>min ≤ med ≤ max:<br>-1.7 ≤ 0 ≤ 2.4<br>IQR (CV) : 1.4 (194.1)	22 distinct values	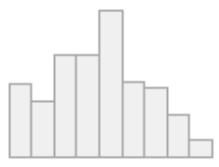	327 (100.0%)	0 (0.0%)
6	zMMM_Conformity [numeric]	Mean (sd) : 0 (1)<br>min ≤ med ≤ max:<br>-0.7 ≤ -0.7 ≤ 4.2<br>IQR (CV) : 1.2 (-241.9)	13 distinct values	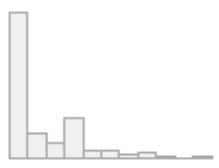	327 (100.0%)	0 (0.0%)
7	zMMM_Expansion [numeric]	Mean (sd) : 0 (1)<br>min ≤ med ≤ max:<br>-1.4 ≤ -0.1 ≤ 2.1<br>IQR (CV) : 1.8 (-568.5)	22 distinct values	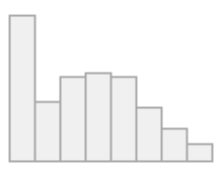	327 (100.0%)	0 (0.0%)

No	Variable	Stats / Values	Freqs (% of Valid)	Graph	Valid	Missing
8	zMMM_Routine [numeric]	Mean (sd) : 0 (1)<br>min ≤ med ≤ max:<br>-1.4 ≤ 0 ≤ 2.2<br>IQR (CV) : 1.8 (607.4)	-1.37! : 58 (17.7%)<br>-0.93! : 33 (10.1%)<br>-0.49! : 49 (15.0%)<br>-0.05! : 44 (13.5%)<br>0.39! : 54 (16.5%)<br>0.83! : 34 (10.4%)<br>1.27! : 27 (8.3%)<br>1.71! : 16 (4.9%)<br>2.15! : 12 (3.7%)<br>!rounded		327 (100.0%)	0 (0.0%)

Generated by summarytools (https://github.com/dcomtois/summarytools) 1.1.5 (R (https://www.r-project.org/) version 4.5.1)
2026-05-24

```
fviz_eig(prcomp(dta1  , center = TRUE, scale. = TRUE))
```

```
## Warning in geom_bar(stat = "identity", fill = barfill, color = barcolor, :
## Ignoring empty aesthetic: `width`.
```

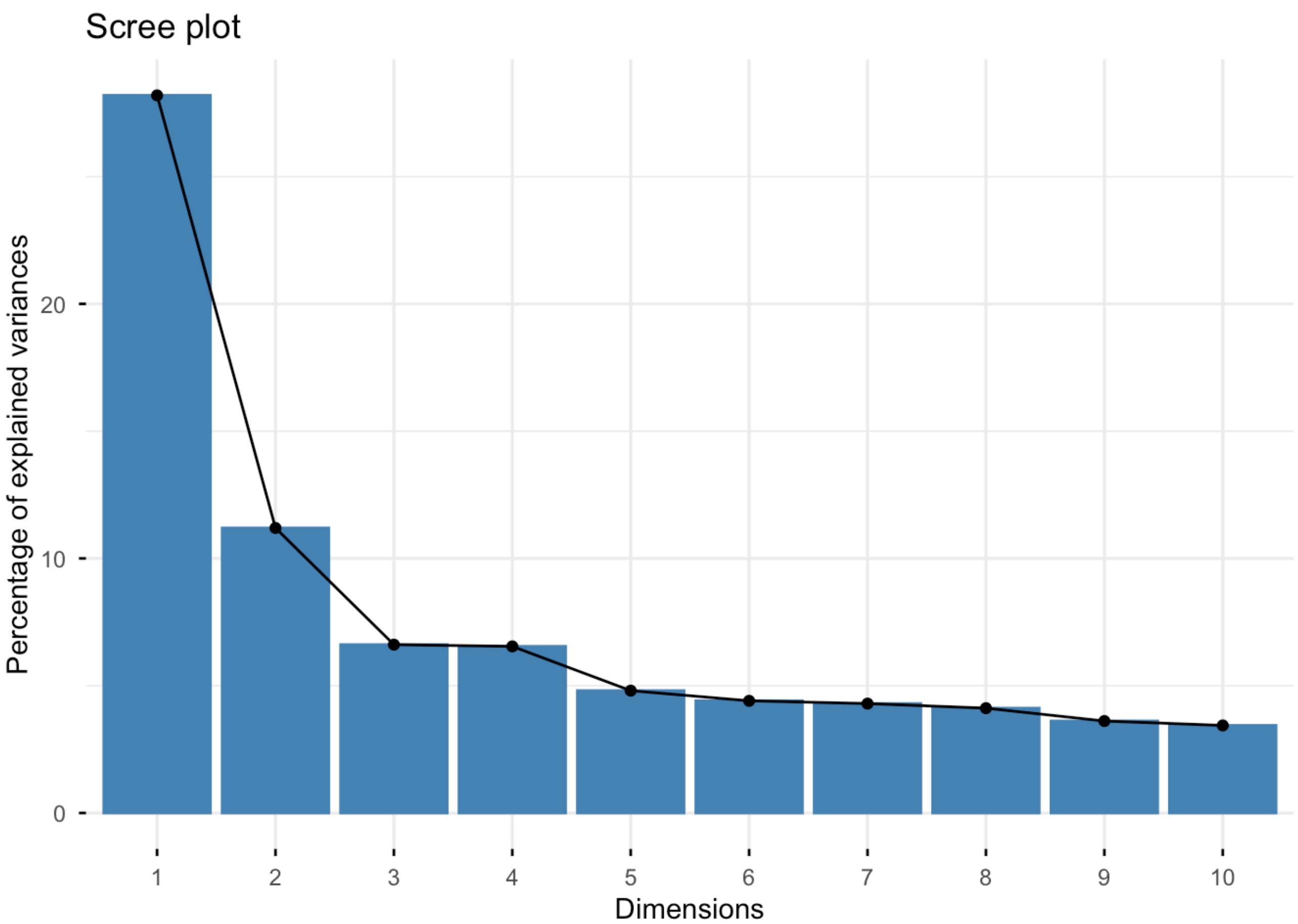


```
fviz_eig(prcomp(dta2 , center = TRUE, scale. = TRUE))
```

```
## Warning in geom_bar(stat = "identity", fill = barfill, color = barcolor, :
## Ignoring empty aesthetic: `width`.
```

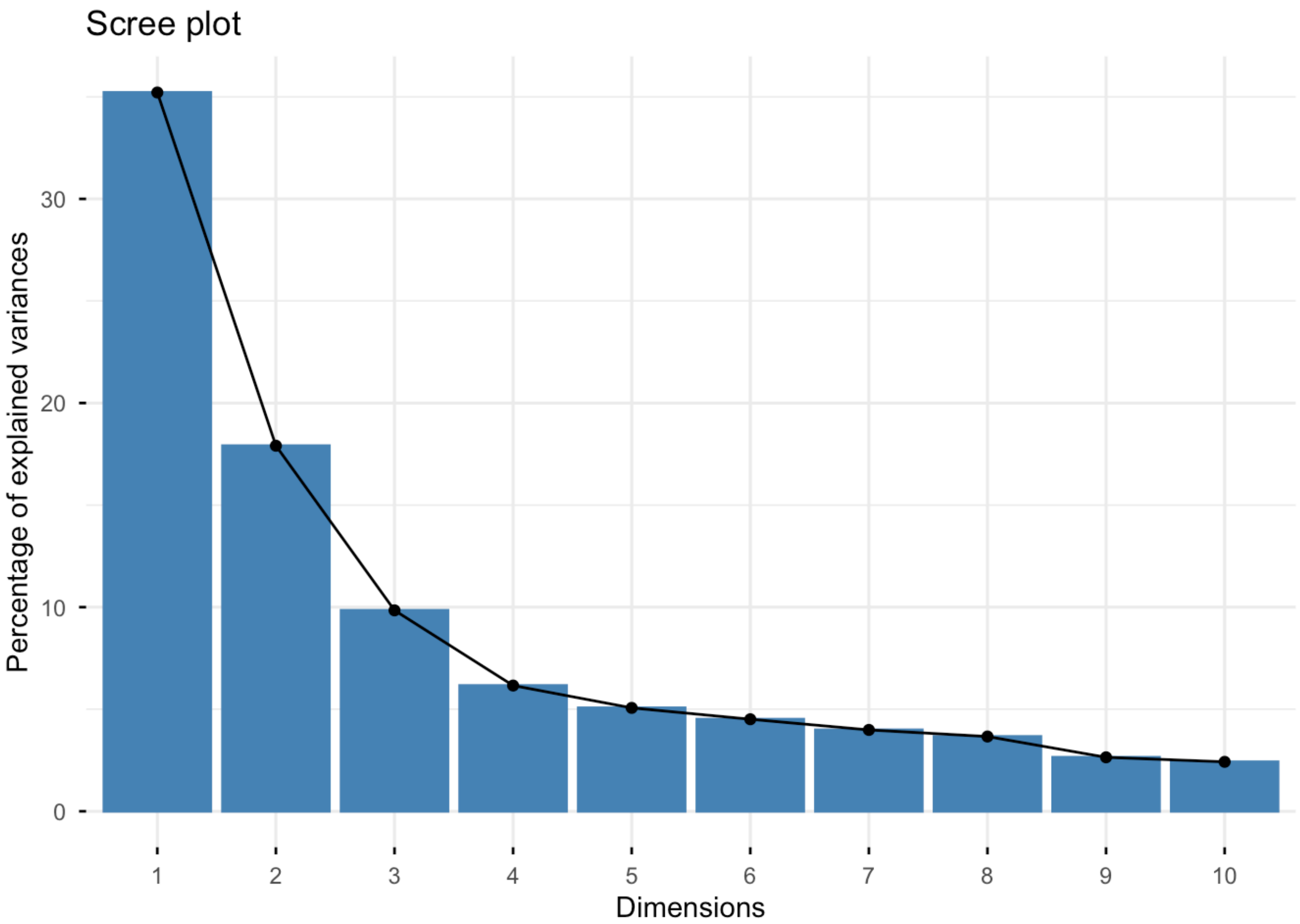


```
fviz_eig(prcomp(dta3 , center = TRUE, scale. = TRUE))
```

```
## Warning in geom_bar(stat = "identity", fill = barfill, color = barcolor, :
## Ignoring empty aesthetic: `width`.
```

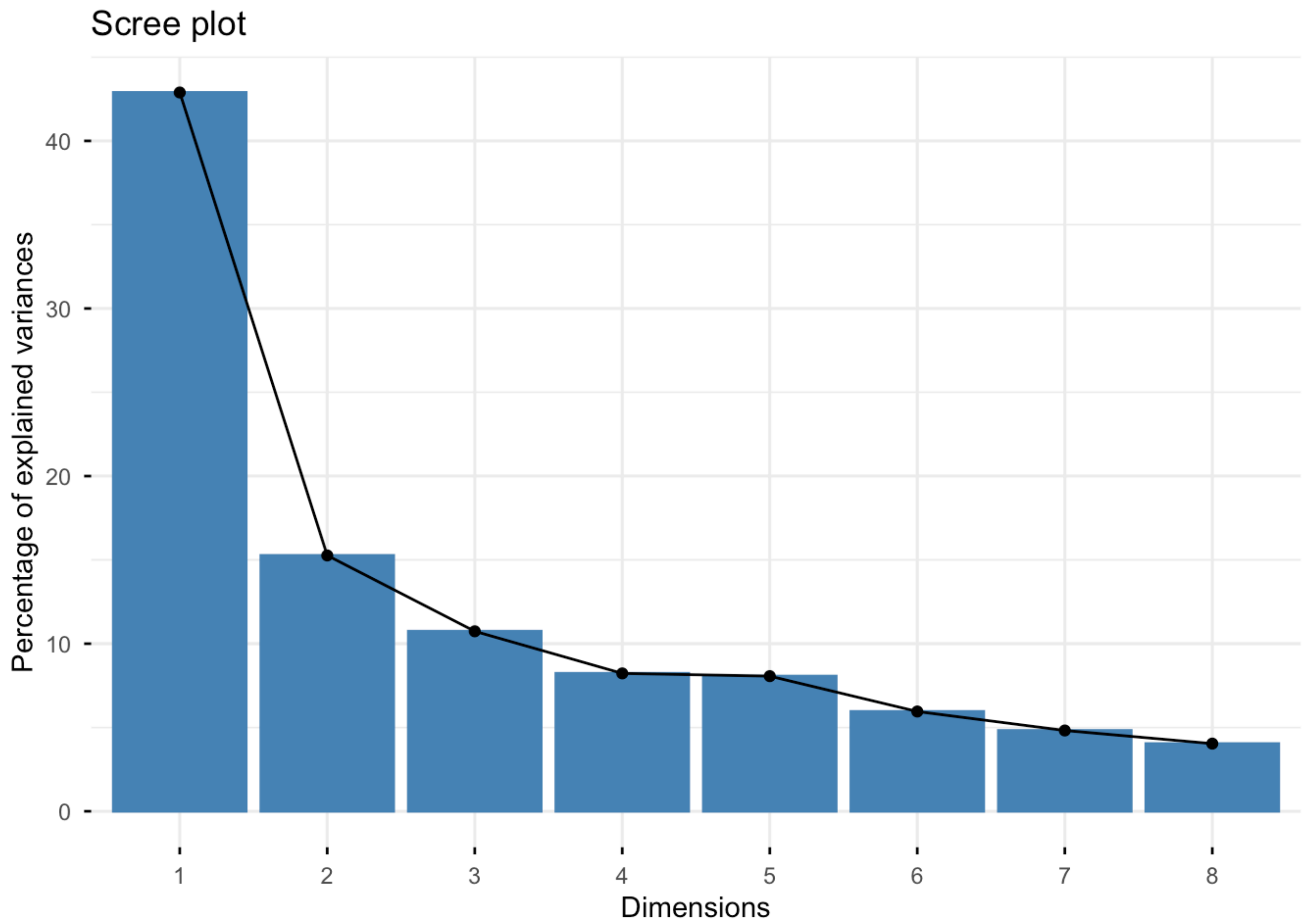

Generate simulated data

```
set.seed(12457643)
dta1<- gen_data(dta1,100)

set.seed(12457643)
dta2<- gen_data(dta2,100)

set.seed(12457643)
dta3<- gen_data(dta3,100)
```

Simulation results

```
set.seed(1234)
results_dta1 <- extract_results(dta1, 4, 8)

set.seed(1234)
results_dta2 <- extract_results(dta2, 2, 8)

set.seed(1234)
results_dta3 <- extract_results(dta3, 2, 5)
```

```
p1 <- plot_results(results_dta1, base = 0.625) + labs(tag = "(A)")
p2 <- plot_results(results_dta2, base = 0.5) + labs(tag = "(B)")
p3 <- plot_results(results_dta3, base = 0.5) + labs(tag = "(C)")
```

```
combined <- (p1 / p2 / p3) & theme(legend.position = "bottom")
combined + plot_layout(guides = "collect")
```

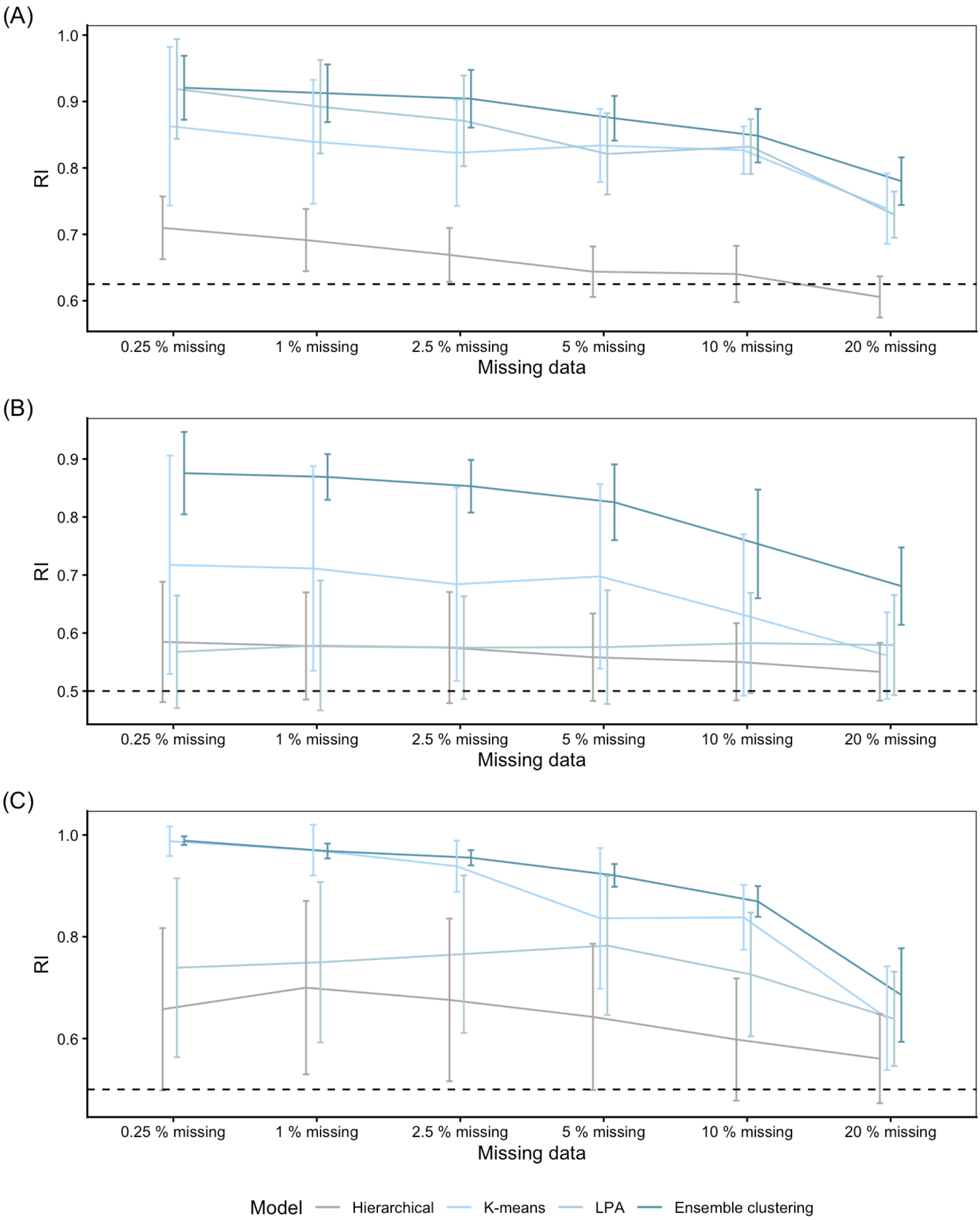

Supplement: Supplementary file 4 [file 13428_2026_3158_MOESM5_ESM.pdf]
