# Supplementary material for: Ensemble clustering: A practical tutorial
Source: Behav Res Methods. 2026 Sep 3;58(10):286. doi: 10.3758/s13428-026-03158-y (PMC13541929; doi:10.3758/s13428-026-03158-y)
Supplement: Supplementary file 5 [file 13428_2026_3158_MOESM1_ESM.docx]

Ensemble clustering: A practical tutorial

Supplementary Material I: Supplementary tables and figures

Table S1. Terminologies

| **Steps** | **Description** |
| --- | --- |
| Unsupervised learning | Unsupervised learning is a type of machine learning that identifies patterns in a dataset without reference to known or labelled outcomes. |
| "Ground truth" | "Ground truth" refers to the actual, real-world, known classification or properties of data points, against which the accuracy of predictive models or clustering algorithms can be measured. |
| The impossibility theorem for clustering | No clustering algorithm can simultaneously satisfy all three key properties: scale-invariance, richness, and consistency, highlighting the inherent trade-offs in designing and selecting clustering methods. |
| Model-based clustering | Model-based clustering assigns data points (observations) into clusters based on the assumption that the data is generated from a mixture of finite probability distributions, each representing a cluster. |
| Latent Class Analysis (LCA) | LCA is a model-based clustering method for categorical variables. |
| Latent Profile Analysis (LPA) | LPA is a model-based clustering method for continuous variables. |
| Centre-based partitioning clustering | Centre-based partitioning clustering is a method that divides data into clusters based on the proximity of data points to the centre of the cluster, typically using algorithms such as k-means where each cluster is defined by its central point. |
| Hierarchical clustering | Hierarchical clustering is a method of cluster analysis which seeks to build a hierarchy of clusters, either by successively merging smaller clusters into larger ones (agglomerative approach) or by progressively dividing a large cluster into smaller clusters (divisive approach), without requiring a predetermined number of clusters. |
| Density-based clustering | Density-based clustering is a type of clustering algorithm that identifies clusters as areas of higher density within the data space, separated by areas of lower density, allowing it to discover clusters of arbitrary shape without requiring a predefined number of clusters. |
| Graph-based clustering | Graph-based clustering is an approach that constructs a graph from the data, where data points are nodes and edges represent relationships or similarities between them, and then uses graph theory methods to identify clusters based on the connectivity and the structure of the graph. |
| Louvain algorithm | The Louvain algorithm is a popular modular-based graph clustering method, commonly used in network analysis. The model optimizes modularity by iteratively grouping nodes into communities to maximize the density of intra-community edges compared to inter-community edges. |
| Spectral clustering | Spectral clustering is a technique that uses the eigenvalues of a similarity matrix derived from the data to perform dimensionality reduction before clustering the data, effectively grouping points based on their connectivity in a graph representation |
| Walktrap algorithm | Walktrap algorithm is a community detection method that identifies clusters in a graph based on the idea that random walks tend to get trapped within densely connected parts of the graph, thus revealing community structure. |
| Meta-learning methods | Meta-learning methods are approaches in machine learning that aim to improve learning algorithms by integrating results from multiple models. This is commonly achieved by learning outputs from a variety of models and combine results. |
| Projected to different subspaces | Projected to different subspaces refers to the technique of transforming data into lower-dimensional spaces using various criteria or methods, to reveal underlying patterns, clusters, or simplify the data for analysis, with each projection emphasizing different aspects of the data. |
| Random-k | Random-k is a technique in ensemble clustering that randomly selects the number of clusters across BCs. |
| K-mode | K-mode is a clustering algorithm designed for categorical data, extending the k-means paradigm by replacing means with modes for centre calculations and using a different dissimilarity measure to handle categorical objects. |
| Binary cluster association matrix | A binary cluster association matrix is a two-dimensional matrix used in clustering analysis that represents the presence or absence of associations between pairs of observations, indicating whether each pair of observations belongs to the same cluster (1) or different clusters (0). |
| K-means-based Consensus clustering (KCC) | KCC is an ensemble clustering model that is scalable model for large-scale data, which applies k-means with generalized distance functions based on binary cluster association matrix (Wu et al., 2015). |
| Bipartite graph | Bipartite graph is a type of graph where nodes can be divided into two distinct sets such that every edge connects a vertex from one set to a vertex in the other set; no edge connects nodes within the same set. |
| HyperGraph-Partitioning Algorithm (HGPA) | HGPA is a graph-based consensus clustering model, which applies hypergraph partitioning package HMETI on hypergraph constructed from binary association matrix (Strehl and Ghosh, 2002) |
| Pairwise association | A pairwise association quantifies the relationship between pairs of observations in a dataset, typically indicating the degree of similarity or dissimilarity between each pair. |
| Co-association matrix | Co-association matrix, known as consensus matrix, is a type of pairwise association matrix measuring consensus of BCs. Each element in the matrix indicates the frequency or proportion of times two observations are placed in the same cluster across all BCs. |
| Evidence accumulation Clustering (EAC) | EAC is a consensus clustering method proposed by Fred & Jain (2002) which applies a single linkage hierarchical clustering on the co-association matrix. |
| Consensus clustering | Consensus clustering commonly refers to the model proposed by Monti et al. (2003), which applies the hierarchical clustering on the co-association matrix. |
| Weighted graph | Weighted graph is a type of graph with nodes connected by edges with numerical values as weight, representing quantities such as similarity and distance. |
| Hypergraph | A hypergraph is a generalisation of graph structure where edges, called hyperedges, can connect any number of nodes, allowing for the modelling of complex, many-to-many relationships. |
| Hyperedge | Hyperedges are elements of a hypergraph that generalise the concept of edges in a traditional graph. Unlike standard edges that connect exactly two nodes, hyperedges can connect any number of nodes. |
| Cluster-based Similarity Partitioning Algorithm (CSPA) | CSPA uses co-association matrix (as a distance measure) to construct weighted graph. Then graph partitioning algorithm METIS is used to partition the graph into subgraphs (Strehl & Ghosh, 2002) |
| Connected-triple-based similarity (CTS) | CTS is a similarity measure that evaluates not only the direct grouping of observation pairs within the same cluster but also assesses the degree of similarity between clusters when observations are placed into separate clusters (Iam-On et al., 2011) |
| Link-based Clustering Ensemble (LCE) | LCE is a method proposed by am-On et al. (2011), that enhances clustering consensus by evaluating and combining the linkage information (similarity between data points) across different clustering solutions to produce a more accurate and stable final clustering outcome. |
| Meta-Clustering Algorithm (MCLA) | MCLA is an ensemble clustering technique that treats each base clustering result as a hypergraph, where clusters are hyperedges, and employs a consensus function to merge similar hyperedges, effectively grouping the base clusters into a more coherent and robust final clustering solution (Strehl & Ghosh, 2002). |
| Adaptive Clustering Ensemble (ACE) | ACE (Alqurashi & Wang, 2019) is an iterative approach based on binary clustering association matrix. ACE iteratively aggregate the BCs that are most similar based on similarity between clusters until the intended candidate clusters are produced. A final refinement step will be conducted to address observations that remain uncertain. |
| Silhouette index | Silhouette index is a metric used to evaluate the quality of clustering by measuring how similar an object is to its own cluster compared to other clusters. It’s values ranging from -1 (indicating incorrect clustering) to +1 (indicating very tight clustering), where a high value signifies that the object is well matched to its own cluster and poorly matched to neighbouring clusters. |
| Calinski–Harabasz (CH) Index | CH Index cis a metric for evaluating clustering quality that measures the ratio of the sum of between-clusters dispersion to the sum of within-cluster dispersion, with higher values indicating better defined clusters. |
| Dunn index | Dunn index is a metric for evaluating clustering quality that measures the ratio between the smallest distance between observations in different clusters to the largest intra-cluster distance, with higher values indicating better clustering by maximizing inter-cluster separation and minimizing intra-cluster distance. |
| Gap index | Gap index is a statistical method for estimating the optimal number of clusters in a dataset by comparing the log within-cluster sum of squares (WSS) against a null reference distribution of the data, with the optimal clustering indicated by the maximum gap between the observed WSS and the expected WSS under the null distribution. |
| Principal Components Analysis (PCA) | PCA is a dimensionality reduction technique. It transforms a set of possibly correlated variables into a smaller number of uncorrelated variables called principal components, which preserves as much of the data's variation as possible. |
| Multidimensional Scaling (MDS) | MDS is a dimensionality reduction technique, that project high dimensional data down to low dimensions space, while preserving pairwise distances between observations |
| T-distributed Stochastic Neighbor Embedding (t-SNE) | T-SNE is a dimensionality reduction algorithm that is particularly well suited for the visualization of high-dimensional datasets by mapping them to a lower-dimensional space in a way that preserves the relative distances between points, making similar data points appear close to each other and dissimilar points appear far apart. |
| Nonnegative matrix factorization (NMF) | NMF is a computational technique used to decompose a nonnegative matrix into the product of two lower-rank nonnegative matrices, often used for feature extraction and dimensionality reduction in data analysis, revealing underlying patterns or components in the data. |
| Uniform Manifold Approximation and Projection (UMAP) | UMAP is a dimensionality reduction technique that constructs a high-dimensional graph representing the data and then optimizes a low-dimensional graph to be as structurally similar as possible, effectively preserving both local and global data structures in a lower-dimensional space. |
| Cophenetic correlation coefficient | Cophenetic correlation coefficient is a measure used to evaluate the quality of a hierarchical clustering, quantifying how faithfully the clustering dendrogram preserves the pairwise distances between the original unclustered data points. |
| External validity | External validity refers to the extent to which the results of a study can be generalized or applied to other settings, populations, or time periods beyond the specific conditions of the original experiment or study. |
| Multivariate Imputation by Chained Equations (MICE) | MICE is a statistical technique for imputing missing values in datasets by iteratively modelling each variable with missing data as a function of other variables in a round-robin fashion, creating multiple imputations to reflect the uncertainty around the missing data. |

**Figure S1.** Simple illustrations of a weighted graph, a bipartite graph and a hypergraph estimated from three Base Clusterings (BCs), $C_{1}, C_{2}, \mathrm{and} C_{3}$, each with two partitions. In weighted graphs, edges between pairs of nodes are assigned weights that represent the strength of connection (e.g., $C_{1}, C_{2}, \mathrm{and} C_{3}$, A and C were more frequently clustered into the same group compared with A and B). Bipartite graph are graphs with nodes divided into two distinct sets (observations and clustering labels), and connections are allowed only between sets, not within a set. Hypergraphs are graphs linking observations through hyperedges, where a single hyperedge can connect more than two nodes simultaneously, corresponding to the shared membership of a cluster.

| (A) |  |
| --- | --- |
| (B) |  |

**Figure S2** Popularity of R packages indicated by (A) CRAN and (B) Bioconductor download. Note: frequency of download does not necessarily indicate level of use, as it may reflect installations (e.g., due to updates) as well as their broader integration within computational ecosystems (e.g., package clue is used for diverse functions beyond clustering, which can inflate download counts relative to direct methodological use). In addition, CRAN and Bioconductor download statistics are not directly comparable, as they are derived from different infrastructure systems, use different logging and aggregation methods.

**Figure S3.** Potential Cross-Validation (CV) procedures with a nested inner–outer loop structure. The inner loop generate diversity to improve robust clustering solutions, while the outer loop facilitates understanding of broader stability of clustering results under different modelling specifications (e.g., BC models, consensus function, and number of clusters) collectively with stability indicators in the inner loops. This two-layered approach is useful as stability across individual BCs may be low when weak learners or heterogeneous models are used, while the ensemble solution may remain stable.

Table S2. Different types of validation strategies modified based on concepts introduced by Brucar et al. (2023)

| **Methods** | **Description** |
| --- | --- |
| Internal validation | The process evaluates how well the clustering solution captures the underlying structure of the data, using fit indices computed either on the full dataset or within a cross‑validation framework. |
| External validation | The process evaluates how well the clustering solution can be reproduced in an independent data set when the same analytical pipeline is applied. One way to do this is to simply compare the feature profiles of the clusters (e.g., distributions of source variables) obtained in the training and testing samples. Another approach is to build a prediction model on the training data (using source variables included in clustering model), apply it to the testing data, and then compare the predicted cluster assignments with the clusters that are obtained independently in the testing data. |
| Predictive validation | The process evaluates how well the clustering solution can be explained/predicted by variables that were not used the clustering model. This can be achieved using the original training data, testing the capacity of external variables for explaining the clustering solution. It can also be evaluated using a testing data using similar framework of external validation. |
| Translational validity | The process evaluates the meaningfulness of the clustering solution for real world settings (e.g., clinical decision‑making). In practice this can be evaluated by how well the clustering solutions can explain, predict, or modify outcomes (e.g., predating longitudinal trajectory of outcomes, explain treatment outcome heterogeneity). This can also be obtained via qualitative assessments with end users, e.g., interpretability and meaningfulness for clinical decision making. |

Table S3. Template for pre-registration of ensemble clustering models

| **Steps** | **Description** | **Example** |
| --- | --- | --- |
| Description of the data and variables | Details on the data source as well as individual variables collected and intended to be used in the clustering analysis. | The data will be generated from a theoretical model with the following procedures:   - generate a mixture of three multivariate normal distributions (with different means and variances) on a three-dimensional space representing three clusters with *n*=300, 150 and 150; - randomly project the original three-dimensional data onto a nine-dimensional space, creating a data set with 600 observations and 12 variables; and - add random errors to individual variables and additional outliers.   This dataset will be generated to demonstrate the processes of ensemble clustering and to validate the stability of ensemble clustering compared to traditional single-run clustering models. Additionally, a separate dataset was created by randomly removing cells to introduce missing data for further demonstration purposes. |
| Theory | Rationale/theory supporting clustering analysis, for example, the theory supporting existing heterogeneity and why the measured variables can be used to identify possible subgroups | Since the data was generated from a mixture of three multivariate normal distributions with some degree of separation, we expect that these clusters can be effectively identified using ensemble clustering models. The original data was randomly projected into higher-dimensional spaces, with additional noise introduced. All 12 variables will be used in the analysis. |
| Data pre-processing procedures | Any pre-processing procedures that can potentially be included to address issues such as high dimensionality, non-linearity, sparseness, missingness and outliers. Will any pre-clustering testing be conducted? | We will demonstrate how to:   - remove outliers using the 3×IQR rule; - conduct feature scaling to normalise the range of values across variables; - reduction dimensionality using methods such as Principal Component Analysis (PCA) and Multidimensional Scaling (MDS). Dimensions reduce to will be determined by variance explained criteria (we will retain slightly more dimensions as suggested); and - demonstrate missing data imputation with chained random forests.   Since this is simulated data, no pre-clustering tests will be conducted. |
| Methods for generating BCs | Details in method(s) that will be used in generating BCs, including: (1) how source data for BCs will be generated, (2) what BC models will be used, (3) how BC models will be parameterised (e.g., number of clusters and number of iterations for k-means), and (4) how many BCs will be generated. | We will use the data for diverse demonstrations of the ensemble process. We will randomly sample of 80% of the original observations and 80% of the variables to demonstrate sampling methods for generating source data for BC. K-means, Gaussian Mixture Models, and hierarchical clustering will be used as BC models, as these methods are the most commonly used methods in the field. We will use up to 5 clusters in the BC generation process with 100 BCs generated for each BC method. |
| Consensus function | Description of type(s) of consensus function(s) to be used and any additional parameterisation (e.g., range of final number of clusters). | We will demonstrate the use of four types of consensus methods:   - majority voting; - methods based on consensus matrix, e.g., spectral clustering, Local Cluster Ensemble (LCE), and GMM; and - methods based on Binary encoding clustering, e.g., k-means. |
| Selecting modelling parameters | Procedures on how to select key parameters, e.g., optimal number of clusters. | Optimal number of clusters will be chosen based on consensus matrix plot, the empirical cumulative distribution function (CDF) of the consensus matrix as well as Proportion of Ambiguous Clustering (PAC) score as recommended by Monti et al. (2003) and Șenbabaoğlu et al. (2014)  We will also demonstrate a permutation test, as suggested in Dwyer et al. (2022). |
| Post-clustering procedures | Visual representation of clustering results. Evaluation of cluster meaningfulness based on source data as well as additional variables that were not included in clustering models. Any procedures for testing generalisability of the results if required, e.g., whether similar clusters can be obtained in an external validation sample. | As this is a tutorial and with generated data, visualisation and external validation will not be conducted. |
| Methods for implementation | Computer program(s), package(s), and function(s) if possible | We will use multiple packages and functions including:   - from stats package functions for dimensionality reduction, e.g., prcomp and cmdscale; - functions from diceR package for main ensembel clustering models, e.g., diceR, consensus_cluster, majority_voting, graph_heatmap, and LCE; - indiviudal clustering model will also be sourced from relevent packages, e.g., spectralClustering from RclusTool package and kmeans from stats package; - alternative models using similar framework, e.g., ConsensusClusterPlus function from ConsensusClusterPlus package; and - missRanger from missRanger package for missing data imputation |
| Sensitivity analysis | Additional sensitivity analysis that uses different model(s) and/or sub-samples of the data. | We will not include sensitivity analysis as this is a practical tutorial. |

Table S3. Description of the data from published studies

| **Study** | **Description** |
| --- | --- |
| Cotton et al. (2022) | **Study**: Data were collected from 1067 young people (aged 12-25 years) accessing *headspace* services in Australia to evaluate underlying subgroups reporting different types of quality of life.  **Measure**: AQoL-6D is used to measure quality of life which includes 20 items on Likert scale.  **Model**: Latent Classes Analysis (LCA)  **Results**: the study identified 4 distinct clusters with different types and severity in impairment of quality of life.  **Link to source data**: https://github.com/ready4-dev/heterodox |
| Jakobson and Rigby (2021) | **Study**: Data were collected from 201 young adults to examine interrelationships between alexithymia, SPS, and sensory processing styles and distinguish the subtypes of alexithymia.  **Measure**: The TAS-20, comprising three subscales: DIF (7 items), DDF (5 items), and EOT (8 items), is used to measure alexithymia, with a 5-point Likert scale for each item. The HSPS, which includes EOE, LST, and AES, along with the OS scale, assesses SPS. The 15-item OS scale, consisting of NPS, APS, and AS, uses a 7-point Likert scale for each item. The DASS-21, which measures levels of depression, anxiety, and stress, employs a 4-point Likert scale for each item. The AASP, measuring LR, Seek, Sen, and SA, utilizes a 5-point Likert scale for each item.  **Results**: The study revealed five distinct classes of individuals, distinguished by the relative strength of different alexithymia traits and differences in interoceptive accuracy and sensory processing styles.  **Link to source data**: https://doi.org/10.34990/FK2/603TSA |
| Amiet et al. (2020) | **Study**: Data were collected from 329 people who used cannabis at least weekly, to investigate the mental health and behavioural correlates of differences in their motives and expectations for cannabis use.  **Measure**: The 24-item Extended-MMM is used to measure various motivations for cannabis use. Cannabis-related consequences are assessed using the 45-item CEQ. The 42-item CAPE evaluate positive (20 items) and negative (14 items) psychotic experiences, and depressive symptoms (8 items). Symptoms of depression, anxiety, and stress are measured by the DASS-21. The 18-item Apathy Evaluation Scale assess levels of apathy. The 16-item CUPIT is employed to detect problematic cannabis use. Illicit substance abuse is used to measure using the 10-item DAST with a 'Yes' or 'No' response format. The 6-item FTND assess nicotine dependence, and the 10-item AUDIT evaluate alcohol dependence and harmful drinking consequences.  **Model**: Latent Classes Analysis (LCA)  **Results**: The study identified two distinct clusters among regular cannabis users. One cluster, characterized by low motives and expectancies, showed lower engagement in cannabis-related activities and fewer symptoms. The other, with high motives and expectancies, was associated with more significant cannabis use, a higher likelihood of cannabis use disorder, and greater symptoms of psychosis, depression, anxiety, and stress.  **Link to source data**: https://www.frontiersin.org/journals/psychiatry/articles/10.3389/fpsyt.2020.599365/full#supplementary-material |

**Reference**

Amiet, D., Youssef, G. J., Hagg, L. J., Lorenzetti, V., Parkes, L., Solowij, N., & Yücel, M. (2020). Young Adults With Higher Motives and Expectancies of Regular Cannabis Use Show Poorer Psychosocial Functioning. *Front Psychiatry*, *11*, 599365. <https://doi.org/https://doi.org/10.3389/fpsyt.2020.599365>

Brucar, L. R., Feczko, E., Fair, D. A., & Zilverstand, A. (2023). Current Approaches in Computational Psychiatry for the Data-Driven Identification of Brain-Based Subtypes. *Biological Psychiatry*, *93*(8), 704–716. <https://doi.org/10.1016/j.biopsych.2022.12.020>

Cotton, S. M., Hamilton, M. P., Filia, K., Menssink, J. M., Engel, L., Mihalopoulos, C., Rickwood, D., Hetrick, S. E., Parker, A. G., Herrman, H., Telford, N., Hickie, I., McGorry, P. D., & Gao, C. X. (2022). Heterogeneity of quality of life in young people attending primary mental health services. *Epidemiol Psychiatr Sci*, *31*, e55. <https://doi.org/https://doi.org/10.1017/s2045796022000427>

Dwyer, D. B., Buciuman, M.-O., Ruef, A., Kambeitz, J., Sen Dong, M., Stinson, C., Kambeitz-Ilankovic, L., Degenhardt, F., Sanfelici, R., Antonucci, L. A., Lalousis, P. A., Wenzel, J., Urquijo-Castro, M. F., Popovic, D., Oeztuerk, O. F., Haas, S. S., Weiske, J., Hauke, D., Neufang, S.,…Consortium, P. (2022). Clinical, Brain, and Multilevel Clustering in Early Psychosis and Affective Stages. *JAMA Psychiatry*, *79*(7), 677–689. <https://doi.org/https://doi.org/10.1001/jamapsychiatry.2022.1163>

Jakobson, L. S., & Rigby, S. N. (2021). Alexithymia and Sensory Processing Sensitivity: Areas of Overlap and Links to Sensory Processing Styles. *Frontiers in Psychology*, *12*. <https://doi.org/https://doi.org/10.3389/fpsyg.2021.583786>

Monti, S., Tamayo, P., Mesirov, J., & Golub, T. (2003). Consensus Clustering: A Resampling-Based Method for Class Discovery and Visualization of Gene Expression Microarray Data. *Machine Learning*, *52*(1), 91–118. <https://doi.org/https://doi.org/10.1023/A:1023949509487>

Șenbabaoğlu, Y., Michailidis, G., & Li, J. Z. (2014). Critical limitations of consensus clustering in class discovery. *Scientific Reports*, *4*(1), 6207. <https://doi.org/https://doi.org/10.1038/srep06207>
